# A systematic review on the relationship between socioeconomic conditions and emotional disorder symptoms during Covid-19: unearthing the potential role of economic concerns and financial strain

**DOI:** 10.1186/s40359-024-01715-8

**Published:** 2024-04-26

**Authors:** Jee Kei Chan, Aleya A. Marzuki, Samira Vafa, Arjun Thanaraju, Jie Yap, Xiou Wen Chan, Hanis Atasha Harris, Khushi Todi, Alexandre Schaefer

**Affiliations:** 1 Department of Psychology, Monash University Malaysia, Jalan Lagoon Selatan, 47500 Bandar Sunway, Petaling Jaya, Selangor Darul Ehsan Malaysia; 2 Department of Psychology, Sunway University Malaysia, Jalan Universiti, No 5, 47500 Bandar Sunway, Petaling Jaya, Selangor Darul Ehsan Malaysia; 3 Sunway University Malaysia, Room: 4-4-11, Jalan Lagoon Selatan, Bandar Sunway, Petaling Jaya, 47500 Selangor Malaysia

**Keywords:** Socioeconomic conditions, Social determinants, Mental health, Emotional health, Depression, Anxiety

## Abstract

**Background:**

Covid-19 has disrupted the lives of many and resulted in high prevalence rates of mental disorders. Despite a vast amount of research into the social determinants of mental health during Covid-19, little is known about whether the results are consistent with the social gradient in mental health. Here we report a systematic review of studies that investigated how socioeconomic condition (SEC)—a multifaceted construct that measures a person’s socioeconomic standing in society, using indicators such as education and income, predicts emotional health (depression and anxiety) risk during the pandemic. Furthermore, we examined which classes of SEC indicators would best predict symptoms of emotional disorders.

**Methods:**

Following PRISMA guidelines, we conducted search over six databases, including Scopus, PubMed, etc., between November 4, 2021 and November 11, 2021 for studies that investigated how SEC indicators predict emotional health risks during Covid-19, after obtaining approval from PROSPERO (ID: CRD42021288508). Using Covidence as the platform, 362 articles (324 cross-sectional/repeated cross-sectional and 38 longitudinal) were included in this review according to the eligibility criteria. We categorized SEC indicators into ‘actual versus perceived’ and ‘static versus fluid’ classes to explore their differential effects on emotional health.

**Results:**

Out of the 1479 SEC indicators used in these 362 studies, our results showed that 43.68% of the SEC indicators showed ‘expected’ results (i.e., higher SEC predicting better emotional health outcomes); 51.86% reported non-significant results and 4.46% reported the reverse. Economic concerns (67.16% expected results) and financial strains (64.16%) emerged as the best predictors while education (26.85%) and living conditions (30.14%) were the worst.

**Conclusions:**

This review summarizes how different SEC indicators influenced emotional health risks across 98 countries, with a total of 5,677,007 participants, ranging from high to low-income countries. Our findings showed that not all SEC indicators were strongly predictive of emotional health risks. In fact, over half of the SEC indicators studied showed a null effect. We found that perceived and fluid SEC indicators, particularly economic concerns and financial strain could best predict depressive and anxiety symptoms. These findings have implications for policymakers to further understand how different SEC classes affect mental health during a pandemic in order to tackle associated social issues effectively.

**Supplementary Information:**

The online version contains supplementary material available at 10.1186/s40359-024-01715-8.

## Background

Covid-19, caused by the acute respiratory syndrome coronavirus 2 (SARS-CoV-2), was first discovered in December 2019 in the Wuhan city of China. The World Health Organization (WHO) first declared the outbreak a Public Health Emergency of International Concern on January 30, 2020 [1] and, soon after, a pandemic on March 11, 2020 [1]. In addition to collective fear of the virus exacerbated by its high infectiousness and growing death rate, emergence of the Covid-19 pandemic also led to a worldwide socioeconomic crisis [2, 3]. Many countries were forced to implement movement restrictions and instantaneous lockdown measures to contain the virus and doing so has greatly crippled the global economy [4]. Thus, Covid-19 has emerged as a common stressor to all, as it affected businesses, trades, and production of goods, which has consequently affected the income of a large number of individuals [5].

Covid-19 has been recognized as the worst pandemic of the century, in terms of scale and infection rate [6], and has profoundly impacted people’s mental health [7]. Given this, it is of great relevance to consider whether the social gradient in mental health would continue to be shown in a crisis of such magnitude. Defined as an inverse linear relationship between one’s socioeconomic status and/or conditions and mental health status, the social gradient in mental health theory posits that an individuals’ mental health follows a gradient that is in-line with his or her socioeconomic position in society, and such a relationship exists along a continuum [8]. Indeed, the relationship between SEC and mental health has been well-documented (e.g., [9–11]), with studies reporting moderate-to-strong associations between socioeconomic standing and subjective well-being and/or mental health (e.g., [12–15]). However, few have investigated whether specific SEC indicators are more predictive of mental health conditions over others [16].

### Social Economic Conditions (SEC) Indicators

SEC has been defined as an umbrella concept that encompasses both actual (objective) and perceived (subjective) status of a person or a group in a given social context [17]. This should include different facets, such as economic, education, occupation [18], and subjective self-evaluation [19]: (a) economic here refers to traditional material metrics, such as income and assets, which should include both individually- and family-owned (e.g., household income, family assets etc.); (b) education typically refers to years of education attained by an individuals or their parents; (c) occupation is used to reflect the complexity and the intellectual demands of jobs held [17]; and (d) self-evaluated SEC, measured using tools such as the MacArthur Scale of Subjective Social Status (MSS) [19], relies on individuals’ self-assessment of their socioeconomic standing in the context of their countries or communities.

However, even if SEC is defined as an overarching concept that includes multiple components, many SEC indicators (e.g., income, education, occupation etc.) do not seem to correlate strongly with one another [20]. Using the example provided in Farah [20], a plumber may not have attained as high an education level as an adjunct professor, but it is the plumber who could be earning a much higher income due to the shortage in this profession. A similar situation can be seen in traditional business owners who may not be highly educated, but could be earning much more than the average population. Thus, it is not surprising that past surveys have found a correlation of only between 0.2 and 0.7 (generally below 0.5) among SEC measures such as income, education, and occupation [21, 22].

In relation, studies have suggested that different SEC indicators are separate, standalone constructs that represent different dimensions of one’s socioeconomic position in society [23]. Relevant to our review, different SEC indicators exhibit differential effects on our emotional health. For instance, higher education (typically viewed as a proxy for good socioeconomic standing) has been linked to higher depressive symptoms, while the opposite was shown for income [24]. Hence, although SEC indicators may overlap, it is valuable for them to be investigated as separate variables to better elucidate their unique effects on emotional health. To-date, it is equivocal as to whether there is a specific SEC measure or cluster of measures that best predicts changes in emotional health, and hence this warrants further investigation, especially with the unique contextual opportunity brought about by Covid-19 as a natural global stressor.

#### Actual versus perceived SEC indicators

The basic tenet of social inequalities of mental health is the consequences of an uneven distribution of resources across social domains [25]. The fact that the social gradient in health is so robustly observed for a wide range of mental and physical health outcomes [26, 27] and has persisted since the early nineteenth century [28] across both developing and developed nations [29] suggests that the ‘fundamental’ cause of health inequalities is due to SEC disparities [30].

‘Resources’, referred to in the theory of fundamental causes, include tangible material possessions (e.g., wealth, income, assets, social capitals), and intangible ones (e.g., knowledge, power, prestige), which are disproportionately owned by the upper economic classes (e.g., [31–34]). More importantly, these resources are deemed to be ‘flexible” in that individuals can utilize them in “different ways and in different situations” [30 pS29]. For example, elite individuals have the privilege of choosing world-class treatment for psychiatric conditions, even if that means travelling overseas, and moreover high SEC individuals in positions of power can choose to reduce their workloads (or change jobs for the matter) if they feel that their mental health has been compromised by their work environment. All these privileges and flexibilities endowed by possession of key resources are posited to be the reason for the existence of the social gradient in mental health.

However, studies have shown that in addition to the ‘actual’ possession, the ‘perceived’ lack of such SEC resources could also play a role in the social inequalities in health [35, 36]. Numerous studies using various form of perceived SEC indicators, such as *financial threat* [37], *debt stress* [38], and *money-management stress* [39], *perceived financial strain* [40], have reported that such perceived financial well-being indicators could affect subjective well-being and mental health. More importantly, recent studies have provided evidence that such financial well-being indicators could potentially mediate the relationship between actual SEC indicators and emotional health [37, 39].

Although actual and perceived SEC indicators are interrelated, as individuals from low SEC backgrounds are more likely to have more concerns about their financial situations [41, 42], there are studies reporting otherwise. For example, individuals from objectively high-SEC backgrounds may still perceive themselves as ‘poor’ [43]. Additionally, there are individuals who do not consider themselves poor despite actually being objectively low in SEC as indicated by traditional income or asset-based measures [44]. In a study by Wang et al. [45] in rural China, 29% of households perceived and reported feeling poor even though they do not meet the objective criteria for poverty. Interestingly, a study by Chang et al. [46] which investigated 1,605 households in Hong Kong, showed that while only 29.06% of the respondents meet the criteria as living below the poverty line, more than 50% of them perceived themselves as poor.

Thus, in this review, we investigated how ‘actual’ and ‘perceived’ SEC categories may be differentially associated with emotional health symptoms in the context of Covid-19.

#### Static versus fluid SEC indicators

Aside from objectivity of one’s socioeconomic position, it may also be important to compare SEC indicators that are either stagnant or change over time. Past studies have showed that negative changes to one’s socioeconomic position could affect mental health [47, 48]. As a matter fact, the socioeconomic disruptions following disasters, be it man-made or natural, have been shown to be detrimental to mental well-being, as the financial disturbances would result in stress escalation, leading to various mental health issues, such as depression and anxiety [49]. More importantly, such negative consequences are usually more prominent in the low SEC population, as they are likely to lack resources that are needed to cope with the changes following crisis [49].

However, not all SEC indicators are capable to reflect such changes. For instance, education and occupation class are relatively time-invariant and may remain static even in a global health crisis while variables such as income are subject to change. More importantly, research typically compared low and high SEC between individuals, but few have explored how individual’s changes in SEC over time can impact mental well-being [50]. For the few studies which have investigated how changes in SEC influence mental health, the results were mixed.

First, Sareen et al. [47] showed that in addition to having a low income, a decline (i.e., change) in household income was significantly related to a higher risk of mood disorders. This was echoed in a systematic review and meta-analysis by Thomson et al. [48], and in a longitudinal study by Lorant et al. [50], where short-term fluid changes to one’s SEC was associated with greater depression symptoms—although the effects of SEC on mental health was more apparent between subjects instead of within. Conversely, another longitudinal study by Benzeval and Judge [51] in Britain found that a decline in income had only a minor effect on mental health. Similarly, Levesque et al. [52] reported a lack of evidence to support that changes in SEC has any unique effect on mental health separate from the effects of static or current SEC.

In this review, we aimed to investigate how different measurements of one’s SEC, be it static or fluid, are associated with emotional health symptoms (i.e., depression and anxiety), within the context of Covid-19.

### Current review

We conducted this systematic review with the aim of answering three pertinent research questions. First, in light of the socioeconomic disruptions brought about by the Covid-19 crisis, we sought to investigate how various classes of SEC indicators were associated with emotional health symptoms (i.e., anxiety and depression). Secondly, we aimed to compare SEC indicators to evaluate whether there were differences in how strongly specific indicators predicted mental health outcomes. Lastly, we assessed whether different groups of SEC indicators (static vs fluid, perceived vs actual) show dissociable effects on emotional health symptoms.

## Materials and method

This article constitutes a systematic review, which follows the Preferred Reporting Items for Systematic Reviews and Meta-Analyses (PRISMA) guidelines and statement [53]. This work has also been registered with PROSPERO (ID: CRD42021288508).

Article search was conducted over six popular databases (Scopus, ProQuest, PubMed, PsyInfo, OvidMedline, Web of Science), from November 4, 2021 after PROSPERO approved the registration, till November 11, 2021. Five keywords were used: (a) depression or anxiety; (b) Covid-19; (c) socioeconomic; (d) financial; and (e) economic. The combination of the keywords used in the searches was ((depression OR anxiety) AND covid* AND (socioeconomic* OR ses OR financ* OR economic*)). To ensure that only articles conducted on Covid-19 were retrieved, we restricted the publication date to be January 1, 2020 and beyond. The search strategy was developed by the lead author and was used consistently for each database. All subsequent reviews, extraction and consensus were jointly decided by the teams comprising eight reviewers and three assistants. Any disagreements between review authors were resolved through discussion. We used the Covidence, a web-based collaboration software platform that streamlines the production of systematic and other literature reviews, to assist us in the whole process of the systematic review. The characteristics of the qualitative data based on the PICO model are shown in Table 1.

**Table 1:** The PICO model

PICO	Description
Population	All populations and sub-samples
Intervention/Exposure	Covid-19 and its consequences, such as quarantine, lockdown etc
Comparison	The differential effects between various SEC indicators on emotional health
Outcomes	The effect of Covid-19 on the relationship between SEC and emotional health

### Eligibility criteria

As we are only 20 months into the pandemic at the time of this review, and in consideration of articles that may still be in the pipeline of publishing, we have decided to include, in addition to published articles, preprints as long as they fulfil our inclusion criteria, which includes: (a) depression and/or anxiety must be studied as the main outcome and measured using validated inventories or scales, such as the *Center for Epidemiologic Studies Depression Scale* (CES-D), *Patient Health Questionnaire* (PHQ), *State-Trait Anxiety Inventory* (STAI), etc.; (b) socioeconomic status and/or conditions must be studied as a predictor of depression and/or anxiety; articles using SEC as demographic information were excluded; (c) only quantitative studies were included; personal narrative, qualitative, case studies, meta-analyses or review articles were excluded. However, mixed-method studies were included; (d) only articles published on or after January 1, 2020 were included to ensure studies are COVID-19 related; and (e) English version must have been available.

### Screening and data extraction

The review process, including screening and data extraction, was carried out on Covidence. For each stage of the screening (title/abstract and full-text reviews), all studies were carefully reviewed by a team comprised of eight members, working independently. Two votes were required for each article to be decided as included or excluded. In case of any conflict, the lead author (JKC) was tasked to resolve it. Data extraction for each article was similarly carried out by any two members of the team independently. A consensus was reached between the two reviewers should there be any conflict.

### Quality appraisal

The methodological quality and risk of bias of studies eligible for review were assessed using the Joanna Briggs Institute (JBI) critical appraisal tools for cross-sectional [54] and cohort studies [55]. The tool assesses the trustworthiness, relevance, and results of published papers [54, 55]. The assessment was conducted and verified independently by any two reviewers. Any disagreements between reviewers regarding the qualification and analysis of articles were resolved through discussion.

### Data synthesis and coding

In consideration of the diverse ways SEC were measured and used in various studies, we have developed a coding scheme to assist us in data synthesis (Table 2).

**Table 2:** Legend table of the coding scheme for SEC

Variable	Description	Code
Education	Education level; years of education; parents’ education etc	1
Income	Personal income; household income	2
Occupation/Employment	Different categories of jobs; years of working; job loss etc	3
Socioeconomic Status (SES)	MacArthur^a^; census data; composite index etc	4
Living Condition	Household and neighbour condition; urban/rural etc	5
Foods/Basic Supplies	Food stocks; basic necessities adequacy and sufficiency etc	6
Financial Strain	Abilities to pay bills/rental/mortgage; sufficient to retire; financial state; financial wellbeing, etc	7
Economic Concerns	Fear of job loss; worry about not having enough money; job/financial security stress; concerns about income/work changes; concerns about the future economic scenario	8
Savings/Assets	Savings; pension; property; land; vehicles, etc	9

^a^MacArthur Scale of Subjective Social Status (MSS; Adler et al., 2000)

In addition, we further categorized the SEC indicators into ‘static/fluid’ and ‘actual/perceived’ categories depending on how they were measured. ‘Static’ refers to measurements that assessed SEC at a single time-point whereas ‘fluid’ measurements assessed the changes in SEC. ‘Actual’ and ‘perceived’ categorized the measurements in accordance to whether SEC was quantitatively or subjectively assessed. To clarify our point and intention, we used an example listed in Table 3 to illustrate.

**Table 3:** An example of how SEC was further categorized based on measurement methods

Variable	Static vs Fluid	Actual	Perceived
Monthly Income	Static	a) < $5000 b) $5000-$10,000 c) > $10,000	a) Low b) Medium c) High
	Fluid	a) Income reduced by 10% b) Income reduced by 50% c) Income reduced by 80% d) Total loss of income	a) Reduced a little bit b) Reduced quite a bit c) Reduced quite a lot

Thus, all SEC variables used in articles included in this systematic review were categorized into three levels. Firstly, according to the coding scheme listed in Table 1. Secondly, they were categorized as static or fluid according to the measurement method. Lastly, the SEC variables were further classified as actual or perceived, depending on how they were assessed. The intention for the three levels of categorization was to investigate which method or class of SEC measurements would yield the best result in predicting emotional health risks during the pandemic era.

With the classification, we would then assess how each class of SEC indicators was related to depression and anxiety. For each study, if the SEC class was associated with depression and/or anxiety in accordance with the theory of the social gradient in mental health, as per hypothesized (e.g., higher income/education was associated with lower depression/anxiety), we would count the finding as ‘Expected’. However, if the SEC class was showing the opposite result (e.g., higher income/education was associated with high depression/anxiety), we would count the finding as ‘Contrasting’. In the case that the SEC was not associated with depression and/or anxiety significantly, we would count it as ‘Non-significant’.

## Results

### Search results

**Fig. 1:**
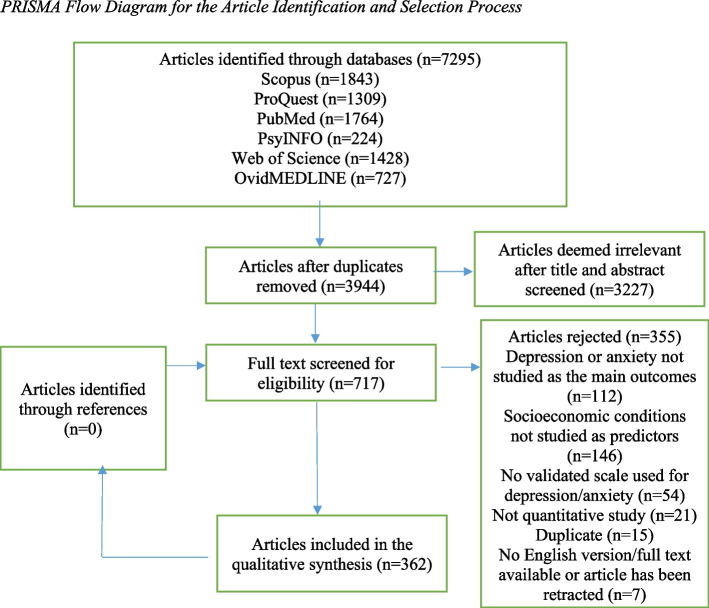
PRISMA flow diagram for the article identification and selection process

Initially, 7295 studies were imported to Covidence. After removing the duplicate studies (*n* = 3351), the abstracts of 3944 studies have been screened via Covidence in line with the defined including/exclusion criteria. Next, 717 studies have been found eligible for the full-text review, out of which, 355 studies were excluded. The main reasons for exclusion at the full-text review stage were as follows: (a) socioeconomic conditions have not been examined as primary predictors; (b) depression or anxiety were not studied as the main outcome variables; (c) mental health has not been assessed using a validated scale; (d) not being a quantitative study; (e) duplicate; and (f) the English version was not available, or the study has been retracted. Eventually, 362 studies were included in the data extraction stage. The details of the search and selection process are illustrated in the PRISMA diagram (Fig. 1).

The articles included in this review involved 324 cross-sectional and repeated cross-sectional studies with 38 longitudinal studies. The total number of participants sampled in these 362 articles was 5,677,007, including 2,342,848 (41.27%) female and 3,334,159 (58.73%) male. The countries where these studies were conducted was summarized in Appendix A [see Supplementary file]. The characteristics and main outcomes of these articles were tabulated as Supplementary Material 2 and Table 4.

**Table 4:** The follow-up outcomes of cohort studies included in the systematic review

Study ID	Quality Assessment	SEC Variable	Measurement Method	Actual / Perceived	Anxiety (Time 2) Inventory Outcomes		Depression (Time 2) Inventory Outcomes		Note
#3451	8	Education	Categorized as a) Primary; b) Secondary or higher	Actual	NA	NA	PHQ-9	Lower education level was associated with higher depression (OR: 1.89, *p* < .001)	
		Concern about work changes	1-item assessed using 10-point Likert scale (0 = No concern and 10 = Maximum concern) categorized into a) Mild (0–4); b) Moderate (5–7); and c) Maximum (8–10): "How much you worry about job changes during quarantine?"	Perceived		NA		Moderate and maximum concern were at higher risk for depression (OR: 1.28, *p* = .02; OR: 2.24, *p* < .001)	
		Running out of money	1-item assessed using 4-point Likert scale (1 = Not concerned and 4 = Very much concerned), grouped into a) No (1 and 2); and b) Yes (3 and 4): "Running out of money to pay expenses, rent and taxes	Perceived		NA		Higher concerns about running out of money was at a significant risk for depression (OR: 1.43, *p* < .001)	
		Employment situation	Categorized as a) Employed; and b) Unemployed	Actual		NA		Unemployed people were at a significantly higher risk of depression (OR = 2.99, *p* < .001)	
#195	11	Race	Black versus Non-black	Actual	PHQ-4	The odds of Black Americans reporting symptoms in a given month are about half that of non-Black Americans, and the differences are statistically significant in all months	PHQ-4	The odds of Black Americans reporting symptoms in a given month are about half that of non-Black Americans, and the differences are statistically significant in all months	
#150	11	Financial stress	Financial stress section of UCLA Life Stress Interview	Unsure	NA	NA	BDI-II for mothers, CES-D for adolescents	Financial stress positively associated with depressive symptoms for youth exhibiting blunted reward processing (β = 0.54, *p* = .007), and negatively associated with depressive symptoms for youth exhibiting increased reward processing (β = -0.89, *p* < .001. No significant effect of financial stress on maternal depression	
#3006	11	Education	Categorised as a) High school; b) High school graduate; c) Vocational/tech school/some college; and d) Bachelor's degree or more	Actual	NA	NA	PHQ-9	Having a high school qualification was associated with more depression than having a bachelor's degree (OR = 1.1, *p* = .024)	
		Household income	Categorised as a) < = 19,999; b) 20,000–44,999; c) 45,000–74,999; and d) > = 75,000	Actual		NA		Having $19,999 or less in household income was associated with 7 times the odds of depression relative to incomes of $75,000 or more (*p* < .001)	
		Household savings	Categorised as a) < = 4,999; and b) > 5,000	Actual		NA		Non-significant	
#203	9	Income	Categorized as a) Lower-income (annual household < $54,000); b) Middle income (> &54,000 and < $100,000); and c) Higher income (> $100,000)	Actual	GAD-7	No statistically significant longitudinal changes in anxiety symptoms by income	NA	NA	
#140	7	Unemployment	Unclear	Actual	PHQ-4	Unemployment remained as the strongest predictor of anxiety for 2020 cohort	PHQ-4	Unemployment became the second strongest predictor of depression for 2020 cohort	
		Household income	Unclear	Actual		Household income remained as the second strongest predictor of anxiety for 2020 cohort		Household income became the strongest predictors of depression for 2020 cohort	
#396	8	Education	Categorized as a) Tertiary or above; b) Secondary; and c) Primary or below	Actual	GAD-7	T2: Non-significant	PHQ-9	T2: Lower education was associated with higher depression (aOR = 1.47, *p* = .045; aOR = 1.99, *p* = .023).	
		Employment	Categorized as a) Employed; b) Dependent; and c) Unemployed	Actual		T2: Unemployed was associated with higher anxiety (aOR = 2.15, *p* = .009)		T2: Unemployed was associated with higher depression (aOR = 1.97, *p* = .018)	
		Monthly household income	Categorized as a) $80,000 or above; b) $60,000-$79,999; c) $40,000-$59,999; d) $20,000-$39,999; and e) $19,999 or below	Actual		T2: Non-significant		T2: Non-significant	
		Income change	Categorized as a) Stable/Increase; and b) Decrease	Perceived		T2: Non-significant		T2: Non-significant	
		Savings	Categorized as a) $3,000,000 or above; b) $2,000,000-$2,999,999; c) $1,000,000-$1,999,999; d) $500,000-$999,999; e) $200,000-$499,999; f) Less than $200,000; and g) None	Actual		T2: No saving was associated with higher anxiety (aOR = 2.61, *p* = .028)		T2: No saving was associated with higher depression (aOR = 2.65, *p* = .022)	
#243	8	COVID-19 related economic stress	Categorized as a) No impact; b) Low impact; and (c) High impact	Perceived	GAD-7	Non-significant		Non-significant	
#214	8	Income loss	US: Yes / No Israel: 5-point Likert scale ((from no income loss to extreme income loss)	Actual	GAD-7 (US) PROMIS (Israel)	Income loss was associated with higher increase in anxiety for US (β = .26, *p* < .001). But non-significant for Israel	PHQ-2 (US) PROMIS (Israel)	Income loss was associated with higher increase in depression for US (β = .26, *p* < .001). But non-significant for Israel	
		Covid-19 Financial worries	Both US and Israel used 5-point Likert scale (from not at all to a great deal)	Perceived		NA		Higher financial worries were associated with higher increase in depression for US (β = .07, *p* = .035). But non-significant for Israel	
		Pre-COVID-19 income	US: Annual income in bracket Israel: 5-point Likert scale (1 = Considerably below average and 5 = Considerably above average)			Higher income was associated with lower anxiety for US (β = -.02, *p* < .001). But non-significant for Israel		Higher income was associated with lower depression for US (β = -.02, *p* < .001). But non-significant for Israel	
#130	11	Pre-crisis socioeconomic status: Relative financial status	Categorized as a) Below average; b) Average; and c) Above average	Perceived	NA	NA	MDI	Participants' report of a below average relative financial status was a significant predictor of depression (B = 0.12, *p* < 0.05). However, participants' report of an above average relative financial status was also a significant predictor of depression (B = 0.10, *p* < 0.05)	
		Pre-crisis socioeconomic status: Difficulty paying usual bills	Categorized as a) Easy or very easy; b) Fairly easy; and c) Rather difficult or difficult	Perceived		NA		Non-significant	
		Pre-crisis socioeconomic status: highest level of education	Categorized as a) Compulsory schooling (ISCED 2; 9 years); b) Secondary school diploma (ISCED 34; 12–13 years); c) Apprenticeship (ISCED 35; 12–13 years); d) Bachelor's degree (ISCED 6; 15 years); and e) Master's degree (ISCED 7; 17 years)	Actual		NA		Secondary school was a significant predictor of higher depression (B = 0.18, *p* < 0.05). However, Master's degree was also a significant predictor of higher depression (B = 0.21, *p* < 0.05)	
		Changes in work situation due to COVID-19	Categorized as a) No change; b) Job loss; c) Partial unemployment; and d) Losing money as self-employed	Actual		NA		Participants' report of self-employed & lost money was a significant predictor of depression (B = 0.36, *p* < 0.05). However, lost job and partial unemployment were not a significant predictor of depression	
#101	9	Job loss	Binary response (Yes/No)	Actual	GAD-7	Non-significant	PHQ-9	Non-significant	
		Income loss	Binary response (Yes/No)	Actual		Non-significant		Non-significant	
		Worries about losing job / employment	Binary response (Yes/No)	Perceived		Worries about losing job/employment showed associations with higher levels of anxiety		Worries about losing job/employment showed associations with higher levels of depression	
		Worries about finances	Binary response (Yes/No)	Perceived		Worries about finances showed associations with higher levels of anxiety		Worries about finances showed associations with higher levels of depression	
#24	9	Education	Categorized as a) No formal education; b) Fundamental education; c) Secondary education; d) University degree; and e) Master or above	Actual	GAD-7	Non-significant	CES-D	There were significant associations between fundamental education and lower follow-up depression (β = -3.81, *p* < .05), secondary education and lower follow-up depression (β = -3.95, *p* < .01), university degree and lower follow-up depression (β = -3.91, *p* < .01), and university degree of master and above and lower follow-up depression (β = -3.58, *p* < .01)	
		Employment status	Categorized as a) Full-time employed; b) In retirement or early retirement; c) In vocational training/retraining/education; d) Looking after home or family; e) Other status; f) Parental leave; g) Part-time employed; h) Permanently sick or disabled; i) Self-employed or working for own family business; and j) Unemployed	Actual		Non-significant		Non-significant	
		Income	Categorized as a) 0–25000 Euros; b) 25,000–75000; c) 75,000–150000; d) > 150,000 Euros; and e) No answer	Actual		Non-significant		Non-significant	
#380	11	Employment status	Categorized as a) Working in Wave 1; b) Paid leave in Wave 1; c) Furloughed in Wave 1; d) Not employed in Wave 1; e) Paid leave in Wave 2; f) Furloughed in Wave 2; and g) Not employed in Wave 2	Actual	NA	NA	PHQ-2, CESD-10	Being employed during Wave 2 was associated with decreased depression (OR = -0.24, *p* < .05)	
		Tertiary Education	Binary response (Yes/No)	Actual		NA		Non-significant	
#372	11	Education	Years of education	Actual	PROMIS Anxiety	Non-significant	PHQ-8	Non-significant	
		Employment status	Categorized as a) Part-time; and b) Full-time	Actual		Non-significant		Non-significant	
		Financial well-being	Measured using the CFPB Financial Well-Being Scale	Perceived		Lower financial well-being was associated with higher anxiety (aB = -0.24, *p* < .05)		Lower financial well-being was associated with higher depression (aB = -0.20, *p* < .05)	
#354	11	Economic adversity	Categorized as a) Faced with new health expenses; b) Experienced adversity but did not reduce food consumption; and c) Reduced food consumption as response to adversity	Actual	GAD-7	People who experienced adversity but did not reduce food consumption had higher odds of anxiety (Ethiopia, OR = 2.36, *p* < .05; Peru, OR = 2.50, *p* < .05; Vietnam, OR = 1.62, *p* < .05). People who reduced food consumption as response to adversity had higher odds of anxiety (Ethiopia, OR = 7.19, *p* < .05; Peru, OR = 2.40, *p* < .05; Vietnam, OR = 1.67, *p* < .05)	PHQ-8	People who experienced adversity but did not reduce food consumption had higher odds of depression (Ethiopia, OR = 3.81, *p* < .05; Peru, OR = 2.05, *p* < .05; Vietnam, OR = 1.70, *p* < .05). People who reduced food consumption as response to adversity had higher odds of depression (Ethiopia, OR = 10.89, *p* < .05; Peru, OR = 2.53, *p* < .05; Vietnam, OR = 1.91, *p* < .05)	
		Employment status	Categorized as a) Did not work before pandemic but working now; b) Worked before pandemic and still working; and c) Worked before pandemic but no longer working	Actual		People who worked before pandemic but no longer working had higher odds of anxiety (Ethiopia, OR = 2.29, *p* < .05; India, OR = 2.50, *p* < .05)		People who worked before pandemic but no longer working had higher odds of depression (Ethiopia, OR = 1.68, *p* < .05; Vietnam, OR = 1.51, *p* < .05)	
#334	11	Annual household income	Categorized as a) < $79,999; and b) $80,000 or more	Actual	STAI-SF	Non-significant	CESD-10	Non-significant	
		COVID-19 impact on income or employment	Binary response (Yes/No)	Perceived		Impact of COVID-19 on income or employment had a 1.42 mean increase in anxiety score (*p* < .001)		Impact of COVID-19 on income or employment had a 3.00 mean increase in depression score (*p* < .001)	
#446	8	Education Personal income Family income Householder Unemployment experience	Categorized as a) Jr high school; b) High school; c) Associate degree or Diploma; d) Bachelor; and e) Master or Doctorate Categorized as a) Low; b) Middle; and c) High Categorized as a) Low; b) Middle; and c) High Binary response (Yes/No) Unclear	Actual Perceived Perceived Actual Actual	NA	NA NA NA NA NA	CES-D	Non-significant Non-significant Non-significant Non-significant Unemployment was a factor associated with increasing depression (*p* = 0.003)	
#1415	11	Education	Categorized as a) Less than high school; b) Some high school, no diploma; c) High school graduate or equivalent; d) Some college, no degree; e) Associate degree; f) Bachelor’s degree; g) Master’s degree; h) Professional school degree; and i) Doctorate degree	Actual	NA	NA	PHQ-8	Individuals with higher education reported a larger increase in depressive symptoms (total effect = .032)	
		Income (household)	Total combined income of all family members 15 years or older who lived in the household over the past year on a 17-point scale (1 = less than $5000 and 17 = $200,000 or above)	Actual		NA		Non-significant	
#1278	11	Financial concerns	11-point Likert scale (0–10) with higher values intended to reflect higher frequency or intensity to the question: “How concerned are you about your financial situation as a result of the pandemic?”	Perceived	GAD-7	Non-significant	PHQ-9	Non-significant	
#1140	11	Job loss	Binary response (Yes/No)	Actual	GAD-7	Non-significant	CESD-SF	Non-significant	
		Education loss	Binary response (Yes/No)	Actual		Non-significant		Non-significant	
		Not employed/ in education (pre-pandemic)	Binary response (Yes/No)	Actual		Non-significant		Non-significant	
		Low SES family	Binary response (Yes/No) – defined as ≤ 1 SD on SES scale aggregating annual gross income, parental education level, and parental occupation prestige from ages 15 -17 years	Actual		Non-significant		Non-significant	
#933	8	Education	Unclear	Actual	GAD-7	Lower educational background was associated with greater anxiety in ALSPAC-parents and ALSPAC-young cohorts, but not in the Generation Scotland cohort	SMFQ	Lower education background was associated with greater depression in ALSPAC parents and Generation Scotland cohorts, but not in the ALSPAC-young cohort	
		Income	Unclear	Actual		Higher income before the pandemic was associated with lower anxiety in ALSPAC-young and Generation Scotland cohort, but not in the ALSPAC-parent cohort		Higher income before the pandemic was associated with lower depression in ALSPAC-parents and Generation Scotland cohorts, but not in the ALSPAC-young cohort	
		Recent financial problems	Unclear	Unclear		NA		NA	
#883	11	Income loss	Categorized as a) No income loss; b) Greater than 0% but less than 50% monthly income loss; and c) Greater than or equal to 50% monthly income loss	Actual	GAD-7	Non-significant	PHQ-9	Those reporting ≥ 50% income loss showed a significant increase in depression scores	
#2773	11	Education	Binary variable (Yes / No) indicating whether or not at least some secondary education was received	Actual	NA	NA	PHQ-8	Non-significant	
		Employment status	Binary indicator (Yes / No) of whether the respondent was engaged in activity resulting in monetary, food or other forms of compensation	Actual		NA	PHQ-8	Non-significant	
		Monthly income	Binary variable (Yes / No) indicating whether or not at least 100,000 Ush is earned per month	Actual		NA	PHQ-8	Non-significant	
		Food security	5 items adapted from the Food Insecurity Experience Scale	Perceived		NA	PHQ-8	High food insecurity was associated with elevated depressive symptoms (aOR = 4.56, p = < .05)	
#7203	11	Income	Categorized as (in £) a) < 15,000; b) 15,000 to < 30,000; c) 30,000 to 45,000; and d) ≥ 45,000	Actual	K6	Those with an annual income of < £15,000 were more likely to develop psychological distress at T2 compared to those with an annual income of ≥ £45,000 (OR = 2.09)	K6	Those with an annual income of < £15,000 were more likely to develop psychological distress at T2 compared to those with an annual income of ≥ £45,000 (OR = 2.09)	
#6670	11	Perceived financial risk due to Covid-19	A single-item that asked participants to report the percent chance they will run out of money because of the coronavirus in the next three months	Perceived	PHQ-4	Perceived financial risks (i.e., running out of money) increased and explained 14–15% of the initial rise in psychological distress between 10–18 March and 1–14 April (β = 0.038)	PHQ-4	Perceived financial risks (i.e., running out of money) increased and explained 14–15% of the initial rise in distress between 10–18 March and 1–14 April (β = 0.038)	
#3282	11	Economic impact payments	Binary response (Yes/No)	Actual	GAD-2	Non-significant	PHQ-2	EIP recipients were significantly associated with increased depression (marginal effect = 0.26)	
#3242	8	Socioeconomic position (SEP)	Categorized into a) Low; b) Medium; and c) High based on a number of measures (e.g., parents' education, number of books at home, number of cars etc.)	Perceived	NA	NA	HSCL (6-items)	No significant changes in SEP inequalities in either boys or girls between T1 and T2. In actual fact, the differences in the share of medium and high SEP girls reporting high depressive symptoms narrowed between T1 and T2, as high SEP girls reported more problems	
#78	7	Education	Highest years of education in the household	Actual	SC-90-R	An extra year of education reduced the toll on anxiety by 0.5 percentage points	SC-90-R	An extra year of education reduced the toll on depression by 0·6 percentage points	
		Income (per capita)	Monthly household income per capita (2017 USD)	Actual		Non-significant		Non-significant	
		Asset index	Unclear	Unclear		Non-significant		Non-significant	
		Beneficiary of conditional cash transfer	Unclear	Unclear		Non-significant		Non-significant	
		Job or Income loss	Unclear	Unclear		Anxiety increased by 6 percentage points for those who reported job or income loss		Non-significant	
#89	11	Income (annual pre-Covid)	Categorized as a) < 10 k;b) 10-20 k; c) 20-30 k; d) 30-40 k; e) 40-50 k; f) 50-60 k; g) 60-70 k; h) 70-80 k; i) 80-90 k; j) 90-100 k; k) 100-150 k; and l) > 150 k	Actual	SAI	Subjects with lower income were significantly related to having higher anxiety (β = -.32, *p* < 0.001)	SDS	Subjects with lower income were significantly related to higher depression (β = -.37, *p* < 0.001)	
#56	11	Employment Status	Categorized as a) Employed; b) Out of work; c) Homemaker; d) Student; and e) Retired	Actual	GAD-7	Non-significant	NA	NA	
						Non-significant		NA	
		Income	Categorized as a) < $50,000; b) $50,000 to $ 90,000; and c) > = $100,000	Actual					
		Income loss	Unclear	Unclear		Having lost income due to COVID-19 was positively associated with moderate or severe anxiety symptoms (aPR = 1.27)		NA	
#5	8	Education	Categorized as a) High school/GED or lower; b) Some college; and c) Bachelor or higher"	Actual	GAD-2	Non-significant	PHQ-2	Non-significant	
		Income (household)	Categorized as a) Less than $49,000; b) $50,000-$99,000; c) $100,000-$149,000; d) $150,000 or higher; and e) Unknown"	Actual		Compared to people with less than $49,000 household income, $50,000 to $99,000 household income have lower risk for psychological distress (aPR = 0.86, *p* < .05); $100,000 to $149,000 household income have lower risk for psychological distress (aPR = 0.80, *p* < .01); and $150,000 and above household income have lower risk for psychological distress (aPR = 0.70, *p* < .001)		Compared to people with less than $49,000 household income, $50,000 to $99,000 household income have lower risk for psychological distress (aPR = 0.86, *p* < .05); $100,000 to $149,000 household income have lower risk for psychological distress (aPR = 0.80, *p* < .01); $150,000 and above household income have lower risk for psychological distress (aPR = 0.70, *p* < .001)."	
#2874	8	Annual household income	Categorized as a) > 200 thousand Yuan; b) 30–100 thousand Yuan; and c) 100–200 thousand Yuan	Actual	NA	NA	PHQ-9	Compared to annual household income of more than 200 thousand Yuan, annual household income of 30 to 100 thousand Yuan (OR = 1.49, *p* < .05) and 100 to 200 thousand Yuan (OR = 1.21, *p* < .05) have higher risk for depression	
		Education	Categorized as a) Bachelor degree or below level; and b) Master degree or higher level	Actual		NA		Non-significant	

### Quality appraisal findings

Among 324 cross-sectional/repeated cross-sectional studies included in the review, 242 articles (74.69%) obtained the maximum score of 8 on JBI criteria for the cross-sectional study, 67 articles (20.68%) got a score of 7, and 15 articles (4.63%) scored 6 and below. Of the 38 longitudinal studies, 24 articles (63.16%) received the maximum of 11 points on JBI criteria for the cohort study, 5 (13.16%) got 10 points, 3 (7.89%) obtained a score of 9, 6 studies got a score of 8 and below (15.79%). Details of the quality appraisal were tabulated as Tables 5 and 6.

**Table 5:** Quality assessments for cross-sectional studies using JBI critical appraisal checklist for cross-sectional studies

ID, First Author (Year)	Were the criteria for inclusion in the sample clearly defined?	Were the study subjects and the setting described in detail?	Was the exposure measured in a valid and reliable way?	Were objective, standard criteria used for measurement of the condition?	Were confounding factors identified?	Were strategies to deal with confounding factors stated?	Were the outcomes measured in a valid and reliable way?	Was appropriate statistical analysis used?
#6778	Yes	Yes	Yes	Yes	Yes	Yes	Yes	Yes
#6255	Yes	Yes	Yes	Yes	Yes	Yes	Yes	Yes
#6116	Yes	Yes	Yes	Yes	Yes	Yes	Yes	Yes
#5913	Yes	Yes	Yes	Yes	Yes	Yes	Yes	Yes
#5493	Yes	Yes	Yes	Yes	No	No	Yes	Yes
#5485	Yes	Yes	Yes	Yes	Yes	Yes	Yes	Yes
#5401	No	Yes	Yes	Yes	Yes	Yes	Yes	Yes
#5398	Yes	Yes	Yes	Yes	No	No	Yes	Yes
#5227	Unclear	Yes	Yes	Yes	Yes	Yes	Yes	Yes
#5189	Yes	Yes	Yes	Yes	Yes	Yes	Yes	Yes
#5104	Yes	Yes	Yes	Yes	Yes	Yes	Yes	Yes
#675	No	Yes	Yes	Yes	Yes	Yes	Yes	Yes
#677	Unclear	Yes	Yes	Yes	Yes	Yes	Yes	Yes
#4928	Yes	Yes	Yes	Yes	Yes	Yes	Yes	Yes
#4908	Yes	Yes	Yes	Yes	Yes	Yes	Yes	Yes
#4660	Yes	Yes	Yes	Yes	Yes	Yes	Yes	Yes
#4501	Yes	Yes	Yes	Yes	Unclear	Unclear	Yes	Yes
#3940	Yes	Yes	Yes	Yes	No	No	Yes	Yes
#1166	Yes	Yes	Yes	Yes	Yes	Yes	Yes	Yes
#3883	Yes	Yes	Yes	Yes	Yes	Yes	Yes	Yes
#3555	Yes	Yes	Yes	Yes	Yes	Yes	Yes	Yes
#3536	No	Yes	Yes	Yes	Yes	Yes	Yes	Yes
#3531	Yes	Yes	Yes	Yes	Yes	Yes	Yes	Yes
#3451	Yes	Yes	Yes	Yes	Yes	Yes	Yes	Yes
#3366	Yes	Yes	Yes	Yes	Yes	Yes	Yes	Yes
#3309	Yes	Yes	Yes	Yes	Yes	Yes	Yes	Yes
#3284	Yes	Yes	Yes	Unclear	Yes	Yes	Yes	Yes
#3272	Yes	Yes	Yes	Yes	Yes	Yes	Yes	Yes
#3266	Yes	Yes	Yes	Yes	Yes	Yes	Yes	Yes
#3238	Yes	No	Yes	Yes	Yes	Yes	Yes	Yes
#3171	Yes	Yes	Yes	Yes	Yes	Yes	Yes	Yes
#3059	Yes	Yes	Yes	Yes	Yes	Yes	Yes	Yes
#3051	Yes	Yes	Yes	Yes	Yes	Yes	Yes	Yes
#3039	Yes	Yes	Yes	Yes	Yes	Yes	Yes	Yes
#3035	Yes	Yes	Yes	Yes	Yes	Yes	Yes	Yes
#2944	Yes	Yes	Yes	Yes	Yes	Yes	Yes	Yes
#2837	Yes	Yes	Yes	Unclear	Yes	Yes	Yes	Yes
#2821	Yes	Yes	Yes	Yes	Yes	Yes	Yes	Yes
#2800	Yes	Yes	Yes	Yes	Yes	Yes	Yes	Yes
#2795	Yes	Yes	Yes	Yes	Yes	Yes	Yes	Yes
#684	Yes	Yes	Yes	Yes	Yes	Yes	Yes	Yes
#2792	Yes	Yes	Yes	Yes	Yes	Yes	Yes	Yes
#2783	Yes	Yes	Yes	Yes	Yes	Yes	Yes	Yes
#2780	Yes	Yes	Yes	Yes	Yes	Yes	Yes	Yes
#2625	Yes	Yes	Yes	Yes	Yes	Yes	Yes	Yes
#2593	Yes	Yes	Yes	Yes	Yes	Yes	Yes	Yes
#2590	Yes	Yes	Yes	Yes	Yes	Yes	Yes	Yes
#2468	Yes	Yes	Yes	Yes	Yes	Yes	Yes	Yes
#2325	Yes	Yes	Yes	Yes	Yes	Yes	Yes	Yes
#2324	Yes	Yes	Yes	Yes	Yes	Yes	Yes	Yes
#2289	Yes	Yes	Yes	Yes	Yes	Yes	Yes	Yes
#2088	Yes	Yes	Unclear	Yes	Yes	Yes	Yes	Yes
#2045	Yes	Yes	Yes	Yes	Yes	Yes	Yes	Yes
#1676	Yes	Yes	Yes	Yes	Yes	Yes	Yes	Yes
#1626	Yes	Yes	Yes	Yes	Yes	Yes	Yes	Yes
#1462	Yes	Yes	Unclear	Yes	Yes	Yes	Yes	Yes
#1473	Unclear	Yes	Yes	Yes	Yes	Yes	Yes	Yes
#1447	Yes	Unclear	Yes	Yes	Yes	Yes	Yes	Yes
#2627	Yes	Yes	No	Yes	Yes	Yes	Yes	Yes
#1454	Yes	Yes	Yes	Yes	Yes	Yes	Yes	Yes
#2742	Yes	Yes	Yes	Yes	Yes	No	Yes	Yes
#1389	Yes	Yes	Yes	Yes	Yes	Yes	Yes	Yes
#812	Yes	Yes	Yes	Yes	Yes	Yes	Yes	Yes
#1387	Yes	Yes	Yes	Yes	Yes	Yes	Yes	Yes
#1359	Yes	Yes	Yes	Yes	Yes	Yes	Yes	Yes
#1345	Yes	Yes	Yes	Yes	Yes	Yes	Yes	Yes
#1339	Yes	Yes	Yes	Yes	Yes	Yes	Yes	Yes
#1284	Yes	Yes	Yes	Yes	Yes	Yes	Yes	Yes
#1251	Yes	Yes	Yes	Yes	Yes	Yes	Yes	Yes
#1226	Yes	Yes	Yes	Yes	Yes	Yes	Yes	Yes
#1080	Yes	Yes	Yes	Yes	Yes	Yes	Yes	Yes
#1077	Yes	Yes	Yes	Yes	Yes	Yes	Yes	Yes
#1023	Yes	Yes	Yes	Yes	Yes	Yes	Yes	Yes
#993	Yes	Yes	Yes	Yes	Yes	Yes	Yes	Yes
#989	Yes	Yes	Yes	Yes	Yes	Yes	Yes	Yes
#986	Yes	Yes	Yes	Yes	Yes	Yes	Yes	Yes
#909	Yes	Yes	Yes	Yes	Yes	Yes	Yes	Yes
#1059	Yes	Yes	Yes	Yes	Yes	Yes	Yes	Yes
#1074	Yes	Yes	Yes	Yes	Yes	Yes	Yes	Yes
#1065	No	Yes	Yes	Yes	Yes	Yes	Yes	Yes
#807	Yes	Yes	Yes	Yes	Yes	Yes	Yes	Yes
#1020	Unclear	Yes	Yes	Yes	Yes	Yes	Yes	Yes
#725	Yes	Yes	Yes	Yes	Yes	Yes	Yes	Yes
#831	Yes	Yes	Yes	Yes	Yes	Yes	Yes	Yes
#688	Yes	Yes	Yes	Yes	Yes	Yes	Yes	Yes
#676	Yes	Yes	Yes	Yes	Yes	Yes	Yes	Yes
#661	Yes	Yes	Yes	Yes	Yes	Yes	Yes	Yes
#633	Yes	Yes	Yes	Yes	Yes	Yes	Yes	Yes
#613	Yes	Yes	Yes	Yes	Yes	Yes	Yes	Yes
#601	Yes	Yes	Yes	Yes	Yes	Yes	Yes	Yes
#593	Yes	Yes	Yes	Yes	Yes	Yes	Yes	Yes
#581	Yes	Yes	Yes	Yes	Yes	Yes	Yes	Yes
#548	Yes	Yes	Yes	Yes	Yes	Yes	Yes	Yes
#536	Yes	Yes	Yes	Yes	Yes	Yes	Yes	Yes
#534	Yes	Yes	Yes	Yes	Yes	Yes	Yes	Yes
#512	Yes	Yes	Yes	Yes	Yes	Yes	Yes	Yes
#507	Unclear	Yes	Yes	Yes	Yes	Yes	Yes	Yes
#503	Yes	Yes	Yes	Yes	Yes	Yes	Yes	Yes
#501	Yes	Yes	Yes	Yes	Yes	Yes	Yes	Yes
#499	Yes	Yes	Yes	Yes	Yes	Yes	Yes	Yes
#497	Yes	Yes	Yes	Yes	Yes	Yes	Yes	Yes
#477	Yes	Yes	Unclear	Yes	Yes	Yes	Yes	Yes
#462	Yes	Yes	Yes	Yes	Yes	Yes	Yes	Yes
#407	Yes	Yes	Yes	Yes	Yes	Yes	Yes	Yes
#396	Yes	Yes	Yes	Yes	Yes	Yes	Yes	Yes
#381	Yes	Yes	Yes	Yes	Yes	Yes	Yes	Yes
#365	Yes	Yes	Yes	Yes	Yes	Yes	Yes	Yes
#349	Yes	Yes	Yes	Yes	Yes	Yes	Yes	Yes
#348	Unclear	Yes	Yes	Yes	Yes	Yes	Yes	Yes
#296	Yes	Yes	Yes	Yes	Yes	Yes	Yes	Yes
#256	Yes	Yes	Unclear	Yes	Yes	Yes	Yes	Yes
#224	Yes	Yes	Yes	Yes	Yes	Yes	Yes	Yes
#204	Yes	Yes	Yes	Yes	Yes	Yes	Yes	Yes
#180	Yes	Yes	Yes	Yes	Yes	Yes	Yes	Yes
#177	Yes	Yes	Yes	Yes	Yes	Yes	Yes	Yes
#172	Yes	Yes	Yes	Yes	Yes	Unclear	Yes	Yes
#171	Yes	Yes	Yes	Yes	Yes	Yes	Yes	Yes
#140	Yes	Yes	Unclear	Yes	Yes	Yes	Yes	Yes
#139	Yes	Yes	Yes	Yes	Yes	Yes	Yes	Yes
#137	Yes	Yes	Unclear	Yes	Yes	Yes	Yes	Yes
#133	Yes	Yes	Yes	Yes	Yes	Yes	Yes	Yes
#126	Yes	Yes	Yes	Yes	Yes	Yes	Yes	Yes
#87	Yes	Yes	Yes	Yes	Yes	Yes	Yes	Yes
#85	Yes	Yes	Yes	Yes	Yes	Yes	Yes	Yes
#65	Yes	Yes	Yes	Yes	Yes	Yes	Yes	Yes
#50	Yes	Yes	Yes	Yes	No	No	Yes	Yes
#28	Yes	Yes	Yes	Yes	Yes	Yes	Yes	Yes
#14	Yes	Yes	Yes	Yes	Yes	Yes	Yes	Yes
#7	Yes	Yes	Yes	Yes	Yes	Yes	Yes	Yes
#2312	Yes	Yes	Yes	Yes	Yes	Yes	Yes	Yes
#3009	Yes	Yes	Yes	Yes	No	No	Yes	Yes
#572	Yes	Yes	Yes	Yes	Yes	Yes	Yes	Yes
#3004	Yes	Yes	Yes	Yes	Yes	Yes	Yes	Yes
#492	Yes	Yes	Yes	Yes	No	No	Yes	Yes
#439	Yes	Yes	Unclear	Yes	Yes	Yes	Yes	Yes
#2992	Yes	Yes	Yes	Yes	Yes	Yes	Yes	Yes
#2879	Yes	Unclear	Yes	Yes	Yes	Yes	Yes	Yes
#2563	Yes	Yes	Yes	Yes	No	Yes	Yes	Yes
#1205	Unclear	Yes	Yes	Yes	Yes	Yes	Yes	Yes
#2578	Yes	Yes	Yes	Yes	Yes	Yes	Yes	Yes
#2910	Yes	Yes	Yes	Yes	Yes	Yes	Yes	Yes
#76	Yes	Yes	Yes	Yes	Yes	Yes	Yes	Yes
#1189	Yes	Yes	No	Yes	Yes	Yes	Yes	Yes
#347	Yes	Yes	Yes	Yes	Yes	Yes	Yes	Yes
#97	Yes	Yes	Yes	Yes	Yes	Yes	Yes	Yes
#940	Yes	Yes	Yes	Yes	Yes	Yes	Yes	Yes
#636	No	Yes	Yes	Yes	Yes	Yes	Yes	Yes
#2123	Yes	Yes	Yes	Yes	No	No	Yes	Unclear
#637	Yes	Yes	Yes	Yes	Yes	Yes	Yes	Yes
#1239	Yes	Yes	Yes	Yes	Yes	Yes	Yes	Yes
#1293	Unclear	Yes	Yes	Yes	Yes	Yes	Yes	Yes
#622	Yes	Yes	Yes	Yes	Yes	Yes	Yes	Yes
#3212	Yes	Yes	Yes	Yes	Yes	Yes	Yes	Yes
#723	Yes	Yes	Yes	Yes	Yes	Yes	Yes	Yes
#1213	Yes	Yes	Yes	Yes	Yes	Yes	Yes	Yes
#3061	Yes	Yes	Unclear	Yes	Yes	Yes	Yes	Yes
#3242	Yes	Yes	Yes	Yes	Yes	Yes	Yes	Yes
#390	Yes	Yes	Yes	Yes	Yes	Yes	Yes	Yes
#630	Yes	Yes	Yes	Yes	Yes	Yes	Yes	Yes
#4762	Yes	Yes	Yes	Yes	Yes	Yes	Yes	Yes
#2815	Yes	Yes	Yes	Yes	Yes	Yes	Yes	Yes
#629	Yes	Yes	Yes	Yes	Yes	Yes	Yes	Yes
#57	Yes	Yes	Unclear	Yes	Yes	Yes	Yes	Yes
#2936	Yes	Unclear	Yes	Yes	Yes	Yes	Yes	Yes
#93	Yes	Yes	Unclear	Yes	Yes	Yes	Yes	Yes
#417	Yes	Yes	Yes	Yes	Yes	Yes	Yes	Yes
#376	Unclear	Yes	Yes	Yes	Yes	Yes	Yes	Yes
#340	Unclear	Yes	Yes	Yes	Yes	Yes	Yes	Yes
#246	Yes	Yes	Yes	Yes	Yes	Yes	Yes	Yes
#556	Yes	Yes	Yes	Yes	Yes	Yes	Yes	Yes
#459	Unclear	Yes	Yes	Yes	Yes	Yes	Yes	Yes
#759	Yes	Yes	Yes	Yes	Yes	Yes	Yes	Yes
#2355	Yes	Yes	Yes	Yes	Yes	Yes	Yes	Yes
#2819	Yes	Yes	Yes	Yes	Yes	Yes	Yes	Yes
#5080	Yes	Yes	Yes	Yes	Yes	Yes	Yes	Yes
#4670	Yes	Yes	Yes	Yes	Yes	Yes	Yes	Yes
#3267	Unclear	Yes	Yes	Yes	Yes	Yes	Yes	Yes
#3097	Yes	Yes	Yes	Yes	Yes	Yes	Yes	Yes
#3046	Unclear	Yes	Unclear	Yes	Yes	Yes	Yes	Yes
#3023	Yes	Yes	Yes	Yes	Yes	Yes	Yes	Yes
#3198	Yes	Yes	Yes	Yes	Yes	Yes	Yes	Yes
#80	Yes	Yes	Unclear	Yes	Yes	Yes	Yes	Yes
#2809	Yes	Yes	Unclear	Yes	Yes	Yes	Yes	Yes
#6641	Yes	Yes	Yes	Yes	Yes	Yes	Yes	Yes
#2055	Yes	Yes	Unclear	Yes	Yes	Yes	Yes	Yes
#702	Yes	Yes	Yes	Yes	Yes	Yes	Yes	Yes
#532	Yes	Yes	Yes	Yes	Yes	Yes	Yes	Yes
#3187	Yes	Yes	Yes	Yes	Yes	Yes	Yes	Yes
#425	Yes	Yes	Yes	Yes	Yes	Yes	Yes	Yes
#529	Yes	Yes	Yes	Yes	Yes	Yes	Yes	Yes
#3053	Yes	Yes	Yes	Yes	Yes	Yes	Yes	Yes
#185	Yes	Yes	Yes	Yes	Yes	Yes	Yes	Yes
#2846	Yes	Yes	Yes	Yes	Yes	Yes	Yes	Yes
#2920	Yes	Yes	Yes	Yes	Yes	Yes	Yes	Yes
#2890	Yes	Yes	Yes	Yes	Yes	Yes	Yes	Yes
#445	Yes	Yes	Yes	Yes	Yes	Yes	Yes	Yes
#546	Yes	Yes	Yes	Yes	Yes	Yes	Yes	Yes
#3104	Yes	Yes	Yes	Yes	Yes	Yes	Yes	Yes
#585	Yes	Yes	Unclear	Yes	Yes	Yes	Yes	Yes
#247	Yes	Yes	Yes	Yes	Yes	Yes	Yes	Yes
#192	Yes	Yes	Yes	Yes	Yes	Yes	Yes	Yes
#389	Yes	Yes	Yes	Yes	Yes	Yes	Yes	Yes
#2358	Yes	Yes	Unclear	Yes	Yes	Yes	Yes	Yes
#440	Yes	Yes	Yes	Yes	Yes	Yes	Yes	Yes
#1353	Yes	Yes	Yes	Yes	Yes	Yes	Yes	Yes
#2583	Yes	Yes	Yes	Yes	Yes	Yes	Yes	Yes
#744	Yes	Yes	Yes	Yes	Yes	Yes	Yes	Yes
#1321	Yes	Yes	Yes	Yes	Yes	Yes	Yes	Yes
#3685	Yes	Yes	Yes	Yes	Yes	Yes	Yes	Yes
#580	Yes	Yes	Unclear	Yes	Yes	Yes	Yes	Yes
#656	Yes	Yes	Yes	Yes	Yes	Yes	Yes	Yes
#2919	Yes	Yes	Yes	Yes	Yes	Yes	Yes	Yes
#443	Yes	Yes	Yes	Yes	Yes	Yes	Yes	Yes
#553	Yes	Yes	Yes	Yes	Yes	Yes	Yes	Yes
#685	Yes	Yes	Yes	Yes	Yes	Yes	Yes	Yes
#687	Yes	Yes	Yes	Yes	Yes	Yes	Yes	Yes
#5204	Yes	Yes	Yes	Yes	Yes	Yes	Yes	Yes
#2372	Yes	Yes	No	Yes	Yes	Yes	Yes	Yes
#2685	Yes	Yes	Yes	Yes	Yes	Yes	Yes	Yes
#660	Yes	Yes	Yes	Yes	Yes	Yes	Yes	Yes
#584	Yes	Yes	Yes	Yes	Yes	Yes	Yes	Yes
#2523	Yes	Yes	Yes	Yes	Yes	Yes	Yes	Yes
#2952	Yes	Yes	Yes	Yes	Yes	Yes	Yes	Yes
#1865	Yes	Yes	Yes	Yes	Yes	Yes	Yes	Yes
#1695	Yes	Yes	Yes	Yes	Yes	Yes	Yes	Yes
#1706	Yes	Yes	Yes	Yes	Yes	Yes	Yes	Yes
#3344	Yes	Yes	Yes	Yes	Yes	Yes	Yes	Yes
#4	Yes	Yes	Yes	Yes	Yes	Yes	Yes	Yes
#227	Yes	Yes	Yes	Yes	Yes	Yes	Yes	Yes
#217	Yes	Yes	Yes	Yes	Yes	Yes	Yes	Yes
#211	Yes	Yes	Yes	Yes	Yes	Yes	Yes	Yes
#208	Yes	Yes	Yes	Yes	Yes	Yes	Yes	Yes
#200	Yes	Yes	Yes	Yes	Yes	Yes	Yes	Yes
#164	Yes	Yes	Yes	Yes	Yes	Yes	Yes	Yes
#156	Yes	Yes	Yes	Yes	Yes	Yes	Yes	Yes
#2991	Yes	Yes	Yes	Yes	Yes	Yes	Yes	Yes
#2999	Yes	Yes	No	Yes	Yes	Yes	Yes	Yes
#3003	Unclear	Yes	Yes	Yes	Yes	Yes	Yes	Yes
#2586	Yes	Yes	Yes	Yes	Yes	Yes	Yes	Yes
#602	Yes	Yes	Yes	Yes	Yes	Yes	Yes	Yes
#5260	Yes	Yes	Yes	Yes	Yes	Yes	Yes	Yes
#2888	Yes	Yes	Yes	Yes	Yes	Yes	Yes	Yes
#44	Yes	Yes	Yes	Yes	Yes	Yes	Yes	Yes
#1397	Yes	Yes	Yes	Yes	Yes	Yes	Yes	Yes
#3419	Yes	Yes	Yes	Yes	Yes	Yes	Yes	Yes
#152	Unclear	Yes	Yes	Yes	Yes	Yes	Yes	Yes
#252	Yes	Yes	Yes	Yes	Yes	Yes	Yes	Yes
#641	No	Yes	Yes	Yes	Yes	Yes	Yes	Yes
#35	Yes	Yes	Yes	Yes	Yes	Yes	Yes	Yes
#616	Yes	Yes	Yes	Yes	Yes	Yes	Yes	Yes
#4852	Yes	Yes	Yes	Yes	Yes	Yes	Yes	Yes
#693	Yes	Yes	Yes	Yes	Yes	Yes	Yes	Yes
#358	Yes	Yes	Yes	Yes	Yes	Yes	Yes	Yes
#491	Yes	Yes	Yes	Yes	Yes	Yes	Yes	Yes
#691	Yes	Yes	Yes	Yes	Yes	Yes	Yes	Yes
#47	Yes	Yes	Yes	Yes	Yes	Yes	Yes	Yes
#2339	Yes	Yes	Yes	Yes	Yes	Yes	Yes	Yes
#336	Yes	Yes	Yes	Yes	Yes	Yes	Yes	Yes
#5662	No	Unclear	Yes	Yes	Unclear	Unclear	Yes	Yes
#370	Yes	Yes	Yes	Yes	Yes	Yes	Yes	Yes
#442	Yes	Yes	Yes	Yes	Yes	Yes	Yes	Yes
#5235	Unclear	Unclear	Yes	Yes	Yes	Yes	Yes	Yes
#5	Yes	Yes	Yes	Yes	Yes	Yes	Yes	Yes
#436	Unclear	Yes	Yes	Yes	Yes	Yes	Yes	Yes
#2326	Unclear	Yes	Yes	Yes	Yes	Yes	Yes	Yes
#2340	Yes	Yes	Yes	Yes	Yes	Yes	Yes	Yes
#2527	Yes	Yes	No	Yes	Yes	Yes	Yes	Yes
#2496	No	Yes	Yes	Yes	Yes	Yes	Yes	Yes
#3188	Yes	Yes	Yes	Yes	Yes	Yes	Yes	Yes
#6487	Yes	Yes	Yes	Yes	Unclear	Unclear	Yes	Yes
#3705	Yes	Yes	Yes	Yes	Yes	Yes	Yes	Yes
#3081	No	Yes	Yes	Yes	No	No	Yes	Yes
#451	No	Yes	Yes	Yes	Yes	No	Yes	Yes
#20	Yes	Yes	Yes	Yes	Yes	Yes	Yes	Yes
#437	Yes	Yes	Yes	Yes	Yes	Yes	Yes	Yes
#418	Yes	Yes	Yes	Yes	Yes	Yes	Yes	Yes
#318	Unclear	Yes	Yes	Yes	Yes	Yes	Yes	Yes
#554	Yes	Yes	Yes	Yes	Yes	Yes	Yes	Yes
#498	Yes	Yes	Yes	Yes	Yes	Yes	Yes	Yes
#478	Unclear	Yes	Yes	Yes	Yes	Yes	Yes	Yes
#460	Yes	Yes	Yes	Yes	Yes	Yes	Yes	Yes
#738	Yes	Yes	Yes	Yes	Yes	Yes	Yes	Yes
#2379	Yes	Yes	Yes	Yes	Yes	Yes	Yes	Yes
#2368	Yes	Yes	Yes	Yes	Yes	Yes	Yes	Yes
#2356	Yes	Yes	Yes	Yes	Yes	Yes	Yes	Yes
#2341	Yes	Yes	Yes	Yes	Yes	Yes	Yes	Yes
#2912	Yes	Yes	Yes	Yes	Yes	Yes	Yes	Yes
#2881	Yes	Yes	Yes	Yes	Yes	Yes	Yes	Yes
#2868	No	Yes	Yes	Yes	Yes	Yes	Yes	Yes
#2765	Yes	Yes	Yes	Yes	Yes	Yes	Yes	Yes
#7242	Unclear	Yes	Yes	Yes	Yes	Yes	Yes	Yes
#6296	Yes	Yes	Yes	Yes	Yes	Yes	Yes	Yes
#5344	Yes	Yes	Yes	Yes	Yes	Yes	Yes	Yes
#5307	No	Yes	Yes	Yes	Yes	Yes	Yes	Yes
#4774	Yes	Yes	Yes	Yes	Yes	Yes	Yes	Yes
#4447	Yes	Yes	Yes	Yes	Yes	Yes	Yes	Yes
#3142	Yes	Yes	Yes	Yes	Yes	Yes	Yes	Yes
#3070	No	Yes	Yes	Yes	Yes	Yes	Yes	Yes
#286	Yes	Yes	Yes	Yes	Yes	Yes	Yes	Yes
#144	Yes	Yes	Yes	Yes	Yes	Yes	Yes	Yes
#2391	Yes	No	Yes	Yes	Yes	Yes	Yes	Yes
#2874	Yes	Yes	Yes	Yes	Yes	Yes	Yes	Yes
#579	Yes	Yes	Yes	Yes	Yes	Yes	Yes	Yes
#539	Yes	Yes	Yes	Yes	Yes	Yes	Yes	Yes
#2822	Yes	Yes	Yes	Yes	Yes	Yes	Yes	Yes
#4946	Yes	Yes	Yes	Yes	Yes	Yes	Yes	Yes
#355	Yes	Yes	Yes	Yes	Yes	Yes	Yes	Yes
#2293	Yes	Yes	Yes	Yes	Yes	Yes	Yes	Yes
#90	Yes	Yes	Yes	Yes	Yes	Yes	Yes	Yes
#26	Yes	Yes	Yes	Yes	Yes	Yes	Yes	Yes
#360	Yes	Yes	Yes	Yes	Yes	Yes	Yes	Yes
#323	Yes	Yes	Yes	Yes	Yes	Yes	Yes	Yes
#313	Yes	Yes	Yes	Yes	Yes	Yes	Yes	Yes
#505	Yes	Yes	Yes	Yes	Yes	Yes	Yes	Yes
#756	Yes	Yes	Yes	Yes	Yes	Yes	Yes	Yes
#2852	Yes	Yes	Yes	Yes	Yes	Yes	Yes	Yes
#2811	Yes	Yes	Yes	Yes	Yes	Yes	Yes	Yes
#7206	Yes	Yes	Yes	Yes	Yes	Yes	Yes	Yes
#5771	Yes	Yes	Yes	Yes	Yes	Yes	Yes	Yes
#3620	Yes	Yes	Yes	Yes	Yes	Yes	Yes	Yes
#3180	Yes	Yes	Yes	Yes	Yes	Yes	Yes	Yes
#3178	Yes	Yes	Yes	Yes	Yes	Yes	Yes	Yes
#3105	Yes	Yes	Yes	Yes	Yes	Yes	Yes	Yes
#3080	Yes	Yes	Yes	Yes	Yes	Yes	Yes	Yes

**Table 6:** Quality assessments for longitudinal studies using JBI critical appraisal checklist for cohort studies

ID	Were the two groups similar and recruited from the same population?	Were the exposures measured similarly to assign people to both exposed and unexposed groups?	Was the exposure measured in a valid and reliable way?	Were confounding factors identified?	Were strategies to deal with confounding factors stated?	Were the groups/participants free of the outcome at the start of the study (or at the moment of exposure)?	Were the outcomes measured in a valid and reliable way?	Was the follow up time reported and sufficient to be long enough for outcomes to occur?	Was follow up complete, and if not, were the reasons to loss to follow up described and explored?	Were strategies to address incomplete follow up utilized?	Was appropriate statistical analysis used?
#1674	Yes	Yes	Yes	Yes	Yes	Yes	Yes	Yes	Yes	Unclear	Yes
#2667	Yes	Yes	Yes	Yes	Yes	Yes	Yes	Yes	Yes	Unclear	Yes
#259	Yes	Unclear	Yes	Yes	Yes	No	Yes	Yes	No	No	Yes
#2739	Yes	Yes	Yes	Yes	Yes	No	Yes	Yes	Unclear	Unclear	Yes
#2832	Yes	Yes	Yes	Yes	Yes	Yes	Yes	Yes	Yes	Yes	Yes
#221	Yes	Yes	No	Yes	Yes	Yes	Yes	Yes	Yes	Yes	Yes
#3006	Yes	Yes	Yes	Yes	Yes	Yes	Yes	Yes	Yes	Yes	Yes
#288	Yes	Yes	Yes	Yes	Yes	Yes	Yes	Yes	Yes	Yes	Yes
#3548	Yes	Yes	No	Yes	Yes	Yes	Yes	Yes	Yes	Yes	Yes
#239	Yes	Yes	Yes	Yes	Yes	Yes	Yes	Yes	Yes	Unclear	Yes
#2849	Yes	Yes	Yes	Yes	Yes	Yes	Yes	Yes	Yes	Unclear	Yes
#278	Yes	Yes	Yes	Yes	Yes	Yes	Yes	Yes	Yes	Yes	Yes
#203	Yes	Yes	Yes	Yes	Yes	Yes	Yes	Yes	No	No	Yes
#2131	Yes	Yes	Yes	Yes	Yes	Yes	Yes	Yes	Yes	Yes	Yes
#7203	Yes	Yes	Yes	Yes	Yes	Yes	Yes	Yes	Yes	Yes	Yes
#78	Yes	No	Unclear	Yes	Yes	Yes	Yes	Yes	NA	NA	Yes
#195	Yes	Yes	Yes	Yes	Yes	Yes	Yes	Yes	Yes	Yes	Yes
#334	Yes	Yes	Yes	Yes	Yes	Yes	Yes	Yes	Yes	Yes	Yes
#6670	Yes	Yes	Yes	Yes	Yes	Yes	Yes	Yes	Yes	Yes	Yes
#372	Yes	Yes	Yes	Yes	Yes	Yes	Yes	Yes	Yes	Yes	Yes
#1278	Yes	Yes	Yes	Yes	Yes	Yes	Yes	Yes	Yes	Yes	Yes
#3282	Yes	Yes	Yes	Yes	Yes	Yes	Yes	Yes	Yes	Yes	Yes
#2773	Yes	Yes	Yes	Yes	Yes	Yes	Yes	Yes	Yes	Yes	Yes
#89	Yes	Yes	Yes	Yes	Yes	Yes	Yes	Yes	Yes	Yes	Yes
#1415	Yes	Yes	Yes	Yes	Yes	Yes	Yes	Yes	Yes	Yes	Yes
#1140	Yes	Yes	Yes	Yes	Yes	Yes	Yes	Yes	Yes	Yes	Yes
#101	Yes	Yes	Yes	Yes	Yes	No	Yes	Unclear	Yes	Yes	Yes
#883	Yes	Yes	Yes	Yes	Yes	Yes	Yes	Yes	Yes	Yes	Yes
#243	Yes	Yes	Yes	Yes	Yes	Unclear	Yes	Yes	Unclear	Unclear	Yes
#214	Yes	Yes	Yes	Yes	Yes	Unclear	Yes	Yes	Unclear	Unclear	Yes
#150	Yes	Yes	Yes	Yes	Yes	Yes	Yes	Yes	Yes	Yes	Yes
#446	Yes	Yes	Yes	Yes	Yes	Yes	Yes	Yes	Yes	Yes	Yes
#933	Yes	Yes	No	Yes	Yes	Yes	Yes	Yes	No	Unclear	Yes
#24	Yes	Yes	Yes	Yes	Yes	Unclear	Yes	Yes	Yes	Unclear	Yes
#380	Yes	Yes	Yes	Yes	Yes	Yes	Yes	Yes	Yes	Yes	Yes
#354	Yes	Yes	Yes	Yes	Yes	Yes	Yes	Yes	Yes	Yes	Yes
#56	Yes	Yes	Yes	Yes	Yes	Yes	Yes	Yes	Yes	Yes	Yes
#130	Yes	Yes	Yes	Yes	Yes	Yes	Yes	Yes	Yes	Yes	Yes

### The relationship between SEC classes and depression/anxiety

As nearly all article used multiple SEC variables in their study, the frequency for each class of SEC used in these studies was first tabulated. In addition, the relationship found between each class of SEC with depression and/or anxiety illustrated in these articles was summarized. The results were showed in Tables 7, 8, 9 and 10.

**Table 7:** SEC indicators and their relationships with depression/anxiety outcomes

SEC Indicator	Static / Fluid	Actual / Perceived	Expected	Non-Sig	Contrasting
Education	Static	Actual	95	226	33
		Perceived	2	2	0
		Unclear^a^	0	3	0
	Fluid	Actual	0	2	0
		Perceived	0	0	0
	Unclear^a^	Unclear^a^	1	1	0
Income	Static	Actual	76	107	8
		Perceived	12	12	3
		Unclear^a^	2	4	0
	Fluid	Actual	25	21	0
		Perceived	12	17	0
		Unclear^a^	3	2	0
Occupation/Employment	Static	Actual	59	108	9
		Perceived	2	1	0
		Unclear^a^	2	0	0
	Fluid	Actual	37	31	1
		Perceived	6	4	0
		Unclear^a^	1	1	0
	Unclear^a^	Unclear^a^	1	1	0
Socioeconomic Status (SES)	Static	Actual	14	12	0
		Perceived	22	21	4
		Hybrid^b^	2	0	0
	Fluid	Actual	0	0	0
		Perceived	6	3	0
	Hybrid^b^	Actual	2	0	0
Living Condition	Static	Actual	13	46	2
		Perceived	9	3	0
	Fluid	Actual	0	0	0
		Perceived	0	0	0
Food/Basic Supplies	Static	Actual	5	3	0
		Perceived	14	9	0
	Fluid	Actual	0	2	0
		Perceived	3	0	0
	Unclear^a^	Unclear^a^	0	2	0
Financial Strain	Static	Actual	11	10	1
		Perceived	59	29	1
	Fluid	Actual	11	3	0
		Perceived	33	18	0
		Hybrid^b^	1	0	0
		Unclear^a^	1	2	0
	Hybrid^b^	Actual	0	0	0
		Perceived	1	0	0
		Hybrid^b^	1	2	0
Economic-Concerns	Static	Actual	0	0	0
		Perceived	52	24	3
	Fluid	Actual	0	0	0
		Perceived	32	15	0
	Unclear^a^	Unclear^a^	6	2	0
Saving/Assets	Static	Actual	9	12	1
		Perceived	0	3	0
		Unclear^a^	0	2	0
	Fluid	Actual	3	1	0
		Perceived	0	0	0

^a^Denotes that the variable was not clearly defined in the article

^b^Denotes that the variable is a hybrid of ‘Static’ and ‘Fluid’ or ‘Actual’ and ‘Perceived’

**Table 8:** Summary of results according to SEC indicator category

SEC Indicator	Expected	Non-Significant	Contrasting
Education	98 (26.85%)	234 (64.11%)	33 (9.04%)
Income	130 (42.76%)	163 (53.62%)	11 (3.62%)
Occupation/Employment	108 (40.91%)	146 (55.30%)	10 (3.79%)
Socioeconomic Status (SES)	46 (53.49%)	36 (41.86%)	4 (4.65%)
Living Condition	22 (30.14%)	49 (67.12%)	2 (2.74%)
Food/Basic Supplies	22 (57.89%)	16 (42.11%)	0 (0.00%)
Financial Strain	118 (64.13%)	64 (34.78%)	2 (1.09%)
Economic-Concerns	90 (67.16%)	41 (30.60%)	3 (2.24%)
Savings/Assets	12 (38.71%)	18 (58.06%)	1 (3.23%)
Total	646 (43.68%)	767 (51.86%)	66 (4.46%)

**Table 9:** Summary of results according to type of SES indicator

SEC Indicator	Expected	Non-Significant	Contrasting
Static	460 (39.59%)	637 (54.82%)	65 (5.59%)
Fluid	174 (58.59%)	122 (41.08%)	1 (0.34%)
Actual	360 (36.04%)	584 (58.46%)	55 (5.51%)
Perceived	265 (60.64%)	161 (36.84%)	11 (2.52%)

**Table 10:** Summary of results according to country income level classification by world bank

SEC Indicator	Expected	Non-Significant	Contrasting
High Income	423 (48.29%)	426 (48.63%)	27 (3.08%)
Upper-Middle Income	160 (35.40%)	257 (56.86%)	35 (7.74%)
Lower-Middle Income	106 (41.57%)	141 (55.29%)	8 (3.14%)
Low Income	19 (42.22%)	25 (55.56%)	1 (2.22%)

Above classification has been adopted from Fantom and Serajuddin [56]

Our results showed that not all SEC indicators were consistently predicting emotional health outcomes during the Covid-19 pandemic, with some (e.g., economic concerns) performing better than others (e.g., education). From Table 8, we can see that across 362 studies with a total of 1479 SEC indicators used, there were only 646 (43.68%) ‘expected’ (i.e., higher SEC predicting better mental health outcomes) results. Conversely, there were 767 (51.86%) non-significant and 66 (4.46%) ‘contrasting’ (i.e., higher SEC predicting worse mental health outcomes) results. Interestingly, this trend was found in both high income, upper-middle and lower-middle income countries, with 48.63% of studies in high income countries, 56.86% in upper-middle and 55.29% in lower-middle income countries finding non-significant results. This trend was also found in low-income countries, with 55.56% of studies in these countries finding non-significant results (please see Table 10). However, the number of studies conducted in low-income countries was notably limited and therefore, should be interpreted with caution.

#### SEC Categories and mental health outcomes

In terms of SEC categories (please refer to Table 2), economic concerns as well as financial strain clusters were found to be the most likely predictors of emotional health outcomes. To illustrate, 67.16% of studies reported that economic concerns, such as financial worry [57], financial security stress [58], and concerns about future economic scenario [59] had a significant ‘expected’ relationship with depression/anxiety outcomes. Similarly, 64.13% of studies reported that financial strain, such as economic burden [60], financial problems [61], and ability to meet expenses during lockdown [62] had a significant ‘expected’ relationship with depression/anxiety outcomes.

Conversely, living conditions and education were found to be the least likely to predict emotional health outcomes. 67.12% of studies reported that living conditions such as size of house [63], area of residence (urban or rural) [64], and neighborhood overall environment quality level [65] had no significant relationship with depression/anxiety outcomes. Similarly, 64.11% of studies reported that educational attainment including number of years of education received [66] and having been to college or not [67] had no significant relationship with depression/anxiety outcomes.

#### Static and fluid SEC indicators and emotional health outcomes

From Table 9, we can see that ‘fluid’ SEC indicators (i.e., measurements that assessed changes in SEC over a period of time) were more likely to predict depression/ anxiety outcomes compared to ‘static’ SEC indicators (i.e., measurements that assessed SEC at a single time-point). To illustrate, 58.59% of studies reported that ‘fluid’ SEC indicators such as loss of employment [68] and reduced family income [69] had a significant ‘expected’ relationship with depression/anxiety outcomes, whereas only 39.59% of studies reported the same for ‘static’ SEC indicators such as current employment status [70] and monthly income [71].

#### Actual and perceived SEC indicators and emotional health outcomes

From Table 9, ‘Perceived’ (i.e., subjectively assessed) SEC indicators were found to be more likely to predict depression/anxiety compared to ‘actual’ (i.e., objectively assessed) SEC indicators. 60.64% of studies reported that ‘perceived’ SEC indicators such as self-reported food insecurity [72] and SEC assessed by the MacArthur Scale of Subjective Social Status [73] had a significant ‘expected’ relationship with depression/anxiety outcomes, whereas only 36.04% of studies reported the same for ‘actual’ SEC indicators such as food security measured by Household Food Security Survey Module [74] and SEC assessed by an asset-based index [75].

## Discussion

Our comprehensive systematic review has identified a wide-array of studies using heterogeneous indicators to predict symptoms of anxiety and depression throughout Covid-19. Despite the variability in measures, our results revealed general patterns that seem to challenge the widely accepted social gradient in mental health and theory of fundamental causes.

### Differences in predictive power of sec indicators

First, we have uncovered that not all SEC indicators were strongly predictive of emotional health symptoms during Covid-19, as majority of studies conducted across the globe reported non-significant relationships between the two variables regardless of country income classification. This contradicts pre-Covid-19 findings reporting moderate-to-strong associations between socioeconomic standing and subjective well-being and/or mental health [12–15]. Overall, around 40% of studies aligned with the social gradient in mental health theory, but more than 50% revealed no significant results. However, using economic concerns as a measure of SEC showed that the social gradient theory is still applicable for most studies.

These findings suggest that the relationship between SEC and mental health may vary in accordance to how SEC was assessed and measured. Our findings are corroborated by one pre-pandemic study [76], which report that self-reported physical health is more intertwined with SEC compared to mental health. This could be due to mental health being more influenced by internal factors such as psychological state or personality, compared to external factors such as SEC.

In the context of Covid-19, lockdown measures may have equalised the risk for mental health conditions as those from higher social classes would have been unable to utilise economic resources to mitigate health concerns and loss of freedom. This is a notion consistent with the theory of fundamental causes described in the introduction section of this review. Indeed, pandemic related stressors may have impacted individuals regardless of socioeconomic class. Uncertainty caused by the pandemic may have been more detrimental to mental health compared to one’s SEC [77], and difficulty coping with uncertainty is a common trait across various mood and anxiety disorders [78, 79].

Reduced access to and availability of mental health services may have also played a role in people of all social classes developing symptoms of anxiety and depression. At the height of the pandemic, countries and health organisations were forced to redirect funding, space, equipment, and facilities towards treating patients experiencing Covid-19 complications. Indeed, a survey by the WHO [80] found that the COVID-19 pandemic has disrupted or halted critical mental health services in 93% of countries worldwide. Moreover, due to social distancing measures, 67% saw disruptions to counselling and psychotherapy appointments, 65% to critical harm reduction services, and 45% to opioid agonist maintenance treatment for opioid dependence. Thus, those with a history of mental health conditions likely experienced worsened symptoms, while those who developed symptoms during the pandemic were unable to access urgent care and treatment, leading to a global mental health crisis transcending SEC.

### Economic concerns and financial strain

In our systematic review, the SEC category, ‘economic concerns’, emerged as being most predictive of emotional health during Covid-19, based on percentage of papers reporting significant relationships. Relevant papers revealed that the construct assessed under this category centred around ‘concerns, worries or stress arising from current or future uncertainty about one’s economic position’, although the items can be phased quite differently, e.g., ‘fear of job loss’, ‘financial insecurity’ etc. This construct may be closely linked with worrying, a transdiagnostic construct that has been shown to be robustly predictive of depression and anxiety [81, 82], though in this aspect, such worrying is economically-induced.

Defined as the tendency to dwell on uncertainty of future problems or events in an obsessive, repetitive and negative manner [83], worrying, particularly pathological worrying, is associated with the onset and intensity of mood and anxiety disorders [84–86]. Constant worrying functions like rumination, which takes up variable cognitive resources, resulting in depleted cognitive functioning abilities that are necessary for daily life [87–89]. Such cognitive deficits are expected to result in reduced problem-solving abilities, leading to adverse life circumstances, which would consequently affect one’s mental health. As illustrated by allostatic load theory, when cumulative effects of life stressors exceed a person’s buffer to cope or adapt, an allostatic overload occurs which results in poorer physical and mental health [90].

In conjunction, several studies in our review reported significant links between emotional health and the financial strain cluster, which encompasses one’s ability to pay bills/rent/mortgage, having sufficient funds to retire, as well as one’s perceived financial state, and financial wellbeing. This is consistent with pre-Covid 19 studies reporting that perceived inability to pay bills or afford food is associated with greater anxiety, depression, stress, feelings of isolation, and alcohol dependence [91–93].

Financial strain may serve as a superior predictor of mental health as it intersects both objective and subjective SEC measures. This means it reflects changes in one’s objective circumstances (e.g., ability to pay bills), while also encompassing subjective measures (e.g., satisfaction with finances) that exerts influence over mental health [94].

Empirically, decreased objective financial resources was found to be associated with increased financial strain, and in turn, financial strain emerged as a strong and robust predictor of poor mental health in older adults [94]. Financial strain and economic concerns being perceived measures also likely strengthens their ability to predict mental health since our review has found that perceived indicators are more correlated with anxiety and depression than objective ones. This will be discussed further in the section entitled ‘Perceived and Objective SEC Indicators’.

### Fluid and static SEC indicators

Research done prior to Covid-19 has reported that fluid indicators of SEC (e.g., job loss or income loss) are highly predictive of poor mental health outcomes [95–97]. The inverse has also been observed, wherein income gains (via, for instance, increasing minimum wage) lead to stark improvement in mental health symptoms [98]. However, to our knowledge, our review is the first to clarify that fluid SEC indicators may be more informative of changes in emotional health compared to static indicators (e.g., current income or occupation) during Covid-19.

A sudden negative change in income or employment would profoundly impact one’s lifestyle; affected individuals would be forced to alter spending habits, cut back on leisure to focus on saving for essential goods, or even resort to drastic measures such as removing their children from school. This period of adjustment can culminate in severe stress. In addition, the shame and stigma associated with job loss or unemployment can also lead to depressive feelings [99]. By contrast, people who have had consistently low income or have been unemployed pre-pandemic may be more resilient to declines in mental health linked to Covid-19 as they are more accustomed to lifestyles associated with poverty.

### Perceived and objective SEC indicators

Next, consistent with prior literature we report that perceived SEC indicators (e.g., measures used for financial strain) correlate more with emotional health outcomes compared to objective indicators. This phenomenon has been observed across continents including in Asia and Europe [76, 100, 101]. Additionally, a current meta-analysis across 357 studies found that subjective SEC corresponded to subjective well-being better than income or educational attainment [15]. Research also reports that objective SEC only affects mental health via promoting changes in subjective SEC [100] suggesting that subjective or perceived SEC serves as an important mediating variable.

One potential reason for perceived SEC being a superior predictor of mental health outcomes is that it perhaps serves as a more precise measure of social position. This is because perceived SEC considers not only current social standing, but past contexts and future prospects [102]. As an example, two individuals with post-graduate qualifications may be considered similar in social standing based on objective measures of SEC. However, if one of them graduated from a less prestigious university, they may rate their subjective SEC as being lower due to future financial and career prospects not being as lucrative.

Another reason is that subjective SEC appears to have more of an influence over physiological stress pathways, as perceiving oneself as financially disadvantaged impacts the hypothalamic–pituitary–adrenal (HPA) axis leading to enhanced production of cortisol [103–105]. Dysregulated HPA is also observed in clinical depression [106]. Thus, poor SEC may be elevating mental health symptoms via dysregulating the HPA axis [101].

### Living conditions and education

Lastly, we have observed that living conditions and education level appear weakly predictive of anxiety and depression symptoms. Work published prior to Covid-19 congruently report mixed findings pertaining to these indicators. On one hand, education, particularly parental education, and crowding (an indicator of living condition) is reported to predict children and adolescent mental health outcomes [107, 108]. In contrast, other systematic reviews and meta-analysis report that education and neighbourhood living conditions do not strongly predict mental-health and well-being [76, 109]. The lack of predictive power may be attributed to these being objective SEC measures which, as highlighted above, do not influence emotional health as strongly as perceived indicators.

The weak link we have observed between education and emotional health may be considered surprising at first as high educational attainment typically leads to lower rates of unemployment and occupations that provide economic resources beneficial to quality of life [110]. Nevertheless, the association between emotional health and education is unlikely to be linear. Research has instead revealed that at higher levels of educational attainment, additional increases in formal education is decreasingly beneficial for mental health [111]. For instance, advancing from an undergraduate to a post-graduate qualification is less significant for mental health compared to going from primary to secondary level of education [111]. Moreover, being overeducated is reported to lead to diminishing mental health, as it is associated with decreased job satisfaction, increased job stress, and greater prevalence of depressive symptoms [24, 111–113]. This may, in part, be due to a skills mismatch and overeducated people feeling under-challenged in their careers [114].

Additionally, it is perhaps difficult to detect a strong effect of living conditions on emotional health due to the studies in our review using extremely varied measures that may not be capturing the same construct or even indeed socioeconomic constructs, including rural versus urban housing, crowding, noise levels, presence of balcony or garden, number of rooms, area of house, etc. Hence, it is difficult to isolate a specific variable that could be most predictive of emotional health within this SEC indicator.

## Conclusion

In conclusion, our systematic review revealed that there are differential effects of various classes of SEC indicators in predicting emotional health. Notably, the economic concerns and financial strain clusters emerged as stronger predictors of depression and anxiety. Surprisingly, classic SEC measures, such as education and income, did not exhibit strong predictability during this period. In addition, ‘fluid’ and ‘perceived’ class of SEC indicators have been shown to display better predictive power on depression and anxiety as compared to ‘static’ and ‘actual’. These findings suggest that the strength of the association between SEC and mental health is dependent upon the class of SEC indicator used.

### Limitations

While our systematic review has compellingly unveiled how diverse SEC indicators differentially affect emotional health during Covid-19, it is not without limitations. First, because of the heterogeneity in measures, we were unable to conduct a meta-analysis to elucidate whether observed trends in studies show statistical significance. Next, our review only included studies with data collected during the pandemic, and hence we could not compare pre-Covid and post-Covid findings in more detail to truly determine whether Covid-19 has resulted in differences in how SEC indicators are predictive of emotional health symptoms. In addition, since our review has only included articles on depression and/or anxiety as the overall gauge of the emotional health status of the general population, we could have missed out articles that studied on other specific mental health disorders during the pandemic, and we could not rule out the linkages between SEC with these specific mental disorders might be different compared to general emotional health as revealed in our review. Thus, though our review has interrogated an important link between SEC and anxiety/depressive symptoms, we acknowledge that it would be imperative in future work to probe other mental health disorders that were also affected by the pandemic. Also, our review only includes articles that were published in journals or in pre-print servers as of November 11, 2021. Articles published after this date were not included in this review.

### Articles included in this review

**Table:** 

1) Silva [115]	2) Bhandari [116]	3) Guerin [117]
4) Alharbi [118]	5) Elezi [119]	6) Campos [120]
7) Dawel [66]	8) Agberotimi [121]	9) Chakraborty [62]
10) Al Zabadi [71]	11) Balakrishnan [122]	12) d'Arqom [123]
13) Bower [70]	14) Chen [65]	15) Alkhamshi [124]
16) Généreux [125]	17) Hall [126]	18) Islam [127]
19) Brouillette [128]	20) Frankel [58]	21) He [129]
22) Badellino [130]	23) da Silva Júnior [131]	24) Sharif Nia [132]
25) Hoque [133]	26) Aruta [134]	27) Dharra [135]
28) Cui [136]	29) Graupensperger [137]	30) Chasson [138]
31) Gong [139]	32) Cost [140]	33) Luo [141]
34) Ettman [142]	35) Hajek [143]	36) Han [144]
37) Heanoy [68]	38) Brunoni [145]	39) Ali [146]
40) De Pietri [73]	41) Ren [147]	42) Hueniken [57]
43) Karing [148]	44) Gouvernet [149]	45) Betini [150]
46) Barcellos [67]	47) Elhadi [151]	48) Ames-Guerrero [152]
49) Ali [61]	50) Hu [153]	51) Harjana [154]
52) Gong [155]	53) Bérard [156]	54) De France [157]
55) Frontera [158]	56) Alpay [159]	57) Hammarberg [160]
58) Conti [161]	59) Irfan [69]	60) Jewell [162]
61) Dhar [163]	62) Ahmmed [164]	63) Figueroa-Quiñones [165]
64) Islam [166]	65) Pensgaard [167]	66) Al Mutair [168]
67) Cevher [169]	68) Chen [60]	69) Zhang [170]
70) Ahmed [171]	71) Karaivazoglou [172]	72) Basheti [173]
73) Flores [174]	74) Abrams [175]	75) Chen [176]
76) Hart [177]	77) Bahar Moni [178]	78) Cao [179]
79) Huang [180]	80) Guo [181]	81) Effati-Daryani [182]
82) Every-Palmer [183]	83) Cortés-Álvarez [184]	84) Fitzpatrick [185]
85) Gur [186]	86) Fu [187]	87) Akkaya-Kalayci [188]
88) Díaz-Jiménez [59]	89) Ben-Kimhy [189]	90) Goularte [190]
91) Fu [191]	92) Ganson [192]	93) Gloster [193]
94) Donnelly [194]	95) Delmastro [195]	96) Fountoulakis [196]
97) Simha [197]	98) Antiporta [198]	99) Hou [199]
100) Fornili [200]	101) Das [201]	102) Fukase [202]
103) Fanaj [203]	104) Blix [204]	105) Feter [205]
106) Harling [206]	107) Batterham [207]	108) Hao [208]
109) Haliwa [209]	110) Cerecero-Garcia [210]	111) Creese [211]
112) Alkhaldi [212]	113) Hoyt [213]	114) Beach [214]
115) Oh [215]	116) BC [216]	117) Davis [217]
118) Beutel [218]	119) Kim [219]	120) Dehkordi [220]
121) Hu [221]	122) Mekhemar [222]	123) Hoffart [223]
124) Adnine [224]	125) Bryson [225]	126) Geren [226]
127) Angwenyi [75]	128) Guerrero [227]	129) Fröhlich [228]
130) Coley [229]	131) Law [230]	132) Sun [231]
133) Heo [232]	134) Hertz-Palmor [233]	135) Lindau [234]
136) Ruengorn [235]	137) Repon [236]	138) Owens [237]
139) Varma [238]	140) Stampini [239]	141) Esteban-Gonzalo [240]
142) Mistry [241]	143) Schmits [242]	144) Feurer [243]
145) Mana [244]	146) Marmet [245]	147) Wright [246]
148) Kobayashi [247]	149) Oginni [248]	150) Maffly-Kipp [249]
151) Shuster [250]	152) Ogrodniczuk [251]	153) Moya [252]
154) Kjeldsted [253]	155) Ochnik [254]	156) Robertson [255]
157) Senturk [256]	158) Sams [257]	159) Sirin [258]
160) Lueck [259]	161) Ribeiro [260]	162) Tasnim [261]
163) Zhang [262]	164) Rudenstine [263]	165) Zheng [264]
166) Shalhub [265]	167) Vujčić [266]	168) Liang [267]
169) Nagasu [268]	170) Vicens [269]	171) Posel[270]
172) Romeo [271]	173) Thombs [272]	174) Wong [273]
175) Meng [274]	176) Porter [275]	177) Knolle [276]
178) Jones [277]	179) Wickens [278]	180) Racine [279]
181) Mikocka-Walus [280]	182) Thayer [281]	183) Landa-Blanco [282]
184) van Rüth [283]	185) Koyucu [284]	186) Ettman [285]
187) Kämpfen [286]	188) Jacques-Aviñó [287]	189) Li [288]
190) Xiao [289]	191) Ueda [290]	192) Garre-Olmo [291]
193) Polsky [292]	194) Silverman [293]	195) Lin [294]
196) Sinawi [295]	197) Kar [296]	198) Serafini [297]
199) Yörük [298]	200) Reagu [299]	201) Khademian [300]
202) Reading Turchioe [301]	203) Saito [302]	204) Torrente [303]
205) Wong [304]	206) Yang [305]	207) Wang [306]
208) Khoury [307]	209) Trujillo-Hernández [308]	210) Luo [309]
211) Wang [310]	212) Lee [311]	213) Lei [312]
214) Ping [313]	215) Solomou [314]	216) Song [315]
217) García-Fernández [316]	218) Gasparro [317]	219) Newby [318]
220) McCracken [319]	221) Islam [320]	222) Zhao [321]
223) Winkler [322]	224) Shatla [323]	225) Massad [324]
226) Iob [325]	227) Nelson [326]	228) Généreux [327]
229) Kuang [328]	230) Smith [329]	231) Khan [330]
232) Van Hees [331]	233) Ballivian [332]	234) Wilson [333]
235) Montano [334]	236) Skapinakis [335]	237) Yáñez [336]
238) Shevlin [337]	239) Wanberg [24]	240) Sayeed [338]
241) Wang [339]	242) Wang [340]	243) Marthoenis [341]
244) Toledo-Fernández [342]	245) Tuan [343]	246) Sisay [344]
247) Mekhemar [345]	248) Klimkiewicz [346]	249) Mâsse [347]
250) Watkins-Martin [348]	251) Nam [349]	252) Malesza [350]
253) Prati [63]	254) López-Castro [351]	255) Ettman [352]
256) Kwong [353]	257) Minhas [354]	258) Meraya[355]
259) Rutland-Lawes [64]	260) Garvey [356]	261) Yao [357]
262) Lai [358]	263) Sultana [359]	264) Wang [360]
265) Liu [361]	266) Kohls [362]	267) Millevert [363]
268) Zheng [364]	269) Passavanti [365]	270) Peng [366]
271) Yan [367]	272) Liu [368]	273) Zhao [369]
274) Vrublevska [370]	275) Shen [371]	276) Kinser [372]
277) Rondung [373]	278) Yadav [374]	279) Song [375]
280) Sazakli [376]	281) Jolliff [377]	282) Hyun [378]
283) Krumer-Nevo [379]	284) Pagorek-Eshel [380]	285) Mistry [381]
286) Kaplan Serin [382]	287) Leaune [383]	288) McDowell [384]
289) Killgore [385]	290) Qiu [386]	291) Zhou [387]
292) Kira [388]	293) Su [389]	294) Wolfson [390]
295) Mekhemar [391]	296) King [392]	297) Teng [393]
298) Saw [394]	299) Oryan [395]	300) Kim [396]
301) Qiu [397]	302) Zhao [398]	303) Miklitz [399]
304) Sundermeir [400]	305) Gangwar [401]	306) Mojtahedi [402]
307) Obrenovic [403]	308) McArthur [404]	309) Oliva [405]
310) Wagner [406]	311) Shahriarirad [407]	312) Okubo [408]
313) Jia [409]	314) Kikuchi [410]	315) Robinson [411]
316) Olibamoyo Olushola [412]	317) Suleiman [413]	318) Mani [414]
319) López Steinmetz [415]	320) Widyana [416]	321) Thomas [417]
322) Li [418]	323) Salameh [419]	324) Zajacova [72]
325) Yamamoto [420]	326) Zwickl [421]	327) Gama [422]
328) Solomou [423]	329) Malek Rivan [424]	330) Nishimura [425]
331) Sabat [426]	332) Torkian [427]	333) Suarez-Balcazar [74]
334) Westrupp [428]	335) Li [429]	336) Scarlett [430]
337) Pinchoff [431]	338) Tsai [432]	339) Msherghi [433]
340) Myhr [434]	341) Kusuma [435]	342) Rassu [436]
343) Sabate [437]	344) Lee [438]	345) Jing [439]
346) Shangguan [440]	347) Liu [441]	348) Van de Velde [442]
349) Saadeh [443]	350) Ravens-Sieberer [444]	351) Mikolajczyk [445]
352) Santangelo [446]	353) Morin [447]	354) Spiro [448]
355) Kira [449]	356) Leach [450]	357) Juchnowicz [451]
358) Song [452]	359) Emery [453]	360) Zarrouq [454]
361) Restar [455]	362) Smallwood [456]	

### Supplementary Information

**Supplementary Material 1.**

**Supplementary Material 2.**

## Data Availability

All data generated or analysed during this study are included in Supplementary Material 2 and Tables 4, 5, 6, 7, 8, 9 and 10.

## Abbreviations

CES-D: Center for Epidemiologic Studies Depression Scale
DSM-5: Diagnostic and Statistical Manual of Mental Disorders, Fifth Edition
EW: Economic-worries
HPA: Hypothalamic–pituitary–adrenal
ICD-10: International Classification of Diseases, 10th Revision
JBI: Joanna Briggs Institute
PHQ: Patient Health Questionnaire
PRISMA: Preferred Reporting Items for Systematic Reviews and Meta-Analyses
SARS-CoV-2: Acute respiratory syndrome coronavirus 2
SEC: Socioeconomic conditions
STAI: State-Trait Anxiety Inventory
WHO: World health organization
