# Supplementary material for: A systematic review on the relationship between socioeconomic conditions and emotional disorder symptoms during Covid-19: unearthing the potential role of economic concerns and financial strain
Source: BMC Psychol. 2024 Apr 26;12:237. doi: 10.1186/s40359-024-01715-8 (PMC11046828; doi:10.1186/s40359-024-01715-8)
Supplement: Supplementary file 1 — Supplementary Material 1. [file 40359_2024_1715_MOESM1_ESM.docx]

**Appendices**

Appendix A

Characteristics of the Included Countries

| **Country** | **The number of times a population included** | | **Region/Continent** | **Global North or South** | | **High, Upper-Middle, Lower-Middle or Low Income (in accordance to World Bank)** | |
| --- | --- | --- | --- | --- | --- | --- | --- |
| Albania | | 1 | Europe & Central Asia | | North | | Upper-Middle Income |
| Algeria | | 1 | Middle East & North Africa | | South | | Lower-Middle Income |
| Andorra | | 1 | Europe & Central Asia | | North | | High Income |
| Angola | | 2 | Sub-Saharan Africa | | South | | Lower-Middle Income |
| Argentina | | 5 | Latin America & Caribbean | | South | | Upper-Middle Income |
| Australia | | 12 | East Asia & Pacific | | North | | High Income |
| Austria | | 3 | Europe & Central Asia | | North | | High Income |
| Azerbaijan | | 1 | Europe & Central Asia | | North | | Upper-Middle Income |
| Bangladesh | | 17 | South Asia | | South | | Lower-Middle Income |
| Belarus | | 1 | Europe & Central Asia | | North | | Upper-Middle Income |
| Belgium | | 5 | Europe & Central Asia | | North | | High Income |
| Brazil | | 12 | Latin America & Caribbean | | South | | Upper-Middle Income |
| Cabo Verde | | 1 | Sub-Saharan Africa | | North | | Lower-Middle Income |
| Canada | | 27 | North America | | North | | High Income |
| Chile | | 2 | Latin America & Caribbean | | South | | High Income |
| China | | 48 | East Asia & Pacific | | South | | Upper-Middle Income |
| Colombia | | 4 | Latin America & Caribbean | | South | | Upper-Middle Income |
| Costa Rica | | 1 | Latin America & Caribbean | | South | | Upper-Middle Income |
| Croatia | | 2 | Europe & Central Asia | | North | | High Income |
| Cyprus | | 4 | Europe & Central Asia | | North | | High Income |
| Czech Republic | | 5 | Europe & Central Asia | | North | | High Income |
| Denmark | | 3 | Europe & Central Asia | | North | | High Income |
| Ecuador | | 2 | Latin America & Caribbean | | South | | Upper-Middle Income |
| Egypt | | 2 | Middle East & North Africa | | South | | Lower-Middle Income |
| El Salvador | | 1 | Latin America & Caribbean | | South | | Upper-Middle Income |
| Ethiopia | | 2 | Sub-Saharan Africa | | South | | Low Income |
| Finland | | 4 | Europe & Central Asia | | North | | High Income |
| France | | 10 | Europe & Central Asia | | North | | High Income |
| Germany | | 15 | Europe & Central Asia | | North | | High Income |
| Greece | | 7 | Europe & Central Asia | | North | | High Income |
| Guadeloupe | | 1 | North America | | South | | Low Income |
| Honduras | | 1 | Latin America & Caribbean | | South | | Lower-Middle Income |
| Hong Kong | | 7 | East Asia & Pacific | | South | | High Income |
| Hungary | | 2 | Europe & Central Asia | | North | | High Income |
| Iceland | | 2 | Europe & Central Asia | | North | | High Income |
| India | | 9 | South Asia | | South | | Lower-Middle Income |
| Indonesia | | 5 | East Asia & Pacific | | South | | Upper-Middle Income |
| Iran | | 10 | Middle East & North Africa | | South | | Lower-Middle Income |
| Iraq | | 1 | Middle East & North Africa | | South | | Upper-Middle Income |
| Ireland | | 4 | Europe & Central Asia | | North | | High Income |
| Israel | | 12 | Middle East & North Africa | | North | | High Income |
| Italy | | 15 | Europe & Central Asia | | North | | High Income |
| Japan | | 13 | East Asia & Pacific | | North | | High Income |
| Jordan | | 6 | Middle East & North Africa | | South | | Lower-Middle Income |
| Kenya | | 2 | Sub-Saharan Africa | | South | | Lower-Middle Income |
| Kosovo | | 2 | Europe & Central Asia | | North | | Upper-Middle Income |
| Kuwait | | 2 | Middle East & North Africa | | South | | High Income |
| Latvia | | 2 | Europe & Central Asia | | North | | High Income |
| Lebanon | | 2 | Middle East & North Africa | | South | | Lower-Middle Income |
| Liberia | | 1 | Sub-Saharan Africa | | South | | Low income |
| Libya | | 1 | Middle East & North Africa | | South | | Upper-Middle income |
| Liechtenstein | | 1 | Europe & Central Asia | | North | | High income |
| Luxembourg | | 1 | Europe & Central Asia | | North | | High income |
| Malaysia | | 7 | East Asia & Pacific | | South | | Upper-Middle income |
| Maldives | | 1 | South Asia | | South | | Upper-Middle income |
| Mexico | | 8 | Latin America & Caribbean | | South | | Upper-Middle income |
| Montenegro | | 1 | Europe & Central Asia | | North | | Upper-Middle income |
| Morocco | | 2 | Middle East & North Africa | | South | | Lower-Middle income |
| Myanmar | | 1 | East Asia & Pacific | | South | | Lower-Middle income |
| Namibia | | 1 | Sub-Saharan Africa | | South | | Upper-Middle income |
| Nepal | | 2 | South Asia | | South | | Lower-Middle income |
| Netherlands | | 5 | Europe & Central Asia | | North | | High income |
| New Zealand | | 3 | East Asia & Pacific | | North | | High income |
| Nigeria | | 4 | Sub-Saharan Africa | | South | | Lower-Middle income |
| North Macedonia | | 1 | Europe & Central Asia | | North | | Upper-Middle income |
| Norway | | 9 | Europe & Central Asia | | North | | High income |
| Oman | | 1 | Middle East & North Africa | | South | | High income |
| Pakistan | | 4 | South Asia | | South | | Lower-Middle income |
| Panama | | 1 | Latin America & Caribbean | | South | | High income |
| Paraguay | | 1 | Latin America & Caribbean | | South | | Upper-Middle income |
| Peru | | 6 | Latin America & Caribbean | | South | | Upper-Middle income |
| Philippines | | 4 | East Asia & Pacific | | South | | Lower-Middle income |
| Poland | | 6 | Europe & Central Asia | | North | | High income |
| Portugal | | 3 | Europe & Central Asia | | North | | High income |
| Qatar | | 1 | Middle East & North Africa | | South | | High income |
| Romania | | 2 | Europe & Central Asia | | North | | High income |
| Russia | | 3 | Europe & Central Asia | | North | | Upper-Middle income |
| Saudi Arabia | | 8 | Middle East & North Africa | | South | | High income |
| Serbia | | 2 | Europe & Central Asia | | North | | Upper-Middle income |
| Singapore | | 3 | East Asia & Pacific | | North | | High income |
| Slovakia | | 2 | Europe & Central Asia | | North | | High income |
| Slovenia | | 2 | Europe & Central Asia | | North | | High income |
| South Africa | | 6 | Sub-Saharan Africa | | South | | Upper-Middle income |
| South Korea | | 5 | East Asia & Pacific | | North | | High income |
| Spain | | 11 | Europe & Central Asia | | North | | High income |
| Sri Lanka | | 1 | South Asia | | South | | Lower-Middle income |
| Sweden | | 5 | Europe & Central Asia | | North | | High income |
| Switzerland | | 6 | Europe & Central Asia | | North | | High income |
| Taiwan | | 1 | East Asia & Pacific | | North | | High income |
| Thailand | | 3 | East Asia & Pacific | | South | | Upper-Middle income |
| Turkey | | 13 | Europe & Central Asia | | North | | Upper-Middle income |
| Uganda | | 2 | Sub-Saharan Africa | | South | | Low income |
| Ukraine | | 2 | Europe & Central Asia | | North | | Lower-Middle income |
| United Arab Emirates | | 2 | Middle East & North Africa | | South | | High income |
| United Kingdom | | 21 | Europe & Central Asia | | North | | High income |
| United States | | 74 | North America | | North | | High income |
| Uruguay | | 1 | Latin America & Caribbean | | South | | High income |
| Vietnam | | 5 | East Asia & Pacific | | South | | Lower-Middle income |
