# Supplementary material for: A systematic review on the relationship between socioeconomic conditions and emotional disorder symptoms during Covid-19: unearthing the potential role of economic concerns and financial strain
Source: BMC Psychol. 2024 Apr 26;12:237. doi: 10.1186/s40359-024-01715-8 (PMC11046828; doi:10.1186/s40359-024-01715-8)
Supplement: Supplementary file 2 — Supplementary Material 2. [file 40359_2024_1715_MOESM2_ESM.zip › Table 4_Revised.docx]

**Table 4**

*The Characteristics of the Studies Included in the Systematic Review*

| Study ID | First Author (Year) | Countries | Population | Study Design | Start Date | End date | Participants (N) | Age  (M, SD, Range) | Female (%, n) | Inclusion Criteria | Exclusion Criteria | | Sampling Method |
| --- | --- | --- | --- | --- | --- | --- | --- | --- | --- | --- | --- | --- | --- |
|  |  |  |  |  |  |  |  |  |  |  |  | |  |
| #6778 | Silva et al. (2021) [115] | Brazil | Undergraduate students | Cross-sectional | Middle of March, 2020 | June, 2020 | 620 | 23.0, 3.7, NA | 78% (484) | Aged >=18 years from any gender regularly enrolled in any  undergraduate course | Incomplete data | | Convenience |
|  |  |  |  |  |  |  |  |  |  |  |  | |  |
| #6255 | Bhandari et al. (2021) [116] | India | Employee | Cross-sectional | NA | NA | 167 | NA | 23.4% (39) | NA | Unemployed respondents | | Convenience |
|  |  |  |  |  |  |  |  |  |  |  |  | |  |
| #6116 | Guerin et al. (2021) [117] | United States | Adults  (>= 18 years) | Cross-sectional | June 10, 2020 | June 25, 2020 | 2,565 | 47.7, NA, 18-94. | 46.0% (1179) | NA | Fewer than half of the questions completed or those that were completed within 5 minutes. | | Random |
|  |  |  |  |  |  |  |  |  |  |  |  | |  |
| #5913 | Alharbi et al. (2021) [118] | Saudi Arabia | Public in Amdadina KSA | Cross-sectional | NA | NA | 430 | NA, NA, 15-45 | 18.1% (78) | NA | NA | | Not Stated |
|  |  |  |  |  |  |  |  |  |  |  |  | |  |
| #5493 | Elezi et al. (2020) [119] | Albania | General | Cross-sectional | April 4, 2020 | April 29, 2020 | 1,678 | 26.5, 8.1, NA | 73.2% (1229) | Age range from 18 to 60 years and living in Albania. | NA | | Convenience |
|  |  |  |  |  |  |  |  |  |  |  |  | |  |
| #5485 | Campos et al. (2020) [120] | Brazil | General | Cross-sectional | May 18, 2020 | June 25, 2020 | 12,196 | 35.2, 13.0, 18-94 | 69.8% (8513) | Brazilian individuals over 18 years of age | NA | | Snowball |
|  |  |  |  |  |  |  |  |  |  |  |  | |  |
| #5401 | Dawel et al. (2020) [66] | Australia | General | Cross-sectional | March 28, 2020 | March 31, 2020 | 1,296 | 46.0, 17.3, NA | 50.1%, (649) | NA | NA | | Quota |
|  |  |  |  |  |  |  |  |  |  |  |  | |  |
| #5398 | Agberotimi et al. (2020) [121] | Nigeria | Healthcare personnel and general | Cross-sectional | March 20, 2020 | April 19, 2020 | 884 (382 healthcare and 502 general) | NA, NA, 18-78 | 45.5% (402) | Aged 18-78 years; healthcare or Nigerian residents with access to the internet; able to read and understand in basic English language and willing to click the agree button to participate | | NA | Snowball |
|  |  |  |  |  |  |  |  |  |  |  | |  |  |

**Table 4 (Continued)**

*The Characteristics of the Studies Included in the Systematic Review*

| Study ID | First Author (Year) | Countries | Population | Study Design | Start Date | End date | Participants (N) | Age  (M, SD, Range) | Female (%, n) | Inclusion Criteria | Exclusion Criteria | | Sampling Method |
| --- | --- | --- | --- | --- | --- | --- | --- | --- | --- | --- | --- | --- | --- |
|  |  |  |  |  |  |  |  |  |  |  |  | |  |
| #5227 | Chakraborty et al. (2020) [62] | India | Dental students/ practitioners | Cross-sectional | May 1 2020 | May 10, 2020 | 335 | 27.0, 5.0, NA | 72% (240) | Dental students and practitioners | NA | | Convenience |
|  |  |  |  |  |  |  |  |  |  |  |  | |  |
| #5189 | Al Zabadi et al. (2020) [71] | Palestine | Adults living in West Bank/ Gaza Strip/ East Jerusalem | Cross-sectional | April 6, 2020 | April 16, 2020 | 2,819 | 29.5, 11.0, NA | 72.8% (2061) | Aged >=18 years and currently living in the West Bank, Gaza Strip, and East Jerusalem. | NA | | Snowball |
|  |  |  |  |  |  |  |  |  |  |  |  | |  |
| #5104 | Balakrishnan et al. (2021) [122] | Malaysia | Women | Cross-sectional | June 2, 2020 | June 9, 2020 | 1,793 | NA | 100% (1793) | Women aged > 24 years old. | NA | | Convenience |
|  |  |  |  |  |  |  |  |  |  |  |  | |  |
| #4928 | d'Arqom et al. (2021) [123] | Indonesia | Mothers | Cross-sectional | July, 2020 | December, 2020 | 610 | NA | 100% (610) | Mothers with school-age  children (elementary-high school); > 18 years old; domiciled in Indonesia during the Covid-19 pandemic | Not Indonesian; without school-age children; < 18 years old; domiciled outside Indonesia during the COVID-19 pandemic | | Cluster |
|  |  |  |  |  |  |  |  |  |  |  |  | |  |
| #4908 | Bower et al. (2021) [70] | Australia | General and people experiencing housing issues or homelessness | Mixed-Method | July 7, 2020 | December 31, 2020 | 2,065 | Me = 43.9, SE = .36, 18-88 | 66.3% (1370) | NA | Did not speak sufficient English to complete the survey | | Convenience plus purposive |
|  |  |  |  |  |  |  |  |  |  |  |  | |  |
|  |  |  |  |  |  |  |  |  |  |  |  | |  |
| #4660 | Chen et al. (2021) [65] | China | General | Cross-sectional | January 20, 2020 | February 13, 2020 | 937 | NA | 65.3% (612) | NA | Invalid information in the options that related to quarantine location. | | Convenience |
|  |  |  |  |  |  |  |  |  |  |  |  | |  |
| #4501 | Alkhamshi et al. (2021) [124] | Saudi Arabia | General | Cross-sectional | Start June, 2020 | End June, 2020 | 1,624 | N/A, N/A, 18 to <=60 | 46.3% (752) | NA | NA | | Snowball |
|  |  |  |  |  |  |  |  |  |  |  | |  |  |
| #3940 | Généreux et al. (2021) [125] | Canada | Adults | Cross-sectional | April 8, 2020 | April 11, 2020 | 600 | NA | NA | Aged >=18 years; living in Canada; answer online. | | NA | Quota |
|  |  |  |  |  |  |  |  |  |  |  |  | |  |

**Table 4 (Continued)**

*The Characteristics of the Studies Included in the Systematic Review*

| Study ID | First Author (Year) | Countries | Population | Study Design | Start Date | End date | Participants (N) | Age  (M, SD, Range) | Female (%, n) | Inclusion Criteria | Exclusion Criteria | Sampling Method |
| --- | --- | --- | --- | --- | --- | --- | --- | --- | --- | --- | --- | --- |
|  |  |  |  |  |  |  |  |  |  |  |  |  |
| #3883 | Hall et al. (2021) [126] | United States | Adults | Cross-sectional | June 22, 2020 | July 5, 2020 | 5,023 | NA | 55.8% (2805) | Aged >=18 years; sufficient English to complete the survey. | NA | Convenience |
|  |  |  |  |  |  |  |  |  |  |  |  |  |
| #3555 | Islam et al. (2021) [127] | Bangladesh | Impoverished urban residents | Cross-sectional | August, 2020 | September, 2020 | 435 | 45.0, 12.0, 18-85 | 45.3% (197) | Aged >=18 years; willing to enrol | Being <18 years; no consent; incomplete surveys | Convenience |
|  |  |  |  |  |  |  |  |  |  |  |  |  |
| #3548 | Brouillette et al. (2021) [128] | Canada | Older adults living with HIV | Cohort | Mid-April, 2020 | June 30, 2020 | 77 | 57.3, 7.0, NA | 10.4% (8) | Living in Montreal;  agreed to be contacted for sub-studies; had access to internet; free from dementia, neurological disorders; substance-use disorder | NA | Convenience |
|  |  |  |  |  |  |  |  |  |  |  |  |  |
| #3536 | Frankel et al. (2021) [58] | United States | Parents | Cross-sectional | April, 2020 | June, 2020 | 119 | 36.3, 4.4, NA | 46.2% (55) | NA | NA | Convenience |
|  |  |  |  |  |  |  |  |  |  |  |  |  |
| #3531 | He et al. (2021) [129] | United States | Low-income parents | Cross-sectional | July, 2020 | September, 2020 | 247 | 31.3, 6.3, NA | 55.5 (137) | First-time parents of a baby less than 9 months; be cohabitating; aged >18 years; making < $75,000 per year; literate at first-grade reading level in either English or Spanish | NA | Convenience |
|  |  |  |  |  |  |  |  |  |  |  |  |  |
| #3451 | Badellino et al. (2021) [130] | Argentina | General | Repeated cross-sectional | T1: March 29, 2020  T2: May 23, 2020 | T1: April 12, 2020  T2: June 12, 2020 | T1: 1,985  T2: 2,839 | T1: 36.8, 14.4, NA  T2: 28.0, 12.2, NA | T1: 75.8% (1505)  T2: 75.3% (2137) | Aged >18years of age; living in Argentina | Having a previous mental disorder; having dyslexia | Snowball |
|  |  |  |  |  |  |  |  |  |  |  |  |  |
|  |  |  |  |  |  |  |  |  |  |  |  |  |

**Table 4 (Continued)**

*The Characteristics of the Studies Included in the Systematic Review*

| Study ID | First Author (Year) | Countries | Population | Study Design | Start Date | End date | Participants (N) | Age  (M, SD, Range) | Female (%, n) | Inclusion Criteria | Exclusion Criteria | Sampling Method |
| --- | --- | --- | --- | --- | --- | --- | --- | --- | --- | --- | --- | --- |
|  |  |  |  |  |  |  |  |  |  |  |  |  |
| #3366 | da Silva Júnior et al. (2021) [131] | Brazil | University students | Cross-sectional | October 27, 2020 | December 11, 2020 | 5,879 | 24.1, 6.4, NA | 73.5% (4324) | Undergraduate students from any area of knowledge, of both sexes, aged 18-59 years | Bariatric-surgery;  pregnant/ lactating;  incomplete/duplicate questionnaires;  did not report or declared race/skin colour as yellow or indigenous; unlikely data | Random |
|  |  |  |  |  |  |  |  |  |  |  |  |  |
| #3309 | Sharif Nia et al. (2021) [132] | Iran | General | Cross-sectional | March 18, 2020 | March 25, 2020 | 70,180 | 41.2, 11.7, N/A | 64.3%, (45143) | Access to the web and literate | NA | Snowball |
|  |  |  |  |  |  |  |  |  |  |  |  |  |
| #3284 | Hoque et al. (2021) [133] | Bangladesh | University students | Cross-sectional | March 13, 2021 | April 5, 2021 | 206 | 20.64, 1.35, NA | 53.4% (110) | Undergraduate student in a public university. | NA | Convenience |
|  |  |  |  |  |  |  |  |  |  |  |  |  |
| #3272 | Aruta (2021) [134] | Philippines | Adults | Cross-sectional | Apr, 2020 | May, 2020 | 401 | 30.1, 11.2, 18-68 | 63.34% (254) | NA | NA | Convenience and snowball |
|  |  |  |  |  |  |  |  |  |  |  |  |  |
| #3266 | Dharra et al. (2021) [135] | India | Nurses | Cross-sectional | NA | NA | 368 | 28.9, 3.7, NA | 58.6% (219) | NA | Under quarantine or just reported back to work after quarantine. | Convenience |
|  |  |  |  |  |  |  |  |  |  |  |  |  |
| #3238 | Cui et al. (2021) [136] | China | College students | Cross-sectional | May, 2020 | June, 2020 | 484 | 20.2, 1.3, 18-26 | 69.2% (335) | NA | NA | Convenience |
|  |  |  |  |  |  |  |  |  |  |  |  |  |
| #3171 | Graupensperger et al. (2021) [137] | United States | Young adults | Cross-sectional | April 6, 2020 | June 11, 2020 | 1,181 | 20.4, 1.8, 18-25 | 59.95% (708) | NA | NA | Random |
|  |  |  |  |  |  |  |  |  |  |  |  |  |
| #3059 | Chasson et al. (2021) [138] | Israel | Pregnant women | Cross-sectional | July 5, 2020 | October 7, 2020 | 1,127 | 28.2, 4.5, 19-44 | 100% (1127) | Aged >18 years, pregnant, and indicated that they could complete questionnaires in Hebrew or Arabic | NA | Convenience |
|  |  |  |  |  |  |  |  |  |  |  |  |  |
| #3051 | Gong et al. (2021) [139] | Iran | Healthcare workers | Cross-sectional | April 5, 2020 | April 20, 2020 | 280 | NA | 60.0% (168) | NA | NA | Snowball |
|  |  |  |  |  |  |  |  |  |  |  |  |  |
|  |  |  |  |  |  |  |  |  |  |  |  |  |

**Table 4 (Continued)**

*The Characteristics of the Studies Included in the Systematic Review*

| Study ID | First Author (Year) | Countries | Population | Study Design | Start Date | End date | Participants (N) | Age  (M, SD, Range) | Female (%, n) | Inclusion Criteria | Exclusion Criteria | Sampling Method |
| --- | --- | --- | --- | --- | --- | --- | --- | --- | --- | --- | --- | --- |
|  |  |  |  |  |  |  |  |  |  |  |  |  |
| #3039 | Cost et al. (2021) [140] | Canada | Children/ adolescents aged <=18 years | Cross-sectional | April 15 2020 | June 19 2020 | 1,013 | 10.5, 3.6, 2-18 | 44% (446) | Children/adolescents aged <=18 years | NA | Convenience |
|  |  |  |  |  |  |  |  |  |  |  |  |  |
| #3035 | Luo et al. (2021) [141] | China | Medical and nursing staff | Cross-sectional | February 18, 2020 | May 7, 2020 | 7,071 | NA | 71%, (5034) | NA | NA | Convenience |
|  |  |  |  |  |  |  |  |  |  |  |  |  |
| #3006 | Ettman et al. (2021) [142] | United States | Adults | Cohort | March, 2020 | April, 2021 | T1 = 1,441 T2 = 1,161 | NA. | T1: 51.9% (718)  T2: 51.8% (574) | From the National Health and Nutrition Examination Survey | Aged <18 years or  missing answers on  depressive symptoms questions | Convenience |
|  |  |  |  |  |  |  |  |  |  |  |  |  |
|  |  |  |  |  |  |  |  |  |  |  |  |  |
|  |  |  |  |  |  |  |  |  |  |  |  |  |
| #2944 | Hajek et al. (2021) [143] | Germany | Middle-aged/ Older adults (>= 40 years) | Cross-sectional | June 8, 2020 | July 22, 2020 | 3,134 | 67.6, 9.7, 46-98 | 49.4% (1548) | NA | NA | Convenience |
|  |  |  |  |  |  |  |  |  |  |  |  |  |
| #2849 | Han et al. (2021) [144] | China | Individuals living in mildly, moderately, and severely impacted provinces | Cohort | T1: 2016  T2: May 10, 2020 | T1: 2016  T2: May 20, 2020" | T1: 950  T2: 1,200 | T1:43.3, 13.6, NA  T2:31.2, 11.6, NA | T1: 54.8% (521)  T2: 64.1% (769) | 18-65 years; responding to all 20 items on the CES-D scale, and living in Hubei, Guangdong, Zhejiang, Sichuan, or Shanxi at the time when the survey was taken. | Participants who did not comply with data collection (e.g., invariance of response or non-compliance). | Cluster |
|  |  |  |  |  |  |  |  |  |  |  |  |  |
| #2837 | Heanoy et al. (2020) [68] | United States and Canada | General | Cross-sectional | March 24, 2020 | March 30, 2020 | 1,506 | 40.2, 15.8, NA | 76.5% (930) | Aged >18 years | NA | Snowball |
|  |  |  |  |  |  |  |  |  |  |  |  |  |
|  |  |  |  |  |  |  |  |  |  |  |  |  |

**Table 4 (Continued)**

*The Characteristics of the Studies Included in the Systematic Review*

| Study ID | First Author (Year) | Countries | Population | Study Design | | Start Date | End date | Participants (N) | | Age  (M, SD, Range) | Female (%, n) | Inclusion Criteria | | Exclusion Criteria | Sampling Method |
| --- | --- | --- | --- | --- | --- | --- | --- | --- | --- | --- | --- | --- | --- | --- | --- |
|  |  |  |  |  | |  |  |  | |  |  |  | |  |  |
| #2832 | Brunoni et al. (2021) [145] | Brazil | ELSA-Brazil cohort | Cohort | | T1: May 18, 2020  T2: July 20, 2020  T3: October 1, 2020 | T1: July 18, 2020  T2: September 20, 2020  T3: December 22, 2020 | T1: 2,117  T2: 1,799  T3: 1,571 | | 62.3, 8.4, NA | 58.2% (1232) | Enrolled at the Sao Paulo research center who completed the third wave and could answer online surveys. | | NA | Convenience |
|  |  |  |  |  | |  |  |  | |  |  |  | |  |  |
| #2821 | Ali et al. (2021) [146] | Bangladesh | Rehabilitation professionals | Cross-sectional | | July 18, 2020 | October 18 2020 | 420 | | NA | 37.9% (159) | Completely filled the form, lived in Bangladesh permanently, and did not leave the profession. | | NA | Convenience |
|  |  |  |  |  | |  |  |  | |  |  |  | |  |  |
| #2800 | De Pietri et al. (2021) [73] | Italy | Non-clinical, non-infected home-quarantined | Cross-sectional | | March 26, 2020 | April 9, 2020 | 660 | | 31.1, 10.3, 18-79 | 86.21% (569) | NA | | Did no answer honestly to all questions or reported being infected by Covid-19. | Convenience |
|  |  |  |  |  | |  |  |  | |  |  |  | |  |  |
| #2795 | Ren et al. (2021) [147] | China | College students | Cross-sectional | | September 14, 2020 | September 20, 2020 | 478 | | NA | 57.1% (273) | NA | | NA | Convenience |
|  |  |  |  |  | |  |  |  | |  |  | |  |  |  |
| #2792 | Hueniken et al. (2021) [57] | Canada | Adults | Repeated cross-sectional | T1: May 8, 2020  T2: May 29, 2020  T3: June 19, 2020  T4: July 10, 2020  T5: September 18, 2020  T6: November 27, 2020 | | T1: May 12, 2020  T2: June 1, 2020  T3: June 23, 2020  T4: July 14, 2020  T5: September 22, 2020  T6: December 1, 2020 | | T1: 1,005  T2: 1,002  T3: 1,005  T4: 1,003  T5: 1,003  T6: 1,003 | NA | T1: 49.6% (498)  T2: 49.6% (497)  T3: 49.7% (499)  T4: 49.1% (492)  T5: 49.7% (498)  T6: 50.1% (503) | | Canadians aged >= 18 years | NA | Quota |
|  |  |  |  |  |  | |  | |  |  |  |  | |  |  |

**Table 4 (Continued)**

*The Characteristics of the Studies Included in the Systematic Review*

| Study ID | First Author (Year) | Countries | Population | Study Design | Start Date | End date | Participants (N) | Age  (M, SD, Range) | Female (%, n) | Inclusion Criteria | Exclusion Criteria | Sampling Method |
| --- | --- | --- | --- | --- | --- | --- | --- | --- | --- | --- | --- | --- |
|  |  |  |  |  |  |  |  |  |  |  |  |  |
| #2783 | Karing et al. (2021) [148] | Germany | University students | Cross-sectional | April 7, 2020 | May 15, 2020 | 2,548 | 23.7, 4.6, NA | 74.8% (1906) | University student. | NA | Convenience |
|  |  |  |  |  |  |  |  |  |  |  |  |  |
| #2780 | Gouvernet et al. (2021) [149] | France | French-speaking | Cross-sectional | April 27, 2020 | May 11, 2020 | 1,753 | NA | 67.8% (1188) | Having partner (but not necessary living together during lockdown). | NA | Convenience |
|  |  |  |  |  |  |  |  |  |  |  |  |  |
| #2739 | Betini et al. (2021) [150] | Canada | Adults | Cohort | April 15, 2020 | July 28, 2020 | T1: 2,200  T2: 2,264  T3: 2,280  T4: 2,201 | NA | T1: 52% (1144)  T2: 52% (1177)  T3: 51% (1162)  T4: 51% (1122) | NA | NA | Quota |
|  |  |  |  |  |  |  |  |  |  |  |  |  |
| #2667 | Barcellos et al. (2021) [67] | United States | Senior population: 60-68 years. | Cohort | Nov, 2019 | Feb, 2020 | T1: 16,644  T2: 16,382 | T1: 64.3, 2.6, 60-68  T2: 63.8, 2.5, 60-68 | NA | Age between 60 and 68 years | NA | Convenience |
|  |  |  |  |  |  |  |  |  |  |  |  |  |
| #2625 | Elhadi et al. (2021) [151] | Libya | General | Cross-sectional | July 18, 2020 | August 23, 2020 | 10,296 | 28.9, 8.5, NA | 77.6% (7991) | Resided in Libya for at least 4 years and did not leave Libya during COVID-19. | NA | Convenience |
| #2593 | Ames-Guerrero et al. (2021) [152] | Peru | General | Cross-sectional | NA | NA | 434 | 33.87, NA, 18-68 | 61.3% (266) | Living in Peru, able to provide informed consent (>=18 years). | Aged <18 years; those whose responses were biased by acquiescence or social desirability. | Snowball |
|  |  |  |  |  |  |  |  |  |  |  |  |  |
| #2590 | Ali et al. (2021) [61] | Bangladesh | Healthcare workers in Dhaka | Cross-sectional | June 6, 2020 | July 6, 2020 | 294 | 28.9, 5.5, 19-50 | 43.5% (128) | HCWs working in a hospital in the Dhaka metropolitan area. | HCWs not working in any hospital settings. | Convenience |
|  |  |  |  |  |  |  |  |  |  |  |  |  |

**Table 4 (Continued)**

*The Characteristics of the Studies Included in the Systematic Review*

| Study ID | First Author (Year) | Countries | Population | Study Design | Start Date | End date | Participants (N) | | | Age  (M, SD, Range) | Female (%, n) | Inclusion Criteria | Exclusion Criteria | Sampling Method |
| --- | --- | --- | --- | --- | --- | --- | --- | --- | --- | --- | --- | --- | --- | --- |
|  |  |  |  |  |  |  |  | | |  |  |  |  |  |
| #2468 | Hu et al. (2021) [153] | China | Civil servants | Cross-sectional | April 9, 2020 | April 11, 2020 | 867 | | | NA | 54.2% (470) | Proficient in operating mobile phones to fill in the questionnaire and informed consent given. | Aged <18 years; not willing to participate or not familiar with cell phones. | Convenience |
|  |  |  |  |  |  |  |  | | |  |  |  |  |  |
| #2325 | Harjana et al. (2021) [154] | Indonesia | Repatriated migrant workers | Cross-sectional | June 4, 2020 | June 30, 2020 | 335 | | | NA | 19.40% (65) | Aged >=18 years; read and understand Bahasa Indonesia. | NA | Snowball |
|  |  |  |  |  |  |  |  | | |  |  |  |  |  |
| #2324 | Gong et al. (2021) [155] | China | Spinocerebellar ataxia (SCA) patients | Cross-sectional | February 8, 2020 | March 8, 2020 | SCA:307 Healthy control:319 | | | NA | SCA (52.4%, 161) Healthy control (52.0%, 166). | Genetic diagnosis of SCAs; signature on the informed consent form; no recent traumatic experiences or other stressful events. | NA | Cluster |
|  |  |  |  |  |  |  |  | | |  |  |  |  |  |
| #2289 | Bérard et al. (2021) [156] | France | General | Cross-sectional | April 17, 2020 | May 10, 2020 | 536 | | | Median: 67 years | 52% (257) | NA | NA | Random |
|  |  |  |  |  |  |  |  | | |  |  |  |  |  |
| #2131 | De France et al. (2022) [157] | Canada | Canadian adolescents in Kingston | Cohort | NA | NA | 136 | | | 16.2, 1.0, NA | 53.7% (73) | Previously taken part in a 2-year, four  wave, longitudinal study on mental health/well-being | NA | Convenience |
|  |  |  |  |  |  |  | |  | |  |  |  |  |  |
| #2088 | Frontera et al. (2021) [158] | United States | Adults | Cross-sectional | Feb 3, 2021 | Feb 5, 2021 | | 999 | | 45, NA, 18-84 | 51% (509) | NA | NA | Stratified |
|  |  |  |  |  |  |  | |  | |  |  |  |  |  |
| #2045 | Alpay et al. (2021) [159] | Turkey | Syrian refugees | Cross-sectional | August, 2020 | September, 2020 | | | 417 | 30.8, 10.6, 18-80 | 59.5% (248) | Aged >18 years; Syrian refugee status | NA | Purposive |
|  |  |  |  |  |  |  | | |  |  |  |  |  |  |
| #1676 | Hammarberg et al. (2020) [160] | Australia | Adults (>=18 years) | Cross-sectional | April 3, 2020 | May 2, 2020 | | | 13,829 | NA | 75.5% (10434) | Aged >=18 years and living in Australia with completed data. | No patient was involved | Convenience |
|  |  |  |  |  |  |  |  | | |  |  |  |  |  |

**Table 4 (Continued)**

*The Characteristics of the Studies Included in the Systematic Review*

| Study ID | First Author (Year) | Countries | | Population | Study Design | Start Date | End date | Participants (N) | | | Age  (M, SD, Range) | Female (%, n) | Inclusion Criteria | Exclusion Criteria | Sampling Method |
| --- | --- | --- | --- | --- | --- | --- | --- | --- | --- | --- | --- | --- | --- | --- | --- |
|  |  |  | |  |  |  |  |  | | |  |  |  |  |  |
| #1674 | Conti et al. (2020) [161] | Italy | Pediatric neuropsychiatric | | Cohort | April 20, 2020 | May 4, 2020 | 141 | | | NA | 19.9% (28) | Aged <18 years; presence of a neuropsychiatric disorder; availability of CBCL scores obtained before the lockdown between September 2019 and February 2020. | NA. | Criterion |
|  |  |  | |  |  |  |  |  | | |  |  |  |  |  |
| #1626 | Irfan et al. (2020) [69] | Malaysia | | University students | Cross-sectional | June, 2020 | July, 2020 | 958 | | | NA | 70.9% (679) | NA | NA | Convenience |
|  |  |  | |  |  |  |  |  | | |  |  |  |  |  |
| #1462 | Jewell et al. (2020) [162] | United States | | General | Cross-sectional | April 7, 2020 | June 1, 2020 | 1,083 | | | NA | 82.4% (884) | Aged >18 years; regardless of country of residence. | NA | Snowball |
|  |  |  | |  |  |  |  |  | | |  |  |  |  |  |
| #1447 | Dhar et al. (2020) [163] | Bangladesh | | University students | Cross-sectional | NA | NA | 15,543 | | | NA | 33.3% (5148) | University students | NA | Convenience |
|  |  |  | |  |  |  |  |  | | |  |  |  |  |  |
| #1389 | Ahmmed et al. (2020) [164] | Bangladesh | | Final year undergraduate students | Cross-sectional | July, 2020 | Aug, 2020 | 52 | | | NA | 40.38% (21) | NA | NA | Convenience |
|  |  |  | |  |  |  |  |  | | |  |  |  |  |  |
| #1387 | Figueroa-Quiñones et al. (2022) [165] | Peru | | University students | Cross-sectional | July, 2020 | Aug, 2020 | | 1,634 | | Me = 24, NA, 20-30. | 70.1% (1146) | Voluntarily participation; complete the survey | NA | Convenience |
|  |  |  | |  |  |  |  | |  | |  |  |  |  |  |
| #1359 | Islam et al. (2021) [166] | Bangladesh | | People infected with Covid-19 | Cross-sectional | Sep 11, 2020 | Oct 13, 2020 | | 1,002 | | 34.7, 13.9, 18-39 | 42.1% (422) | Aged >18 years; tested positive for COVID-19; willingness to complete the survey. | Incomplete responses; not infected with COVID-19; did not consent. | Snowball |
|  |  |  | |  |  |  |  | | |  |  |  |  |  |  |
| #1345 | Pensgaard et al. (2021) [167] | Norway | | Elite athletes | Cross-sectional | June, 2020 | September, 2020 | | | 378 | 26.86, 6.13, 18-59 | 42.1% (159) | Olympic-level and Paralympic-level athlete; members of The Norwegian Athlete Association (NISO). | NA | Convenience |
|  |  |  | |  |  |  |  |  | | |  |  |  |  |  |

**Table 4 (Continued)**

*The Characteristics of the Studies Included in the Systematic Review*

| Study ID | First Author (Year) | Countries | | Population | Study Design | Start Date | End date | Participants (N) | | | Age  (M, SD, Range) | Female (%, n) | Inclusion Criteria | Exclusion Criteria | Sampling Method |
| --- | --- | --- | --- | --- | --- | --- | --- | --- | --- | --- | --- | --- | --- | --- | --- |
|  |  |  | |  |  |  |  |  | | |  |  |  |  |  |
| #1339 | Al Mutair et al. (2021) [168] | Saudi Arabia | General | | Cross-sectional | NA | NA | 5,041 | | | 29.8, 8.6, 18-66 | 67.8% (3420) | Living in Saudi Arabia; aged >=18 years; no previous mental health diagnosis; can read and write Arabic. | NA. | Convenience |
|  |  |  | |  |  |  |  |  | | |  |  |  |  |  |
| #1284 | Cevher et al. (2021) [169] | Turkey | | Farmers | Cross-sectional | May, 2020 | May, 2020 | 2,125 | | | NA | 0% (0) | NA | NA | Random with multi-stage cluster |
|  |  |  | |  |  |  |  |  | | |  |  |  |  |  |
| #1251 | Chen et al. (2021) [60] | China | | Tuberculosis (TB) patients | Cross-sectional | Nov, 2020 | Mar, 2021 | 473 | | | 48.4, 17.6, N/A | 30.9% 77(146) | TB patients; aged >=18 years; without psychosis or communication problems; no difficulty in understanding the contents; comply with the study procedures and agree to participate | Patients who had completed treatment | Convenience |
|  |  |  | |  |  |  |  |  | | |  |  |  |  |  |
| #1226 | Zhang et al. (2021) [170] | Brazil | | General | Cross-sectional | May 9, 2020 | May 22, 2020 | 482 | | | 36.7, 13.5, N/A | 54.1% (261) | NA | NA | Quota |
|  |  |  | |  |  |  |  |  | | |  |  |  |  |  |
| #1080 | Ahmed et al. (2021) [171] | Egypt | | General and health care workers | Cross-sectional | May 1, 2020 | June 1, 2029 | 524 | | | NA | 57.4% (301) | Aged >18 years | Aged <18 years or live outside Egypt. | Snowball |
|  |  |  | |  |  |  |  |  | | |  |  |  |  |  |
| #1077 | Karaivazoglou et al. (2021) [172] | Greece | | General | Cross-sectional | April 10, 2020 | May 4, 2020 | 1,443 | | | NA | 72.9% (1052) | NA | NA | Convenience |
|  |  |  | |  |  |  |  | |  | |  |  |  |  |  |
| #1023 | Basheti et al. (2021) [173] | Jordan | | Healthcare students | Cross-sectional | July 14, 2020 | July 29, 2020 | | 450 | | 21.6, 4.9, NA | 67.1% (302) | Students studying a healthcare-oriented degree in either public or private universities. | NA | Convenience |
|  |  |  | |  |  |  |  | |  | |  |  |  |  |  |
| #993 | Flores et al. (2021) [174] | Mexico | | Government employees | Cross-sectional | June, 2020 | NA | | | 2,016 | NA | 49.8% (1003) | With institutional email; complete responses | With missing covariates; from Ministry of Health. | Convenience |
|  |  |  | |  |  |  |  | | |  |  |  |  |  |  |
|  |  |  | |  |  |  |  | | |  |  |  |  |  |  |
|  |  |  | |  |  |  |  |  | | |  |  |  |  |  |

**Table 4 (Continued)**

*The Characteristics of the Studies Included in the Systematic Review*

| Study ID | First Author (Year) | Countries | Population | | Study Design | Start Date | End date | Participants (N) | | Age  (M, SD, Range) | Female (%, n) | Inclusion Criteria | Exclusion Criteria | Sampling Method |
| --- | --- | --- | --- | --- | --- | --- | --- | --- | --- | --- | --- | --- | --- | --- |
|  |  |  |  | |  |  |  |  | |  |  |  |  |  |
| #989 | Abrams et al. (2021) [175] | United States | | Adults aged 55 and older | Cross-sectional | April 2, 2020 | May 31, 2020 | 6,264 | | NA | NA | Aged >= 55 | NA. | Non-probability + Snowball |
|  |  |  |  | |  |  |  |  | |  |  |  |  |  |
| #986 | Chen et al. (2021) [176] | United States | Low-income women with gynaecologic cancer | | Cross-sectional | March 15, 2020 | April 15, 2020 | 100 | | Me=60, NA, 19-86 | 100% (100) | Active Medicaid insurance; a diagnosis of gynaecologic precancer, cancer, or cancer-associated genetic mutation; and were treated by a gynaecologic oncologist and/or radiation oncologist at one of the participating hospitals between April 1, 2019 and April 1, 2020. | NA | Convenience |
|  |  |  |  | |  |  |  |  | |  |  |  |  |  |
| #909 | Hart et al., (2021) [177] | United States | Parents | | Cross-sectional | May 18, 2020 | May 28, 2020 | 965 | | NA | 56.23% (543) | Parents with at least one child aged <=17 years; residing in the US | NA | Convenience |
|  |  |  |  | |  |  |  |  | |  |  |  |  |  |
| #807 | Bahar Moni et al. (2021) [178] | Malaysia | Patients, university students, and healthcare professionals | | Cross-sectional | Aug, 2020 | Sep, 2020 | 720 | | 31.5, 11.5, 19-76 | 67.1% (483) | Aged >= 18 years and literate enough to respond to online questionnaire in English. | Participants who took <1 minute to complete the questionnaire. | Snowball |
|  |  |  |  | |  |  |  |  | |  |  |  |  |  |
| #725 | Cao et al. (2020) [179] | China | College students | | Cross-sectional | NA | NA | 7,143 | | NA | 69.65% (4975) | NA | NA | Cluster |
|  |  |  |  | |  |  |  |  | |  |  |  |  |  |
| #688 | Huang et al. (2020) [180] | China | Healthcare workers in Radiology Department | | Cross-sectional | Feb 7, 2020 | Feb 9, 2020 | 364 | | NA | 58.8% (214) | Aged >=18 years; nurses and technicians working in the radiology departments; informed of the study and willing to participate in the survey | Past substance abuse/dependence; history of mental illness; current brain lesion or serious physical disease | Convenience |
|  |  |  |  | |  |  |  | |  |  |  |  |  |  |

**Table 4 (Continued)**

*The Characteristics of the Studies Included in the Systematic Review*

| Study ID | First Author (Year) | Countries | Population | | Study Design | Start Date | End date | Participants (N) | | Age  (M, SD, Range) | Female (%, n) | Inclusion Criteria | Exclusion Criteria | Sampling Method |
| --- | --- | --- | --- | --- | --- | --- | --- | --- | --- | --- | --- | --- | --- | --- |
|  |  |  |  | |  |  |  |  | |  |  |  |  |  |
| #676 | Guo et al. (2020) [181] | China | | Patients with skin diseases. | Cross-sectional | April 15, 2020 | April 27, 2020 | 506 | | 33.5, 14.0, NA | 57.1% (289) | NA | NA. | Convenience |
|  |  |  |  | |  |  |  |  | |  |  |  |  |  |
| #661 | Effati-Daryani et al. (2020) [182] | Iran | Pregnant women | | Cross-sectional | March, 2020 | April, 2020 | 205 | | 29.3, 5.5, NA | 100% (205) | Having a file in the health centres of Tabriz; desire to participate and having a mobile phone and a healthy pregnancy. | History of mental illness, medical problems during pregnancy, and high-risk pregnancies. | Cluster |
|  |  |  |  | |  |  |  |  | |  |  |  |  |  |
| #633 | Every-Palmer et al. (2020) [183] | New Zealand | General | | Cross-sectional | April 15, 2020 | April 18, 2020 | 2,010 | | Me=45, NA, NA | 50.8% (1021) | New Zealanders aged between 18 and 90 years | NA | Convenience |
|  |  |  |  | |  |  |  |  | |  |  |  |  |  |
| #613 | Cortés-Álvarez et al. (2020) [184] | Mexico | General | | Cross-sectional | March 30, 2020 | April 5, 2020 | 1,105 | | NA | 62.1% (686) | NA | NA | Snowball |
|  |  |  |  | |  |  |  |  | |  |  |  |  |  |
| #601 | Fitzpatrick et al. (2020) [185] | United States | Adult (>=18 years) | | Cross-sectional | March 23, 2020 | March 30, 2020 | 10,368 | | NA | 51% (5288) | Ages >= 18 years | NA | Post-strata |
|  |  |  |  | |  |  |  |  | |  |  |  |  |  |
| #593 | Gur et al. (2020) [186] | United States | Black pregnant women | | Cross-sectional | April 17, 2020 | May 1, 2020 | 913 | | 32.5, 4.8, 18-46 | 100% (913) | Aged > 18 years; pregnant; completed questionnaire. | NA | Convenience |
|  |  |  |  | |  |  |  |  | |  |  |  |  |  |
| #581 | Fu et al. (2020) [187] | China | Adults | | Cross-sectional | Feb 18, 2020 | Feb 28, 2020 | 1,242 | | NA | 69.7% (866) | Aged >=18 years; living in Wuhan during COVID-19; informed consent electronically | Having baseline psychological or sleep-related diseases; having medications for mental or sleep illnesses; questionnaire with logical errors | Convenience |
|  |  |  |  | |  |  |  | |  |  |  |  |  |  |
| #548 | Akkaya-Kalayci et al. (2020) [188] | Austria and Turkey | Young people (15-25 years) | | Cross-sectional | May 22, 2020 | June 19, 2020 | | Austrians =621, Turkey = 387 | Austria: 20.2,1.6; Turkey: 21.1, 1.1 | Austria: 70.2% Turkey: 26.4% | Aged 15-25; living in their respective countries; sufficient language skills | Not living in Austria or Turkey, insufficient language skills; outside the range of 15-25 years | Convenience |
|  |  |  |  | |  |  |  | |  |  |  |  |  |  |
| #536 | Díaz-Jiménez et al. (2020) [59] | Spain | Social work students | | Cross-sectional | May 1, 2020 | May 24, 2020 | | 365 | 23.2, 6.2, 18-27 | 90.1% (329) | NA | NA | Convenience |
|  |  |  |  | |  |  |  | |  |  |  |  |  |  |

**Table 4 (Continued)**

*The Characteristics of the Studies Included in the Systematic Review*

| Study ID | First Author (Year) | Countries | Population | | Study Design | Start Date | End date | Participants (N) | | Age  (M, SD, Range) | Female (%, n) | Inclusion Criteria | Exclusion Criteria | Sampling Method |
| --- | --- | --- | --- | --- | --- | --- | --- | --- | --- | --- | --- | --- | --- | --- |
|  |  |  |  | |  |  |  |  | |  |  |  |  |  |
| #534 | Ben-Kimhy et al. (2020) [189] | Israel | | Fertility treatment patient | Cross-sectional | April 7, 2020 | April 18, 2020 | 168 | | 37.0, 6.2, 23-54. | 100% (168) | Valid email; received a treatment plan as of Jan, 2020, and had been postpone/discontinued due to COVID-19. | NA. | Convenience |
|  |  |  |  | |  |  |  |  | |  |  |  |  |  |
| #512 | Goularte et al. (2021) [190] | Brazil | General | | Cross-sectional | May 20, 2020 | July 14, 2020 | 1,996 | | 34.2, 12.6, NA | 84.5% (1676) | NA | NA | Convenience |
|  |  |  |  | |  |  |  |  | |  |  |  |  |  |
| #507 | Fu et al. (2021) [191] | China | College students | | Cross-sectional | May 10, 2020 | June 10, 2020 | 89,588 | | NA, NA, 18-30 | 56.3% (50394) | Be a college student | NA | Convenience |
|  |  |  |  | |  |  |  |  | |  |  |  |  |  |
| #503 | Ganson et al. (2021) [192] | United States | Young adults | | Cross-sectional | June 15, 2020 | June 30, 2020 | 4,852 | | 22.5, 0.7, 18-26 | 46.8% (2270) | NA | NA | Convenience |
|  |  |  |  | |  |  |  |  | |  |  |  |  |  |
| #501 | Gloster et al. (2020) [193] | International (78 countries) | General | | Cross-sectional | April 7, 2020 | June 7, 2020 | 9,565 | | 36.9, 13.3, NA | 77.70% (7432) | Aged >=18 years and ability to read one of the 18 languages. | NA | Convenience, random etc. |
|  |  |  |  | |  |  |  |  | |  |  |  |  |  |
| #499 | Donnelly et al. (2021) [194] | United States | General | | Cross-sectional | Late April, 2020 | July, 2020 | 582,440 for depression; 582,796 for anxiety. | | 44.4, 11.9, 18-65 | 61.76% | Aged < 65 years with no missing information. | NA | Cluster |
|  |  |  |  | |  |  |  |  | |  |  |  |  |  |
| #497 | Delmastro et al. (2020) [195] | Italy | Aged > 16 years | | Cross-sectional | June 4, 2020 | June 19, 2020 | 6,700 | | 50.5, 17.9, NA | 52.40% (3510) | Aged > 16 years | NA | Convenience |
|  |  |  |  | |  |  |  | |  |  |  |  |  |  |
| #477 | Fountoulakis et al. (2021) [196] | Greece | Adults aged 18-69 years | | Cross-sectional | April 11, 2020 | May 1, 2020 | | 3,399 | NA | 81.08% (2756) | NA | NA | Post-stratification |
|  |  |  |  | |  |  |  | |  |  |  |  |  |  |
| #462 | Simha et al. (2020) [197] | United Kingdom | General | | Cross-sectional | April 23, 2020 | April 28, 2020 | | 15,691 | 51.2, N/A, N/A | 58.4% (9163) | NA | NA | Probability |
|  |  |  |  | |  |  |  | |  |  |  |  |  |  |
| #407 | Antiporta et al. (2021) [198] | Peru | General | | Cross-sectional | May 4, 2020 | May 11, 2020 | | 57,446 | NA | 51.5% (38,060) | Provide consent and demographic; completed 90% of the survey | NA | Convenience |

**Table 4 (Continued)**

*The Characteristics of the Studies Included in the Systematic Review*

| Study ID | First Author (Year) | Countries | Population | | Study Design | Start Date | End date | Participants (N) | | Age  (M, SD, Range) | | Female (%, n) | Inclusion Criteria | Exclusion Criteria | Sampling Method |
| --- | --- | --- | --- | --- | --- | --- | --- | --- | --- | --- | --- | --- | --- | --- | --- |
|  |  |  |  | |  |  |  |  | |  | |  |  |  |  |
| #396 | Hou et al. (2021) [199] | Hong Kong | | General | Repeated cross-sectional | T1: February 25, 2020  T2: April 15, 2020 | T1: March 19, 2020  T2: May 1, 2020 | T1: 4,021  T2: 2,008 | | NA | | T1: 52.7% (2119)  T2: 52.7% (1058) | Hong Kong Chinese residents; aged >=15 years; Cantonese-speaking. | NA. | Random |
|  |  |  |  | |  |  |  |  | |  | |  |  |  |  |
| #381 | Fornili et al. (2021) [200] | Italy | Academics | | Cross-sectional | April, 2020 | May, 2020 | 18,120 | | NA | | 67.2% (11980) | NA | Psychological distress section not filled. | Convenience |
|  |  |  |  | |  |  |  |  | |  | |  |  |  |  |
| #365 | Das et al. (2021) [201] | Bangladesh | General | | Cross-sectional | April 15, 2020 | May 10, 2020 | 672 | | NA, NA, 15-65 | | 43.3% (291) | Bangladeshi ethnicity; living in Bangladesh; between 15-65 years; understood the questions | History of psychiatric disorders or addiction | Purposive |
|  |  |  |  | |  |  |  |  | |  | |  |  |  |  |
| #349 | Fukase et al. (2021) [202] | Japan | General | | Cross-sectional | July 17, 2020 | July 22, 2020 | 2,708 | | 49.2, 16.3, 20-69 | | 50% (1354) | Ages 20 to 69 years | NA | Quota |
|  |  |  |  | |  |  |  |  | |  | |  |  |  |  |
| #348 | Fanaj et al. (2021) [203] | Kosovo | General | | Cross-sectional | April 27, 2020 | June 5, 2020 | 155 | | 24.5, 9.2, 15-58 | | 75.5% (117) | NA | NA | Snowball |
|  |  |  |  | |  |  |  |  | |  | |  |  |  |  |
| #296 | Blix et al., (2021) [204] | Norway | General | | Cross-sectional | May 19, 2020 | May 26, 2020 | 1,041 | | 54.1, 15.9, NA | | 49% (510) | Access to Internet | NA | Probability |
|  |  |  |  | |  |  |  |  | |  | |  |  |  |  |
| #288 | Feter et al. (2021) [205] | Brazil | Adults | | Cohort | T1: Before Covid-19  T2: July 22, 2020 | T1: Unknown  T2: July 23, 2020 | 2,,314 | | NA | | 76.6% (1772) | NA | NA | Convenience |
|  |  |  |  | |  |  |  | |  |  | |  |  |  |  |
| #278 | Harling et al. (2021) [206] | South Africa | Rural South Africa | | Cohort | April 15, 2020 | December 24, 2020 | | 5,120 households | | NA | NA | Primary respondent must be the resident adult members of household (aged > 18 years) | NA | Cluster |
|  |  |  |  | |  |  |  | |  | |  |  |  |  |  |

**Table 4 (Continued)**

*The Characteristics of the Studies Included in the Systematic Review*

| Study ID | First Author (Year) | Countries | Population | Study Design | Start Date | End date | Participants (N) | | Age  (M, SD, Range) | | Female (%, n) | Inclusion Criteria | Exclusion Criteria | Sampling Method |
| --- | --- | --- | --- | --- | --- | --- | --- | --- | --- | --- | --- | --- | --- | --- |
|  |  |  |  |  |  |  |  | |  | |  |  |  |  |
| #259 | Batterham et al. (2021) [207] | Australia | Adult >= 18 years | Cohort | Late March, 2020 | Mid-June, 2020 | | T1: 1,296  T2: 969  T3: 952  T4: 910  T5: 874  T6: 820  T7: 762 | T1: 46.0, 17.3, NA | | T1: 50.1% (649) | NA | NA | Quota |
|  |  |  |  |  |  |  | |  |  | |  |  |  |  |
| #256 | Hao et al. (2021) [208] | China | Maintenance dialysis patients | Cross-sectional | March 20, 2020 | March 29, 2020 | | 321 | NA,NA,18-65 | | 41.43% (133) | Maintenance haemodialysis/peritoneal dialysis patients; informed consent and able to complete the survey. | History of mental and neurological diseases, long-term alcohol or drug abuse; patients with unclear awareness; cognitive impairment or serious audio-visual impairment; unable to adhere to the rules of the survey or answer the questionnaire. | Random |
|  |  |  |  |  |  |  | |  |  | |  |  |  |  |
| #239 | Haliwa et al. (2021) [209] | United States | Adults | Cohort | T0: December 4, 2019  T1: April 3, 2020  T2: May 14, 2020  T3: June 10, 2020 | T0: December 10, 2019  T1: April 15, 2020  T2: May 25, 2020  T3: June 19, 2020 | | T0: 912  T1: 300  T2: 146  T3: 142 | T1: 41.38, 12.37, 19-84  T2:: 43.75, 12.77, 19-76  T3: 40.46, 12.98, 18-75 | | T1: 58.7% (176)  T2: 53.4% (78)  T3: 50% (71) | NA | NA | Convenience |
|  |  |  |  |  |  |  | |  |  | |  |  |  |  |
|  |  |  |  |  |  |  | |  |  | |  |  |  |  |
|  |  |  |  |  |  |  |  | |  | |  |  |  |  |
| #224 | Cerecero-Garcia et al. (2021) [210] | Mexico | High-risk HIV | Cross-sectional | April 20, 2020 | April 27, 2020 | | 881 | NA | 2.9% Transgender women (17) | | Men who have sex with men (MSM) and transgender women (TGW). | NA | Convenience |
|  |  |  |  |  |  |  | |  |  | |  |  |  |  |

**Table 4 (Continued)**

*The Characteristics of the Studies Included in the Systematic Review*

| Study ID | First Author (Year) | Countries | Population | Study Design | Start Date | End date | Participants (N) | | Age  (M, SD, Range) | Female (%, n) | Inclusion Criteria | Exclusion Criteria | Sampling Method |
| --- | --- | --- | --- | --- | --- | --- | --- | --- | --- | --- | --- | --- | --- |
|  |  |  |  |  |  |  |  | |  |  |  |  |  |
| #221 | Creese et al. (2021) [211] | United Kingdom | Adults over 50 years of age | Cohort | May 13, 2020 | June, 8 2020 | | 3,281 | NA | 80% (2624) | Aged >=50 years; living in the UK; computer/Internet access; able to read and write English; no diagnosis of dementia. | NA | Convenience |
|  |  |  |  |  |  |  | |  |  |  |  |  |  |
| #204 | Alkhaldi et al. (2021) [212] | Saudi Arabia | Adults | Cross-sectional | April 22, 2022 | June 21, 2020 | | 2,393 | NA | 61.3% (1466) | Aged >=18 years; living in Saudi Arabia for at least a week. | NA | Convenience |
|  |  |  |  |  |  |  | |  |  |  |  |  |  |
| #203 | Hoyt et al. (2021) [213] | United States | College students | Cohort | T1: April 25, 2020  T2: July 5, 2020 | T1: April 30, 2020  T2: July 31, 2020 | | T1: 707  T2: 544 | T1: 20.0, 1.3, 18-22  T2: Unclear | T1: 61.0% (431)  T2: Unclear | Young adults; full-time college students aged 18-22 years, and completed the questionnaire. | NA | Convenience |
|  |  |  |  |  |  |  | |  |  |  |  |  |  |
| #180 | Beach et al. (2021) [214] | United States | Family caregivers (FC) and non-caregivers (NC) | Cross-sectional | April 15, 2020. | May 8, 2020. | | FC: 576  NC: 2,933  Ttl: 3,509. | NC: 58.4, 16.7, NA  FC: 59.0, 13.9. | NC: 68.2%, (2000) FC: 75.5%, (435). | NA | NA | Convenience |
|  |  |  |  |  |  |  | |  |  |  |  |  |  |
| #177 | Oh et al. (2021) [215] | United States | College students | Cross-sectional | Sep, 2020 | Dec, 2020 | | 36,875 | NA | NA | NA | NA | Random |
|  |  |  |  |  |  |  | |  |  |  |  |  |  |
| #172 | BC et al. (2021) [216] | Nepal | Quarantined returnee migrants | Mixed-Method | 21st April, 2020 | 15th May, 2020 | | 441 | NA | 3.4% (15) | NA | NA | Convenience |
|  |  |  |  |  |  |  | |  |  |  |  |  |  |
| #171 | Davis et al. (2021) [217] | Liberia | Medical and pharmacy students | Cross-sectional | July 1, 2020 | October 31, 2020 | | 113 | Me = 28 (IQR= 26.32) | 38.1% (43) | Aged >=18 years and those with active email accounts. | NA | Purposive |
|  |  |  |  |  |  |  | |  |  |  |  |  |  |

**Table 4 (Continued)**

*The Characteristics of the Studies Included in the Systematic Review*

| Study ID | First Author (Year) | Countries | Population | Study Design | Start Date | End date | Participants (N) | | Age  (M, SD, Range) | | Female (%, n) | Inclusion Criteria | Exclusion Criteria | Sampling Method |
| --- | --- | --- | --- | --- | --- | --- | --- | --- | --- | --- | --- | --- | --- | --- |
|  |  |  |  |  |  |  |  | |  | |  |  |  |  |
| #140 | Beutel et al. (2021) [218] | Germany | General | Repeated cross-sectional | T1: 2018  T2: May 2, 2020 | T1: 2018  T2: June 29, 2020 | | T1: 2,516  T2: 2,503 | NA | NA | | NA | NA | Quota |
|  |  |  |  |  |  |  | |  |  | |  |  |  |  |
| #139 | Kim et al. (2021) [219] | Korea | General | Cross-sectional | August 14, 2020 | August 31, 2020 | | 2,288 | 39.7, 11.7, 19-60 | | 51.8% (1186) | NA | NA | Quota |
|  |  |  |  |  |  |  | |  |  | |  |  |  |  |
| #137 | Dehkordi et al. (2021) [220] | Iran | Southwest Iran | Cross-sectional | NA | NA | | 2,919 | NA | | 64.33% (1877) | NA | NA | Cluster |
|  |  |  |  |  |  |  | |  |  | |  |  |  |  |
| #133 | Hu et al. (2021) [221] | Japan | Chinese residents in Japan | Cross-sectional | June 22, 2020 | July 14, 2020 | | 497 | 33.7, 6.2, NA | | 48.5% (241) | China origin; aged >=18 years; regardless of their immigration status or the duration of their stay in Japan; lived in Japan from December 2019 to July 14, 2020 | NA | Snowball |
|  |  |  |  |  |  |  | |  |  | |  |  |  |  |
| #126 | Mekhemar et al. (2021) [222] | Germany | Dental nurses | Cross-sectional | July, 2020 | Jan, 2021 | | 252 | NA | | 98% (247) | NA | NA | Convenience |
|  |  |  |  |  |  |  | |  |  | |  |  |  |  |
| #87 | Hoffart et al. (2021) [223] | Norway | Adults | Cross-sectional | March 31, 2020 | April 7, 2020 | | 10,061 | 36.0, 13.5, 18-86 | | 78% (7851) | Aged >= 18 years; living in Norway; experiencing social distancing protocols; informed consent. | NA | Convenience |
|  |  |  |  |  |  |  | |  |  | |  |  |  |  |
| #85 | Adnine et al. (2021) [224] | Morocco | Adults patients diagnosed and followed by rheumatologists for rheumatic disease | Cross-sectional | Early April, 2020 | Late May, 2020 | | 307 | 47, 14, N/A | | 65.8% (202) | NA | Age < 16 years; patients who were unable to respond or refused to participate in the study | Convenience |
|  |  |  |  |  |  |  | |  |  | |  |  |  |  |

**Table 4 (Continued)**

*The Characteristics of the Studies Included in the Systematic Review*

| Study ID | First Author (Year) | Countries | Population | Study Design | Start Date | End date | Participants (N) | | Age  (M, SD, Range) | | Female (%, n) | Inclusion Criteria | | Exclusion Criteria | Sampling Method |
| --- | --- | --- | --- | --- | --- | --- | --- | --- | --- | --- | --- | --- | --- | --- | --- |
|  |  |  |  |  |  |  |  | |  | |  |  | |  |  |
| #65 | Bryson et al. (2021) [225] | Australia | Mothers and children | Cross-sectional | May 6, 2020 | December 6, 2020 | Mothers=319 Children=319 | | | Mothers = Not stated  Children = 6.4, 0.3, 5.9-7.2 | M = 100% (319)  Child = 50.8%, (162) | | During recruitment between 2013 and 2014, pregnant women with expected due dates before 1/10/14, less than 37 weeks of gestation; sufficient English proficiency; home addresses within travel boundaries; self-reported two or more of 10 antenatal adversity risk factors (e.g., young pregnancy; low education etc.) | Enrolled in an existing Tasmanian nurse home visiting program, did not comprehend the recruitment invitation or give consent (e.g. had an intellectual disability); had insufficient English; no way to be contacted; experienced a critical event that excluded their participation (e.g., termination of pregnancy, stillbirth etc.) | Convenience |
|  |  |  |  |  |  |  |  | | |  |  |  | |  |  |
| #50 | Geren et al. (2021) [226] | Turkey | Pregnant women | Cross-sectional | September 1, 2020 | October 1, 2020 | | 322 | | 29.0, 5.6, NA | 100% (322) | NA | | Previously been diagnosed with a psychiatric disease or faced with any condition that could cause anxiety and depression during pregnancy (e.g., personal or family history of COVID-19 etc.). | Convenience |
|  |  |  |  |  |  |  | |  | |  |  |  | |  |  |
| #28 | Angwenyi et al. (2021) [75] | Kenya | Caregivers of young children and pregnant women in urban informal settlements in Kenya | Cross-sectional | September 23, 2020 | October 22, 2020 | | 845 | | 29, 7.2, N/A | 94.79% (801) | Having a child below 5 years (with or without disabilities); being currently pregnant; >18 years; a resident of these informal settlements. | | Decline to provide informed consent. | Non-probability and purposive |
|  |  |  |  |  |  |  | |  | |  |  |  | |  |  |
|  |  |  |  |  |  |  | |  | |  |  |  | |  |  |

**Table 4 (Continued)**

*The Characteristics of the Studies Included in the Systematic Review*

| Study ID | First Author (Year) | Countries | Population | Study Design | Start Date | End date | Participants (N) | | Age  (M, SD, Range) | | Female (%, n) | Inclusion Criteria | | Exclusion Criteria | | Sampling Method |
| --- | --- | --- | --- | --- | --- | --- | --- | --- | --- | --- | --- | --- | --- | --- | --- | --- |
|  |  |  |  |  |  |  |  | |  | |  | |  | |  |  |
| #14 | Guerrero et al. (2021) [227] | Czech Republic | Clients and staffs in residential institutions | Mixed-Method | April 5, 2020 | May 25, 2020 | | Clients=378 Staffs=492 Total=870. | | Client: 45.6, 27.5, NA  Staffs: 45.5, 10.7, NA | Client: 55.2% (207)  Staffs: 78.2% (385) | | Institutions that have had COVID-19 positive cases and those that have had no such cases. | | NA | Convenience |
|  |  |  |  |  |  |  | |  | |  |  |  | |  | |  |
| #7 | Fröhlich et al. (2021) [228] | Switzerland | Elite athletes | Cross-sectional | April 25, 2020 | May 25, 2020 | | 203 | | 24.0, 5.2, NA | 45.3% (92) | NA | | Incomplete baseline data; not participating in Olympic sports or in sports recognized by the International Olympic Committee (IOC); aged < 18 years, or registered outside the inclusion period between April 25, 2020 and May 25, 2020. | | Random |
|  |  |  |  |  |  |  | |  | |  |  |  | |  | |  |
| #4 | Coley et al. (2021) [229] | United States | People living in the US | Repeated cross-sectional | April 21, 2020 | November 23, 2020 | | 1,483,378 | | NA | 51.5% (763939) | NA | | NA | | Convenience |
|  |  |  |  |  |  |  | |  | |  |  |  | |  | |  |
| #243 | Law et al. (2021) [230] | United States | Youth with chronic pain conditions  or chronic headache | Cohort | Apr, 2020 | Jul, 2020 | | 250 families | | 17.21, 1.97, 12-21 | 71.2% (NA) | Families were recruited from existing study cohorts of youth with chronic headache (64.0%) or chronic pain (36.0%). | | NA | | Purposive |
|  |  |  |  |  |  |  | |  | |  |  |  | |  | |  |
| #227 | Sun et al. (2021) [231] | China | University students | Cross-sectional | March 20, 2020 | April 10, 2020 | | 1,912 | | 20.28, 2.10, 18-49 | 69.77% (1334) | Aged >= 18 years; currently enrolled in a Chinese college or university as an undergraduate or graduate student; fluent in the Chinese language | | NA | | Convenience |
|  |  |  |  |  |  |  | |  | |  |  |  | |  | |  |
| #217 | Heo et al. (2021) [232] | South Korea | General adult | Cross-sectional | September 21, 2020 | December 7, 2020 | | 322 | | NA | 75.& (243) | Adults aged >19 years living in South Korea | | NA | | Convenience |
|  |  |  |  |  |  |  | |  | |  |  |  | |  | |  |
|  |  |  |  |  |  |  | |  | |  |  |  | |  | |  |
|  |  |  |  |  |  |  | |  | |  |  |  | |  | |  |

**Table 4 (Continued)**

*The Characteristics of the Studies Included in the Systematic Review*

| Study ID | First Author (Year) | Countries | Population | Study Design | Start Date | End date | Participants (N) | | Age  (M, SD, Range) | | Female (%, n) | Inclusion Criteria | | Exclusion Criteria | | Sampling Method |
| --- | --- | --- | --- | --- | --- | --- | --- | --- | --- | --- | --- | --- | --- | --- | --- | --- |
|  |  |  |  |  |  |  |  | |  | |  | |  | |  |  |
| #214 | Hertz-Palmor et al. (2021) [233] | United States and Israel | General | Cohort | T1: April 6 2020  T2: May 12, 2020 | T1: May 5, 2020  T2: June 21, 2020 | | T1: 2,904/1,267  T2: 1,318/241 | | US: 41.97, 13.55, NA  Israel: 35.21, 12.26, NA | US: 77.8% ( 2259)  Israel: 54.2% (687) | | .NA | | NA | Convenience |
|  |  |  |  |  |  |  | |  | |  |  |  | |  | |  |
| #211 | Lindau et al. (2021) [234] | United States | U.S. women | Cross-sectional | April 10, 2020 | April 24, 2020 | | 3,200 | | NA. NA, 18-90 | 100% (3200) | English-speaking women aged >= 18 years | | NA | | Quota |
|  |  |  |  |  |  |  | |  | |  |  |  | |  | |  |
| #208 | Ruengorn et al. (2021) [235] | Thailand | General | Cross-sectional | April 21, 2020 | May 4, 2020 | | 2,303 | | 34.5, 10.2, NA | 60% (1384) | Being Thai citizens, permanent residents, or non-residents with work permits; aged > 18 years had full-time employment before the national lockdown owing to the COVID-19 out-break; can read and communicate in the Thai language; and can access the Internet | | Those who did not complete the online survey, or spent less than 2 min or more than 60 min on the survey | | Both convenience and snowball |
|  |  |  |  |  |  |  | |  | |  |  |  | |  | |  |
| #200 | Repon et al. (2021) [236] | Bangladesh | Healthcare professionals (HCP) | Cross-sectional | July 15, 2020 | September 20, 2020 | | 355 | | NA, NA, 20-60 | 43% (151) | HCPs working in Bangladesh who were willing to participate in this survey. | | Any previous history of psychiatric disorders, neurological disease, acute medical conditions, or the presence of any chronic diseases. | | Convenience |
|  |  |  |  |  |  |  | |  | |  |  |  | |  | |  |
| #195 | Owens et al. (2021) [237] | United States | Black and non-Black adult (aged >= 18) | Cohort | March, 2020 | November, 2020 | | 6,932 | | NA | 48% (2869) | Active members of the Understanding America Study (UAS) panel | | NA | | Convenience |
|  |  |  |  |  |  |  | |  | |  |  |  | |  | |  |
| #192 | Varma et al. (2021) [238] | 63 countries | General | Cross-sectional | April 9, 2020 | May 25, 2020 | | 1,745 | | 42.97, 14.46, 18-82 | 66.4% (1158) | Complete response to PSQI | | NA | | Convenience |
|  |  |  |  |  |  |  | |  | |  |  |  | |  | |  |
|  |  |  |  |  |  |  | |  | |  |  |  | |  | |  |

**Table 4 (Continued)**

*The Characteristics of the Studies Included in the Systematic Review*

| Study ID | First Author (Year) | Countries | Population | Study Design | Start Date | End date | Participants (N) | | Age  (M, SD, Range) | | Female (%, n) | Inclusion Criteria | | Exclusion Criteria | | Sampling Method |
| --- | --- | --- | --- | --- | --- | --- | --- | --- | --- | --- | --- | --- | --- | --- | --- | --- |
|  |  |  |  |  |  |  |  | |  | |  | |  | |  |  |
| #185 | Stampini et al. (2021) [239] | Italy | Pregnant women and new mothers | Cross-sectional | April 9, 2020 | May 3, 2020 | | 739 | | NA | 100% (739) | | NA | | Did not give consent (n = 8); not pregnant or did not deliver during the pandemic (n = 111); and Not living in Italy during the survey (n = 3) | Snowball |
|  |  |  |  |  |  |  | |  | |  |  |  | |  | |  |
| #164 | Esteban-Gonzalo et al. (2021) [240] | Spain | Pregnant women | Cross-sectional | June 1, 2020 | June 30, 2020 | | 353 | | 35.9, 7.0, NA | 100% (353) | Pregnant; aged >= 18 years; reside in Spain; able to fill out the Spanish questionnaire and to provide written informed consent | | NA | | Convenience |
|  |  |  |  |  |  |  | |  | |  |  |  | |  | |  |
| #156 | Mistry et al. (2021) [241] | Bangladesh | Older adults | Cross-sectional | October, 2020 | October, 2020 | | 1,032 | | NA | 34.5% (356) | Aged >= 60 years | | Adverse mental conditions (clinically proved schizophrenia, bipolar mood disorder, dementia/cognitive impairment); hearing disability; inability to communicate | | Stratified random |
|  |  |  |  |  |  |  | |  | |  |  |  | |  | |  |
| #152 | Schmits et al. (2021) [242] | Belgium | University students in the French-speaking regions of Belgium | Cross-sectional | February 22, 2021 | March 5, 2021 | | 23,307 | | 20.89, 1.96, 18-25 | 69.08%, (16100) | NA | | NA | | Convenience |
|  |  |  |  |  |  |  | |  | |  |  |  | |  | |  |
|  |  |  |  |  |  |  | |  | |  |  |  | |  | |  |

**Table 4 (Continued)**

*The Characteristics of the Studies Included in the Systematic Review*

| Study ID | First Author (Year) | Countries | Population | Study Design | Start Date | End date | Participants (N) | | Age  (M, SD, Range) | | Female (%, n) | Inclusion Criteria | | Exclusion Criteria | | Sampling Method |
| --- | --- | --- | --- | --- | --- | --- | --- | --- | --- | --- | --- | --- | --- | --- | --- | --- |
|  |  |  |  |  |  |  |  | |  | |  | |  | |  |  |
| #150 | Feurer et al. (2021) [243] | United States | Mother-child dyads either with a maternal history of MDD or without. | Cohort | June, 2020 | September, 2020 | | 45 mother-child dyads | | Youth: 12.42, 2.31; 9-16 Mothers: 42.09, 6.44; 30-56) | Youth: 82.2% (NA), Mothers: 100% (NA) | | Completed both baseline and COVID-19 assessments and at least one dyadic member had useable EEG data for the reward task; additional exclusionary criteria for youth included a lifetime history of MDD in the first study, and current MDD in the second study. | | Neurological disorders, traumatic brain injury, active suicidal ideation, lifetime history of bipolar disorder, schizophrenia, or psychosis, or current alcohol and/or substance use disorder in the past 6 months. | Convenience |
|  |  |  |  |  |  |  | |  | |  |  | |  | |  |  |
| #144 | Mana et al. (2021) [244] | Israel, Netherlands, Italy, and Spain | Healthy people over 18 years of age | Cross-sectional | March 19, 2020 | April 24, 2020 | | Israeli: 619, Dutch: 622, Italian: 924, Spanish: 489 | | Israeli: 38.61, 13.11, 18-75; Dutch: 44.71, 18.02, 19-88; Italians: 41.67, 16.84, 18-86; Spanish: 48.32, 13.86, 18-80 | Israeli: 51% (316), Dutch: 72% (445), Italian: 63.4% (586), Spanish: 76% (372) | NA | | NA | | Snowball |
|  |  |  |  |  |  |  | |  | |  |  |  | |  | |  |
| #130 | Marmet et al. (2021) [245] | Switzerland | Young Swiss men | Cohort | T1: April 2019  T2: May 13, 2020 | T1: February 14, 2020  T2: June 8, 2020 | | 2,345 | | 29.07, 1.28, NA | 0% (0) | Young Swiss men who were approximately 19 years old in the years 2010-2012 at three of the six national military recruitment centres (in Lausanne, Windisch and Mels), together covering 21 of Switzerland’s 26 cantons. | | Participants with missing values on predictor variables (about 3%) were excluded. | | Not clear |
|  |  |  |  |  |  |  | |  | |  |  |  | |  | |  |

**Table 4 (Continued)**

*The Characteristics of the Studies Included in the Systematic Review*

| Study ID | First Author (Year) | Countries | Population | Study Design | Start Date | End date | Participants (N) | | Age  (M, SD, Range) | | Female (%, n) | Inclusion Criteria | | Exclusion Criteria | | Sampling Method |
| --- | --- | --- | --- | --- | --- | --- | --- | --- | --- | --- | --- | --- | --- | --- | --- | --- |
|  |  |  |  |  |  |  |  | |  | |  | |  | |  |  |
| #101 | Wright et al. (2021) [246] | United Kingdom | Adults (aged 18+) | Cohort | April 1, 2020 | May 12, 2020 | | 41,909 | | NA | 2 rounds: 5,803 (77.21%)  3 rounds: 3,831 (76.07%)  4 rounds: 4,766 (72.46%)  5 rounds: 9,763 (72.8%)  6 rounds: 7,035 (75.09%) | | Participants with two or more waves of data during this period (n = 48723, observations = 208057, 71.6% of sample who joined the survey by 12 May) with complete data | | NA | Snowballing + targeted |
|  |  |  |  |  |  |  | |  | |  |  |  | |  | |  |
| #97 | Kobayashi et al. (2021) [247] | Japan | Workers | Cross-sectional | October 1, 2020 | November 23, 2020 | | 3,464 | | NA | 24.5% (847) | Workers in Fukushima Prefecture who belong to the Fukushima Branch of the Japanese Trade Union Confederation and related organizations | | NA | | Convenience |
|  |  |  |  |  |  |  | |  | |  |  |  | |  | |  |
| #93 | Oginni et al. (2021) [248] | Nigeria | Adults | Cross-sectional | NA | NA | | 966 | | Me=29.0., IQR=12.0 | 49.6% (479) | Aged >= 18 years; resident in Nigeria for at least six months prior to the lockdown, fluent in English, able to use the internet and reported no severe cognitive or physical impairments | | NA | | Convenience |
|  |  |  |  |  |  |  | |  | |  |  |  | |  | |  |
| #90 | Maffly-Kipp et al. (2021) [249] | Canada | General | Cross-sectional | March, 2020 | June, 2020 | | 11,227 | | 35.36, 13.27, 18-85 | 69.9% (NA) | Adult aged >= 18 years | | NA | | Random |
|  |  |  |  |  |  |  | |  | |  |  |  | |  | |  |
| #89 | Shuster et al. (2021) [250] | United States | Adults | Cohort | April 2, 2020 | June 4, 2020 | | 1,456 | | 35.04, 13.08, 18-64 | 49.18% (716) | Aged between 18-64 years; current US resident | | NA | | Convenience |

**Table 4 (Continued)**

*The Characteristics of the Studies Included in the Systematic Review*

| Study ID | First Author (Year) | Countries | Population | Study Design | Start Date | End date | Participants (N) | | Age  (M, SD, Range) | | Female (%, n) | Inclusion Criteria | | Exclusion Criteria | | Sampling Method |
| --- | --- | --- | --- | --- | --- | --- | --- | --- | --- | --- | --- | --- | --- | --- | --- | --- |
|  |  |  |  |  |  |  |  | |  | |  | |  | |  |  |
| #80 | Ogrodniczuk et al. (2021) [251] | Canada | Help-seeking adult men | Cross-sectional | April 1, 2020 | May 30, 2020 | | 434 | | 39.76, 14.04, 18-80 | 0% (0) | | Aged >= 18 year; having online access; being able to read and understand English; self-identifying as male; residing in Canada, and were visiting an eHealth depression resource (HeadsUpGuys.org) | | NA | Convenience |
|  |  |  |  |  |  |  | |  | |  |  |  | |  | |  |
| #78 | Moya et al. (2021) [252] | Colombia | Caregivers | Cohort | T1: March, 2018  T2: July 2018  T3: March, 2019  T4: July, 2019 | NA | | T1 and T2: 573  T3 and T4: 803 | | T1 and T2: 29.09, 9.30, NA  T3 and T4: 29.05, 9.24), NA | T1 and T2: 93.9% (538)  T3 and T4: 97.3% (781) | Caregivers who took part in a cluster-randomised trial of Semillas de Apego, a psychosocial group programme based on the Child-Parent Psychotherapy | | NA | | Cluster |
|  |  |  |  |  |  |  | |  | |  |  |  | |  | |  |
| #76 | Kjeldsted et al. (2021) [253] | Denmark | Cancer patients | Cross-sectional | June 24, 2020 | July 17, 2020 | | 1,160 | | Me = 68, NA, 29-91 | 71% (822) | All patients with breast, lung, gastrointestinal, urological, and gynaecological cancers who had at least one doctor’s appointment in the outpatient clinics between 15 March and 30 April 2020 identified from the hospital’s electronic outpatient lists who had a validated ‘e-Boks' | | NA | | Convenience |
|  |  |  |  |  |  |  | |  | |  |  |  | |  | |  |
| #57 | Ochnik et al. (2021) [254] | 9 countries | University student | Cross-sectional | May, 2020 | July, 2020 | | 2,349 | | NA | 69.3% (1627) | NA | | Respondents who did not want to reveal their gender (n = 6) | | Convenience |
|  |  |  |  |  |  |  | |  | |  |  |  | |  | |  |
|  |  |  |  |  |  |  | |  | |  |  |  | |  | |  |

**Table 4 (Continued)**

*The Characteristics of the Studies Included in the Systematic Review*

| Study ID | First Author (Year) | Countries | Population | Study Design | Start Date | End date | Participants (N) | | Age  (M, SD, Range) | | Female (%, n) | Inclusion Criteria | | Exclusion Criteria | | Sampling Method |
| --- | --- | --- | --- | --- | --- | --- | --- | --- | --- | --- | --- | --- | --- | --- | --- | --- |
|  |  |  |  |  |  |  |  | |  | |  | |  | |  |  |
| #56 | Robertson et. al (2021) [255] | United States | Adults | Cohort | March 28, 2020 | April, 2021 | | 6740 | | Me = 37, NA, NA | 3526 (52.3%) | | Reside in the USA or a US territory; aged >= 18 years; provide valid email address and demonstrate early engagement in longitudinal study activities, including:  (a) completion of V1 (which provided the opportunity to consent for serologic testing) and  (b) completion of at least one additional screening visit in addition to V1 (ie, V0 or V2) or provision of a baseline specimen for serologic testing (S1) | | NA | Combination of snowball and convenience sampling |
|  |  |  |  |  |  |  | |  | |  |  |  | |  | |  |
| #47 | Senturk et. al (2021) [256] | Turkey | Remote workers | Cross-sectional | October 25, 2020 | December 24, 2020 | | 459 | | 35.64, 6.84, 24-60 | 44.7% (205) | No remote working experience prior to the COVID-19 pandemic; WFH for at least six months after the COVID-19 pandemic declaration; WFH at the time of the questionnaire; graduation from the university | | NA | | Snowball |
|  |  |  |  |  |  |  | |  | |  |  |  | |  | |  |
| #44 | Sams et al. (2021) [257] | United States | Dwelling older adults | Cross-sectional | June 16, 2020 | June 25, 2020 | | 501 | | NA | 66.3% (324) | NA | | Under 60 years old; non-English speaking; lived outside of the US | | Convenience |
|  |  |  |  |  |  |  | |  | |  |  |  | |  | |  |
|  |  |  |  |  |  |  | |  | |  |  |  | |  | |  |
|  |  |  |  |  |  |  | |  | |  |  |  | |  | |  |
|  |  |  |  |  |  |  | |  | |  |  |  | |  | |  |

**Table 4 (Continued)**

*The Characteristics of the Studies Included in the Systematic Review*

| Study ID | First Author (Year) | Countries | Population | Study Design | Start Date | End date | Participants (N) | | Age  (M, SD, Range) | | Female (%, n) | Inclusion Criteria | | Exclusion Criteria | | Sampling Method |
| --- | --- | --- | --- | --- | --- | --- | --- | --- | --- | --- | --- | --- | --- | --- | --- | --- |
|  |  |  |  |  |  |  |  | |  | |  | |  | |  |  |
| #35 | Sirin et al. (2021) [258] | Turkey | Senior citizens living in the central district of Ankara | Mixed-Method | June 1, 2020 | June 10, 2020 | | 278 | | 72.64, 6.323, NA | 56.1% (156) | | Aged >= 65 years; registered with family physicians living in the central districts of Ankara | | NA | Random |
|  |  |  |  |  |  |  | |  | |  |  |  | |  | |  |
| #26 | Lueck et al. (2021) [259] | United States | General | Cross-sectional | NA | June, 2020 | | 5,010 | | 44.53, 16.92, NA | 50.3% (2520) | NA | | NA | | Quota |
|  |  |  |  |  |  |  | |  | |  |  |  | |  | |  |
| #24 | Ribeiro et al. (2021) [260] | United Kingdom | Luxembourgish residents | Cohort | T1: April 15, 2020  T2: May 5, 2020 | T1: May 6, 2020  T2: May 20, 2020 | | 1,756 | | 48.27, 15.00, 18-64 | 50.74% (891) | NA | | NA | | Stratified |
|  |  |  |  |  |  |  | |  | |  |  |  | |  | |  |
| #20 | Tasnim et al. (2021) [261] | Bangladesh | Individuals with medical conditions | Cross-sectional | November, 2020 | January, 2021 | | 971 | | 42.29, 15.86, 18-80 | 49.9%, (485) | Being a Bangladeshi resident; having at least one pre-existing medical condition; having internet access; being willing to take part in the survey | | Having incomplete surveys | | Convenience |
|  |  |  |  |  |  |  | |  | |  |  |  | |  | |  |
| #5 | Zhang et al. (2021) [262] | United States | Adult residents of Hawaii | Repeated Cross-sectional | April 23, 2020 | November 23, 2020 | | 11,194 | | NA, NA, 18-88 | 51.0% (6440) | Residing in Hawaii; aged >= 18 years | | NA | | Convenience |
|  |  |  |  |  |  |  | |  | |  |  |  | |  | |  |
| #437 | Rudenstine et al. (2021) [263] | United States | University students | Cross-sectional | April 8, 2020 | May 2, 2020 | | 1,821 | | 26.2, NA, 18-77 | 71.6% (1301) | Aged 18 years or older; currently enrolled in at least one course across six CUNY campuses | | NA | | Convenience |
| #436 | Zheng et al. (2021) [264] | United States, Canada | North Americans | Cross-sectional | March 18, 2020 | May 29, 2020 | | 2,463 | | 44.9, 15.3, 14-91 | 83.6% (2059) | Resident of Canada or the US | | NA | | Convenience |
|  |  |  |  |  |  |  | |  | |  |  |  | |  | |  |
| #425 | Shalhub et al. (2021) [265] | 58 countries | Vascular surgeons | Cross-sectional | April 14, 2020 | April 24, 2020 | | 1,609 | | NA | 28.6% (461) | NA | | NA | | Convenience |
|  |  |  |  |  |  |  | |  | |  |  |  | |  | |  |
| #418 | Vujčić et al. (2021) [266] | Serbia | General | Cross-sectional | March 23, 2020 | April 25, 2020 | | 1,057 | | 36.1, 15.0, 18-88 | 67.7% (716) | Aged 18 years or older; residents of the Republic of Serbia | | NA | | Snowball |

**Table 4 (Continued)**

*The Characteristics of the Studies Included in the Systematic Review*

| Study ID | First Author (Year) | Countries | Population | Study Design | Start Date | End date | Participants (N) | Age  (M, SD, Range) | Female (%, n) | Inclusion Criteria | Exclusion Criteria | Sampling Method |
| --- | --- | --- | --- | --- | --- | --- | --- | --- | --- | --- | --- | --- |
|  |  |  |  |  |  |  |  |  |  |  |  |  |
| #417 | Liang et al. (2021) [267] | China | Older Adults | Cross-sectional | June 15, 2020 | July 10, 2020 | 516 | 67.6, 6.6, NA | 57.9% (299) | Never been infected with COVID-19; no cognitive impairments; have access to mobile phone or laptop; Can read and listen Mandarin | NA | Snowball |
|  |  |  |  |  |  |  |  |  |  |  |  |  |
| #390 | Nagasu et al. (2021) [268] | Japan | General | Cross-sectional | March 26, 2020 | March 28, 2020 | 11,342 | NA, NA, 20-64 | 49.4% (5,608) | Registered with MACROMILL INC | NA | Quota |
| #389 | Vicens et al. (2021) [269] | Spain | General | Cross-sectional | April 22, 2020 | May 14, 2020 | 1,607 | 39.4, 13.6, NA | 74.4% (1,195) | Aged 18 years or older | NA | Snowball |
| #380 | Posel et al. (2021) [270] | South Africa | Adults | Cohort | T1: May 7, 2020  T2: July 13, 2020 | T1: July 13, 2020  T2: August 13, 2020 | 2,213 | 39.2, 11.5, 18-89 | 57.7% (1,277) | Adults who were employed before the pandemic | NA | Stratified |
| #376 | Romeo et al. (2021) [271] | Italy | University students and general workers | Cross-sectional | March 19, 2020 | April 5, 2020 | 956 | Students: 23.4, 2.7, NA  Workers: 33.3, 6.9, NA | Students: 77.4% (370)  Workers: 67.8% (324) | NA | NA | Snowball |
| #372 | Thombs et al. (2020) [272] | 4 countries | People with systemic sclerosis | Cohort | T1: July 1, 2019  T2: April 9, 2020 | T1: December 31, 2019  T2: April 27, 2020 | 435 | 56.9, 12.6, NA | 88.5% (385) | Aged 18 years or older; met criteria for systemic sclerosis | NA | Convenience |
| #370 | Wong et al. (2021) [273] | Malaysia | Adults | Cross-sectional | May 12, 2020 | September 5, 2020 | 1,163 | 35.2, 11.9, 18-84 | 81.3% (946) | Aged 18 years or older; Malaysian | Have chronic medical condition; pregnant or breastfeeding; never been infected with COVID-19 | Convenience |
| #360 | Meng et al. (2021) [274] | China | College students | Cross-sectional | February 14, 2020 | February 21, 2020 | 3,351 | 21.2, 2.6, NA | 60.6% (2,002) | Full-time college students | NA | Snowball |

**Table 4 (Continued)**

*The Characteristics of the Studies Included in the Systematic Review*

| Study ID | First Author (Year) | Countries | Population | Study Design | Start Date | End date | Participants (N) | Age  (M, SD, Range) | Female (%, n) | Inclusion Criteria | Exclusion Criteria | Sampling Method |
| --- | --- | --- | --- | --- | --- | --- | --- | --- | --- | --- | --- | --- |
|  |  |  |  |  |  |  |  |  |  |  |  |  |
| #354 | Porter et al. (2021) [275] | 4 countries | Adolescents and young adults | Cohort | August, 2020 | October, 2020 | 8,988 | Adolescents: NA, NA, 18-19  Young adults: NA, NA, 25-26 | 49% (4,378) | Born in the year 1994, 1995, 2001, or 2002; Living in Ethiopia, India, Peru, or Vietnam | NA | NA |
|  |  |  |  |  |  |  |  |  |  |  |  |  |
| #347 | Knolle et al. (2021) [276] | Germany, United Kingdom | General | Cross-sectional | April 27, 2020 | May 31, 2020 | 780 | Germany: 45.4, 14.8, NA  UK: 39.0, 16.0, NA | Germany: 71.2% (385)  UK: 73.6% (176) | NA | NA | Snowball |
| #340 | Jones et al. (2021) [277] | United States | College students | Cross-sectional | April 14, 2020 | April 22, 2020 | 2,282 | NA, NA, 18-25 | 57.9% (1,523) | NA | NA | Simple random selection |
| #336 | Wickens et al. (2021) [278] | Canada | English-speaking adults | Cross-sectional | May 8, 2020 | May 12, 2020 | 1,005 | NA | 49.7% (498) | Members of the Asking Canadians web panel; Canadian; English-speaking | NA | Convenience |
| #334 | Racine et al. (2021) [279] | Canada | Mothers | Cohort | T1: April, 2012  T2: June, 2014 | T1: October, 2014  T2: October, 2016 | 1,301 | NA | 100% (1,301) | Female; participated in the All Our Families pregnancy cohort | NA | Convenience |
| #323 | Mikocka-Walus et al. (2021) [280] | Australia | Parents | Cross-sectional | April, 2020 | NA | 2,110 | 38.3, 7.1, 19-69 | 80.6% (1,701) | Aged 18 years or older; Australian; English-speaking; parent of a child aged 0-18 years | NA | Convenience |
| #318 | Thayer et al. (2021) [281] | United States | Pregnant women | Cross-sectional | April 16, 2020 | April 30, 2020 | 2,099 | 31.3, 4.4, NA | 100% (2,099) | NA | NA | Convenience |
| #313 | Landa-Blanco et al. (2021) [282] | 5 countries | Adults | Cross-sectional | March 29, 2020 | April 17, 2020 | 1,559 | 33.5, 11.4, NA | 71.4% (1,113) | Living in Honduras, Chile, Costa Rica, Mexico, or Spain | NA | Non-probabilistic sampling |

**Table 4 (Continued)**

*The Characteristics of the Studies Included in the Systematic Review*

| Study ID | First Author (Year) | Countries | Population | Study Design | Start Date | End date | Participants (N) | Age  (M, SD, Range) | Female (%, n) | Inclusion Criteria | Exclusion Criteria | Sampling Method |
| --- | --- | --- | --- | --- | --- | --- | --- | --- | --- | --- | --- | --- |
|  |  |  |  |  |  |  |  |  |  |  |  |  |
| #247 | van Rüth et al. (2021) [283] | Germany | Homeless people | Cross-sectional | May 25, 2020 | June 3, 2020 | 111 | 44.3, 12.6, 12-86 | 20% (22) | NA | NA | Cluster |
|  |  |  |  |  |  |  |  |  |  |  |  |  |
| #246 | Koyucu et al. (2021) [284] | Turkey | Pregnant women | Cross-sectional | June 5, 2020 | June 12, 2020 | 989 | 30.4, 4.3, 18-45 | 100% (989) | Women aged between 18 and 45 years; pregnant | Have a history of psychiatric disorder | Convenience |
|  |  |  |  |  |  |  |  |  |  |  |  |  |
| #579 | Ettman et al. (2020) [285] | United States | General | Cross-sectional | March 31, 2020 | April 13, 2020 | 1441 | NA, NA, 18-39 | 51.9% (718) | Adults aged 18 years or older who could speak English and who had completed an AmeriSpeak survey  in the past 6 months | NA | Probability |
| #572 | Kämpfen et al. (2020) [286] | United States | General | Cross-sectional | March 10, 2020 | March 31, 2021 | 6585 | 48.43, 16.58, NA | NA | Respondents who completed the survey on the same day they started it and for which they had non-missing information about their mental health characteristics | NA | Random |
| #556 | Jacques-Aviñó et al. (2020) [287] | Spain | General | Cross-sectional | April 8, 2020 | May 28, 2020 | 7053 | 44.8, 13.8, 18 and above | 71.1% (5014) | To be 18 years or older and live in Spain during lockdown. | NA | Convenience and snowball |
|  |  |  |  |  |  |  |  |  |  |  |  |  |
| #554 | Li et al. (2020) [288] | Hong Kong | General | Cross-sectional | February 25, 2020 | April 29, 2020 | 3011 | NA,NA,NA | 54.9% (1652) | Hong Kong Chinese; 15 years of age or older; Cantonese-speaking | NA | Probability |
|  |  |  |  |  |  |  |  |  |  |  |  |  |
|  |  |  |  |  |  |  |  |  |  |  |  |  |
| #553 | Xiao et al. (2020) [289] | China | Online networkers | Cross-sectional | August 27, 2020 | August 30, 2020 | 2015 | NA, NA, NA | 50.72% (1022) | NA | NA | Convenience |

**Table 4 (Continued)**

*The Characteristics of the Studies Included in the Systematic Review*

| Study ID | First Author (Year) | Countries | Population | Study Design | Start Date | End date | Participants (N) | Age  (M, SD, Range) | Female (%, n) | Inclusion Criteria | Exclusion Criteria | Sampling Method |
| --- | --- | --- | --- | --- | --- | --- | --- | --- | --- | --- | --- | --- |
|  |  |  |  |  |  |  |  |  |  |  |  |  |
| #546 | Ueda et al. (2020) [290] | Japan | General | Repeated cross-sectional | T1: April 16, 2020  T2: May 15, 2020 | T1: April 18, 2020  T2: May 17, 2020 | 2000 | NA, NA, NA | 50.4% (1008) | NA | NA | Stratified |
|  |  |  |  |  |  |  |  |  |  |  |  |  |
| #539 | Garre-Olmo et al (2021) [291] | Spain | General | Cross-sectional | April 8, 2020 | May 4, 2020 | 692 | 50.2, 16.3, 18.9-90.9 | 54.8% (379) | 18 years and over, signing the informed consent | Subjects with terminal illness, cognitive impairment or dementia, intellectual disability, or institutionalized were not included. | Stratified random |
| #532 | Polsky et al. (2020) [292] | Canada | General | Cross-sectional | May 4, 2020 | May 10, 2020 | 4481 | NA, NA, NA | 50.7% (504) | Canadian population aged 15 or older | Respondents with missing data on household food security status | Convenience |
| #529 | Silverman et al. (2020) [293] | United States | Postpartum patients | Cross-sectional | June 2, 2020 | June 30, 2020 | 516 | Ambulatory Practices: 27, NA, 16-40  Faculty Practice: 33, NA, 19-48 | 100% (516) | NA | NA | Cluster |
| #505 | Lin et al. (2020) [294] | China | General | Cross-sectional | January 24, 2020 | February 24, 2020 | 2446 | 18-24 years old, N=1,674, 68%  25-39 years old, N=537, 22%  40-68 years old, N=235, 9.6% | 70.0% (1,713) | The respondents were mainland China residents who were between 18 and 70 years of age. | NA | Convenience |

**Table 4 (Continued)**

*The Characteristics of the Studies Included in the Systematic Review*

| Study ID | First Author (Year) | Countries | Population | Study Design | Start Date | End date | Participants (N) | Age  (M, SD, Range) | Female (%, n) | Inclusion Criteria | Exclusion Criteria | Sampling Method |
| --- | --- | --- | --- | --- | --- | --- | --- | --- | --- | --- | --- | --- |
|  |  |  |  |  |  |  |  |  |  |  |  |  |
| #498 | Sinawi et al. (2021) [295] | Oman | General | Cross-sectional | March 20, 2020 | April 20, 2020 | 1538 | NA, NA, NA | 74.8%, (1151) | Omani citizens and residents; 18 years and above; had access to social media platforms or institutional emailing systems; could provide an electronically signed informed consent form | Had diagnosis of moderate to severe intellectual disability; did not sign the consent; provided incomprehensive or incomplete questionnaires; did not meet the inclusion criteria | Convenience |
|  |  |  |  |  |  |  |  |  |  |  |  |  |
| #492 | Kar et al. (2021) [296] | UK | General | Cross-sectional | March 29, 2020 | April 7, 2020 | 733 | 37.2, 13.7, 18-76 | 41.5% (304) | NA | NA | Convenience |
| #491 | Serafini et al. (2021) [297] | United States | Patients of mental health clinic | Cross-sectional | March, 2020 | May, 2020 | 35 | NA, NA, 20-79 | 74%, (26) | Being a patient in the EHHOP Mental Health Clinic | NA | Convenience |
| #478 | Yörük et al. (2021) [298] | Turkey | Nurses and midwives | Cross-sectional | May 30, 2020 | June 13, 2020 | 377 | 32.20, 8.11, 20-54 | NA, NA | NA | NA | Convenience |
| #460 | Reagu et al. (2021) [299] | Qatar | Individuals in institutional quarantine and isolation | Cross-sectional | June 1, 2020 | July 31, 2020 | 748 | 38.6, 12.1, 18-94 | 48.1%, (360) | Consenting adults aged above 18 years; in quarantine or isolation due to the COVID-19 pandemic | Inability to consent for participation in the study due to underlying mental health conditions or severe physical health symptoms due to COVID-19 infection; inability to engage with the written questionnaires due to illiteracy, learning difficulty, or other | Convenience |
|  |  |  |  |  |  |  |  |  |  |  |  |  |
| #459 | Khademian et al. (2021) [300] | Iran | General | Cross-sectional | April 18, 2020 | April 28, 2020 | 1498 | NA | 77.3% (1158) | NA | NA | Snowball |

**Table 4 (Continued)**

*The Characteristics of the Studies Included in the Systematic Review*

| Study ID | First Author (Year) | Countries | Population | Study Design | Start Date | End date | Participants (N) | Age  (M, SD, Range) | Female (%, n) | Inclusion Criteria | Exclusion Criteria | Sampling Method |
| --- | --- | --- | --- | --- | --- | --- | --- | --- | --- | --- | --- | --- |
|  |  |  |  |  |  |  |  |  |  |  |  |  |
| #451 | Reading Turchioe et al. (2021) [301] | United States | General | Cross-sectional | March 24, 2020 | April 4, 2020 | 963 | 45, 15.8, 18-83 | 50.7% (488) | NA | NA | Random |
| #446 | Saito et al. (2021) [302] | Japan | Employees | Cohort | T1: March 26, 2020  T2: June 26,2020 | T1:April 6, 2020  T2: July 2, 2020 | Permanent employees: 1373  Non-permanent employees: 685 | Permanent employees: 40.78, 10.54, 15-59  Non-permanent employees: 40.22, 11.64, 15-59 | Perm.: 40.3%, (553)  Non-perm.: 70.5%, (483) | Workers employed by general companies. | a) Civil servants; b) Self-employed individuals. | Convenience |
| #445 | Torrente et al. (2021) [303] | Argentina | General | Cross-sectional | March 24, 2020 | March 25, 2020 | 10053 | 41.55, 11.52, 18-84 | 83.4% (8384) | > 18 years | NA | Convenience |
| #443 | Wong et al. (2021) [304] | Malaysia | General | Repeated cross-sectional | T1: 25th Jan, 2021  T2: 22nd Feb, 2021  T3: 18th Mar, 2021 | T1: 21st Feb, 2021  T2: 17th Mar, 2021  T3: 3rd Apr, 2021 | 962 | 35.5, 11.2, NA | 68.6% (660) | Respondents were from the general public of Malaysia and aged 18-70 years | NA | Convenience and Snowball |
| #442 | Yang et al. (2021)  [305] | United States | General | Cross-sectional | April 27, 2020 | May 11, 2020 | 2667 | 36.3, NA, 18-89 | 46.0%, (1226) | Residing in the USA;  members of Amazon Mechanical Turk (MTurk); had 90% and above approval ratings from previous MTurk tasks | NA | Convenience |
| #440 | Wang et al. (2021) [306] | 7 countries | General | Cross-sectional | NA | NA | 4479 | NA | 67.8% (3035) | NA | NA | Convenience |
| #439 | Khoury et al. (2021) [307] | Canada | Pregnant women | Cross-sectional | June 3, 2020 | July 31, 2020 | 303 | 33.13, 4.22, 19-44 | 100% (303) | Live in Canada; read/write English; aged ≥18; 36 weeks gestation | NA | Convenience |

**Table 4 (Continued)**

*The Characteristics of the Studies Included in the Systematic Review*

| Study ID | First Author (Year) | Countries | Population | Study Design | Start Date | End date | Participants (N) | Age  (M, SD, Range) | Female (%, n) | Inclusion Criteria | Exclusion Criteria | Sampling Method |
| --- | --- | --- | --- | --- | --- | --- | --- | --- | --- | --- | --- | --- |
|  |  |  |  |  |  |  |  |  |  |  |  |  |
| #759  #756  #744  #738 | Trujillo-Hernández et al. (2021) [308]  Luo (2021) [309]  Wang et al. (2021) [310]  Lee et al. (2021) [311] | Mexico  United States  China  Singapore | General  Middle-aged and older adults  Healthcare workers  Older adults | Cross-sectional  Cross-sectional  Cross-sectional  Cross-sectional | October 5, 2020  June 11, 2020  March 2, 2020  May 13, 2020 | October 24, 2020  NA  April 2, 2020  June 9, 2020 | 501  3,246  19,379  496 | 32.7, 10.3, NA  NA, NA, NA  NA, NA, NA  73.8, 7.6, NA | 68.5% (NA)  56.8% (1137)  80.0% (15,429)  54.8%, (272) | Being at least 18 years of age or older  Older than 46 years  Members of the Chinese Hospital Association (CHA)  Aged 60 years and older; had previously passed 6-CIT; without severe cognitive, hearing, or speech impediment; not diagnosed with COVID-19; and resided in Singapore during the lockdown | NA  NA  Participants from Hubei and provinces with fewer than 50 completed questionnaires; questionnaires completed within 3 mins were excluded; questionnaires that reported a starting work age of less than 16; and questionnaires with age in years minus the number of years spent working yielding a number less than 16  NA | Convenience  Random  Consecutive  Convenience |

**Table 4 (Continued)**

*The Characteristics of the Studies Included in the Systematic Review*

| Study ID | First Author (Year) | Countries | Population | Study Design | Start Date | End date | Participants (N) | Age  (M, SD, Range) | Female (%, n) | Inclusion Criteria | Exclusion Criteria | Sampling Method |
| --- | --- | --- | --- | --- | --- | --- | --- | --- | --- | --- | --- | --- |
|  |  |  |  |  |  |  |  |  |  |  |  |  |
| #723  #702  #693  #691  #687 | Lei et al. (2020) [312]  Ping et al. (2020) [313]  Solomou et al. (2020) [314]  Song et al. (2020) [315]  García-Fernández et al. (2020) [316] | China  China  Cyprus  China  Spain | Adults affected by quarantine and those unaffected  General  Adults  Working adults  Healthy people below and over 60 years old | Cross-sectional  Cross-sectional  Cross-sectional  Cross-sectional  Cross-sectional | February 4, 2020  March 2, 2020  April 3, 2020  April 9, 2020  March 29, 2020 | February 10, 2020  March 10, 2020  April 9, 2020  April 22, 2020  April 5, 2020 | 1,593  1,139  1,642  709  1,639 | 32.3, 9.8, NA  38.3, 12.5, 12-78  NA, NA, NA  35.35, 6.61, NA  NA, NA, NA | 61.3% (976)  59.6% (679)  71.6%, (1176)  74.2%, (526)  ≥ 60 years: 58.7%, (NA) <60 years: 69.2% (NA) | Reside in South-eastern China; and >= 18 years of age  NA  Being 18 years old and above  Working adult; Not infected by COVID-19; and began to work after the Spring Festival  NA | Missing data; responses with obviously false answer; duplicate IP address; and response time less than 2 minutes.  215 respondents were not residents that lived in Changzhi city according to the location; and 146 were deemed unusable due to using time is less than 100 seconds  NA  NA  Healthcare workers; and those with current and/or past mental illnesses. | Convenience  Convenience  Snowball  Snowball  Snowball |

**Table 4 (Continued)**

*The Characteristics of the Studies Included in the Systematic Review*

| Study ID | First Author (Year) | Countries | Population | Study Design | Start Date | End date | Participants (N) | Age  (M, SD, Range) | Female (%, n) | Inclusion Criteria | Exclusion Criteria | Sampling Method |
| --- | --- | --- | --- | --- | --- | --- | --- | --- | --- | --- | --- | --- |
|  |  |  |  |  |  |  |  |  |  |  |  |  |
| #685 | Gasparro et al. (2020) [317] | Italy | Dentist | Cross-sectional | April 17, 2020 | May 3, 2020 | 735 | 44.8, 12.4, 27-70 | 32.7% (240) | Being a Doctor of Dental Science; having at least 2 years of professional experience; working in Italy; and Not being retired | NA | Random |
|  |  |  |  |  |  |  |  |  |  |  |  |  |
| #684  #677  #675  #660  #656 | Newby et al. (2020) [318]  McCracken et al. (2020) [319]  Islam et al. (2020) [320]  Zhao et al. (2020) [321]  Winkler et al. (2020) [322] | Australia  Sweden  Bangladesh  United States  Czech Republic | General  General  University students  Chinese visiting scholars  Adults | Cross-sectional  Cross-sectional  Cross-sectional  Cross-sectional  Repeated cross-sectional | March 27, 2020  May 14, 2020  May 6, 2020  April 20, 2020  T1: November, 2017; T2: May, 2020 | April 7, 2020  June 11, 2020  May 12, 2020  April 21, 2020  T1: November, 2017; T2: May, 2020 | 5,071  1,212  476  311  T1: 3,306; T2: 3,021 | NA, NA, NA  36.1, 15.2, 18-88  NA, NA, NA  NA, NA, 18-60  T1: 48.8, 17.2, NA; T2: 46.8, 16.0, NA | 85.8% (4348)  73.8%, (895)  32.8% (156)  31.8% (99)  T1: 53.7% (1,774); T2: 52.3% (1,581) | Current country of residence is Australia; and age listed as 18 or above  NA  NA  Have permanent jobs in China; receive funding support for their research visit to the US; and stay in the US during the COVID-19 pandemic  Aged ≥18 years; and non-institutionalised | NA  NA  NA  Chinese undergraduate and postgraduate students who were studying in the US; and visiting scholars who had returned to China at the time of the interview  NA | Convenience  Convenience  Snowball  Snowball  Random |

**Table 4 (Continued)**

*The Characteristics of the Studies Included in the Systematic Review*

| Study ID | First Author (Year) | Countries | Population | Study Design | Start Date | End date | Participants (N) | Age  (M, SD, Range) | Female (%, n) | Inclusion Criteria | Exclusion Criteria | Sampling Method |
| --- | --- | --- | --- | --- | --- | --- | --- | --- | --- | --- | --- | --- |
|  |  |  |  |  |  |  |  |  |  |  |  |  |
| #641  #637 | Shatla et al. (2020) [323]  Massad et al. (2020) [324] | Saudi Arabia  Jordan | Adults  General | Cross-sectional  Cross-sectional | April 30, 2020  March 23, 2020 | May 10, 2020  March 30, 2020 | 1,921  5,274 | NA, NA, 18-75  NA, NA, NA | 49.7%, (954)  55.3%, (2914) | NA  Aged 18 years or older; had given consent; lived in Jordan; and use/speak Arabic as their native language | NA  NA | Snowball  Snowball |
|  |  |  |  |  |  |  |  |  |  |  |  |  |
| #636  #630  #629  #622 | Iob et al. (2020) [325]  Nelson et al. (2020) [326]  Généreux et al. (2020) [327]  Kuang et al. (2020) [328] | United Kingdom  United States, Canada, and Europe  8 countries  China | Individuals with high risk  Adults  Adults  Psoriasis patients | Cohort  Cross-sectional  Cross-sectional  Cross-sectional | March 21, 2020  March 19, 2020  April 8, 2020  February 25, 2020 | May 4, 2020  April 10, 2020  June 12, 2020  March 6, 2020 | 51,417  2,065  8,806  926 | NA, NA, NA  34.4, 11.5, 18-77  NA, NA, NA  33.1, 12.2, NA | 51.1% (26,276)  69.2% (1,429)  51.9% (NA)  36.9% (342) | NA  18 years old or older  Any adults (≥18 years) living in each of the eight countries/regions; and able to answer an online questionnaire  NA | NA  Predominantly English speaking countries and geographic regions with sizable sample (n>100 per region); participants were removed if they did not meet age criteria of being 18 or older (n=12); if they did not successfully complete an attention check (n=130); if they had an invalid IP address (n=4)  NA  NA | Stratified  Convenience; and Snowball  Convenience  Convenience |

**Table 4 (Continued)**

*The Characteristics of the Studies Included in the Systematic Review*

| Study ID | First Author (Year) | Countries | Population | Study Design | Start Date | End date | Participants (N) | Age  (M, SD, Range) | Female (%, n) | Inclusion Criteria | Exclusion Criteria | Sampling Method |
| --- | --- | --- | --- | --- | --- | --- | --- | --- | --- | --- | --- | --- |
|  |  |  |  |  |  |  |  |  |  |  |  |  |
| #616  #602 | Smith et al. (2020) [329]  Khan et al. (2020) [330] | United Kingdom  Bangladesh | Adults  University/ college students | Cross-sectional  Cross-sectional | March 17, 2020  April 9, 2020 | NA  April 23, 2020 | 932  505 | NA, NA, NA  NA, NA, 19-25 | 63.3%, (590)  37.2%, (188) | Adults aged ≥18 years; currently residing in the UK; and self-isolating/social distancing due to COVID-19  Being a student of college or university; being home-quarantined; able to speak Bengali; and have been residing in Bangladesh during the outbreak | NA  NA | Convenience  Convenience |
|  |  |  |  |  |  |  |  |  |  |  |  |  |
| #585  #584  #580 | Van Hees et al. (2020) [331]  Ballivian et al. (2020) [332]  Wilson et al. (2020) [333] | 18 countries  Argentina  United States | Persons with epilepsy  People with HIV  Employed individuals | Cross-sectional  Cross-sectional  Cross-sectional | April 10, 2020  NA  April 6, 2020 | May 18, 2020  NA  April 12, 2020 | 399  1,336  474 | 38.22, 12.09, NA  45.8, 10.3, 18-82  Me = 40, NA, 19-85 | 80.2% (320)  33.2% (444)  46.4% (220) | If they provided e-consent; reported to be a PWE or a caretaker/parent of a PWE; and were older than 18 years  NA  Individuals who reported some level of employment | 37 respondents who reported not to be a PWE nor a parent/caretaker of a PWE; and 24 who were younger than 18 years old were excluded  NA  Six participants were excluded from analyses based on problematic response patterns | Convenience  Criterion  Convenience |

**Table 4 (Continued)**

*The Characteristics of the Studies Included in the Systematic Review*

| Study ID | First Author (Year) | Countries | Population | Study Design | Start Date | End date | Participants (N) | Age  (M, SD, Range) | Female (%, n) | Inclusion Criteria | Exclusion Criteria | Sampling Method |
| --- | --- | --- | --- | --- | --- | --- | --- | --- | --- | --- | --- | --- |
|  |  |  |  |  |  |  |  |  |  |  |  |  |
| #1706 | Montano et al. (2020) [334] | Philippines | General | Cross-sectional | June 6, 2020 | June 15, 2020 | 433 | 25.5, NA, 15-65 | 74.4% (322) | Filipino; current location must be inside Philippines; and can understand English | Current location is outside Philippines | Convenience |
| #1695 | Skapinakis et al. (2020) [335] | Greece | General | Cross-sectional | April 8, 2020 | April 12, 2020 | 3379 | 42.0, 12.6, NA | 73% (NA) | Aged ≥ 18 years | NA | Convenience |
|  |  |  |  |  |  |  |  |  |  |  |  |  |
| #1473 | Yáñez et al. (2020) [336] | Peru | Healthcare workers | Cross-sectional | April10, 2020 | May 2, 2020 | 303 | NA, NA, NA | 64% (194) | NA | NA | Convenience |
|  |  |  |  |  |  |  |  |  |  |  |  |  |
|  |  |  |  |  |  |  |  |  |  |  |  |  |
| #1454 | Shevlin et al. (2020) [337] | United Kingdom | General | Cross-sectional | March 23, 2020 | March 28, 2020 | 2,025 | 45.4, 15.9, 18-83 | 51.7% (1047) | Aged 18 years or older; able to read and write in English; and resident of the UK | Aged < 18 years; unable to read and write in English; not resident of the UK | Quota |
|  |  |  |  |  |  |  |  |  |  |  |  |  |
| #1415 | Wanberg et al. (2020) [24] | United States | Adults | Cohort | T1: April, 2019  T2: April 16, 2020 | T1: June, 2019  T2: April 19, 2020 | 1,143 | NA, NA, NA | 55.6% (635) | Participant in the RAND American Life Panel | NA | Convenience |
|  |  |  |  |  |  |  |  |  |  |  |  |  |
| #1397 | Sayeed et al. (2020) [338] | Bangladesh | Bangladesh students | Cross-sectional | April 29, 2020 | May 7, 2020 | 589 | NA, NA, NA | 34.3% (202) | Ability to read Bangla; A student; Stays at home during the pandemic; and lives in Bangladesh for the duration of the government-mandated lockdown | NA | Snowball |

**Table 4 (Continued)**

*The Characteristics of the Studies Included in the Systematic Review*

| Study ID | First Author (Year) | Countries | Population | Study Design | Start Date | End date | Participants (N) | Age  (M, SD, Range) | Female (%, n) | Inclusion Criteria | Exclusion Criteria | Sampling Method |
| --- | --- | --- | --- | --- | --- | --- | --- | --- | --- | --- | --- | --- |
|  |  |  |  |  |  |  |  |  |  |  |  |  |
| #1353 | Wang et al. (2021) [339] | China | COVID-19 patients | Cross-sectional | February 2, 2020 | March 5, 2020 | 460 (including 187 HCWs) | NA, NA, NA | 64.6% (297) | COVID-19 patients who were not in critical conditions; capable of completing the survey | NA | Stratified random |
|  |  |  |  |  |  |  |  |  |  |  |  |  |
| #1321 | Wang et al. (2021) [340] | China | Primary medical staff | Cross-sectional | NA | NA | Study group: 180; Control group: 182 | Study group: 38.0, 10.0, NA; Control group: 37.7, 10.0, NA | Study group: 48.9% (88); Control group: 41.8% (76) | Study group: Participated in first-line epidemic prevention; and voluntarily participated in this study; Control group: h  Healthy people who did not suffer from COVID-19; voluntarily participated in this study; and junior high school education level or above | Study group: Complicated with mental and physical diseases; and did not complete all scale measurements; Control group: Complicated with other mental and physical diseases; and did not complete all scale measurements | Cluster |
|  |  |  |  |  |  |  |  |  |  |  |  |  |
| #1293 | Marthoenis et al. (2021) [341] | Indonesia | Nurses | Cross-sectional | July 20, 2020 | August 19, 2020 | 491 | NA, NA, 22-56 | 72.5% (375) | NA | NA | Convenience |
|  |  |  |  |  |  |  |  |  |  |  |  |  |
| #1278 | Toledo-Fernández et al. (2021) [342] | Mexico | Adults between 18 and 60 years old | Cohort | T1: April 8, 2020  T2: May 11, 2020 | T1: April 18, 2020  T2: May 27, 2020 | 670 | NA, NA, 18-60 | 58.2% (390) | NA | Inconsistent data between two waves (e.g., mismatched reported emails) – 118 cases excluded (14.97%) | Convenience |
|  |  |  |  |  |  |  |  |  |  |  |  |  |

**Table 4 (Continued)**

*The Characteristics of the Studies Included in the Systematic Review*

| Study ID | First Author (Year) | Countries | Population | Study Design | Start Date | End date | Participants (N) | Age  (M, SD, Range) | Female (%, n) | Inclusion Criteria | Exclusion Criteria | Sampling Method |
| --- | --- | --- | --- | --- | --- | --- | --- | --- | --- | --- | --- | --- |
|  |  |  |  |  |  |  |  |  |  |  |  |  |
| #1239 | Tuan et al. (2021) [343] | Vietnam | Healthcare workers | Cross-sectional | August 1, 2020 | August 31, 2020 | 611 | 32.5, 8.4, NA | 74.1% (453) | Aged ≥18 years; healthcare professionals including physicians, nurses, and technicians; working at one of the hospitals in Da Nang city and Quang Nam province; and agreeing to participate in the survey by providing an online consent form | NA | Snowball |
|  |  |  |  |  |  |  |  |  |  |  |  |  |
| #1213 | Sisay et al. (2021) [344] | Ethiopia | Street traditional women coffee vendors | Cross-sectional | December 10, 2020 | December 30, 2020 | 180 | 31.8, 10.0, NA | 100% (180) | Women aged ≥18 years; working on street traditional coffee vending in Harar town; and available during the data collection period | NA | Convenience |
|  |  |  |  |  |  |  |  |  |  |  |  |  |
| #1205 | Mekhemar et al. (2021) [345] | Germany | Dental students | Cross-sectional | July, 2020 | January, 2021 | 211 | NA, NA, NA | 73.5% (155) | NA | NA | Convenience |
|  |  |  |  |  |  |  |  |  |  |  |  |  |
| #1189 | Klimkiewicz et al. (2021) [346] | Poland | Psychiatric patients and general | Cross-sectional | April 1, 2020 | May15, 2020 | Patient group: 212; Healthy group: 207 | Patient: 36, NA, 19-74; Healthy: 35, NA, 19-74 | Patient: 69.3% (147); Healthy: 69.1% (143) | Psychiatric outpatient clinics in Warsaw, Poland, and had a diagnosis of somatoform, neurotic or stress-related disorders, or depression, according to the ICD-10; eligible patients also had a psychiatric consultation during the last year | Healthy participants who reported receiving psychiatric care were moved to the patient group | Patients: convenience; Healthy: snowball |
|  |  |  |  |  |  |  |  |  |  |  |  |  |

**Table 4 (Continued)**

*The Characteristics of the Studies Included in the Systematic Review*

| Study ID | First Author (Year) | Countries | Population | Study Design | Start Date | End date | Participants (N) | Age  (M, SD, Range) | Female (%, n) | Inclusion Criteria | Exclusion Criteria | Sampling Method |
| --- | --- | --- | --- | --- | --- | --- | --- | --- | --- | --- | --- | --- |
|  |  |  |  |  |  |  |  |  |  |  |  |  |
| #1166 | Mâsse et al. (2021) [347] | Canada | Grade 7 Canadian children | Cross-sectional | May, 2020 | June, 2020 | 254 | Mother: 45.5, 4.8, NA  Child: 13, 0.1, 5-17 | Mother: 100% (254)  Child: 54% (254) | Both child and parent have completed the questionnaire in English | Participants with missing data on the main outcome of the study | Cluster |
|  |  |  |  |  |  |  |  |  |  |  |  |  |
| #1140 | Watkins-Martin et al. (2021) [348] | Canada | Young adults | Cohort | T1: Spring, 2018  T2: July, 2020 | T1: Spring, 2018  T2: August, 2020 | 1039 | T1: 20 years  T2: 22 years | 59.9% (622) | Participants in Quebec longitudinal Study of Child Development (QLSCD) and have provided their mental health information on both time points | NA | Convenience |
|  |  |  |  |  |  |  |  |  |  |  |  |  |
| #1074 | Nam et al. (2021) [349] | Vietnam | General | Cross-sectional | April, 2020 | April, 2020 | 1,249 | NA, NA, NA | 58.4% (730) | Aged ≥ 18 years old; living in Vietnam during the survey period; access to internet to answer the questionnaire via Google Form | NA | Snowball |
|  |  |  |  |  |  |  |  |  |  |  |  |  |
| #1065 | Malesza et al. (2021) [350] | Poland | General | Cross-sectional | March 29, 2020 | April 17, 2020 | 1,019 | 37.7, 15.2, 18-74 | 54.1% (551) | NA | Diagnosed with COVID-19; inattentive and negligent responding | Convenience |
|  |  |  |  |  |  |  |  |  |  |  |  |  |
| #1059 | Prati (2021) [63] | Italy | General | Cross-sectional | April, 2020 | April, 2020 | 1,569 | 31.3, 12.4, 18-72 | 21.5% (1278) | Living in Italy; aged ≥18 years | NA | Snowball |
|  |  |  |  |  |  |  |  |  |  |  |  |  |
| #1020 | López-Castro et al. (2021) [351] | United States | Students | Cross-sectional | May 1, 2020 | May 31, 2020 | 909 | NA, NA, NA | 69.2% (629) | NA | NA | Convenience |
|  |  |  |  |  |  |  |  |  |  |  |  |  |
| #940 | Ettman et al. (2021) [352] | United States | Adults | Cross-sectional | March 31, 2020 | April 13, 2020 | 1441 | NA, NA, NA | 49.8% (718) | United States adults aged ≥ 18 years from NORC AmeriSpeak standing panel | Participants with missing data on depression | Probability |

**Table 4 (Continued)**

*The Characteristics of the Studies Included in the Systematic Review*

| Study ID | First Author (Year) | Countries | Population | Study Design | Start Date | End date | Participants (N) | Age  (M, SD, Range) | Female (%, n) | Inclusion Criteria | Exclusion Criteria | Sampling Method |
| --- | --- | --- | --- | --- | --- | --- | --- | --- | --- | --- | --- | --- |
|  |  |  |  |  |  |  |  |  |  |  |  |  |
| #933 | Kwong et al. (2021) [353] | United Kingdom | Data from Avon Longitudinal Study of Parents and Children | Cohort | Index generation: April 9, 2020  Parent generation: April 9, 2020  Generation Scotland: April 17, 2020 | Index generation: May 14, 2020  Parent generation: May 14, 2020  Generation Scotland: May 17, 2020 | Index generation: 2827  Parent Generation: 3579  Generation Scotland: 4208 | Index generation: 27.6, 0.5, NA  Parent Generation: 58.7, 4.82, NA  Generation Scotland: 59.2, 12.0, NA | NA | Completed at least one mental health measure during the COVID-19 survey | NA | Random |
|  |  |  |  |  |  |  |  |  |  |  |  |  |
| #883 | Minhas et al. (2021) [354] | Canada | Emerging adults with alcohol misuse | Cohort | T1: 5 months earlier  T2: June 17, 2020 | T1: 5 months earlier  T2: July 1, 2020 | 473 | T1: 23.4, 1.2, NA  T2: 23.8, 1.3, NA | 58.4% (276) | Heavy episodic drinking (HED: ≥ 4/3 standard drinks for male/female) on at least 2 days in the past month or at least one HED episode and one cannabis use episode per month (i.e., high-risk substance use: 8% of the sample; aged 19.5 to 23 years; fluency in written English; and no current or past psychosis (i.e., schizophrenia, schizoaffective disorder, or similar conditions) | NA | Convenience |
|  |  |  |  |  |  |  |  |  |  |  |  |  |
| #831 | Meraya et al. (2021) [355] | Saudi Arabia | Mothers and pregnant women | Cross-sectional | July, 2020 | August, 2020 | 628 | 31.8, 7.2, NA | 100% (628) | Aged 18 years and older; either had a child under 10 years of age or were pregnant | NA | Snowball |
|  |  |  |  |  |  |  |  |  |  |  |  |  |
| #812 | Rutland-Lawes et al. (2021) [64] | United Kingdom | Middle-aged and older adults | Cross-sectional | June, 2020 | July, 2020 | 5,331 | 70.3, 9.3, NA | 57.0%, NA | Participants with data on all the variables under investigation | Participants with missing data | Convenience |

**Table 4 (Continued)**

*The Characteristics of the Studies Included in the Systematic Review*

| Study ID | First Author (Year) | Countries | Population | Study Design | Start Date | End date | Participants (N) | Age  (M, SD, Range) | Female (%, n) | Inclusion Criteria | Exclusion Criteria | Sampling Method |
| --- | --- | --- | --- | --- | --- | --- | --- | --- | --- | --- | --- | --- |
|  |  |  |  |  |  |  |  |  |  |  |  |  |
| #2742  #2685  #2627  #2586  #2583 | Garvey et al. (2021) [356]  Yao et al. (2021) [357]  Lai et al. (2021) [358]  Sultana et al. (2021) [359]  Wang et al. (2021) [360] | Spain  United States  China  Bangladesh  China | Undergraduate students in Spain  American families  Hong Kong citizens  Wage earners  Pregnant women | Cross-sectional  Repeated cross-sectional  Repeated cross-sectional  Cross-sectional  Cross-sectional | NA  April 23, 2020  July, 2019  May 20, 2020  February 24, 2020 | NA  September, 2020  July, 2020  May 30, 2020  March 3, 2020 | 198  1,576,770  1,112  NA  15,428 | 33.8, 3.6, NA  48.0, NA, NA  43.4, 16.8, NA  31.4, 8.7, 18 – 75  NA | 67% (133)  52% (814,08)  46% (515)  23% (161)  100% (15,428) | NA  NA  Hong Kong Chinese residents; above 15 years old; Cantonese-speaking  Bangladeshi workers living in the country during the COVID-19 outbreak; aged ≥18 years; being able to understand the Bangla language  Female; aged ≥ 18 years; Chinese speaking; currently pregnant; using maternal healthcare services of Maternal and Child Health Hospitals of the Chinese Preventive Medicine Association from all provincial level administrative regions in China; provided complete information | NA  NA  NA  NA  Planned to terminate pregnancy | Convenience  Stratified  Random  Convenience  Convenience |

**Table 4 (Continued)**

*The Characteristics of the Studies Included in the Systematic Review*

| Study ID | First Author (Year) | Countries | Population | Study Design | Start Date | End date | Participants (N) | Age  (M, SD, Range) | Female (%, n) | Inclusion Criteria | Exclusion Criteria | Sampling Method |
| --- | --- | --- | --- | --- | --- | --- | --- | --- | --- | --- | --- | --- |
|  |  |  |  |  |  |  |  |  |  |  |  |  |
| #2578  #2563  #2527  #2523  #2496  #2391  #2379  #2372 | Liu et al. (2021) [361]  Kohls et al. (2021) [362]  Millevert et al. (2021) [363]  Zheng et al. (2021) [364]  Passavanti et al. (2021) [365]  Peng et al. (2021) [366]  Yan et al. (2021) [367]  Liu et al. (2020) [368] | United States  Germany  19 countries  China  7 countries  China  China  China | United States pregnant women during the COVID-19 pandemic  German university students  Persons with epilepsy  Middle-aged to older adults  General  Intensive care unit (ICU) workers  People under quarantine  General public | Cross-sectional  Cross-sectional  Cross-sectional  Cross-sectional  Cross-sectional  Cross-sectional  Cross-sectional  Cross-sectional | May 6, 2020  July 1, 2020  July 26, 2020  February 28, 2020  April 17, 2020  April 1, 2020  February 29, 2020  February 1, 2020 | May 8, 2020  August 1, 2020  December 3, 2020  March 11, 2020  April 20, 2020  April 8, 2020  April 10, 2020  February 10, 2020 | 715  3,382  407  3,730  1,612  731  1,260  2,992 | NA  NA  34.5, 14.0, NA  54.4, 6.0, NA  28.0, 9.4, NA  NA  NA  NA | 100% (715)  70% (2,374)  75% (304)  54% (2,026)  60% (968)  59% (428)  44% (552)  54% (1,532) | 18 to 44 years of age  Currently enrolled as a student  Persons with epilepsy  Aged ≥ 50 years; completed all the survey questions  NA  NA  Had a travel history to high-risk areas or countries; placed under mandatory quarantine in Ningxia Province, China  Chinese citizens aged ≥ 18 years | NA  NA  Not a person with epilepsy; not a caretaker of a person with epilepsy  Infected with COVID-19  NA  NA  Could not access the Internet or other mobile devices due to vision or other disabilities leading to an inability to finish the online questionnaire  NA | Convenience  Convenience  Convenience  Convenience  Convenience  Convenience  Convenience  Snowball |

**Table 4 (Continued)**

*The Characteristics of the Studies Included in the Systematic Review*

| Study ID | First Author (Year) | Countries | Population | Study Design | Start Date | End date | Participants (N) | Age  (M, SD, Range) | Female (%, n) | Inclusion Criteria | Exclusion Criteria | Sampling Method |
| --- | --- | --- | --- | --- | --- | --- | --- | --- | --- | --- | --- | --- |
|  |  |  |  |  |  |  |  |  |  |  |  |  |
| #2368  #2358  #2356  #2355  #2341  #2340 | Zhao et al. (2021) [369]  Vrublevska et al. (2021) [370]  Shen et al. (2021) [371]  Kinser et al. (2021) [372]  Rondung et al. (2021) [373]  Yadav et al. (2021) [374] | China  Latvia  China  United States  Sweden  Nepal | Medical staff  General  General  Pregnant and postpartum women  General  University students | Cross-sectional  Cross-sectional  Cross-sectional  Cross-sectional  Cross-sectional  Cross-sectional | November 5, 2020  July 6, 2020  February 4, 2021  April 1, 2020  March 26, 2020  June 1, 2020 | November 12, 2020  July 27, 2020  February 26, 2021  June 1, 2020  April 5, 2020  June 7, 2020 | 123  2,608  2,361  524  1,503  409 | 37.0, 7.9, NA  46.4, 14.6, NA  29.7, 6.9, NA  32.6, 4.5, 18 – 48  NA  22.1, 2.9, 18 – 37 | 72% (89)  52% (1,344)  60% (1,419)  100% (524)  82% (1,232)  83% (340) | Medical staff; could read a Chinese questionnaire; WeChat user; volunteered for the survey  NA  Chinese citizens aged ≥ 18 years; being able to understand and read Chinese  Adult pregnant and post-partum (up to 6 months post-delivery) women  Aged ≥ 18 years  Health science students studying at graduate and postgraduate levels; aged ≥ 18 years | Unable to understand the questionnaire  NA  Aged < 18; resided in China for less than 12 months  NA  NA  NA | Convenience  Randomized stratified  Convenience  Snowball  Convenience  Convenience |

**Table 4 (Continued)**

*The Characteristics of the Studies Included in the Systematic Review*

| Study ID | First Author (Year) | Countries | Population | Study Design | Start Date | End date | Participants (N) | Age  (M, SD, Range) | Female (%, n) | Inclusion Criteria | Exclusion Criteria | Sampling Method |
| --- | --- | --- | --- | --- | --- | --- | --- | --- | --- | --- | --- | --- |
|  |  |  |  |  |  |  |  |  |  |  |  |  |
| #2339  #2326  #2312  #2293  #2123 | Song et al. (2020) [375]  Sazakli et al. (2021) [376]  Jolliff et al. (2021) [377]  Hyun et al. (2021) [378]  Krumer-Nevo et al. (2021) [379] | China  Greece  United States  South Korea  Israel | Chinese adults  University students  Adolescents  General public  Poverty versus the non-poverty | Cross-sectional  Cross-sectional  Repeated cross-sectional  Cross-sectional  Mixed-method | February 8, 2020  April 15, 2020  T1: October, 2019; T2: March 31, 2020  March 17, 2020  May, 2020 | March 9, 2020  May 7, 2020  T1: February, 2020; T2: April 3, 2020  March 31, 2020  May, 2020 | 3,180  2,009  T1: 100; T2: 134  1,014  273 | 34.1, 12.5, NA  22.0, 3.4, NA  T1: 14.7, 1.3, NA; T2: 15.2, 1.4, NA  NA  39.4, 12.1, 20–68 | 72% (2,294)  67% (1,352)  T1: 66% (66); T2: 69% (92)  49% (498)  68% (186) | China citizens; not diagnosed with COVID-19  NA  Aged 13 to 17 years; lived in the United States; spoke English  NA  Poverty: Earning up to 15,000 NIS per month, receiving basic income allowance or alimony payments from the National Insurance Institute, needing food during crisis, being treated by social workers, inability to pay loans; Non-poverty: NA | Have a history of severe mental disorders which affect brain metabolism; encountered a significant life event in the past 6 months such as losing relatives; a prevention and control frontline personnel including medical staff and their family members; diagnosed or suspected COVID-19 patients.  NA  NA  NA  NA | Convenience  Convenience  Convenience  Stratified  Snowball and cluster |

**Table 4 (Continued)**

*The Characteristics of the Studies Included in the Systematic Review*

| Study ID | First Author (Year) | Countries | Population | Study Design | Start Date | End date | Participants (N) | Age  (M, SD, Range) | Female (%, n) | Inclusion Criteria | Exclusion Criteria | Sampling Method |
| --- | --- | --- | --- | --- | --- | --- | --- | --- | --- | --- | --- | --- |
|  |  |  |  |  |  |  |  |  |  |  |  |  |
| #2055  #1865 | Pagorek-Eshel et al. (2021) [380]  Mistry et al. (2021) [381] | Israel  Myanmar | Arab adults  Older adults | Cross-sectional  Cross-sectional | May, 2020  October, 2020 | July, 2020  October, 2020 | 665  416 | 31.7, 11.1, 18–65  NA | 72% (476)  40% (165) | Aged ≥ 18 years; belonging to the Arab sector; Provided informed consent  Aged ≥ 60 years; residing in Rohingya refugee camps situated in the South-Eastern part of Bangladesh | NA  Severe mental illnesses (clinically proven schizophrenia, bipolar mood disorder, dementia/cognitive impairment); hearing disability; unable to communicate | Convenience and snowball  Convenience |
|  |  |  |  |  |  |  |  |  |  |  |  |  |
| #3004 | Kaplan Serin et al. (2021) [382] | Turkey | Nursing students | Cross-sectional | June 1, 2020 | June 30, 2020 | 344 | 20.9, 2.3, NA | 71% (244) | NA | NA | Convenience |
|  |  |  |  |  |  |  |  |  |  |  |  |  |
| #3003 | Leaune et al. (2021) [383] | France | University students | Cross-sectional | April 6, 2020 | April 13, 2020 | 1,765 | 21.8, 4.2, NA | 82% (1,433) | NA | NA | Snowball |
|  |  |  |  |  |  |  |  |  |  |  |  |  |
| #2999 | McDowell et al. (2021) [384] | United States | Adults in employment prior to COVID-19 | Cross-sectional | April 3, 2020 | April 7, 2020 | 2,301 | NA, NA, NA | 66% (NA) | Students, faculty, staff, and alumni of Iowa State University | Missing employment, mental health, and covariate data; implausible values for body mass index (i.e. 4 standard deviations above the mean) and activity (i.e. >16 hours/day of physical activity or >20 hours/day of physical activity and sitting) | Convenience and snowball |
|  |  |  |  |  |  |  |  |  |  |  |  |  |
|  |  |  |  |  |  |  |  |  |  |  |  |  |

**Table 4 (Continued)**

*The Characteristics of the Studies Included in the Systematic Review*

| Study ID | First Author (Year) | Countries | Population | Study Design | Start Date | End date | Participants (N) | Age  (M, SD, Range) | Female (%, n) | Inclusion Criteria | Exclusion Criteria | Sampling Method |
| --- | --- | --- | --- | --- | --- | --- | --- | --- | --- | --- | --- | --- |
|  |  |  |  |  |  |  |  |  |  |  |  |  |
| #2992 | Killgore et al. (2021) [385] | United States | General | Cross-sectional | April 9, 2020 | April 10, 2020 | 1,013 | 36.7, 12.1, NA | 56.4% (567) | Located within the United States (verified by IP address geo-coordinates); ≥18 years old; reported English as primary language | Failing to correctly answer embedded attention check question | Stratified |
|  |  |  |  |  |  |  |  |  |  |  |  |  |
| #2991 | Qiu et al. (2021) [386] | China | Psychiatric patients and their family members | Cross-sectional | April 27, 2020 | May 8, 2020 | 500 (269 psychiatric patients and 231 family members) | Psychiatric patients: 27.1, 11.9, 20-34 Family members: 41.6, 10.4, 35-49 | Patients: 63% (168) Family members: 56% (129) | Psychiatric patients: ≥16 years old; previously diagnosed by psychiatrists to suffer from various psychiatric illnesses based on ICD-10 criteria Family members: ≥18 years old; do not suffer from psychiatric illness; have caregiving relationship with patients | Inability to complete a survey; presence of severe chronic medical disorders (including neurological, cardiovascular, respiratory, endocrine, and inflammatory disorders); suspected or confirmed case of COVID-19 | Convenience |
|  |  |  |  |  |  |  |  |  |  |  |  |  |
| #2952 | Zhou et al. (2021) [387] | China | Elderly | Cross-sectional | February 19, 2020 | March 19, 2020 | 1,278 | NA, NA, NA | 55% (707) | ≥60 years old | Serious cognitive impairment; confirmed or suspected cases of COVID-19; incomplete information or logic problem | Convenience |
|  |  |  |  |  |  |  |  |  |  |  |  |  |
| #2936 | Kira et al. (2021) [388] | Turkey | Adults | Cross-sectional | October 2, 2020 | November 13, 2020 | 262 | 28.3, 10.4, 18-73 | 29% (77) | >18 years old; being literate enough to access and complete the online survey; consent to participate | NA | Snowball |
|  |  |  |  |  |  |  |  |  |  |  |  |  |

**Table 4 (Continued)**

*The Characteristics of the Studies Included in the Systematic Review*

| Study ID | First Author (Year) | Countries | Population | Study Design | Start Date | End date | Participants (N) | Age  (M, SD, Range) | Female (%, n) | Inclusion Criteria | Exclusion Criteria | Sampling Method |
| --- | --- | --- | --- | --- | --- | --- | --- | --- | --- | --- | --- | --- |
|  |  |  |  |  |  |  |  |  |  |  |  |  |
| #2920 | Su et al. (2021) [389] | China | Medical staff | Cross-sectional | February 3, 2020 | February 17, 2020 | 2,920 (470 frontliners and 2,450 non-frontliners) | NA, NA, NA | 87% (2,525) | Medical staff | NA | Convenience |
| #2919 | Wolfson et al. (2021) [390] | United States | Low-income adults | Cross-sectional | March 19, 2020 | March 24, 2020 | 1,476 | NA, NA, NA | 50% (744) | NA | Completed the survey in <4 minutes; indicated to live outside the United States; missing data | Quota |
| #2912 | Mekhemar et al. (2021) [391] | Germany | Dentists | Cross-sectional | July, 2020 | November, 2020 | 732 | NA, NA, NA | 60% (437) | None | None | Convenience |
| #2910 | King et al. (2021) [392] | United States | Pregnant women | Cross-sectional | March, 2020 | May, 2020 | 725 | Pre-pandemic: 32.6, 5.0, 20-44 Pandemic: 33.7, 4.4, 19-50 | 100% (725) | Currently pregnant; aged ≥ 18 years; fluent in English; no immediate plans to leave the geographic area | Bipolar disorder; psychosis; severe learning disabilities | Convenience |
| #2890 | Teng et al. (2021) [393] | China | Frontline staff | Cross-sectional | March 1, 2020 | March 15, 2020 | 2,614 | NA, NA, NA | 56% (1,453) | Frontline staff | NA | Convenience |
| #2888 | Saw et al. (2021) [394] | Singapore | Migrant workers | Cross-sectional | June 22, 2020 | October 11, 2020 | 1,011 | NA, NA, 21-60 | 0% (0) | Aged ≥ 21 years; has a government work permit for employment status | NA | Convenience |
| #2881 | Oryan et al. (2021) [395] | Israel | General | Cross-sectional | April 19, 2020 | May 2, 2020 | 655 | NA, NA, NA | 63% (409) | Aged ≥ 18 years; fluent in Hebrew | Failed to complete all the questionnaires | Snowball |
| #2879 | Kim (2021) [396] | United States | Working-aged adults | Repeated cross-sectional | September 2, 2020 | December 21, 2020 | 91,222 | NA, NA, 18-64 | NA | 18-64 years old; reported a loss of household employment income since the beginning of the pandemic | NA | Convenience |

**Table 4 (Continued)**

*The Characteristics of the Studies Included in the Systematic Review*

| Study ID | First Author (Year) | Countries | Population | Study Design | Start Date | End date | Participants (N) | Age  (M, SD, Range) | Female (%, n) | Inclusion Criteria | Exclusion Criteria | Sampling Method |
| --- | --- | --- | --- | --- | --- | --- | --- | --- | --- | --- | --- | --- |
|  |  |  |  |  |  |  |  |  |  |  |  |  |
| #2874 | Qiu et al. (2021) [397] | China | Healthcare workers | Single-centre comparative cross-sectional | T1: February 8, 2020 T2: May 27, 2020 | T1: February 15, 2020 T2: June 7, 2020 | T1: 1,717 T2: 2,214 | T1: NA, NA, NA T2: NA, NA, NA | T1: 84% (1,436) T2: 87% (1,918) | Employees of Tongji Hospital who directly provided medical services to patients with confirmed or suspected case of COVID-19 | History of psychiatric disease | Random |
| #2868 | Zhao et al. (2021) [398] | Hong Kong | Adults | Cross-sectional | April 9, 2020 | April 23, 2020 | 1,501 | NA, NA, NA | 53% (829) | NA | NA | Probability |
| #2852 | Miklitz et al. (2021) [399] | Germany | Psychogeriatric patients | Cross-sectional | April 17, 2020 | June 30, 2020 | 219 | 73.0, 0.6, NA | 52% (NA) | Patients are diagnosed with psychiatric disorder or neurodegenerative disease; relatives of patients are ≥18 years old and can complete the questionnaire in German | NA | Convenience |
| #2846 | Sundermeir et al. (2021) [400] | United States | Adults | Cross-sectional | December 15, 2020 | December 23, 2020 | 8,355 | NA, NA, NA | 52% (4,222) | Aged ≥ 18 years old | Missing data; selected “Other” for gender | Random |
| #2822 | Gangwar et al. (2021) [401] | India | Adults | Cross-sectional | June 2, 2020 | June 15, 2020 | 119 | 36.0, 18.0, NA | 52% (62) | Healthy; ≥18 years old | Having systemic or psychiatric illness; taking medication that can affect higher brain functions | Snowball |
| #2819 | Mojtahedi et al. (2020) [402] | United Kingdom and Ireland | General | Cross-sectional | Sample A: April 23, 2020 Sample B: May 18, 2020 | Sample A: May 21, 2020 Sample B: May 25, 2020 | 723 | 35.1, 13.7, 18-78 | 56% (407) | Aged ≥ 18 years; speak English proficiently | NA | Convenience |
| #2815 | Obrenovic et al. (2021) [403] | United States | White-collar employees | Cross-sectional | May, 2020 | August, 2020 | 347 | NA, NA, NA | 49% (168) | Completed the questionnaire | Incomplete responses | Purposive |
|  |  |  |  |  |  |  |  |  |  |  |  |  |

**Table 4 (Continued)**

*The Characteristics of the Studies Included in the Systematic Review*

| Study ID | First Author (Year) | Countries | Population | Study Design | Start Date | End date | Participants (N) | Age  (M, SD, Range) | Female (%, n) | Inclusion Criteria | Exclusion Criteria | Sampling Method |
| --- | --- | --- | --- | --- | --- | --- | --- | --- | --- | --- | --- | --- |
|  |  |  |  |  |  |  |  |  |  |  |  |  |
| #2811 | McArthur et al. (2021) [404] | Canada | Children | Cross-sectional | May, 2020 | August, 2020 | 846 | NA, NA, NA | Children: 47% (398) | Mothers provided consent for child to participate; children provided assent to participate | Incomplete or invalid data | Convenience |
| #2809 | Oliva et al. (2021) [405] | Italy | Paediatric subjects and children and adolescents with neuro-psychiatric disorder | Cross-sectional | May 10, 2020 | May 31, 2020 | Paediatric subjects (P): 9,688 Children and adolescents with neuro-psychiatric disorder (N): 289 | NA, NA, NA | P: 48% (4,622)  N: 35% (101) | Families with at least one child aged <18 years and having access to internet and/or social media; children and adolescents with neuropsychiatric disorder followed by the Child Neurology and Psychiatry Unit at the Department of Human Neuroscience at Sapienza University of Rome | NA | Convenience |
| #2773 | Wagner et al. (2021) [406] | Uganda | People living with HIV | Cohort | T1: March, 2020 T2: June, 2020 | T1: March, 2020 T2: September, 2020 | 280 | 37.5, 12.8, NA | 63% (176) | Aged ≥18 years; on antiretroviral therapy ≥2 years; having adherence problems (i.e. showing lack of viral suppression at most recent assay, being sent to adherence counselling within past 6 months, or showing disease stage 3 or 4 as per WHO guidelines) | NA | Cluster |
| #2765 | Shahriarirad et al. (2021) [407] | Iran | General | Cross-sectional | March 2, 2020 | March 8, 2020 | 8,591 | 34.4, 11.3, 15-87 | 66% (5,703) | Aged ≥ 15 years | NA | Convenience |
|  |  |  |  |  |  |  |  |  |  |  |  |  |

**Table 4 (Continued)**

*The Characteristics of the Studies Included in the Systematic Review*

| Study ID | First Author (Year) | Countries | Population | Study Design | Start Date | End date | Participants (N) | Age  (M, SD, Range) | Female (%, n) | Inclusion Criteria | Exclusion Criteria | Sampling Method |
| --- | --- | --- | --- | --- | --- | --- | --- | --- | --- | --- | --- | --- |
|  |  |  |  |  |  |  |  |  |  |  |  |  |
| #7242 | Okubo et al. (2021) [408] | Japan | General | Cross-sectional | August 25, 2020 | September 30, 2020 | 24,819 | NA, NA, 15-79 | 51.1% (12,425) | NA | NA | Convenience |
|  |  |  |  |  |  |  |  |  |  |  |  |  |
|  |  |  |  |  |  |  |  |  |  |  |  |  |
|  |  |  |  |  |  |  |  |  |  |  |  |  |
| #7206 | Jia et al. (2011) [409] | China | Rural residents | Cross-sectional | February 16, 2020 | March 10, 2020 | 3,892 | NA, NA, NA | 39.9% (1,551) | Have been living in small rural towns and villages for at least five days during the COVID-19 lockdown | NA | Convenience |
|  |  |  |  |  |  |  |  |  |  |  |  |  |
|  |  |  |  |  |  |  |  |  |  |  |  |  |
| #7203 | Kikuchi et al. (2021) [410] | Japan | General | Cohort | T1: Feb 25, 2020 T2: April 1, 2020 T3: May 12, 2020 | T1: Feb 27, 2020  T2: April 6, 2020  T3: May 17, 2020 | 1,993 | 50.5, 15.8, 20-70 | 55.5% (1,008) | NA | NA | Quota |
| #6670 | Robinson et al. (2021) [411] | United States | General | Cohort | T1: Feb 25, 2020  T2: April 1, 2020  T3: May 12, 2020 | T1: Feb 27, 2020  T2: April 6, 2020  T3: May 17, 2020 | 7,138 | 49, 16.5, NA | 51.2% (3,655) | Adults included in the Understanding America Study (UAS) | Responses made between 19th and 31st March as this period marked the beginning of state-wide stay-at-home orders; Responses not submitted within the 14 day survey period; Incomplete data | Convenience |
|  |  |  |  |  |  |  |  |  |  |  |  |  |
| #6641 | Olibamoyo Olushola et al. (2021) [412] | Nigeria | General | Cross-sectional | April 25, 2020 | May 4, 2020 | 755 | NA | 56.7% (428) | People living in Lagos, Ogun, and Abuja after four weeks into the COVID-19 pandemic lockdown | Incomplete data | Convenience |
|  |  |  |  |  |  |  |  |  |  |  |  |  |
| #6487 | Suleiman et al. (2022) [413] | Jordan | General | Cross-sectional | August 1, 2020 | October, 2020 | 1,820 | NA, NA, 18-55 | 55.8% (1,015) | 18 years and older; Living in Jordan | NA | Random |

**Table 4 (Continued)**

*The Characteristics of the Studies Included in the Systematic Review*

| Study ID | First Author (Year) | Countries | Population | Study Design | Start Date | End date | Participants (N) | Age  (M, SD, Range) | Female (%, n) | Inclusion Criteria | Exclusion Criteria | Sampling Method |
| --- | --- | --- | --- | --- | --- | --- | --- | --- | --- | --- | --- | --- |
|  |  |  |  |  |  |  |  |  |  |  |  |  |
| #6296 | Mani et al. (2020) [414] | India | Young adults | Cross-sectional | NA | NA | 618 | NA, NA, 18-34 | 43.0%, (266) | Young adults between the ages of 18 and 34 years | Pre-existing psychiatric disorders; survey forms without responses to the anxiety scale | Snowball |
|  |  |  |  |  |  |  |  |  |  |  |  |  |
|  |  |  |  |  |  |  |  |  |  |  |  |  |
|  |  |  |  |  |  |  |  |  |  |  |  |  |
| #5771 | López Steinmetz et al. (2020) [415] | Argentine | General | Cross-sectional | March 30, 2020 | May 23, 2020 | 1,100 | 31.45, 0.35, NA | 80.27% (883) | Argentineans aged >= 18 years. | NA | Convenience |
|  |  |  |  |  |  |  |  |  |  |  |  |  |
| #5662 | Widyana et al. (2020) [416] | Indonesia | General | Cross-sectional | NA | NA | 587 | NA | 65.6% (385) | NA | NA | Convenience |
|  |  |  |  |  |  |  |  |  |  |  |  |  |
| #5344 | Thomas et al. (2020) [417] | United Arab Emirates | General | Cross-sectional | April 8, 2020 | April 22, 2020 | 1,039 | 28.33, 11.38, NA | 85.6% (859) | Residents of the UAE; aged >= 18 years | NA | Convenience |
|  |  |  |  |  |  |  |  |  |  |  |  |  |
|  |  |  |  |  |  |  |  |  |  |  |  |  |
|  |  |  |  |  |  |  |  |  |  |  |  |  |
| #5307 | Li et al. (2020) [418] | China | Teachers | Cross-sectional | February 4, 2020 | February 12, 2020 | 88,611 | 36.22, 9.02, NA | 76.93% (68169) | NA | Aged < 18 or > 100 years; took less than 100 seconds to complete entire survey | Cluster |
|  |  |  |  |  |  |  |  |  |  |  |  |  |
| #5260 | Salameh et al. (2020) [419] | Lebanon | Adults | Cross-sectional | May 10, 2020 | May 20, 2020 | 502 | 42.47, 14.06, NA | 52.7% (265) | Aged >= 18 years; have access to the internet | NA | Convenience |

**Table 4 (Continued)**

*The Characteristics of the Studies Included in the Systematic Review*

| Study ID | First Author (Year) | Countries | Population | Study Design | Start Date | End date | Participants (N) | Age  (M, SD, Range) | Female (%, n) | Inclusion Criteria | Exclusion Criteria | Sampling Method |
| --- | --- | --- | --- | --- | --- | --- | --- | --- | --- | --- | --- | --- |
|  |  |  |  |  |  |  |  |  |  |  |  |  |
| #5235 | Zajacova et al. (2020) [72] | Canada | General | Cross-sectional | March 29, 2020 | May 10, 2020 | 4.600 | NA | NA | Canadian; aged >= 15 years | Institutionalized adults and residents of the Yukon, Nunavut, and Northwest Territories. | Convenience |
| #5204 | Yamamoto et al. (2020) [420] | Japan | General | Cross-sectional | May 11, 2020 | May 12, 2020 | 11,333 | 46.3, 14.6, 18-89 | 52.4% (5939) | Living in the seven prefectures where the emergency declaration was first applied (Tokyo, Kanagawa, Osaka, Saitama, Chiba, Hyogo, and Fukuoka) | Aged < 18 years; high school students; living outside the seven prefectures | Purposive |
|  |  |  |  |  |  |  |  |  |  |  |  |  |
| #5080 | Zwickl et. al (2021) [421] | Australia | Transgender people | Cross-sectional | May 1, 2020 | June 30, 2020 | 1019 | Me = 29, NA, 16-80 | 52.2% (532) | Currently living in Australia; identification as trans; aged > = 16 years. | Duplicate or incomplete responses | Convenience |
|  |  |  |  |  |  |  |  |  |  |  |  |  |
|  |  |  |  |  |  |  |  |  |  |  |  |  |
| #4946 | Gama et al. (2021) [422] | Brazil | Mothers  of children with confirmed or presumed diagnosis of  congenital Zika syndrome (CZS). | Cross-sectional | NA | NA | 41 | 29.36, 6.24, 20-42 | 100% (41) | Mothers  of children with confirmed or presumed diagnosis of  congenital Zika syndrome (CZS). | Mothers who did not have access to the internet | Convenience |
|  |  |  |  |  |  |  |  |  |  |  |  |  |
| #4852 | Solomou et al. (2021) [423] | Cyprus | University students | Cross-sectional | June 3, 2020 | June 26, 2020 | 387 | NA | 73.1% (283) | Aged >= 17 years; University students from any educational institution in Cyprus | NA | Convenience |
|  |  |  |  |  |  |  |  |  |  |  |  |  |

**Table 4 (Continued)**

*The Characteristics of the Studies Included in the Systematic Review*

| Study ID | First Author (Year) | Countries | Population | Study Design | Start Date | End date | Participants (N) | Age  (M, SD, Range) | Female (%, n) | Inclusion Criteria | Exclusion Criteria | Sampling Method |
| --- | --- | --- | --- | --- | --- | --- | --- | --- | --- | --- | --- | --- |
|  |  |  |  |  |  |  |  |  |  |  |  |  |
|  |  |  |  |  |  |  |  |  |  |  |  |  |
| #4774 | Malek Rivan et al. (2021) [424] | Malaysia | Community-dwelling middle-aged and older adults | Cross-sectional | April 1, 2020 | Jun 1, 2020 | 535 | 71.18, 5.72, NA | 55.5% (297) | Malaysian citizens aged >= 52 years; normal hearing; able to converse in either the Malay, English, Chinese, or Tamil languages; no documented major psychiatric illnesses or mental disorders | NA | Purposive |
|  |  |  |  |  |  |  |  |  |  |  |  |  |
| #4762 | Nishimura et al. (2021) [425] | Japan | Medical students | Cross-sectional | June 8, 2020 | June 14, 2020 | 473 | 22.0, 3.3, NA | 34% (161) | Medical students who belonged to the OUSM as of April 1, 2020 (i.e, the first day of the academic year in Japan), | NA | Convenience |
|  |  |  |  |  |  |  |  |  |  |  |  |  |
| #4670 | Sabat et al. (2021) [426] | India | General | Cross-sectional | July 1, 2020 | August 1, 2020 | 914 | 30.7, 13.1, 21-40 | 39.8% (364) | Individuals aged > 15 years and those able to read English | Not providing informed consent. | Snowball |
|  |  |  |  |  |  |  |  |  |  |  |  |  |
|  |  |  |  |  |  |  |  |  |  |  |  |  |
| #4447 | Torkian et al. (2021) [427] | Iran | General | Cross-sectional | April 19, 2020 | May 12, 2020 | 3,446 | 34.2, 11.60, NA | 66.7% (2300) | Aged > 18 years; able to read and write | NA | Convenience |

**Table 4 (Continued)**

*The Characteristics of the Studies Included in the Systematic Review*

| Study ID | First Author (Year) | Countries | Population | Study Design | Start Date | End date | Participants (N) | Age  (M, SD, Range) | Female (%, n) | Inclusion Criteria | Exclusion Criteria | Sampling Method |
| --- | --- | --- | --- | --- | --- | --- | --- | --- | --- | --- | --- | --- |
|  |  |  |  |  |  |  |  |  |  |  |  |  |
| #3705 | Suarez-Balcazar et al. (2021) [74] | United States | Latinx caregivers of children with IDD. | Cross-sectional | July, 2020 | March, 2021 | 37 | 43.9, 6.9, NA | 100% (37) | Caregiver identified as a mother (or other female primary caregiver who has custody of child) of Latinx descent; the caregiver had a child with IDD between 6 and 17 years of age; the focal child had a diagnosis of autism spectrum disorder, down syndrome, and/or intellectual disability; and the focal child was able to walk. | NA | Convenience |
|  |  |  |  |  |  |  |  |  |  |  |  |  |
| #3685 | Westrupp et al. (2021) [428] | Australia | Parents and children | Cross-sectional | April 8, 2020 | April 28, 2020 | 2,365 | Parents: 38.30, 7.07, NA  Childs: 8.66, 5.14, NA | Parents: 80.7%  Childs: 48.6% | Resided in Australia and were aged >= 18 years, English speaking, and a current parent of a child aged 0-18 years | NA | Convenience |
|  |  |  |  |  |  |  |  |  |  |  |  |  |
| #3620 | Li et al. (2020) [429] | Pakistan | University students | Cross-sectional | May 2, 2020 | May 16, 2020 | 640 | NA | 57.2% | University students at local and foreign universities | NA | Snowball |
|  |  |  |  |  |  |  |  |  |  |  |  |  |
| #3419 | Scarlett et al. (2021) [430] | France | People living in temporary and / or emergency accommodation during France's first lockdown | Cross-sectional | May 2, 2020 | June 7, 2020 | 527 | NA | 24.1% (127) | NA | Aged < 18 years old; significantly inebriated or presenting cognitive disorders that prevented consent. | Convenience |
|  |  |  |  |  |  |  |  |  |  |  |  |  |
| #3344 | Pinchoff et al. (2021) [431] | Kenya | Adolescent | Cross-sectional | June, 2020 | Aug, 2020 | 2,195 | NA | 76.3% (1674) | NA | NA | Random |
|  |  |  |  |  |  |  |  |  |  |  |  |  |

**Table 4 (Continued)**

*The Characteristics of the Studies Included in the Systematic Review*

| Study ID | First Author (Year) | Countries | Population | Study Design | Start Date | End date | Participants (N) | Age  (M, SD, Range) | Female (%, n) | | Inclusion Criteria | Exclusion Criteria | Sampling Method |
| --- | --- | --- | --- | --- | --- | --- | --- | --- | --- | --- | --- | --- | --- |
|  |  |  |  |  |  |  |  |  |  | |  |  |  |
| #3282 | Tsai et al. (2021) [432] | United States | Adults | Cohort | T1: May, 2020  T2: Sep, 2020 | T1: June, 2020  T2: Oct, 2020 | 3,169 | Received EUP before June (n=2612): 54.10, 16.64, NA  Received EIP after June (n=149): 63.21, 23.77, NA  Did not receive EIP (n=408): 51.66, 21.20, NA | Received EUP before June (n=2612) - Male: 37.8% (964)  Received EIP after June (n=149) - Male: 33.9% (78)  Did not receive EIP (n=408) - Male: 33.4% (182) | | Adults who were at least 22 years old, living in the USA and reported an annual personal gross income of $75 000 or less; fulfilled the validity checks and completed the baseline assessment. Participants who had completed â‰¥ 50 approved previous human intelligence tasks (HITs) and had an HIT approval rating â‰¥50% were invited | NA | Convenience |
|  |  |  |  |  |  |  |  |  |  | |  |  |  |
| #3267 | Msherghi et al. (2021) [433] | Libya | General | Cross-sectional | May, 2020 | June, 2020 | 8,084 | 27.2, 8.9, 25-50 | 63% (5090) | | NA | NA | Convenience |
|  |  |  |  |  |  |  |  |  |  | |  |  |  |
| #3242 | Myhr et al. (2021) [434] | Norway | Adolescents | Repeated cross-sectional | T1: Spring 2020 (before lockdown)  T2: May 14, 2020 | T1: Spring 2020  T2: May 20, 2020 | T1: 2,443  T2: 2,011 | NA | 8th grade (32%), 9th grade (33%), 10th grade (35%) | | Students in the 10 participating municipalities enrolled in level 2 in the International Standard Classification of Education (ISCED) | Individuals with missing information on gender (n=77) and family SEP (n=294). | Convenience |
|  |  |  |  |  |  |  |  |  |  | |  |  |  |
| #3212 | Kusuma et al. (2021) [435] | Bangladesh, India, Pakistan and Sri Lanka | Adults | Cross-sectional | March, 2020 | July, 2020 | 29,809 | 45.1, 14.1, NA | 61% (18111) | | Aged >= 18 years | Current pregnancy, or serious illness expected to reduce life expectancy to less than 12 months | Stratified |
|  |  |  |  |  |  |  |  |  |  |  | |  |  |

**Table 4 (Continued)**

*The Characteristics of the Studies Included in the Systematic Review*

| Study ID | First Author (Year) | Countries | Population | Study Design | Start Date | End date | Participants (N) | Age  (M, SD, Range) | Female (%, n) | Inclusion Criteria | Exclusion Criteria | Sampling Method |
| --- | --- | --- | --- | --- | --- | --- | --- | --- | --- | --- | --- | --- |
|  |  |  |  |  |  |  |  |  |  |  |  |  |
| #3188 | Rassu et al. (2021) [436] | United States | Adult patients with non-specific chronic low back pain. | Cross-sectional | Early-September, 2020 | Mid-October, 2020 | 97 | 48.5, 11.8, NA | 70.7% (70) | Aged >= 18 years with confirmed nonspecific chronic low back pain who were actively seeking professional healthcare and reported experiencing moderate pain (more than or equal to 4/10) and low back-pain related disability (Oswestry Disability Index is more than or equal to 24) prior to enrolling in the parent clinical trial. | Cancer other than skin cancer; any changes in urination or bowel movement likely related to back pain, non-English speaking; serious pathology as a cause of low back pain, including neoplasm, inflammatory disease, vertebral osteomyelitis, or other conditions; having received physical therapy for low back pain during the 90 days prior to being approached for the parent trial; having received Cognitive Behavioral Therapy or mindfulness therapy during the 90 days prior to being approached for the parent trial; having undergone any lumbar spine surgery during the year prior to being approached; current pregnancy; or current receipt of treatment or counselling for substance use (not including attending meetings of recovery programs such as Alcoholics Anonymous or Narcotics Anonymous). | Cluster |
|  |  |  |  |  |  |  |  |  |  |  |  |  |

**Table 4 (Continued)**

*The Characteristics of the Studies Included in the Systematic Review*

| Study ID | First Author (Year) | Countries | Population | Study Design | Start Date | End date | Participants (N) | Age  (M, SD, Range) | Female (%, n) | Inclusion Criteria | Exclusion Criteria | Sampling Method | |
| --- | --- | --- | --- | --- | --- | --- | --- | --- | --- | --- | --- | --- | --- |
|  |  |  |  |  |  |  |  |  |  |  |  |  | |
| #3187 | Sabate et al. (2021) [437] | France | Participants with and without irritable bowel syndrome | Cross-sectional | March 31, 2020 | April 15, 2020 | 304 | 46.8, 16.8, NA | 75.3% (229) | NA | NA | Snowball | |
|  |  |  |  |  |  |  |  |  |  |  |  |  | |
| #3180 | Lee et al. (2021) [438] | South Korea | Middle school students in public school | Cross-sectional | September, 2020 | October, 2020 | 328 | 14.42, 0.71, 12-16 | 55% | Middle school students living in Gyeonggi Province, South Korea. | Students decline to participate in the online survey; parents refused their children's participation. | Random | |
|  |  |  |  |  |  |  |  |  |  |  |  |  | |
| #3178 | Jing et al. (2021) [439] | China | University and college students | Cross-sectional | February 20, 2020 | February 22, 2020 | 17,876 | NA, NA, NA | 71.7% (12818) | University and college students living in mainland China during the COVID-19 pandemic; students able to complete the questionnaire on a cell phone or computer, and informed consent. | Students unable to use a computer or cell phone to complete the questionnaire or refusing to participate in the survey. | Convenience | |
|  |  |  |  |  |  |  |  |  |  |  |  |  | |
| #3142 | Shangguan et al. (2021) [440] | China | Pregnant women | Cross-sectional | February 28, 2020 | April 26, 2020 | 2,120 | 30.51, 9.67, NA | 100% (2120) | Pregnant Chinese woman; registered in an online self-help intervention program targeting crisis intervention during the COVID-19 epidemic | NA | Convenience | |
|  |  |  |  |  |  |  |  |  |  |  |  |  | |
|  |  |  |  |  |  |  |  |  |  |  |  |  | |
| #3105 | Liu et al. (2021) [441] | China | Chinese adults in urban and rural areas during the COVID-19 outbreak | Cross-sectional | February 1, 2020 | February 10, 2020 | 2,858 | NA, NA, NA | 53.6% (1532) | Chinese citizens who obtained written informed consent and were over 18 years old | Any conditions that affected the quality of the questionnaire, including<10 min of response time, confusion of logic, etc.; non-caregivers were excluded in the sample that meets care needs. | | Snowball |

**Table 4 (Continued)**

*The Characteristics of the Studies Included in the Systematic Review*

| Study ID | First Author (Year) | Countries | Population | Study Design | Start Date | End date | Participants (N) | Age  (M, SD, Range) | Female (%, n) | Inclusion Criteria | Exclusion Criteria | Sampling Method | | |
| --- | --- | --- | --- | --- | --- | --- | --- | --- | --- | --- | --- | --- | --- | --- |
|  |  |  |  |  |  |  |  |  |  |  |  |  | | |
| #3104 | Van de Velde et al. (2021) [442] | 26 countries | Higher-education students | Cross-sectional | April 7, 2020 | April 27, 2020 | 20,103 | NA, NA, NA | 73.9% (14864) | Enrolled in a higher education program, aged >= 17 years, and provided informed consent | Ph.D. students were excluded | Convenience | | |
|  |  |  |  |  |  |  |  |  |  |  |  |  | | |
| #3097 | Saadeh et. al (2021) [443] | Jordan | Undergraduate students | Cross-sectional | NA | NA | 6,157 | 19.79, 1.67, 17-30 | 71.3% (4388) | Undergraduate students at the University of Jordan (UJ, located in Amman) who voluntarily completed its questions | NA | Convenience | | |
|  |  |  |  |  |  |  |  |  |  |  |  |  | | |
| #3081 | Ravens-Sieberer et al. (2021) [444] | Germany | Children and adolescents | Cross-sectional | May 26, 2020 | June 10, 2020 | 1,586 | 12.25, 3.30, 7-17 | 50% (793) | NA | Implausible data | Convenience | | |
|  |  |  |  |  |  |  |  |  |  |  |  |  | | |
| #3080 | Mikolajczyk et al. (2021) [445] | United States, United Kingdom, Canada, Netherlands, Germany, Ireland, Switzerland, and Greece. | Community-dwelling adults with spinal cord injury (SCI) | Cross-sectional | May 1, 2020 | August 31, 2020 | 187 | 57.0, 14.5, NA | 25.1% (47) | Had a diagnosis of SCI or disorder (SCI/D) through self-report;  Aged >= 18 years or older; and were able to comprehend English | Participant information is incomplete; demographic information is incomplete; injury information is incomplete; missing all data on measures | Convenience | | |
|  |  |  |  |  |  |  |  |  |  |  |  |  | | |
| #3070 | Santangelo et al. (2021) [446] | Italy | Nursing students | Cross-sectional | April, 2020 | April, 2020 | 525 | 21.8, 3.83, NA | 70.29%  (369) | NA | NA | Convenience | | |
|  |  |  |  |  |  |  |  |  |  |  |  | | |  |
| #3061 | Morin et al. (2021) [447] | 13 countries across four continents | General (aged 18 and above) | Cross-sectional | May, 2020 | August, 2020 | 22,330 | 41.9, NA, 18-95 | 65.6% (14648) | Aged >= 18 years | Failed to provide consent (n =977); did not indicate gender and age (mandatory for weighting, n =431); or did not complete the Insomnia Severity Index ( n=1746) were excluded | | Convenience | |

**Table 4 (Continued)**

*The Characteristics of the Studies Included in the Systematic Review*

| Study ID | First Author (Year) | Countries | Population | Study Design | Start Date | End date | Participants (N) | Age  (M, SD, Range) | Female (%, n) | Inclusion Criteria | Exclusion Criteria | Sampling Method | | |
| --- | --- | --- | --- | --- | --- | --- | --- | --- | --- | --- | --- | --- | --- | --- |
|  |  |  |  |  |  |  |  |  |  |  |  |  | | |
| #3053 | Spiro et al. (2020) [448] | United Kingdom | Performing arts professionals | Cross-sectional | April 1, 2020 | June 15, 2020 | 385 | 44.08, 13.9, 18-86 | 63% (242) | Those who reported working in at least “music or sound arts” and/or “performing arts” in a range of capacities (including performing, teaching, music therapy, and managing) | Exclude if there was evidence of response bias, such as straight-lining (n = 3) or extreme responses (n = 1) | Respondent Driven | | |
|  |  |  |  |  |  |  |  |  |  |  |  |  | | |
| #3046 | Kira et al. (2021) [449] | Arab countries | General | Cross-sectional | April 28, 2020 | May 25, 2020 | 1,374 | 31.68, 12.92, 18-75 | 82% | NA | NA | Snowball | | |
|  |  |  |  |  |  |  |  |  |  |  |  |  | | |
| #3023 | Leach et al. (2021) [450] | United States | General | Cohort | June, 2020 | NA | 2,359 | 57.4, 9.65, NA | 65% (1534) | NA | Participants were excluded from analyses if they were missing responses to any of the items used to calculate the PHQ-4 score(n=70) | Convenience | | |
|  |  |  |  |  |  |  |  |  |  |  |  |  | | |
| #3009 | Juchnowicz et al. (2021)[451] | Poland | University students | Cross-sectional | April 20, 2020 | April 26, 2020 | 2,172 | 22.1, 2.2, NA | 73% (1585) | NA | NA | Convenience | | |
|  |  |  |  |  |  |  |  |  |  |  |  |  | | |
|  |  |  |  |  |  |  |  |  |  |  |  |  | | |
|  |  |  |  |  |  |  |  |  |  |  |  | | |  |
|  |  |  |  |  |  |  |  |  |  |  |  | |  | |
|  |  |  |  |  |  |  |  |  |  |  |  | |  | |

**Table 4 (Continued)**

*The Characteristics of the Studies Included in the Systematic Review*

| Study ID | First Author (Year) | Countries | Population | Study Design | Start Date | End date | Participants (N) | Age  (M, SD, Range) | Female (%, n) | Inclusion Criteria | Exclusion Criteria | Sampling Method |
| --- | --- | --- | --- | --- | --- | --- | --- | --- | --- | --- | --- | --- |
|  |  |  |  |  |  |  |  |  |  |  |  |  |
| #358 | Song et al. (2021) [452] | United States | Chinese international students | Cross-sectional | NA | NA | 261 | China: 20.1, 2.9, NA  Abroad: 20.3, 2.8, NA | China: 48% (60)  Abroad: 58.1% (79) | Native Chinese students who are studying at U.S. universities | NA | Snowball |
| #355 | Emery et al. (2020) [453] | United States | Young adults | Cross-sectional | April, 2020 | October, 2020 | 670 | 25.2, 1.9, 21-29 | 62% (418) | NA | NA | Convenience |
|  |  |  |  |  |  |  |  |  |  |  |  |  |
| #286 | Zarrouq et al. (2021) [454] | Morocco | General | Cross-sectional | April 3, 2020 | April 30, 2020 | 1,435 | 32.2, 10.5, NA | 43% (624) | Aged 18 years or older; Moroccan | NA | Snowball |
| #252 | Restar et al. (2021) [455] | Global | Trans and non-binary people | Cross-sectional | April 16, 2020 | August 3, 2020 | 849 | NA | NA | Aged 18 years or older; a trans or non-binary individual | NA | Convenience |
| #3198 | Smallwood et. al (2021) [456] | Australia | Frontline healthcare workers | Cross-sectional | August 27, 2020 | October 23, 2020 | 7,846 | NA, NA, NA | 80.9% (6344) | Australian HCWs, comprising medical, nursing, allied health, medical laboratory, administrative and other support staff, who self-identified as frontline HCWs in secondary or primary and community care | NA | Convenience |
|  |  |  |  |  |  |  |  |  |  |  |  |  |
