# Supplementary material for: A systematic review on the relationship between socioeconomic conditions and emotional disorder symptoms during Covid-19: unearthing the potential role of economic concerns and financial strain
Source: BMC Psychol. 2024 Apr 26;12:237. doi: 10.1186/s40359-024-01715-8 (PMC11046828; doi:10.1186/s40359-024-01715-8)
Supplement: Supplementary file 2 — Supplementary Material 2. [file 40359_2024_1715_MOESM2_ESM.zip › Table 5.docx]

**Table 5**

*The Main Outcomes of the Studies Included in the Systematic Review*

| Study ID | Quality Assessment | SEC  Variable | Measurement  Method | Actual / Perceived | | Anxiety    Inventory Outcomes | | Depression    Inventory Outcomes | | Note | |
| --- | --- | --- | --- | --- | --- | --- | --- | --- | --- | --- | --- |
|  |  |  |  |  |  | |  |  |  | |  |
| #6778 | 8 | Socioeconomic Status (SES) | Latent variable assessed using 3 indicators, a) admission through social inclusion quotas; b) monthly family income; and c) reduction of family income | Actual | DASS-21 | | Lower SES increased negative affectivity (e.g., anxiety; B = -1.05, p = .002) | DASS-21 | Lower SES increased negative affectivity (e.g., depression; B = -1.05, p = .002 | |  |
|  |  |  |  |  |  | |  |  |  | |  |
| #6255 | 8 | Fear of job loss due to recession | 3-items inventory measured on 5-point Likert scale (1 = Strongly Disagree and 5 = Strongly Agree) to assess job insecurity and financial impact. | Perceived | GAD-7 | | Non-significant | NA | NA | |  |
|  |  |  |  |  |  | |  |  |  | |  |
| #6116 | 8 | Employment status | Categorized as a) Lost job after 3/1/2020; b) Temporarily laid off/furloughed; c) Hours reduced; d) Hours increased; e) Different schedule; and f) No change. | Actual | GAD-2 | | Lost job after 3/1/2020 was associated with more anxiety (B = 1.08, p = .013). | PHQ-2 | Lost job after 3/1/2020 was associated with worse depression (B = .91, p = .038) | |  |
|  |  |  |  |  |  | |  |  |  | |  |
|  |  | Education | Categorized as a) < High school graduate; b) High school graduate; c) Some college or Associate degree; d) Bachelor's degree; and e) Graduate or professional degree. | Actual |  | | Non-significant |  | Non-significant | |  |
|  |  |  |  |  |  | |  |  |  | |  |
|  |  | Household income (pre-pandemic) | Categorized as a) <$34999; b) $35000-74999; c) $75000-124999; d) $125000-199999; and e) > $200000. | Actual |  | | Lower household income was associated with worse anxiety (B = -0.02, p = .008). |  | Lower household income was associated with worse depression (B = -0.03, p < .001). | |  |
|  |  |  |  |  |  | |  |  |  | |  |
| #5913 | 8 | Education | Categorized as a) Secondary; b) Bachelor; and c) Master | Actual | GAD-7 | | Non-significant | PHQ-9 | Non-significant | |  |
|  |  |  |  |  |  | |  |  |  | |  |

**Table 5 (Continued)**

*The Main Outcomes of the Studies Included in the Systematic Review*

| Study ID | Quality Assessment | SEC  Variable | Measurement  Method | Actual / Perceived | | Anxiety    Inventory Outcomes | | Depression    Inventory Outcomes | | Note | | |
| --- | --- | --- | --- | --- | --- | --- | --- | --- | --- | --- | --- | --- |
|  |  |  |  |  |  | |  |  |  | |  | |
| #5493 | 6 | Education | Categorized as a) Primary; b) Secondary; and c) Tertiary. | Actual | GAD-7 | | Non-significant | PHQ-9 | Non-significant | |  | |
|  |  |  |  |  |  | |  |  |  | |  | |
|  |  | Employment status | Categorized as a) No; b) Yes, but it was interrupted after pandemic; c) Yes, I work from home after pandemic; and d) Yes, I continue to go to work like before pandemic. | Actual |  | | Those who were jobless from the pandemic scored higher on anxiety (F= 9.760, p < 0.05). |  | Those who were jobless from the pandemic scored higher on depression (F= 16.051, p < 0.05). | |  | |
|  |  |  |  |  |  | |  |  |  | |  | |
| #5485 | 8 | Monthly income | Categorized as a) <240.00 USD; b) 240.00 USD - 383.00 USD; c) 384.00 USD - 1,652.00 USD; d) 1653.00USD - 2153.00 USD; e) >2154.00 USD | Actual | DASS-21 | | The lower the income level, the higher the chance of anxiety: *Ref= Category e  category a (OR= 2.69, p< .001)  category b (OR= 2.47, p< .001)  category c (OR= 1.72, p< .001)  category d = (OR= 1.31, p< .001) | DASS-21 | The lower the income level, the higher the chance of depression: *Ref= Category e  category a (OR= 1.68, p< .001)  category b (OR= 1.70, p< .001)  category c (OR= 1.37, p< .001)  category d = Non-significant | |  | |
|  |  |  |  |  |  | |  |  |  | |  | |
|  |  | Education | Categorized as a) Complete elementary I school; b) Complete elementary II school; c) Complete high school; d) Complete higher education; e) Complete graduate school | Actual |  | | Non-significant |  | Lower education levels increased the likelihood of depressive symptoms: completed up to high school (OR= 1.55, p< .001); completed higher education (OR= 1.17, p< .001) | |  | |
|  |  |  |  |  |  | |  |  |  | |  | |
| #5401 | 7 | Education | Years of education received | Actual | GAD-7 | | Non-significant | PHQ-9 | Non-significant | |  | |
|  |  |  |  |  |  | |  |  |  | |  | |
|  |  | Loss of job | Yes/No | Actual |  | | Non-significant |  | Non-significant | |  | |
|  |  |  |  |  |  | |  |  |  | |  | |
|  |  | Financial distress | 6 point Likert scale ranging from Not at All to Extremely. | Perceived |  | | Financial distress was significantly associated with higher anxiety (p < .001) |  | Financial distress was significantly associated with higher depression (p<0.001) | | |  |
|  |  |  |  |  |  | |  |  |  | | |  |
| #5398 | 5 | SES | Categorized as a) Above standard income; b) Standard income; and c) Below standard income | Perceived | GAD-7 | | Non-significant | PHQ-9 | Depressive symptoms was significantly higher among respondents with standard income compare to those above standards and below standard incomes (p < .05) | | |  |
|  |  |  |  |  |  | |  |  |  | | |  |

**Table 5 (Continued)**

*The Main Outcomes of the Studies Included in the Systematic Review*

| Study ID | Quality Assessment | SEC  Variable | Measurement  Method | Actual / Perceived | | Anxiety    Inventory Outcomes | | Depression    Inventory Outcomes | | Note | |
| --- | --- | --- | --- | --- | --- | --- | --- | --- | --- | --- | --- |
|  |  |  |  |  |  | |  |  |  | |  |
| #5227 | 7 | Current professional status | Categorized as a) Undergraduate student, b) Postgraduate student, c) Intern, d) Private practitioner, e) Consultant dentist; f) Academic staff | Actual | NA | | NA | PHQ-9 | Non-significant | |  |
|  |  |  |  |  |  | |  |  |  | |  |
|  |  | Able to meet expenses during lockdown | Categorized as a) With difficulty; b) With other source of income; c) Easily | Perceived |  | | NA |  | Practitioners who were able to meet their financial expenses easily during lockdown were the least likely to be depressed (AOR= 0.50, p =.016) | |  |
| #5189 | 8 | Education | Categorized as a) Secondary or less; b) College; c) Master of doctorate | Actual | NA | | NA | DASS-21 | Non-significant | |  |
|  |  |  |  |  |  | |  |  |  | |  |
|  |  | Monthly income | Categorized as a) < 2000; b) 2000-5000; and c) > 5000 | Actual |  | | NA |  | Lower income (<2000) was associated with higher risk for mild to moderate depression (OR = 1.48, p = .007) but not with severe or extremely severe depression. | |  |
|  |  |  |  |  |  | |  |  |  | |  |
|  |  | Enough food supply | 1-item binary variable (Yes/No) to assess food sufficiency to withstand quarantine period. | Perceived |  | | NA |  | Insufficient food supply was associated with higher risk for severe to extremely severe depression (OR = 1.86, p < .001). | |  |
|  |  |  |  |  |  | |  |  |  | |  |
| #5104 | 7 | Financial issues | 6-items measured on 5-point Likert scale (0 = Not relevant and 4 = Strongly agree) with item such as "I have lost my income during MCO". | Hybrid | NA | | NA | DASS-21 | Financial issues were significantly associated with depression symptoms (B = .249, p < .001). | |  |
|  |  |  |  |  |  | |  |  |  | |  |

**Table 5 (Continued)**

*The Main Outcomes of the Studies Included in the Systematic Review*

| Study ID | Quality Assessment | SEC  Variable | Measurement  Method | Actual / Perceived | | Anxiety    Inventory Outcomes | | Depression    Inventory Outcomes | | Note | | | |
| --- | --- | --- | --- | --- | --- | --- | --- | --- | --- | --- | --- | --- | --- |
|  |  |  |  |  |  | |  |  |  | |  | | |
| #4908 | 8 | Education | 1-item binary variable (Yes/No) to assess whether they completed Year 12 | Actual | GAD-7 | | Non-significant | PHQ-9 | Non-significant | | |  | |
|  |  |  |  |  |  | |  |  |  | | |  | |
|  |  | Employment | Categorised as a) Unemployed, b) Student, c) Employed, d) Outside the labour market (homemaker/volunteer/retired) | Actual |  | | People involved in labour force (employed) showed lower odds of anxiety (OR = 0.67, p< .05) |  | Non-significant | | |  | |
|  |  |  |  |  |  | |  |  |  | | |  | |
|  |  | Housing Tenure | Categorized as a) Owners; b) Rented c) Other (homeless, staying with friends/family, other arrangements) | Actual |  | | Non-significant |  | Non-significant | | |  | |
|  |  |  |  |  |  | |  |  |  | | |  | |
|  |  | Household Income | Ranging from ‘No more than $300 per week’ to ‘More than $2700 per week) | Actual |  | | Non-significant |  | Non-significant | | |  | |
|  |  | Natural light | 1-item binary variable (Yes/No) to assess satisfaction with the amount of natural light in dwelling. | Perceived |  | | Non-significant |  | Natural light significantly predict depression (aOR = .64, p < .05), | | |  | |
|  |  | Internet space | 1-item binary variable (Yes/No) to assess Internet access in dwelling. | Actual |  | | Non-significant |  | Non-significant | | | |  |
|  |  | Outside access | 1-item binary variable (Yes/No) to assess whether one’s dwelling provided access to outside space for leisure. | Actual |  | | Non-significant |  | Non-significant | | | |  |
|  |  | Frequency of noise | Categorized as a) Never; b) Not very often; c) Fairly often; d) Very often to assess frequency bothered by noise while indoors at home. | Perceived |  | | Frequency of noise, significantly predicted higher anxiety (aOR = 1.18, p < .05). |  | Frequency of noise, significantly predicted higher depression (aOR = 1.30, p < .05). | | | |  |
|  |  |  |  |  |  | |  |  |  | | | |  |

**Table 5 (Continued)**

*The Main Outcomes of the Studies Included in the Systematic Review*

| Study ID | Quality Assessment | SEC  Variable | Measurement  Method | Actual / Perceived | | Anxiety    Inventory Outcomes | | Depression    Inventory Outcomes | | | Note | | | |
| --- | --- | --- | --- | --- | --- | --- | --- | --- | --- | --- | --- | --- | --- | --- |
|  |  |  |  |  |  | |  |  | |  | |  | | |
| #4660 | 8 | Neighbourhood noise level | 1-item variable measured on (1=Extremely quiet and 5 = Extremely noisy). | Perceived | K6 | | Psychological distress increases with the perception of neighbourhood noise level (B = 0.49, p < .05). | K6 | | Psychological distress increases with the perception of neighbourhood noise level (B = 0.49, p < .05). | |  | | |
|  |  |  |  |  |  | |  |  | |  | | |  | |
|  |  | Indoor sunshine level | 1-item variable measured on (1=Extremely little indoor sunshine and 5 = Extremely sufficient indoor sunshine). | Perceived |  | | Psychological distress decreases with perception of sunshine (B = -0.77, p < .01). | |  | Psychological distress decreases with perception of sunshine (B = -0.77, p < .01). | | |  | |
|  |  |  |  |  |  | |  |  | |  | | |  | |
|  |  | Indoor air quality level | 1-item variable measured on (1=Extremely bad indoor and 5 = Extremely good). | Perceived |  | | Psychological distress decreases with perception of indoor air quality (B = -0.67, p < .05). |  | | Psychological distress decreases with perception of indoor air quality (B = -0.67, p < .05). | |  | | |
|  |  |  |  |  |  | |  |  | |  | |  | | |
|  |  | Neighbourhood overall environment quality level | 1-item variable measured on (1=Extremely very poor and 5 = Extremely very good). | Perceived |  | | Non-significant |  | | Non-significant | | |  | |
|  |  |  |  |  |  | |  |  | |  | | |  | |
|  |  | Household monthly income (yuan) | Categorized as a) <= 3000; b) 3000-10000; c) 10000-20000; d) 20000-30000; e) 30000-50000; and f) >= 50000. | Actual |  | | Non-significant |  | | Non-significant | | |  | |
|  |  |  |  |  |  | |  |  | |  | | |  | |
| #4501 | 5 | Education | Categorized as a) Secondary or lower; b) Bachelor; and c) Postgraduate | Actual | GHQ-12 | | Non-significant | GHQ-12 | | Non-significant | | |  | |
|  |  |  |  |  |  | |  |  | |  | | |  | |
|  |  | Monthly income | Categorized as a) < 5000 SAR; b) 5000-10,000 SAR; c) > 10,000 SAR | Actual |  | | Non-significant |  | | Non-significant | | |  | |
|  |  |  |  |  |  | |  |  | |  | | |  | |
| #3940 | 6 | Financial losses | 1-item binary variable (Yes/No) to assess experience of financial losses of any kind due to COVID-19. | Perceived | NA | | NA | GAD-7 | | Non-significant | | |  | |
|  |  |  |  |  |  | |  |  | |  | | |  | |
| #3883 | 8 | Income | Categorized as a) <$45000; b) $45000-$74999; and c) >= $75000. | Actual | GAD-7 | | Low-income group had 1.65 times greater odds of a GAD-7 score >= 10 compared to those in the high-income group (p < .001). | PHQ-8 | | Low-income group had 1.38 times greater odds of a PHQ-8 score >=10 compared to those in the high-income group (p = .009). | | |  | |
|  |  |  |  |  |  | |  |  | |  | | |  | |
|  |  |  |  |  |  | |  |  | |  | | | |  |

**Table 5 (Continued)**

*The Main Outcomes of the Studies Included in the Systematic Review*

| Study ID | Quality Assessment | SEC  Variable | Measurement  Method | Actual / Perceived | | Anxiety    Inventory Outcomes | | Depression    Inventory Outcomes | | | Note | | |
| --- | --- | --- | --- | --- | --- | --- | --- | --- | --- | --- | --- | --- | --- |
|  |  |  |  |  |  | |  |  | |  | |  | |
| #3555 | 8 | Education | Categorized as a) Secondary level (6-10 grades); b) Primary level (1-5 grades); c) No formal education | Actual | NA | | NA | PHQ-9 | | Non-significant | |  | |
|  |  |  |  |  |  | |  |  | |  | | |  |
|  |  | Occupation | Categorized as a) Housewife; b) workers; c) Day labourer; d) Rickshaw puller; e) Small shop keeping; f) Jobless; g) Others | Actual |  | | NA | |  | Joblessness was associated with depression (β = .11, p = .043) | | |  |
|  |  |  |  |  |  | |  |  | |  | | |  |
|  |  | Monthly family income | Categorized as a) >10,000 BDT; b) <=10,000 BDT | Actual |  | | NA |  | | Non-significant | |  | |
|  |  |  |  |  |  | |  |  | |  | |  | |
|  |  | Job loss due to COVID-19 | 1-item binary variable (Yes/No) to assess whether respondent experienced job loss due to the COVID-19 pandemic | Actual |  | | NA |  | | Non-significant | | |  |
|  |  |  |  |  |  | |  |  | |  | | |  |
|  |  | Experiencing food scarcity due COVID-19 | 1-item binary variable (Yes/No) to assess whether respondent is experiencing food scarcity due to the COVID-19 pandemic | Perceived |  | | NA |  | | Non-significant | | |  |
|  |  |  |  |  |  | |  |  | |  | | |  |
| #3548 | 11 | Enough money to meet one’s needs | Categorised as a) Not at all/a little, b) Moderately, c) Mostly/completely | Perceived | HADS | | Not having enough money to meet one’s needs (OR = 3.18) was predictive of distress | HADS | | Not having enough money to meet one’s needs (OR = 3.18) was predictive of distress | | |  |
|  |  |  |  |  |  | |  |  | |  | | |  |
|  |  | Worry about soon not having enough money to meet one's needs | Unclear | Perceived |  | | Worrying about possibility of not having enough was predictive of distress (OR = 4.42) |  | | Worrying about possibility of not having enough was predictive of distress (OR = 4.42) | | |  |
|  |  |  |  |  |  | |  |  | |  | | |  |
| #3536 | 7 | Job/financial security stress | 3-items variable on 5-point Likert scale (1=“Extremely” and 5=“Not at all”) to assess COVID-related job/financial security stress: “To what extent do you feel you or your  family financial stability is at risk?” | Perceived | STAI | | Job/financial security stress was statistically significant and positively related to anxiety (β = .062, p = .030) | NA | | NA | | |  |
|  |  |  |  |  |  | |  |  | |  | | |  |

**Table 5 (Continued)**

*The Main Outcomes of the Studies Included in the Systematic Review*

| Study ID | Quality Assessment | SEC  Variable | Measurement  Method | Actual / Perceived | | Anxiety    Inventory Outcomes | | Depression    Inventory Outcomes | | Note | | |
| --- | --- | --- | --- | --- | --- | --- | --- | --- | --- | --- | --- | --- |
|  |  |  |  |  |  | |  |  |  | |  | |
| #3531 | 8 | Job or income loss and inability to make ends meet | Changes in employment status and in participants' "ability" to pay bills" and "ability to buy basic needs" since the pandemic began | Hybrid | NA | | NA | PHQ-9 | Increase of economic stressors since the pandemic began was positively and significantly associated with paternal mental health scores (β= 0.19, p < .05) | |  | |
|  |  |  |  |  |  | |  |  |  | | |  |
| #3451 | 8 | Education | Categorized as a) Primary; b) Secondary or higher | Actual | NA | | NA | PHQ-9 | Non-significant | | | Please see Table 4 for T2 |
|  |  |  |  |  |  | |  |  |  | | |  |
|  |  | Concern about work changes | 1-item assessed using 10-point Likert scale (0 = No concern and 10 = Maximum concern) categorized into a) Mild (0-4); b) Moderate (5-7); and c) Maximum (8-10): "How much you worry about job changes during quarantine?" | Perceived |  | | NA |  | This variable was used in T2 only | |  | |
|  |  |  |  |  |  | |  |  |  | |  | |
|  |  | Running out of money | 1-item assessed using 4-point Likert scale (1 = Not concerned and 4 = Very much concerned), grouped into a) No (1 and 2); and b) Yes (3 and 4): "Running out of money to pay expenses, rent and taxes. | Perceived |  | | NA |  | This variable was used in T2 only | | |  |
|  |  |  |  |  |  | |  |  |  | | |  |
|  |  |  |  |  |  | |  |  |  | | |  |
|  |  | Employment situation | Categorized as a) Employed; and b) Unemployed | Actual |  | | NA |  | This variable was used in T2 only | | |  |
|  |  |  |  |  |  | |  |  |  | | |  |
| #3366 | 8 | Economic Class | Individuals were classified into six economic class: A (45-100 points); B1 (38-44 points); B2 (29-37 points); C1 (23-28 points); C2 (17-22 points); D-E (0-16points) according to The Critério de Classificação Econômica Brasil. | Actual | GAD-7 | | Non-significant | NA | NA | | |  |
|  |  |  |  |  |  | |  |  |  | | |  |
|  |  |  |  |  |  | |  |  |  | | |  |

**Table 5 (Continued)**

*The Main Outcomes of the Studies Included in the Systematic Review*

| Study ID | Quality Assessment | SEC  Variable | Measurement  Method | Actual / Perceived | | Anxiety    Inventory Outcomes | | Depression    Inventory Outcomes | | | Note | | |
| --- | --- | --- | --- | --- | --- | --- | --- | --- | --- | --- | --- | --- | --- |
|  |  |  |  |  |  | |  |  | |  | |  | |
| #3309 | 8 | Education | Categorized as a) Diploma or less; b) Associate degree; c) Bachelor; d) Masters or Doctorate | Actual | STAI | | Non-significant | NA | | NA | |  | |
|  |  |  |  |  |  | |  |  | |  | | |  |
|  |  | Economic situation | Categorized as a) Good; b) Moderate; c) Poor | Perceived |  | | Non-significant |  | | NA | | |  |
|  |  |  |  |  |  | |  |  | |  | | |  |
| #3284 | 7 | Parents' years of schooling | In years | Actual |  | | Students whose fathers are more educated are less likely to feel anxiety. (B = -1.11 p =.001) |  | | NA | |  | |
|  |  |  |  |  |  | |  |  | |  | |  | |
|  |  | Monthly family income | Categorized as a) below 20000 BDT, b) 20000-30000, c)30000-45000, d) 45000-60000, e) above 60000 | Actual |  | | Non-significant |  | | NA | | |  |
|  |  |  |  |  |  | |  |  | |  | | |  |
|  |  |  |  |  |  | |  |  | |  | | |  |
|  |  |  |  |  |  | |  |  | |  | | |  |
| #3272 | 7 | Financial difficulties | 1-item variable measured on a 5-point Likert scale (1 = Not at all and 5 = To a very great extent): "Do you or your family currently experience financial difficulties due to the Coronavirus crisis [such as unemployment, reduced business activity and so on]?" | Perceived | BSI | | Greater financial difficulties was associated with greater psychological distress (β = .19, p < .001). | | BSI | Greater financial difficulties was associated with greater psychological distress (β = .19, p < .001). | | |  |
|  |  |  |  |  |  | |  | |  |  | | |  |
| #3266 | 8 | Education | Categorized as a) Diploma in nursing; and b) Graduate degree. | Actual | GAD-7 | | Lower education was associated with higher anxiety (OR = 22.247, p < .001). | NA | | NA | | |  |
|  |  |  |  |  |  | |  |  | |  | | |  |
|  |  | SES | Categorized as a) Lower middle class; and b) Upper middle class. | Perceived |  | | Non-significant |  | |  | | |  |
|  |  |  |  |  |  | |  |  | |  | | |  |

**Table 5 (Continued)**

*The Main Outcomes of the Studies Included in the Systematic Review*

| Study ID | Quality Assessment | SEC  Variable | Measurement  Method | Actual / Perceived | | Anxiety    Inventory Outcomes | | Depression    Inventory Outcomes | | | Note | | |
| --- | --- | --- | --- | --- | --- | --- | --- | --- | --- | --- | --- | --- | --- |
|  |  |  |  |  |  | |  |  | |  | |  | |
| #3238 | 7 | Family income loss due to COVID-19 | 1-item variable measured on a 5-point scale (1 = much higher and 5 = much lower) | Perceived | BAI | | No direct relationship | CES-D | | No direct relationship | |  | |
|  |  |  |  |  |  | |  |  | |  | | |  |
|  |  | Economic pressure due to Covid-19 | 1-item variable measured on a 5-point scale (1 = no pressure at all and 5 = a great deal of pressure) | Perceived |  | | No direct relationship |  | | No direct relationship | | |  |
|  |  |  |  |  |  | |  |  | |  | | |  |
|  |  | Family income | Categorized as a) below 30K yuan, b) 30 K to <80 K yuan c) 80 K to <150 K yuan d) 150 K to <350 K yuan e) 350 K to <800 K yuan f) 800 K to <2,000 K yuan g) 2000 K yuan and above | Actual |  | | No direct relationship |  | | No direct relationship | |  | |
|  |  |  |  |  |  | |  |  | |  | |  | |
| #3171 | 8 | Job insecurity stressors | 3-items inventory measured on a 5-point Likert scale (1 = Not at all and 5 = Extremely). E.g., "How concerned are you about Covid-19 causing a decrease in hours for your job?" | Perceived | PHQ-4 | | Non-significant | PHQ-4 | | Non-significant | | |  |
|  |  |  |  |  |  | |  |  | |  | | |  |
|  |  | Financial stressors | 3-items inventory measured on a 5-point Likert scale (1 = Not at all and 5 = Extremely). E.g., "How concerned are you about Covid-19 affecting your ability to pay rent?" | Perceived |  | | Financial stressors were associated with higher anxiety (β = .15, p < .01). |  | | Financial stressors were associated with higher depression (β = .15, p < .01). | | |  |
|  |  |  |  |  |  | |  |  | |  | | |  |
| #3059 | 8 | Education | Academic degree or not | Actual | MHI-5 | | Not having an academic degree contributed to higher psychological distress (β = -.08, p < .01). | | MHI-5 | Not having an academic degree contributed to higher psychological distress (β = -.08, p < .01). | | |  |
|  |  |  |  |  |  | |  | |  |  | | |  |
|  |  | Economic status | Categorized as a) Below average; b) Average; and c) Above average. | Perceived |  | | Non-significant |  | | Non-significant | | |  |
|  |  |  |  |  |  | |  |  | |  | | |  |
|  |  |  |  |  |  | |  |  | |  | | |  |
|  |  |  |  |  |  | |  |  | |  | | |  |

**Table 5 (Continued)**

*The Main Outcomes of the Studies Included in the Systematic Review*

| Study ID | Quality Assessment | SEC  Variable | Measurement  Method | Actual / Perceived | | Anxiety    Inventory Outcomes | | Depression    Inventory Outcomes | | | Note | | |
| --- | --- | --- | --- | --- | --- | --- | --- | --- | --- | --- | --- | --- | --- |
|  |  |  |  |  |  | |  |  | |  | |  | |
| #3051 | 8 | Education | Categorized as a) Below diploma or 12 years of education; b) Diploma with 12 years of education; c) Student or graduate of a 2-year college degree; d) student or graduate of a 4-year college degree; e) student or graduate of a master degree; and f) student or graduate of doctoral degree | Actual | GAD-2 | | Non-significant | PHQ-4 | | Non-significant | |  | |
|  |  |  |  |  |  | |  |  | |  | | |  |
| #3039 | 8 | Household income | CRISIS questionnaire (reported by parents) | Actual | CRISIS | | Non-significant | CRISIS | | Non-significant | | |  |
|  |  |  |  |  |  | |  |  | |  | | |  |
|  |  | Economic impact of COVID-19 | CRISIS questionnaire (based on 2-items in CRISIS reported by parents) | Perceived |  | | Improvement in anxiety was associated with greater economic concerns (OR = 5.57, p = 0.006) |  | | Non-significant | |  | |
|  |  |  |  |  |  | |  |  | |  | |  | |
| #3035 | 7 | Yearly income | Categorized as (renminbi/per year) a) <50,000; b) 50,000-100,000; c) 110,000-150,000; d) 160,000-200,000; e) 210,000-300,000; f) 310,000-400,000; g) >400,000 | Actual | GAD-7 | | Non-significant | PHQ-9 | | Non-significant | | |  |
|  |  |  |  |  |  | |  |  | |  | | |  |
| #3006 | 11 | Education | Categorized as a) High school; b) High school graduate; c) Vocational/tech school/some college; d) Bachelor's degree or more | Actual | NA | | NA | PHQ-9 | | Non-significant | | | Please see Table 4 for T2 |
|  |  |  |  |  |  | |  |  | |  | | |  |
|  |  | Household income | Categorized as a) <=19,999, b) 20,000-44,999 c) 45,000-74,999 d) >=75,000 | Actual |  | | NA |  | | Having $19,999 or less in household income was associated with 2.3 times the odds of depression relative to  incomes of $75,000 or more (p = .009) | | |  |
|  |  |  |  |  |  | |  | |  |  | | |  |
|  |  | Household savings | Categorised as a) <=4,999, b) >5,000 | Actual |  | | NA |  | | Persons with less than  $5,000 in household savings were more likely to report depressive symptoms (OR = 1.5, p =.035) | | |  |

**Table 5 (Continued)**

*The Main Outcomes of the Studies Included in the Systematic Review*

| Study ID | Quality Assessment | SEC  Variable | Measurement  Method | Actual / Perceived | | Anxiety    Inventory Outcomes | | Depression    Inventory Outcomes | | | Note | | |
| --- | --- | --- | --- | --- | --- | --- | --- | --- | --- | --- | --- | --- | --- |
|  |  |  |  |  |  | |  |  | |  | |  | |
| #2944 | 8 | Education | Categorized as a) Low education; b) Medium education; c) High education | Perceived | NA | | NA | CESD-10 | | Low education was associated with higher depression (B = 1.40, p < .01) as compared to Medium education. High education, however did not differ significantly in depression compared to Medium education. | | |  |
|  |  |  |  |  |  | |  |  | |  | | |  |
|  |  | Employment | Categorized as a) Retired; b) Unemployed; and c) Employed | Actual |  | | NA |  | | Retired was associated with lower depression (B = -0.41, p < .05) as compared to employed. But unemployed did not differ significantly in depression compared to employed. | |  | |
|  |  |  |  |  |  | |  |  | |  | |  | |
|  |  | Household income (Euro) | Open-ended question | Actual |  | | NA |  | | Non-significant | | |  |
|  |  |  |  |  |  | |  |  | |  | | |  |
| #2849 | 10 | Years of education | Number of years of formal education | Actual | NA | | NA | CESD-20 | | Years of education was negatively related to depression (β = -.023, p < .01). | | |  |
|  |  |  |  |  |  | |  |  | |  | | |  |
|  |  | Income changes | Dummy coded as 1 = Furlough, decreased income, 0 = No Change | Actual |  | | NA |  | | Furlough/decreased income was associated with higher depression (β = .16, p < .05). | | |  |
|  |  |  |  |  |  | |  | |  |  | | |  |
| #2837 | 7 | Job loss | Job loss / No job loss | Actual | DASS-21 | | Participants with job loss were more anxious (F= 21.23, p < .001) | DASS-21 | | Participants with job loss were more depressed (F= 14.67, p <0.001) | | |  |
|  |  |  |  |  |  | |  |  | |  | | |  |
|  |  | Education | Categorized as a) Less than high school; b) High school of equivalent, c) Associate, d) Undergraduate, e) Graduate or above | Actual |  | | Higher education was significantly associated with lower anxiety (β = -.12, p < .01) |  | | Higher education was significantly associated with lower depression (β = -.15, p < .01) | | |  |
|  |  |  |  |  |  | |  |  | |  | | |  |
|  |  |  |  |  |  | |  |  | |  | | |  |

**Table 5 (Continued)**

*The Main Outcomes of the Studies Included in the Systematic Review*

| Study ID | Quality Assessment | SEC  Variable | Measurement  Method | Actual / Perceived | | Anxiety    Inventory Outcomes | | Depression    Inventory Outcomes | | | Note | | |
| --- | --- | --- | --- | --- | --- | --- | --- | --- | --- | --- | --- | --- | --- |
|  |  |  |  |  |  | |  |  | |  | |  | |
| #2832 | 11 | Education | Having college or not | Actual | DASS-21 | | Having college degree was associated with lower depression (OR = 0.52, p < .001). | DASS-21 | | Having college degree was associated with lower depression (OR = 0.41, p = .001). | |  | |
|  |  |  |  |  |  | |  |  | |  | | |  |
|  |  | Concerns about income | 3-items inventory (e.g., ""Are you concerned about an income decrease in the next few months?"") measured on 3-point scale (1 = Mildly concerned and 3 = Very concerned) , which were summed up and then dichotomized; the higher scores (>=8) were defined  as having ""Concerns about income" | Perceived |  | | Concerns about income was associated with higher anxiety (OR = 2.03, p < .001). |  | | Non-significant | | |  |
|  |  |  |  |  |  | |  |  | |  | | |  |
| #2821 | 8 | Lost income source/employment | 1-item binary variable (Yes/No). | Actual | DASS-21 | | Non-significant | DASS-21 | | Non-significant | |  | |
|  |  |  |  |  |  | |  |  | |  | |  | |
|  |  | Economic constrain | 1-item binary variable (Yes/No). | Perceived |  | | Economic constrain significantly associated with anxiety (aOR = 3.111, p < .001). |  | | Economic constrain significantly associated with depression (aOR = 3.415, p < .001). | | |  |
|  |  |  |  |  |  | |  |  | |  | | |  |
|  |  | Financial threat | Financial Threat Scale | Perceived |  | | Financial threat was positively associated with anxiety (β = .29, p < .001) |  | | Financial threat was positively associated with depression (β = .27, p < .001) | | |  |
|  |  |  |  |  |  | |  |  | |  | | |  |
|  |  | Financial wellbeing | Financial Wellbeing Scale | Perceived |  | | Financial wellbeing was negatively associated with anxiety (β = .24, p<0.001). |  | | Financial wellbeing was negatively associated with depression (β = .27, p < .001). | | |  |
|  |  |  |  |  |  | |  | |  |  | | |  |
|  |  | Economic hardship | Economic Hardship Scale | Actual |  | | Non-significant |  | | Non-significant | | |  |
|  |  |  |  |  |  | |  |  | |  | | |  |
| #2800 | 8 | Education | Years of education completed | Actual | BAI | | Non-significant | NA | | NA | | |  |
|  |  |  |  |  |  | |  |  | |  | | |  |
|  |  | SES | MacArthur Subjective SES scale | Perceived |  | | Increase in anxiety predicted lower levels of SES (p < .05). |  | | NA | | |  |
|  |  |  |  |  |  | |  |  | |  | | |  |
|  |  | Lost job | Yes/No | Actual |  | | Non-significant |  | | NA | | |  |
|  |  |  |  |  |  | |  |  | |  | | |  |

**Table 5 (Continued)**

*The Main Outcomes of the Studies Included in the Systematic Review*

| Study ID | Quality Assessment | SEC  Variable | Measurement  Method | Actual / Perceived | | Anxiety    Inventory Outcomes | | Depression    Inventory Outcomes | | | Note | | |
| --- | --- | --- | --- | --- | --- | --- | --- | --- | --- | --- | --- | --- | --- |
|  |  |  |  |  |  | |  |  | |  | |  | |
| #2795 | 7 | Degree of family economic status influenced by COVID-19 | Measured on 5-point Likert scale (1 = Little and 5 = Much) | Perceived | SAS | | Rating the degree of family economic status influenced by COVID-19 as 2 (OR=4.56, p=<.001), 4 (OR=4.70, p=<.001), and 5 (OR=4.35, p=.002) is associated with anxiety | PHQ-9 | | Rating the degree of family economic status influenced by COVID-19 as 2 (OR=1.86, p=.003), 4 (OR=3.28, p=<.001), and 5 (OR=2.26, p=.006) is associated with depression | |  | |
|  |  |  |  |  |  | |  |  | |  | | |  |
| #2792 | 8 | Education | Categorized as a) High school or less; b) Some post-high school education; c) University or college; and d) Prefer not to answer. | Actual | GAD-7 | | The importance of education in predicting emotional distress was low. | CESD-3 | | The importance of education in predicting emotional distress was low. | | |  |
|  |  |  |  |  |  | |  |  | |  | | |  |
|  |  | Household income (CAD$) | Categorized as a) Less than 40000; b) 40000-79000; c) 80000-119000; d) 120000 or more; and e) Prefer not to answer | Actual |  | | The importance of household income in predicting emotional distress was low. |  | | The importance of household income in predicting emotional distress was low. | |  | |
|  |  |  |  |  |  | |  |  | |  | |  | |
|  |  | Financial worry | 1-item variable measured on 4-point Likert scale (1 = Not at all worried and 4 = Very worried): "How worried are you about the impact of COVID on your financial situation?" | Perceived |  | | Financial worry was the most important variable to predict emotional distress. Low to moderate levels of worry were associated with decreased or average predicted distress, while severe worries increased predicted distress (0.41 for “very worried” about finances) |  | | Financial worry was the most important variable to predict emotional distress. Low to moderate levels of worry were associated with decreased or average predicted distress, while severe worries increased predicted distress (0.41 for “very worried” about finances) | | |  |
|  |  |  |  |  |  | |  |  | |  | | |  |
| #2783 | 8 | Financial worries in Covid-19 | Measured on a 5- point Likert scale: (1= Not worried and 5 = Very worried) | Perceived | GAD-7 | | COVID-19 related stressors (financial situation) significantly predicted anxiety (β = .047, p = .011) | PHQ-8 | | COVID-19 related stressors (the financial situation) significantly predicted depression (β = .065, p = .001) | | |  |
|  |  |  |  |  |  | |  |  | |  | | |  |
| #2780 | 8 | Socio-professional | Categorized as a) Executives and higher intellectual professions; b) Trade people; c) Non-professional; d) Employees; e) Primary/secondary students; f) Manual workers; g) Intermediate profession; and h) Retired. | Actual | GAD-7 | | Not working (unemployed or students) was associated with greater vulnerability to anxiety (Unemployed: aOR = 1.79, p = .010; Student: aOR = 1.68; p = .005) | MDI | | Farmers (aOR = 6.11, p = .018), unemployed people (aOR = 2.58, p < .001), students (aOR = 2.24, p = .011) and manual workers (aOR = 2.63, p = .041) were found to have greater vulnerability to depression. | | |  |
|  |  |  |  |  |  | |  | |  |  | | |  |

**Table 5 (Continued)**

*The Main Outcomes of the Studies Included in the Systematic Review*

| Study ID | Quality Assessment | SEC  Variable | Measurement  Method | Actual / Perceived | | Anxiety    Inventory Outcomes | | Depression    Inventory Outcomes | | Note | | |
| --- | --- | --- | --- | --- | --- | --- | --- | --- | --- | --- | --- | --- |
|  |  |  |  |  |  | |  |  |  | |  | |
| #2739 | 8 | Education | Categorized as a) Elementary/High School; b) College; c) University | Actual | NA | | NA | InterRAI | Non-significant | |  | |
|  |  |  |  |  |  | |  |  |  | | |  |
|  |  | Financial status -difficulty making ends meet | Two comparison: a) Before and after Covid-19 vs. None; and b) Before or after Covid-19 vs. None. | Perceived |  | | NA |  | The odds were 1.93 times higher in people that had financial concerns before and after the pandemic than people without financial concerns (P < .001). | | |  |
|  |  |  |  |  |  | |  |  |  | | |  |
| #2667 | 10 | Income | Categorized as a) Income less than $50,000; and b) Income of $50,000 or more. | Actual | NA | | NA | PHQ-2 | Non-significant | |  | |
|  |  |  |  |  |  | |  |  |  | |  | |
|  |  | Education | College / No college | Actual |  | | NA |  | Non-significant | | |  |
|  |  |  |  |  |  | |  |  |  | | |  |
|  |  | Loss of Job/Income | An indicator for whether the respondent reported losing his/her job or a substantial portion of income as a result of COVID-19. | Actual |  | | NA |  | Loss of job/income significantly predicted increase in depressive symptoms (β = 0.093) | | |  |
|  |  |  |  |  |  | |  |  |  | | |  |

**Table 5 (Continued)**

*The Main Outcomes of the Studies Included in the Systematic Review*

| Study ID | Quality Assessment | SEC  Variable | Measurement  Method | Actual / Perceived | | Anxiety    Inventory Outcomes | | Depression    Inventory Outcomes | | | Note | | |
| --- | --- | --- | --- | --- | --- | --- | --- | --- | --- | --- | --- | --- | --- |
|  |  |  |  |  |  | |  |  | |  | |  | |
| #2625 | 8 | Education | Categorized as a) Elementary; b) Preparatory, c) Secondary/diploma; d) Bachelor/higher degree; e) Post graduate (master, PhD); f) other | Actual | GAD-7 | | Respondents with a higher educational level were more likely to exhibit anxiety symptoms. | PHQ-2 | | A higher education level was associated with a higher likelihood of depressive symptoms, and bachelor degree holders were 2.17 times more likely to exhibit depressive symptoms, whilst post-graduate degree holders or students were 2.42 times more likely to exhibit depressive symptoms. | | |  |
|  |  | Occupation | Categorized as a) full-time employee; b) part-time employee; c) student; d) unemployed; e) retired; f) other | Actual |  | | Full-time employee during pandemic seems to associate with higher anxiety: Category B: OR= .71, p=<.001; Category C: OR= .69, p==.001; Category D: OR= .78, p= =.004;Category E: OR= .53, p= =.035" | |  | Full-time employee during pandemic seems to associate with higher depression: Category B: OR= .74, p= <.001; Category C: OR= .72, p= <.001; Category E: OR= .30, p= <.001" | | |  |
|  |  |  |  |  |  | |  | |  |  | | |  |
|  |  | Financial status | Categorized as a) Stable income; b) Non-stable income | Perceived |  | | Non-significant | |  | Non-significant | | |  |
|  |  |  |  |  |  | |  | |  |  | | |  |
|  |  | Financial issue during Covid-19 | 1-item binary variable (Yes/No) to assess whether participants were facing financial difficulties due to the impact of Covid-19. | Perceived |  | | Having financial issues during the COVID-19 pandemic had 1.33 times higher odds to exhibit anxiety symptoms (p =<.001) | |  | Having financial issues during the COVID-19 pandemic had 1.52 higher odds to exhibit depressive symptoms (p =<.001)  " | | |  |
|  |  |  |  |  |  | |  | |  |  | | |  |
| #2593 | 8 | Education | Categorized as a) High school; b) College; c) Bachelor; and d) Postgraduate. | Actual | GHQ-28 | | Graduate (B = 13.18), undergraduate (B = 6.45) and college (B = 1.61) qualifications were predictive of higher anxiety. | | GHQ-28 | Graduate (B = 4.84), undergraduate (B = 3.02) and college (B = 0.16) qualifications were predictive of severe depression | | |  |
|  |  | Work | Working or not | Actual |  | | Non-significant | |  | Non-significant | | |  |
|  |  |  |  |  |  | |  | |  |  | | |  |
| #2590 | 8 | Facing financial problem | 1-item binary variable (Yes/No) to assess whether participants were facing financial difficulties due to the impact of Covid-19. | Perceived | GAD-2 | | Non-significant | | PHQ-9 | Facing financial problem was significant risk factor for depression (aOR = 2.196, p = .005). | | |  |
|  |  |  |  |  |  | |  | |  |  | | |  |

**Table 5 (Continued)**

*The Main Outcomes of the Studies Included in the Systematic Review*

| Study ID | Quality Assessment | SEC  Variable | Measurement  Method | Actual / Perceived | | Anxiety    Inventory Outcomes | | Depression    Inventory Outcomes | | | Note | | |
| --- | --- | --- | --- | --- | --- | --- | --- | --- | --- | --- | --- | --- | --- |
|  |  |  |  |  |  | |  |  | |  | |  | |
| #2468 | 8 | Education | Categorized as a) Undergraduate; b) Junior college; c) High school; d) Graduate | Actual | GAD-7 | | Non-significant | | PHQ-9 | Compared with participants with a high school diploma, those with a college degree appeared to be vulnerable to depression disorders (aOR = 2.157; p < .001) | | |  |
|  |  |  |  |  |  | |  | |  |  | | |  |
|  |  | Working years | Categorized as a) 10-20 years, b) 5-10 years, c) >=5 years, d) >=20 years. | Actual |  | | Compared with participants with fewer years of work experience, those with longer years of work experience were associated with severe symptoms of anxiety (aOR = 0.755 ; p = 0.032) | |  | Compared with participants with fewer years of work experience, those with longer years of work experience were associated with severe symptoms of depression (aOR = 0.616; p = 0.046) | | |  |
|  |  |  |  |  |  | |  | |  |  | | |  |
| #2325 | 8 | Education | High school graduated or University graduated. | Actual | DASS-21 | | University graduates were 70% less likely to get anxiety (aOR = 0.3, p = .03). | | DASS-21 | University graduates were less likely to get depression by 77% (aOR = 0.23, p = .003) | | |  |
|  |  |  |  |  |  | |  | |  |  | | |  |
|  |  | Compensation from company | Yes/No | Actual |  | | Non-significant | |  | Non-significant | | |  |
|  |  |  |  |  |  | |  | |  |  | | |  |
| #2324 | 8 | Education | Categorized as a) University; b) Middle school; c) Primary school. | Actual | SAS | | Education level was significantly associated with anxiety (β = -.219, p < .001) for SCA sample. | | SDS | Education level was significantly associated with depression (β = -.214, p < .001) for SCA sample. | | |  |
|  |  |  |  |  |  | |  | |  |  | | |  |
| #2289 | 8 | Housing condition | Categorized as a) House without balcony, terrace, or garden during lockdown; b) Home with balcony or terrace; and c) Home with garden. | Actual | GAD-7 | | Home with no terrace or balcony during lockdown was associated with higher anxiety (p = .005) | | PHQ-9 | Home with no terrace or balcony during lockdown was associated with higher depression (p = .005). | | |  |
|  |  |  |  |  |  | |  | |  |  | | |  |

**Table 5 (Continued)**

*The Main Outcomes of the Studies Included in the Systematic Review*

| Study ID | Quality Assessment | SEC  Variable | Measurement  Method | Actual / Perceived | | Anxiety    Inventory Outcomes | | Depression    Inventory Outcomes | | | | Note | | |
| --- | --- | --- | --- | --- | --- | --- | --- | --- | --- | --- | --- | --- | --- | --- |
|  |  |  |  |  |  | |  |  | |  | | |  | |
| #2131 | 11 | Financial impact | Assessed using an item  from the COVID-19 Adolescent Symptom and Psychological Experience Questionnaire. The item asked youth to indicate if their family had experienced any of the indices of financial difficulties listed, including job loss by one or more adults, difficulty paying bills or buying necessities, adult filing for unemployment, and adult applying for public assistance. | Actual | MASC | | Non-significant | | CDI | Non-significant | | | |  |
|  |  |  |  |  |  | |  | |  | |  | | |  |
| #2088 | 7 | Years of education | Number of years of completed education | Actual | Neuro-QoL | | More years of education was associated with lower anxiety (AOR = 0.92, p = .009). | | Neuro-QoL | | Non-significant | | |  |
|  |  |  |  |  |  | |  | |  |  | | | |  |
|  |  | Unemployment | Unclear | Actual |  | | Non-significant | |  | Unemployment was associated with higher depression (AOR = 1.75, p = .012) | | | |  |
|  |  |  |  |  |  | |  | |  |  | | | |  |
|  |  | Food insecurity | Unclear | Unclear |  | | Non-significant | |  | Non-significant | | | |  |
|  |  |  |  |  |  | |  | |  |  | | | |  |
| #2045 | 8 | Traumatic economic stress (TES) | 4-items on 5-point Likert scale (1 = not at all and 5 = very much): E.g., "The Coronavirus (COVID-19) has impacted me negatively from a financial point of view". | Perceived | GAD-7 | | Traumatic economic stress significantly predict anxiety (β = .17; p = .008) | | PHQ-9 | Non-significant | | | |  |
|  |  |  |  |  |  | |  | |  |  | | | |  |
| #1676 | 8 | Socioeconomic position (SEP) | Measured using SEIFA quintiles (1 = Lowest and 5 = Highest), which was derived from postcode | Actual | GAD-7 | | Higher SEP was associated with lower odds of anxiety in women aged 18-49 (quintile 3: OR = 0.73; quintile 5: 0.72), but not in women 50 and above, and in men. | | PHQ-9 | Higher SEP was associated with lower odds of anxiety in women aged 18-49 (quintile 3: OR = 0.66; quintile 5: 0.62) and in women 50 years and above, (quintile 5: OR = 0.71) but not in men. | | | |  |
|  |  |  |  |  |  | |  | |  |  | | | |  |
|  |  | Lost job | 1-item binary variable (Yes/No) to assess whether participants experienced job loss. | Actual |  | | Losing a job was associated with higher odds of anxiety in women aged 50 years and above (OR = 1.56), but not in younger women or men. | |  | Losing a job was associated with increased depression risk for both sexes and all age groups (women 18-49: aOR = 1.51; women 50 and above: aOr = 1.81; men 18-49: aOR = 1.65; men 50 and above: aOR = 1.69) | | | |  |

**Table 5 (Continued)**

*The Main Outcomes of the Studies Included in the Systematic Review*

| Study ID | Quality Assessment | SEC  Variable | Measurement  Method | Actual / Perceived | | Anxiety    Inventory Outcomes | | Depression    Inventory Outcomes | | | | Note | | |
| --- | --- | --- | --- | --- | --- | --- | --- | --- | --- | --- | --- | --- | --- | --- |
|  |  |  |  |  |  | |  |  | |  | | |  | |
| #1674 | 10 | Financial hardship | 1-item binary variable (Yes/No) to assess whether the family of the patient indicated that they experienced financial hardship during the lockdown period. | Perceived | CBCL | | Non-significant | | NA | NA | | | |  |
|  |  |  |  |  |  | |  | |  | |  | | |  |
| #1626 | 7 | Living area | Categorized as a) Rural; b) Urban | Actual | GAD-7 | | Non-significant | | NA | | NA | |  | |
|  |  |  |  |  |  | |  | |  |  | | | |  |
|  |  | Family income decreased | 1-item binary variable (Yes/No) to assess whether the respondent's family income had decreased due to COVID-19 | Perceived |  | | Decreased family income due to COVID-19 was associated with higher anxiety (OR = 1.71, p < .001) | |  | NA | | | |  |
|  |  |  |  |  |  | |  | |  |  | | | |  |
| #1462 | 7 | Health care insurance | Categorized as a) Full coverage, b) Partial coverage, c) Medicare, d) No insurance | Actual | GAD-7 | | Individuals who were on Medicare/Medicaid and individuals with no coverage were 1.97 and 4.48 times more likely to report moderate or severe anxiety compared to individuals with full coverage, respectively (OR= 1.97, P=.03; OR = 4.48, P =.01). | | PHQ-2 | Individuals who had partial coverage and individuals with no coverage were 2.67 and 3.22 times more likely to report depressive symptomology compared to individuals with full coverage, respectively (OR = 2.67, P =.02; OR= 3.22, P =.01) | | | |  |
|  |  |  |  |  |  | |  | |  |  | | | |  |
|  |  | Work status | Categorized as a) Remote before and after COVID, b) Unemployed prior to COVID, c) Work outside home, d) No longer working due to COVID, e) Working remotely due to COVID | Actual |  | | Non-significant | |  | Individuals who were no longer working due to COVID-19 were 2.25 times more likely to report symptoms of depression compared to individuals who were working remotely before and after COVID (“no change” group; OR = 2.25, P =.02). | | | |  |
|  |  |  |  |  |  | |  | |  |  | | | |  |
|  |  | Financial concern | Unclear | Unclear |  | | Financial concerns were significantly related to higher anxiety (OR = 1.32, p < .01) | |  | Financial concerns was significantly related to higher depression (OR = 1.49, p <.001) | | | |  |
|  |  |  |  |  |  | |  | |  |  | | | |  |
|  |  | Economy-related concern | Unclear | Unclear |  | | Non-significant | |  | Non-significant | | | |  |
|  |  |  |  |  |  | |  | |  |  | | | |  |
|  |  | Food access concern | Unclear | Unclear |  | | Food access concerns were significantly related to higher anxiety (OR = 1.39, p < .001) | |  | Food access concerns was significantly related to higher depression (OR = 1.29, p < .01) | | | |  |
|  |  |  |  |  |  | |  | |  |  | | | |  |

**Table 5 (Continued)**

*The Main Outcomes of the Studies Included in the Systematic Review*

| Study ID | Quality Assessment | SEC  Variable | Measurement  Method | Actual / Perceived | | Anxiety    Inventory Outcomes | | Depression    Inventory Outcomes | | | | | Note | | |
| --- | --- | --- | --- | --- | --- | --- | --- | --- | --- | --- | --- | --- | --- | --- | --- |
|  |  |  |  |  |  | |  |  | |  | | | |  | |
| #1447 | 7 | Financial condition | Categorized as a) Steady; b) Not steady. | Perceived | GAD-7 | | Unstable financial condition was associated with higher anxiety (OR = 2.92, p < .001). | | NA | NA | | | | |  |
|  |  |  |  |  |  | |  | |  | |  | | | |  |
| #1389 | 7 | Family status | Categorized as a) Poor; b) Middle class; c) Upper middle class | Perceived | GAD-7 | | Poor family status was risk factor for anxiety (OR = 34.11, p = .008) | | NA | NA | | | |  | |
|  |  |  |  |  |  | |  | |  |  | | | | |  |
|  |  | Steady family income | 1-item binary variable (Yes/No) to assess whether family income is steady | Perceived |  | | Non-significant | |  | NA | | | | |  |
|  |  |  |  |  |  | |  | |  |  | | | | |  |
| #1387 | 8 | Decreased family income in quarantine | 1-item binary variable (Yes/No) to assess whether family income is decreased | Perceived | NA | | NA | | PHQ-9 | Non-significant | | | | |  |
|  |  |  |  |  |  | |  | |  |  | | | | |  |
|  |  | Occupation | Categorized as a) Study and work; and b) Only study. | Actual |  | | NA | |  | Non-significant | | | | |  |
|  |  |  |  |  |  | |  | |  |  | | | | |  |
| #1359 | 8 | SES | Categorized into a) Lower, b) Middle and c) Upper based on monthly family income | Perceived | NA | | NA | | PHQ-9 | Lower (aOR = 2.22) and Middle (aOR = 1.82) SES significantly associated with more severe depression symptoms compared to Upper SES | | | | |  |
|  |  |  |  |  |  | |  | |  |  | | | | |  |
| #1345 | 7 | Economy | Worries over the financial situation due to Covid-19 | Perceived | HSCL-10 | | Worries over the financial situation due to COVID-19 were associated with significant increased risks of anxiety (OR=1.65) | | HSCL-10 | Worries over the financial situation due to COVID-19 were associated with significant increased risks of depression (OR=1.80) | | | | |  |
|  |  |  |  |  |  | |  | |  |  | | | | |  |
| #1339 | 8 | SES | Categorized as a) Poor; b) Good; c) Very Good; and d) Excellent | Perceived | AV-MHI-38 | | SES significantly predict psychological distress (β= -0.17, p < .001) | | AV-MHI-38 | | | SES significantly predict psychological distress (β = -0.17, p < .001) | | |  |
|  |  |  |  |  |  | |  | |  |  | | | | |  |

**Table 5 (Continued)**

*The Main Outcomes of the Studies Included in the Systematic Review*

| Study ID | Quality Assessment | SEC  Variable | Measurement  Method | Actual / Perceived | | Anxiety    Inventory Outcomes | | Depression    Inventory Outcomes | | | Note | | |
| --- | --- | --- | --- | --- | --- | --- | --- | --- | --- | --- | --- | --- | --- |
|  |  |  |  |  |  | |  |  | |  | |  | |
| #1284 | 8 | Education | Categorized as a) Primary; b) Middle school; c) High school; d) University. | Actual | GAD-7 | | Non-significant | | NA | NA | | |  |
|  |  |  |  |  |  | |  | |  |  | | |  |
|  |  | Farm income | Categorized as a) Low; b) Intermediate; c) High, in comparison to other farmers in the same village. | Perceived |  | | Anxiety was higher in high-income farmers than in other farmers | |  | NA | | |  |
|  |  |  |  |  |  | |  | |  |  | | |  |
|  |  | Land size (Hectares) | Categorized as a) 0-5; b) 5-10; c) 10-15; d) 15-30; and e) > 30. | Actual |  | | Anxiety levels of farmers with a land of 30 hectares or less were higher than those of farmers with a land of more than 30 hectares. | |  | NA | | |  |
|  |  |  |  |  |  | |  | |  |  | | |  |
|  |  | Use of a tractor | Yes / No | Actual |  | | Non-significant | |  | NA | | |  |
|  |  |  |  |  |  | |  | |  |  | | |  |
|  |  | Truck use | Yes / No | Actual |  | | Anxiety was higher in truck farmers (OR = 2.117, p < .001) | |  | NA | | |  |
|  |  |  |  |  |  | |  | |  |  | | |  |
| #1251 | 8 | Education | Categorized as a) Primary school or below; b) Middle school or above | Actual | K-10 | | Patients who had a middle school education or above were more likely to stay away from psychological distress (OR =.521, p =.041) | | K-10 | Patients who had a middle school education or above were more likely to have less psychological distress (OR=.521, p=.041) | | |  |
|  |  |  |  |  |  | |  | |  |  | | |  |
|  |  | Economic burden | Categorized as a) Low; b) High | Perceived |  | | Patients with a high economic burden were more likely to have psychological distress (OR = 1.697, p = .044) | |  | Patients with a high economic burden were more likely to have psychological distress (OR = 1.697, p = .044) | | |  |
|  |  |  |  |  |  | |  | |  |  | | |  |
| #1226 | 7 | Education | Categorized as a) Middle school; b) High school; c) College/university; d) Postgraduate | Actual | GAD-7 | | Non-significant | | PHQ-9 | Non-significant | | |  |
|  |  |  |  |  |  | |  | |  |  | | |  |

**Table 5 (Continued)**

*The Main Outcomes of the Studies Included in the Systematic Review*

| Study ID | Quality Assessment | SEC  Variable | Measurement  Method | Actual / Perceived | | Anxiety    Inventory Outcomes | | Depression    Inventory Outcomes | | | Note | | |
| --- | --- | --- | --- | --- | --- | --- | --- | --- | --- | --- | --- | --- | --- |
|  |  |  |  |  |  | |  |  | |  | |  | |
| #1080 | 5 | Education | Categorized as a) Secondary; b) Graduate; c) Master; d) Doctorate | Actual | BAI | | Only doctorate level was significantly associated with lower anxiety (B = -2.282, p < .05). | | BDI-II | Non-significant. | | |  |
|  |  |  |  |  |  | |  | |  |  | | |  |
|  |  | Employment | Categorized as a) Employed; b) Unemployed; c) Retired; and d) Student | Actual |  | | Unemployed or student status was associated with higher anxiety (B = 2.891, p < .05; B = 5.288, p < .01). | |  | Student status (but not unemployment) was associated with higher depression (B = 4.738, p < .01). | | |  |
|  |  |  |  |  |  | |  | |  |  | | |  |
|  |  | SES | Assessed using the Arabic version of the socioeconomic scale, which consisted of four dimensions, namely, level of education, employment, total family monthly income, and lifestyle of the family. | Actual |  | | Low SES was associated with higher anxiety (B = 2.773, p < .01). But high SES was not significantly associated with lower anxiety | |  | Low SES was associated with higher depression (B = 1.725, p < .05) and high SES was associated with lower depression (B = -2.006, p < .05). | | |  |
|  |  |  |  |  |  | |  | |  |  | | |  |
| #1077 | 7 | Education | Categorized as a) Secondary; b) University degree; c) postgraduate; d) PhD; e) Other | Actual | HADS | | Postgraduate degree (B =- 0.928, p = .002) and PhD degree (B = -1.570, p = .002) negatively predicted anxiety. | | HADS | Postgraduate degree (B = -0.915, p = .001) and PhD degree (B = -1.298, p = .006) emerged as significant predictors of lower depression | | |  |
| #1023 | 8 | Income (family monthly) | Categorized as a) Less than 500 JD; b) 500-1000 JD; c) More than 1000 JD; d) More than 2000 JD; and e) More than 3000 JD. | Actual | HADS | | Lower family monthly incomes predict higher anxiety (β = -.099, p = .039). | | HADS | Non-significant | | |  |
|  |  |  |  |  |  | |  | |  |  | | |  |
| #993 | 8 | Education | Categorized as a) Less than University; b) University; and c) Graduate degree. | Actual | GAD-7 | | Non-significant | | CESD-7 | Education level of “Less than University”, “University” (OR = 1.48, p = .027) and “Graduate degree (OR = 1.60, p = .038) are associated with higher odds of depression. | | |  |
|  |  |  |  |  |  | |  | |  |  | | |  |
|  |  | Household income | Categorized as a) <$17500; b) $17500-$31500; c) > $31000; and d) Preferred not to respond. | Actual |  | | Non-significant | |  | Non-significant | | |  |
|  |  |  |  |  |  | |  | |  |  | | |  |
|  |  | Financial difficulties in household | Binary response (Yes/No) to assess whether any household member who has stopped receiving income in the past 2 weeks, whose salary was reduced, or who lost their job | Actual |  | | Financial difficulties was associated with higher anxiety symptoms (OR = 1.55, p < .001). | |  | Non-significant | | |  |

**Table 5 (Continued)**

*The Main Outcomes of the Studies Included in the Systematic Review*

| Study ID | Quality Assessment | SEC  Variable | Measurement  Method | Actual / Perceived | | Anxiety    Inventory Outcomes | | Depression    Inventory Outcomes | | | | Note | | | |
| --- | --- | --- | --- | --- | --- | --- | --- | --- | --- | --- | --- | --- | --- | --- | --- |
|  |  |  |  |  |  | |  |  | |  | | |  | | |
| #989 | 8 | Job transitions | Categorized as a) Lost employment; b) Furloughed; c) Reduced hours or income; and d) Work from home | Actual | BAI | | Non-significant | | CESD-8 | Job transition was significantly associated with higher depression (F[5, 6933] = 4.43, p = .0005). | | | |  | |
|  |  |  |  |  |  | |  | |  |  | | | |  | |
| #986 | 8 | Education | Categorized as a) <= GED or equivalent; b) > GED or equivalent | Actual | GAD-7 | | Non-significant | | NA | NA | | | |  | |
|  |  |  |  |  |  | |  | |  |  | | | |  | |
|  |  | Income | Categorized as a) < $21000; b) $21000-$40000; and c) > $40000. | Actual |  | | Higher annual income, defined as >$40,000, decreased the odds of experiencing anxiety by a factor of 0.04 (p = .001). | |  | NA | | | |  | |
|  |  |  |  |  |  | |  | |  |  | | | |  | |
| #909 | 8 | Financial loss or not having enough basic supplies | Binary response (Yes / No) to the question: “Which of the following are you experiencing (or did you experience) during COVID-19?” | Perceived | Kessler Psychological Distress Scale | | Financial loss or not having enough basic supplies due to COVID-19 was significantly associated with higher likelihood of anxiety (B = 0.38, p < .01) | | Kessler Psychological Distress Scale | | Financial loss or not having enough basic supplies due to COVID-19 was significantly associated with higher depression (Î² = 0.38, p < .001) | | |  | |
|  |  |  |  |  |  | |  | |  | |  | | |  | |
|  |  | Income or job loss due to COVID-19 | Yes / No | Actual |  | | Income or job loss due to COVID-19 was significantly associated with higher likelihood of anxiety (B = 0.63, p < .001) | |  | Income or job loss due to COVID-19 was significantly associated with higher likelihood of depression (B = 0.63, p < .001) | | | | |  |
|  |  |  |  |  |  | |  | |  |  | | | | |  |
| #807 | 8 | Education | Categorized as a) Secondary; b) Diploma; c) Degree (Bachelor); and d) Masters and above | Actual | K10 | | Non-significant | | K10 | Non-significant | | | |  | |
|  |  |  |  |  |  | |  | |  |  | | | |  | |
|  |  | Employment status | Categorized as a) Unemployed/Home duties; b) Job affected by Covid-19 (lost job/working hours reduced etc.); and c) Have an income source (employed/Government benefits) | Actual |  | | Non-significant | |  | Non-significant | | | |  | |
|  |  |  |  |  |  | |  | |  |  | | | |  | |
|  |  | Perceived distress due to change of employment status | Categorized as a) A little or none; b) Moderate to a great deal | Perceived |  | | Non-significant | |  | Non-significant | | | |  | |
|  |  |  |  |  |  | |  | |  |  | | | |  | |
|  |  | Covid-19 impacted financial situation | Yes / No | Perceived |  | | Impacted financial situation significantly associated with psychological distress (aOR = 2.16, p < .001). | |  | Impacted financial situation significantly associated with psychological distress (aOR = 2.16, p < .001). | | | |  | |

**Table 5 (Continued)**

*The Main Outcomes of the Studies Included in the Systematic Review*

| Study ID | Quality Assessment | SEC  Variable | Measurement  Method | Actual / Perceived | | Anxiety    Inventory Outcomes | | Depression    Inventory Outcomes | | | | Note | | | |
| --- | --- | --- | --- | --- | --- | --- | --- | --- | --- | --- | --- | --- | --- | --- | --- |
|  |  |  |  |  |  | |  |  | |  | | |  | | |
| #725 | 8 | Place of residence | Categorised into a) Urban, b) Rural-urban, and c) Rural | Actual | GAD-7 | | Living in urban areas, in contrast to rural areas, was a protective factor against anxiety (OR= 0.810, p < .05) | | NA | NA | | | |  | |
|  |  |  |  |  |  | |  | |  |  | | | |  | |
|  |  | Steady family income | Categorised as a) Steady family income and b) Not having steady family income | Perceived |  | | Having stable family income was a protective factor against anxiety (OR = 0.726, p < .05) | |  | NA | | | |  | |
|  |  |  |  |  |  | |  | |  |  | | | |  | |
| #688 | 8 | Fear or inability to pay rent or mortgage | Yes / No | Perceived | SAS | | Non-significant | | NA | NA | | | |  | |
|  |  |  |  |  |  | |  | |  |  | | | |  | |
|  |  | Education | Categorized as: a) College degree or below; b) Bachelor; and c) Postgraduate or above | Actual |  | | Non-significant | |  | | NA |  | | | |
|  |  |  |  |  |  | |  | |  | |  | | |  | |
| #676 | 8 | Employment status | Categorized as a) "I am unemployed since the epidemic"; and b) "My employment status has been unaffected since the epidemic" | Actual | GAD-7 | | Unemployment was associated with higher anxiety (aOR = 2.60, p = .001). | | PHQ-9 | Unemployment was associated with higher depression (aOR = 2.11, p = .003). | | | | |  |
|  |  |  |  |  |  | |  | |  |  | | | | |  |
|  |  | Income change | Categorized as a) "I have completely lost my income"; b) My monthly income has decreased"; and c) "My monthly income has been unaffected" | Perceived |  | | Complete loss of income was associated with higher anxiety (aOR = 2.48, p = .02). | |  | Reduced and complete loss of income was associated with higher depression (aOR = 2.22, p = .001; aOR = 2.56, p = .002). | | | |  | |
|  |  |  |  |  |  | |  | |  |  | | | |  | |
| #661 | 8 | Education | Categorized as a) Secondary; b) High school; c) Diploma; and d) University. | Actual | DASS-21 | | Non-significant | | DASS-21 | Non-significant | | | |  | |
|  |  |  |  |  |  | |  | |  |  | | | |  | |
|  |  | Sufficiency of income for expenses | Categorized as a) Completely sufficient; b) Fairly sufficient; and c) Insufficient. | Perceived |  | | Non-significant | |  | Non-significant | | | |  | |
|  |  |  |  |  |  | |  | |  |  | | | |  | |
|  |  | Residence | Categorized as a) Personal; b) Rental; and c) Other (parents', relatives' or corporate house) | Actual |  | | Non-significant | |  | Non-significant | | | |  | |

**Table 5 (Continued)**

*The Main Outcomes of the Studies Included in the Systematic Review*

| Study ID | Quality Assessment | SEC  Variable | Measurement  Method | Actual / Perceived | | Anxiety    Inventory Outcomes | | Depression    Inventory Outcomes | | | | | Note | | | |
| --- | --- | --- | --- | --- | --- | --- | --- | --- | --- | --- | --- | --- | --- | --- | --- | --- |
|  |  |  |  |  |  | |  |  | | |  | | |  | | |
| #633 | 8 | Employment status | Categorized as a) Employee; b) Self-employed, c) Business owner, d) Not currently in the work force, e) Retired, and f) Never had a job | Actual | K10/ GAD-7 | | Non-significant | | K10 | | Non-significant | | | |  | |
|  |  |  |  |  |  | |  | |  | |  | | | |  | |
|  |  | Type of work | Categorized as a) Non-essential worker; b) Essential worker; and c) Not working | Actual |  | | Non-significant | |  | | Non-significant | | | |  | |
|  |  |  |  |  |  | |  | |  | |  | | | |  | |
|  |  | Job loss | Categorized as a) Lost or less work; and b) Not lost or less work | Actual |  | | Increased rates of distress were seen among those who reported having lost their jobs or experienced a reduction in work as a result of the pandemic (aOR= 1.75, p = .003) | |  | | Increased rates of distress were seen among those who lost their jobs or experienced a reduction in work as a result of the pandemic  (aOR= 1.75, p= .003 | | | |  | |
|  |  |  |  |  |  | |  | |  | |  | | | |  | |
| #613 | 8 | Education | Categorized as a) Primary; b) Secondary; c) High school; d) College; e) Specialty; f) Master; and g) Doctorate | Actual | DASS-21 | | Non-significant | | DASS-21 | | | Non-significant |  | | | |
|  |  |  |  |  |  | |  | |  | | |  | | |  | |
|  |  | Income (monthly) | Categorized as a) 0 to 4k; b) 4001 to 8k; c) 8001 to 12k; d) 12001 to 16k; and e) >16k | Actual |  | | Non-significant | |  | Non-significant | | | | | |  |
|  |  |  |  |  |  | |  | |  | |  | | | | |  |
|  |  | Risk of job loss | Yes / No | Perceived |  | | Non-significant | |  | | Non-significant | | | |  | |
|  |  |  |  |  |  | |  | |  | |  | | | |  | |
|  |  | Monthly income loss (monthly) | Yes / No | Actual |  | | Non-significant | |  | | Non-significant | | | |  | |
|  |  |  |  |  |  | |  | |  | |  | | | |  | |
| #601 | 8 | Employment status | Working / Not working | Actual | GAD-7 | | Unemployed reported more anxiety symptoms (β = .12 p < .01). | | CES-D | | Unemployed reported more depressive symptoms (β = .10 p < .01). | | | |  | |
|  |  |  |  |  |  | |  | |  | |  | | | |  | |
| #593 | 8 | SES | Census-based geocoding for neighbourhood-based variables (e.g. median household income), obtained using participant zip codes. | Actual | GAD-7 | | Non-significant | | PHQ-9 | | Non-significant | | | |  | |
|  |  |  |  |  |  | |  | |  | |  | | | |  | |
|  |  | Ethnicity | Black as compared to White | Actual |  | | Non-significant | |  | | Non-significant | | | |  | |
|  |  |  |  |  |  | |  | |  | |  | | | |  | |

**Table 5 (Continued)**

*The Main Outcomes of the Studies Included in the Systematic Review*

| Study ID | Quality Assessment | SEC  Variable | Measurement  Method | Actual / Perceived | | Anxiety    Inventory Outcomes | | Depression    Inventory Outcomes | | | | | | Note | | | | |
| --- | --- | --- | --- | --- | --- | --- | --- | --- | --- | --- | --- | --- | --- | --- | --- | --- | --- | --- |
|  |  |  |  |  |  | |  |  | | |  | | | |  | | | |
| #581 | 8 | Education | Categorized as a) College and below; and b) Bachelor's degree and above | Actual | GAD-7 | | Non-significant | | PHQ-9 | | Non-significant | | | | |  | | |
|  |  |  |  |  |  | |  | |  | |  | | | | |  | | |
|  |  | Income (monthly) | Categorized as a) <1000 CNY; b) 1000-5000 CNY; and c) >5000 CNY | Actual |  | | Those with monthly income more than 1000 (1000–5000: OR = 1.44, p = 0.035; >5000: OR = 1.47, p = 0.046), were more likely to have anxiety | |  | | Those with monthly income more than 1000  (1000–5000: OR = 1.83, p = 0.000;  >5000: OR = 1.45, p = 0.027),  showed higher risk of depression | | | | | |  | |
|  |  |  |  |  |  | |  | |  | |  | | | | | |  | |
| #548 | 8 | SES | Binary response (Yes / No) to assess financial problem because of Covid-19 pandemic | Perceived | PGWB | | Financial problem was significantly associated with higher anxiety (p < .05). | | PGWB | | | Financial problem was significantly associated with higher depression (p < .05). | | | | |  | |
|  |  |  |  |  |  | |  | |  | | |  | | | | |  | |
| #536 | 8 | Place of residence | Categorised as a) Urban; b) Semi-rural; and c) Rural | Actual | DASS-21 | | Non-significant | | NA | | | | NA |  | | | | |
|  |  |  |  |  |  | |  | |  | | | |  | | |  | | |
|  |  | Stable family income | Having stable family income / Not having stable family income | Perceived |  | | Non-significant | |  | NA | | | | | | | |  |
|  |  |  |  |  |  | |  | |  | |  | | | | | | |  |
|  |  | Concern about future economic scenario | Concerned about future economic scenario / Not being concerned about future economic scenario | Perceived |  | | Concern about the future economic scenario was significantly associated with mild to moderate (aOR = 2.37, p < .05) and severe (aOR = 3.03, p < .05) levels of anxiety | |  | | NA | | | | |  | | |
|  |  |  |  |  |  | |  | |  | |  | | | | |  | | |
| #534 | 8 | Economic level | Categorized as a) Below average; b) Average; and c) Above average. | Perceived | MHI-5 | | Non-significant. | | MHI-5 | | Non-significant. | | | | |  | | |
|  |  |  |  |  |  | |  | |  | |  | | | | |  | | |

**Table 5 (Continued)**

*The Main Outcomes of the Studies Included in the Systematic Review*

| Study ID | Quality Assessment | SEC  Variable | Measurement  Method | Actual / Perceived | | Anxiety    Inventory Outcomes | | Depression    Inventory Outcomes | | | | Note | | |
| --- | --- | --- | --- | --- | --- | --- | --- | --- | --- | --- | --- | --- | --- | --- |
|  |  |  |  |  |  | |  |  | |  | | |  | |
| #512 | 8 | Education | Categorized as a) Primary school; and b) Graduate, postgraduate | Actual | PROMIS Anxiety V.8a | | Higher education was associated with lower anxiety (B = -1.59, p < .001). | PROMIS Depression V.8a | | | Higher education was associated with lower depression (B = -1.74, p < .001). | | |  |
|  |  |  |  |  |  | |  |  | | |  | | |  |
|  |  | Income (household) | Categorized as (in $) a) > 4480; b) 1993-4480; c) 1029-1993; d) 569-1029; e) 324-569; f) 136-324; and g) < 136. | Actual |  | | Higher household income was associated with lower anxiety (B = -2.31, p < .001). | |  | Higher household income was associated with lower depression (B = -2.39, p < .001). | | | |  |
|  |  |  |  |  |  | |  | |  |  | | | |  |
|  |  | Employment status | Categorized as a) Employed; b) Self-employed; c) Unemployed; d) Homemaker; e) Student; and f) Retired/retired on disability. | Actual |  | | Non-significant | |  | Non-significant | | | |  |
|  |  |  |  |  |  | |  | |  |  | | | |  |
|  |  |  |  |  |  | |  | |  |  | | | |  |
| #507 | 7 | Parents' education | Categorized as a) Junior high school and below; and b) High school and above | Actual | GAD-7 | | Father's education of high school and above was associated with higher risk of anxiety (OR = 1.055, p =.000) | | NA | NA | | | |  |
|  |  |  |  |  |  | |  | |  |  | | | |  |
|  |  | Income (family) | Categorized as a) Good; b) Fair; and c) Bad | Perceived |  | | Having a bad family income (OR = 1.52, p =.000) was associated with higher risk of anxiety. But not for fair family income. | |  | NA | | | |  |
|  |  |  |  |  |  | |  | |  |  | | | |  |
| #503 | 8 | Employment loss | Binary response (Yes / No) to assess whether the respondent or anyone in their household experienced a loss of employment due to COVID-19 | Actual | GAD-7 | | Direct or household employment loss was associated with higher anxiety (aRR = 1.22, p < .001) | | PHQ-9 | Direct or household employment loss was associated with higher depression (aRR = 1.25, p < .001) | | | |  |
|  |  |  |  |  |  | |  | |  |  | | | |  |
|  |  | Expected employment loss | Binary variable (Yes / No) to assess whether the respondent expects themselves or anyone in the household to experience a loss of employment soon due to COVID-19 | Perceived |  | | Expected direct or household employment loss was associated with higher anxiety (aRR = 1.56, p < .001) | |  | Expected direct or household employment loss was associated with higher depression (aRR = 1.63, p < .001) | | | |  |

**Table 5 (Continued)**

*The Main Outcomes of the Studies Included in the Systematic Review*

| Study ID | Quality Assessment | SEC  Variable | Measurement  Method | Actual / Perceived | | Anxiety    Inventory Outcomes | | | Depression    Inventory Outcomes | | | | | Note | | | | | |
| --- | --- | --- | --- | --- | --- | --- | --- | --- | --- | --- | --- | --- | --- | --- | --- | --- | --- | --- | --- |
|  |  |  |  |  |  | |  | |  | |  | | | |  | | | | |
| #501 | 8 | Employment status | Categorized as a) Working-full-time; b) Working part-time; c) Unemployed; d) On parental leave; and e) Retired | Actual | NA | | NA | Multidimensional State Boredom Scale (using 2 items of disengagement subscale of it) | | | | Non-significant | | | |  | | | |
|  |  |  |  |  |  | |  |  | | | |  | | | |  | | | |
|  |  | Education | Categorized as a) Primary school; b) High school; c) Some college/university; d) Graduated from college/university; e) Master/postgraduate; f) Doctoral level; and g) Other | Actual |  | | NA |  | | | | Less depression symptoms were observed for higher education - Doctoral (OR= -.31, B = -.67); moderate effect sizes | | | | |  | | |
|  |  |  |  |  |  | |  |  | | | |  | | | | |  | | |
|  |  | Finance | Categorized as a) Have gotten better; b) Stayed about the same; and c) Have gotten worse | Perceived |  | | NA | | |  | Non-significant | | | | |  | | | |
|  |  |  |  |  |  | |  | | |  |  | | | | |  | | | |
|  |  | Obtain all basic supplies | Yes / No | Actual |  | | NA |  | | | | Not being able to obtain all basic supplies was associated with higher depression with moderate effect size (B= 0.49, OR= .21). | | | | | |  | |
|  |  |  |  |  |  | |  | | |  | |  | | | | | |  | |
| #499 | 8 | Household income shock | Binary variable (Yes / No) to the question: "Have you, or has anyone in your house-hold, experienced a loss of employment income since March 13, 2020?" | Actual | GAD-2 | | Adults experiencing an income shock in their household have 83% greater odds of anxiety compared to adults without household income shock (p <.001) | | | PHQ-2 | | Adults experiencing an income shock in their household have 75% greater odds of depression compared to adults without household income shock (p < .001) | | | | | | |  |
|  |  |  |  |  |  | |  | | |  | |  | | | | | | |  |
|  |  | Education | Categorized as a) Less than high school diploma; b) High school diploma; c) Some college; d) Associate's degree; and e) Bachelor's degree or graduate degree. | Actual |  | | Odds of anxiety were greater for respondents with less education. | | |  | | | Odds of depression were greater for respondents with less education. | | |  | | | |
|  |  |  |  |  |  | |  | | |  | | |  | | |  | | | |

**Table 5 (Continued)**

*The Main Outcomes of the Studies Included in the Systematic Review*

| Study ID | Quality Assessment | SEC  Variable | Measurement  Method | Actual / Perceived | | Anxiety    Inventory Outcomes | | Depression    Inventory Outcomes | | | | Note | | |
| --- | --- | --- | --- | --- | --- | --- | --- | --- | --- | --- | --- | --- | --- | --- |
|  |  |  |  |  |  | |  |  | |  | | |  | |
| #497 | 8 | SES | 5-point Likert scale (1 = Worst and 5 = Best) | Perceived | SMFQ | | Being poor was associated with greater mental distress (B = 0.215) | SMFQ | | | Being poor was associated with greater mental distress (B = 0.215) | | |  |
|  |  |  |  |  |  | |  |  | | |  | | |  |
|  |  | Unemployed | Yes / No | Actual |  | | Being unemployed was associated with greater mental distress (B = 0.127) | |  | Being unemployed was associated with greater mental distress (B = 0.127) | | | |  |
|  |  |  |  |  |  | |  | |  |  | | | |  |
|  |  | Laid-off | Yes / No | Actual |  | | Being laid-off was associated with greater mental distress (B = 0.319) | |  | Being laid-off was associated with greater mental distress (B = 0.319) | | | |  |
|  |  |  |  |  |  | |  | |  |  | | | |  |
| #477 | 7 | Education | Unclear | Actual | NA | | NA | | CES-D | Non-significant | | | |  |
|  |  |  |  |  |  | |  | |  |  | | | |  |
|  |  | Employment status | Categorized as a) Unemployed; b) Self-employed; c) Civil servants; d) Private clerks; and e) University/college students | Actual |  | | NA | |  | Non-significant | | | |  |
|  |  |  |  |  |  | |  | |  |  | | | |  |
|  |  | Changes in economic situation | Unclear | Unclear |  | | NA | |  | Worsened economic situation was associated with higher depression (β = -.068, p = .016) | | | |  |
|  |  |  |  |  |  | |  | |  |  | | | |  |
| #462 | 8 | Financial vulnerability | Categorized as a) Very low level of vulnerability; b) Low level of vulnerability; c) Medium level of vulnerability; d) High level of vulnerability; and e) Extreme level of vulnerability | Perceived | GHQ-12 | | Individuals who perceived that they were financially vulnerable due to COVID-19 were more likely to experience mental distress (B = 0.294, p < .001) | | GHQ-12 | Individuals who perceived that they were financially vulnerable due to COVID-19 were more likely to experience mental distress (B = 0.294, p < .001) | | | |  |
|  |  |  |  |  |  | |  | |  |  | | | |  |
| #407 | 8 | Education | Categorized as a) Less than high school; b) Completed high school' and c) Undergraduate or higher | Actual | NA | | NA | | PHQ-2 | Having an undergraduate degree or above had a lower prevalence ratio of depression. | | | |  |
|  |  |  |  |  |  | |  | |  |  | | | |  |
|  |  | Income (household) | Categorized as a) Up to 930 PEN; b) 931-1860 PEN; c) 1862 - 2790 PEN; d) 2791 - 4650 PEN; e) More than 4650 PEN; and f) Prefer not to respond | Actual |  | | NA | |  | Having higher household income had a lower prevalence ratio of depression. | | | |  |
|  |  |  |  |  |  | |  | |  |  | | | |  |
|  |  |  |  |  |  | |  | |  |  | | | |  |

**Table 5 (Continued)**

*The Main Outcomes of the Studies Included in the Systematic Review*

| Study ID | Quality Assessment | SEC  Variable | Measurement  Method | Actual / Perceived | | Anxiety    Inventory Outcomes | | Depression    Inventory Outcomes | | | | Note | | |
| --- | --- | --- | --- | --- | --- | --- | --- | --- | --- | --- | --- | --- | --- | --- |
|  |  |  |  |  |  | |  |  | |  | | |  | |
| #396 | 8 | Education | Categorized as a) Tertiary or above; b) Secondary; and c) Primary or below. | Actual | GAD-7 | | T1: Non-significant | PHQ-9 | | | T1: Non-significant | | | Please see Table 4 for T2 |
|  |  |  |  |  |  | |  |  | | |  | | |  |
|  |  | Employment status | Categorized as a) Employed; b) Dependent; and c) Unemployed. | Actual |  | | T1: Non-significant | |  | T1: Unemployed was associated with higher depression (aOR: 1.52, p = .026). | | | |  |
|  |  |  |  |  |  | |  | |  |  | | | |  |
|  |  | Income (monthly household) | Categorized as a) $80000 or above; b) $60000-$79999; c) $40000-$59999; d) $20000-$39999; and e) $19999 or below. | Actual |  | | T1: Non-significant | |  | T1: Non-significant | | | |  |
|  |  |  |  |  |  | |  | |  |  | | | |  |
|  |  | Income change | Categorized as a) Stable/Increase; and b) Decrease. | Perceived |  | | T1: Income decline was associated with higher anxiety (aOR = 1.45, p < .001). | |  | T1: Income decline was associated with higher depression (aOR = 1.48, p < .001). | | | |  |
|  |  |  |  |  |  | |  | |  |  | | | |  |
|  |  | Savings | Categorized as a) $3,000,000 or above; b) $2,000,000-$2,999,999; c) $1,000,000-$1,999,999; d) $500,000-$999,999; e) $200,000-$499,999; f) Less than $200,000; and g) None. | Actual |  | | T1: Non-significant | |  | T1: Non-significant | | | |  |
|  |  |  |  |  |  | |  | |  |  | | | |  |
| #381 | 8 | Income (monthly household) | Categorized as a) Low (<1500 euro); b) Medium (between 1500 euro and 3000 euro); and c) High (>3000 euro). | Actual | HADS | | Lower income was associated with higher psychological distress (B = -0.28, p = .004). | | HADS | Lower income was associated with higher psychological distress (B = -0.28, p = .004). | | | |  |
|  |  |  |  |  |  | |  | |  |  | | | |  |
|  |  | Education level in the family | Categorized as a) Primary; b) Secondary; c) University degree; d) Master degree; and e) PhD or equivalent. | Actual |  | | Lower level of education was associated with higher psychological distress (B = -0.31, p < .001). | |  | Lower level of education was associated with higher psychological distress (B = -0.31, p < .001). | | | |  |
|  |  |  |  |  |  | |  | |  |  | | | |  |
|  |  | House with a garden or balcony | Yes / No | Actual |  | | House with a garden or balcony was associated with lower psychological distress (B = -1.34, p < .001). | |  | House with a garden or balcony was associated with lower psychological distress (B = -1.34, p < .001). | | | |  |
|  |  |  |  |  |  | |  | |  |  | | | |  |
|  |  |  |  |  |  | |  | |  |  | | | |  |

**Table 5 (Continued)**

*The Main Outcomes of the Studies Included in the Systematic Review*

| Study ID | Quality Assessment | SEC  Variable | Measurement  Method | Actual / Perceived | | Anxiety    Inventory Outcomes | | Depression    Inventory Outcomes | | | | Note | | |
| --- | --- | --- | --- | --- | --- | --- | --- | --- | --- | --- | --- | --- | --- | --- |
|  |  |  |  |  |  | |  |  | |  | | |  | |
| #365 | 8 | Economic status | Categorised into a) Low; b) Middle; and c) High | Perceived | GAD-7 | | Non-significant | PHQ-9 | | | Respondents from the middle economic class were 1.62 times more likely than those from high economic class to have depression (OR = 1.62, p = .033). But not significant for low economic class. | | |  |
|  |  |  |  |  |  | |  |  | | |  | | |  |
|  |  | Place of residence | Categorised into a) Urban; and b) Rural | Actual |  | | Non-significant | |  | Urban respondents were 0.66 times less likely to have depression than rural respondents (OR = 0.66, p = .044) | | | |  |
|  |  |  |  |  |  | |  | |  |  | | | |  |
|  |  | Education | Categorised into a) Illiterate; b) Primary; c) Secondary; d) Higher secondary; and e) Graduate/above | Actual |  | | Non-significant | |  | Non-significant | | | |  |
|  |  |  |  |  |  | |  | |  |  | | | |  |
| #349 | 8 | Employment status | Categorized as a) Full-time worker; b) No regular employment; and c) Unemployed (including homemaker, retired, and jobless). | Actual | NA | | NA | | PHQ-9 | Not working was associated with higher depression (OR = 1.85, p < .01). | | | |  |
|  |  |  |  |  |  | |  | |  |  | | | |  |
|  |  | Income (household) | Categorized as a) < 2mill JPY; b) 2-8mill JPY; and c) > 8mill JPY | Actual |  | | NA | |  | Household income > 8mill JPY was associated with lower depression (OR = 0.45, p < .01). | | | |  |
|  |  |  |  |  |  | |  | |  |  | | | |  |
|  |  | Economic impact | Categorized as a) Without impact; b) Negative impact; and c) Positive impact | Perceived |  | | NA | |  | Negative impact was associated with higher depression (OR = 1.33, p < .05). | | | |  |
|  |  |  |  |  |  | |  | |  |  | | | |  |
| #348 | 7 | Education | Categorized as a) Primary; b) Secondary; c) Bachelor; d) Master; and e) Doctoral | Actual | NA | | NA | | PHQ-9 | Non-significant | | | |  |
|  |  |  |  |  |  | |  | |  |  | | | |  |
|  |  | Economic Situation | Categorized as a) Poor; b) Middle; and c) Good | Perceived |  | | NA | |  | Non-significant | | | |  |
|  |  |  |  |  |  | |  | |  |  | | | |  |
|  |  | Employment status | Categorized as a) No; b) seasonal; and c) Yes | Actual |  | | NA | |  | Non-significant | | | |  |
|  |  |  |  |  |  | |  | |  |  | | | |  |

**Table 5 (Continued)**

*The Main Outcomes of the Studies Included in the Systematic Review*

| Study ID | Quality Assessment | SEC  Variable | Measurement  Method | Actual / Perceived | | Anxiety    Inventory Outcomes | | Depression    Inventory Outcomes | | | | Note | | |
| --- | --- | --- | --- | --- | --- | --- | --- | --- | --- | --- | --- | --- | --- | --- |
|  |  |  |  |  |  | |  |  | |  | | |  | |
| #296 | 8 | Relative economic situation | Categorized as a) Above average; b) Average; and c) Below average | Perceived | HSCL-5 | | Relative economic situation/pre-existing economic challenges (B = .37, p < .001) associated with more psychological distress | HSCL-5 | | | Relative economic situation/pre-existing economic challenges (B = .37, p < .001) associated with more psychological distress | | |  |
|  |  |  |  |  |  | |  |  | | |  | | |  |
|  |  | Covid-related unemployment | Yes / No | Actual |  | | Non-significant | |  | Non-significant | | | |  |
|  |  |  |  |  |  | |  | |  |  | | | |  |
| #288 | 11 | Education | Categorized as a) High school or less; b) College degree; and c) Postgraduate. | Actual | HADS | | Non-significant | | HADS | Non-significant | | | |  |
|  |  |  |  |  |  | |  | |  |  | | | |  |
|  |  | Economic impact of Covid-19 | 1-item variable categorized as a) No change; b) Decreased; and c) Increased to the question: "Did social distancing affect your monthly income?" | Perceived |  | | Decreased monthly income (aPR = 1.15, p = .011) were associated with a higher likelihood of more severe anxiety symptoms during social distancing. | |  | Decreased monthly income (aPR = 1.23, p = .047) were associated with a higher likelihood of more severe depressive symptoms during social distancing. | | | |  |
|  |  |  |  |  |  | |  | |  |  | | | |  |
| #278 | 11 | Education | Categorised as a) Less than complete secondary education; b) Complete secondary education; and c) Diploma/certificate/degree | Actual | GAD-2 | | Anxiety was higher in households with members that had a diploma / certificate/degree (PR = 1.16) | | PHQ-2 | Depression was higher in households with members that had a diploma / certificate/degree (PR = 1.33) | | | |  |
|  |  |  |  |  |  | |  | |  |  | | | |  |
|  |  | Household income sources in 2019 | Categorised as a) Full/part-time employees; b) Pension grants; and c) Non-pension grants | Actual |  | | Non-significant | |  | Non-significant | | | |  |
|  |  |  |  |  |  | |  | |  |  | | | |  |
|  |  | Household asset index quintiles | Categorised as node-specific household wealth quintile a) Lowest; b) Second lowest; c) Middle; d) Second highest; and e) Highest | Actual |  | | Non-significant | |  | Non-significant | | | |  |
|  |  |  |  |  |  | |  | |  |  | | | |  |

**Table 5 (Continued)**

*The Main Outcomes of the Studies Included in the Systematic Review*

| Study ID | Quality Assessment | SEC  Variable | Measurement  Method | Actual / Perceived | | Anxiety    Inventory Outcomes | | Depression    Inventory Outcomes | | | | Note | | | |
| --- | --- | --- | --- | --- | --- | --- | --- | --- | --- | --- | --- | --- | --- | --- | --- |
|  |  |  |  |  |  | |  |  | |  | | |  | | |
| #259 | 11 | Education | Years of education | Actual | GAD-7 | | Non-significant | PHQ-9 | | | Non-significant | | |  | |
|  |  |  |  |  |  | |  |  | | |  | | |  | |
|  |  | Job loss | Yes / No | Actual |  | | Non-significant |  | | Non-significant | | | | |  |
|  |  |  |  |  |  | |  | |  |  | | | |  | |
|  |  | Covid-19 related financial distress | Yes / No | Perceived |  | | Higher distress was associated with higher anxiety score | |  | Higher distress was associated with higher depression score | | | |  | |
|  |  |  |  |  |  | |  | |  |  | | | |  | |
| #256 | 7 | Income (monthly) | <5000 RMB or >5000 RMB | Actual | SAS | | Risk of anxiety for monthly income of less than 5,000 RMB was 4.223 times higher than that in patients with a monthly income of more than 5,000 RMB. | | SDS | Risk of depression for monthly income of less than 5,000 RMB was 3.562 times higher than that among patients with a monthly income of more than 5,000 RMB | | | |  | |
|  |  |  |  |  |  | |  | |  |  | | | |  | |
| #239 | 10 | Financial concerns | 5-items inventory to assess participants' concern about personal, family economy (4-items) and U.S. economy (1-item). | Perceived | GAD-7 | | Greater financial concern was significantly associated with greater anxiety (Sample 1 and 2 only), with small effect size. | | PHQ-8 | Greater financial concern was significantly associated with greater depression (Sample 1 only), with small effect size. | | | |  | |
|  |  |  |  |  |  | |  | |  |  | | | |  | |
| #224 | 8 | Education | Categorized as a) Less than University; and b) University degree or higher | Actual | NA | | NA | | CESD-10 | Depression was highly prevalent and nearly seven times higher in men with a university degree | | | |  | |
|  |  |  |  |  |  | |  | |  |  | | | |  | |
|  |  | Job loss because of Covid-19 | Yes / No | Actual |  | | NA | |  | Job loss are associated with an increased  risk of depression | | | |  | |
|  |  |  |  |  |  | |  | |  |  | | | |  | |
| #221 | 10 | Education | Categorised as a) School – 16; b) 16 -18; c) Undergrad; and d) Postgrad | Actual | GAD-7 | | Non-significant | | PHQ-9 | Non-significant | | | |  | |
|  |  |  |  |  |  | |  | |  |  | | | |  | |
|  |  | Employment status | Categorised as a) Employed (full-time); b) Employed (part-time); c) Self-employed; d) Retired; and e) Unemployed | Actual |  | | Being employed full time was associated with lower anxiety (IRR = .88, p = .02) | |  | Being retired was associated with greater depression (IRR = 1.11, p = .001) | | | |  | |
|  |  |  |  |  |  | |  | |  |  | | | |  | |
|  |  | Negative financial Impact | Not stated | Unclear |  | | Non-significant | |  | Non-significant | | | |  | |
|  |  |  |  |  |  | |  | |  |  | | | |  | |

**Table 5 (Continued)**

*The Main Outcomes of the Studies Included in the Systematic Review*

| Study ID | Quality Assessment | SEC  Variable | Measurement  Method | Actual / Perceived | | Anxiety    Inventory Outcomes | | Depression    Inventory Outcomes | | | | | Note | | | |
| --- | --- | --- | --- | --- | --- | --- | --- | --- | --- | --- | --- | --- | --- | --- | --- | --- |
|  |  |  |  |  |  | |  |  | |  | | | |  | | |
| #204 | 8 | Education | Categorized as a) Until secondary/equivalent; b) Pre-U/Diploma; c) University; d) High diploma; e) Master; and f) PhD | Actual | HADS | | Pre-U/Diploma holders has the highest risk for anxiety compared to other education levels (aOR = 5.290, p < .001). | HADS | | | Pre-U/Diploma holders has the highest risk for depression compared to other education levels (aOR = 2.556, p < .001). | | | |  | |
|  |  |  |  |  |  | |  |  | | |  | | | |  | |
|  |  | Employment status | Categorized as a) Unemployed/Not working/Other; b) Working part-time; c) Retired; and d) Healthcare worker | Actual |  | | Working fulltime or healthcare worker was associated with higher risk for anxiety (aOR = 1.850, p = .001; aOR = 1.725, p < .001). |  | | Non-significant | | | | | |  |
|  |  |  |  |  |  | |  | |  |  | | | | |  | |
|  |  | Income (gross household) | Categorized as a) < USD 1333 per month; b) USD 2666.67-3999.74; c) USD 4000-5333.07; d) USD 5333.34-6666.41; e) > USD 6666.67; and f) Don't know/Prefer not to say | Actual |  | | Higher gross household income was associated with lower risk for anxiety | |  | Non-significant | | | | |  | |
|  |  |  |  |  |  | |  | |  |  | | | | |  | |
| #203 | 9 | Income | Categorized as a) Lower-income (annual household < $54,000); b) Middle income (>&54000 and <$100000); and c) Higher income (>$100000). | Actual | GAD-7 | | Non-significant | | NA | NA | | | | | Please see Table 4 for T2 | |
|  |  |  |  |  |  | |  | |  |  | | | | |  | |
| #180 | 8 | Education | Categorized as a) HS or less; b) Some college; c) Bachelor's degree; and d) Master's degree or higher | Actual | PROMIS-29 Profile v2.0 | | Non-significant. | | PROMIS-29 Profile v2.0 | | | Non-significant. | | |  | |
|  |  |  |  |  |  | |  | |  | | |  | | |  | |
|  |  | Income | Categorized as a) <$25,000; b) $25,000-$49,999; c) $50,000-$99,999; and d) $100,000 and higher. | Actual |  | | Lower income was associated with higher anxiety. | |  | Lower income was associated with higher depression. | | | | |  | |
|  |  |  |  |  |  | |  | |  |  | | | | |  | |
| #177 | 8 | Financial distress | Categorized as a) A lot less stressful; b) Somewhat less stressful; c) No significant change; d) Somewhat more stressful; and e) A lot more stressful | Perceived | GAD-7 | | Financial distress was associated with higher anxiety (OR = 1.50, p < .001) | | PHQ-9 | Financial distress was associated with greater depression (OR = 1.67, p < .001) | | | | |  | |
|  |  |  |  |  |  | |  | |  |  | | | | |  | |

**Table 5 (Continued)**

*The Main Outcomes of the Studies Included in the Systematic Review*

| Study ID | Quality Assessment | SEC  Variable | Measurement  Method | Actual / Perceived | | Anxiety    Inventory Outcomes | | Depression    Inventory Outcomes | | | | | Note | | | |
| --- | --- | --- | --- | --- | --- | --- | --- | --- | --- | --- | --- | --- | --- | --- | --- | --- |
|  |  |  |  |  |  | |  |  | |  | | | |  | | |
| #172 | 7 | Education | Categorized as a) Illiterate; b) Basic; and c) Secondary and above | Actual | BAI | | Non-significant | BDI | | | Non-significant | | | |  | |
|  |  |  |  |  |  | |  |  | | |  | | | |  | |
|  |  | Income | Categorized as a) < 15000 NPR; and b) > 15000 NPR | Actual |  | | Non-significant |  | | Non-significant | | | | | |  |
|  |  |  |  |  |  | |  | |  |  | | | | |  | |
| #171 | 8 | Financial worries | Categorized as a) Not worried; b) Somewhat; and c) Very worried | Perceived | NA | | NA | | PHQ-8 | Factors associated with a positive PHQ-8 depression screen were being “very worried” about household finances (OR = 2.27, p = 0.06)." | | | | |  | |
|  |  |  |  |  |  | |  | |  |  | | | | |  | |
|  |  | Income loss | Yes/ No / Not applicable | Actual |  | | NA | |  | Non-significant | | | | |  | |
|  |  |  |  |  |  | |  | |  |  | | | | |  | |
|  |  | Food security | 2 questions that reflect the household food security and food stock | Perceived |  | | NA | |  | | | Non-significant |  | | | |
|  |  |  |  |  |  | |  | |  | | |  | | |  | |
| #140 | 7 | Unemployment | Unclear | Unclear | PHQ-4 | | Unemployment was the strongest predictor of anxiety for 2018 cohort. | | PHQ-4 | Unemployment was the strongest predictor of depression for 2018 cohort. | | | | | Please see Table 4 for T2 | |
|  |  |  |  |  |  | |  | |  |  | | | | |  | |
|  |  | Income (household) | Unclear | Unclear |  | | Household income was the second strongest predictor of anxiety for 2018 cohort. | |  | Household income was the second strongest predictor of depression for 2018 cohort. | | | | |  | |
|  |  |  |  |  |  | |  | |  |  | | | | |  | |
| #139 | 8 | Financial difficulties due to COVID-19 | Binary response (Yes/No) to assess whether the respondent experienced financial difficulties due to the COVID-19 pandemic | Perceived | PHQ-4 | | Financial difficulties due to COVID-19 was associated with higher anxiety (OR = 1.428, p = .006) | | PHQ-4 | Financial difficulties due to COVID-19 was associated with greater depression (OR = 1.282, p = .027) | | | | |  | |
|  |  |  |  |  |  | |  | |  |  | | | | |  | |
| #137 | 7 | Education | Categorized as a) High school; b) Diploma; c) Bachelor; d) Masters; and e) PhD | Actual | SCL-48 | | Non-significant | | SCL-48 | Non-significant | | | | |  | |
|  |  |  |  |  |  | |  | |  |  | | | | |  | |
|  |  | Income | Unclear | Unclear |  | | Non-significant | |  | Non-significant | | | | |  | |

**Table 5 (Continued)**

*The Main Outcomes of the Studies Included in the Systematic Review*

| Study ID | Quality Assessment | SEC  Variable | Measurement  Method | Actual / Perceived | | Anxiety    Inventory Outcomes | | Depression    Inventory Outcomes | | | | | Note | | | |
| --- | --- | --- | --- | --- | --- | --- | --- | --- | --- | --- | --- | --- | --- | --- | --- | --- |
|  |  |  |  |  |  | |  |  | |  | | | |  | | |
| #133 | 8 | Education | Categorised as a) High school or below = 1; and b) College and above = 2 | Actual | DASS-21 | | Higher education levels were more likely to have lower levels of anxiety (B = 0.80, p < .05) | DASS-21 | | | Higher education levels were more likely to have lower levels of depression (B = 0.43, p < .05) | | | |  | |
|  |  |  |  |  |  | |  |  | | |  | | | |  | |
|  |  | Economical influence | Categorized as a) No influence = 1; b) Less than 50% of monthly income = 2; and c) More than 50% of monthly income = 3 | Actual |  | | Higher economic influence level were more likely to have higher levels of anxiety (B = 0.84, p < .05). |  | | Higher economic influence level were more likely to have higher levels of depression (B = 0.97, p < .05) | | | | | |  |
|  |  |  |  |  |  | |  | |  |  | | | | |  | |
|  |  | Occupation | Categorized as a) Medical staff, b) Educator, and c) Others (student) | Actual |  | | Non-significant | |  | Non-significant | | | | |  | |
|  |  |  |  |  |  | |  | |  |  | | | | |  | |
| #126 | 8 | COVID-19 being a personal financial threat | Binary response (Yes / No) to assess whether the respondents thought the COVID-19 pandemic represented a private financial risk | Perceived | DASS-21 | | COVID-19 being a personal financial threat was associated with higher anxiety (B = -.30, p = .00) | | DASS-21 | COVID-19 being a personal financial threat was associated with higher depression (B = -.32, p =.00) | | | | |  | |
|  |  |  |  |  |  | |  | |  |  | | | | |  | |
| #87 | 8 | Financial worry | 2-items inventory on 4-point Likert scale to the question: "I worry that I will lose my job" and "I am worry about my personal economy" | Perceived | GAD-7 | | Worry about personal economy was connected to the GAD symptom generalized worry | | PHQ-9 | | | Worry about personal economy was connected to the MDD symptom sleep problems |  | | | |
|  |  |  |  |  |  | |  | |  | | |  | | |  | |
| #85 | 8 | Education | Categorized as a) Analphabetic; b) Primary; c) Secondary; and d) University | Actual | GAD-7 | | Non-significant | | PHQ-9 | Non-significant | | | | |  | |
|  |  |  |  |  |  | |  | |  |  | | | | |  | |
|  |  | Income (monthly) | Categorized as a) <1000; b) 1000-2000; c) 2000-4000; d) 4000-6000; and e) > 6000 (in Dirhams) | Actual |  | | Lower monthly income were associated with higher risk of major symptoms of anxiety (OR = 1.79, p < .026). | |  | Non-significant | | | | |  | |
|  |  |  |  |  |  | |  | |  |  | | | | |  | |

**Table 5 (Continued)**

*The Main Outcomes of the Studies Included in the Systematic Review*

| Study ID | Quality Assessment | SEC  Variable | Measurement  Method | Actual / Perceived | | Anxiety    Inventory Outcomes | | Depression    Inventory Outcomes | | | Note | | |
| --- | --- | --- | --- | --- | --- | --- | --- | --- | --- | --- | --- | --- | --- |
|  |  |  |  |  |  | |  |  | |  | |  | |
| #65 | 8 | Changes to financial circumstances | Two (Yes / No) items assessing if mother and family member had experienced job loss, and two (Yes / No) items assessing if mother and family member had reduced ability to earn | Perceived | DASS-21 | | Mothers = Non-significant;  Children = Maternal job or income loss was associated with poorer child mental health (B = 0.37, p = 0.006) | | DASS-21 | Mothers = Non-significant;  Children = Maternal job or income loss was associated with poorer child mental health (B = 0.37, p = 0.006) | | |  |
|  |  |  |  |  |  | |  | |  |  | | |  |
|  |  | Current financial hardship | Five (Yes / No) items assessing if family experience of the following financial hardships/difficulties paying: a) Mortgage or rent; b) Household bills; c) Food; d) Healthcare; and e) Home or car insurance | Perceived |  | | Mothers = Greater financial hardship was associated with higher self-reported maternal mental health symptoms (B = 0.27, p < .001);  Children = Non-significant | |  | Mothers = Greater financial hardship was associated with higher self-reported maternal mental health symptoms (B = 0.27, p < .001);  Children = Non-significant | | |  |
|  |  |  |  |  |  | |  | |  |  | | |  |
| #50 | 6 | Education | Categorized as a) Absent; b) Primary; c) Secondary; and d) University. | Actual | NA | | NA | | BDI-II | Non-significant | | |  |
|  |  |  |  |  |  | |  | |  |  | | |  |
|  |  | Income | Categorized as a) Low (< $500); b) Moderate (500-1000); and c) High (>1000). | Actual |  | | NA | |  | Non-significant | | |  |
|  |  |  |  |  |  | |  | |  |  | | |  |

**Table 5 (Continued)**

*The Main Outcomes of the Studies Included in the Systematic Review*

| Study ID | Quality Assessment | SEC  Variable | Measurement  Method | Actual / Perceived | | Anxiety    Inventory Outcomes | | Depression    Inventory Outcomes | | | Note | | |
| --- | --- | --- | --- | --- | --- | --- | --- | --- | --- | --- | --- | --- | --- |
|  |  |  |  |  |  | |  |  | |  | |  | |
| #28 | 8 | Education | Categorized as a) Tertiary; b) Primary and below; and c) Secondary school | Actual | GAD-7 | | Non-significant | | PHQ-9 | Non-significant | | |  |
|  |  |  |  |  |  | |  | |  |  | | |  |
|  |  | Job loss (Covid period) | Categorized as a) Not affected or affected to a less extent; and b) Very much affected | Perceived |  | | Non-significant | |  | Job loss significantly predict higher depression (aOR = 2.88, p < .001) | | |  |
|  |  |  |  |  |  | |  | |  |  | | |  |
|  |  | SES | Assessed using a 9-asset index, which included questions on items available in people’s homes, e.g., bicycle, television, motorbike, refrigerator, mobile phone, radio, motor vehicle, and internet connectivity. | Actual |  | | Non-significant | |  | Higher SES significantly predict lower depression (aOR = 0.83, p = .041) | | |  |
|  |  |  |  |  |  | |  | |  |  | | |  |
|  |  | Ability to pay utilities (Covid period) | Categorized as a) Not affected or affected to a less extent; and b) Very much affected | Perceived |  | | Ability significantly predict lower anxiety (aOR = 0.34, p = .016) | |  | Ability significantly predict lower depression (aOR = 0.45, p = .026) | | |  |
|  |  |  |  |  |  | |  | |  |  | | |  |
|  |  | Ability to repay loans affected (Covid period) | Categorized as a) Not affected or affected to a less extent; and b) Very much affected | Perceived |  | | Ability significantly predict lower anxiety (aOR = 0.48, p = .024) | |  | Non-significant | | |  |
|  |  |  |  |  |  | |  | |  |  | | |  |
| #14 | 8 | Economic worries | 2-items inventory measured on 3-point Likert scale (0 = Minimum and 2 = Maximum): E.g., "I am afraid that I may experience serious economic problems as a result of COVID-19" | Perceived | GAD-7 | | Economic worries were associated with higher anxiety in Clients only (OR = 1.85, p < .01). Non-significant for Staffs | | PHQ-9 | Economic worries were associated with higher depression in Clients (OR = 2.28, p < .01) as well as Staffs (OR = 2.05, p < .01). | | |  |
|  |  |  |  |  |  | |  | |  |  | | |  |
| #7 | 8 | Need additional occupation | Yes / No | Perceived | NA | | NA | | PHQ-9 | Participants that need an additional occupation were associated with higher depression (β = .607, p < .037). | | |  |
|  |  |  |  |  |  | |  | |  |  | | |  |
|  |  | Financial existential fears | 0 = No financial existential fears to 100 = Very severe financial existential fears. | Perceived |  | | NA | |  | Non-significant | | |  |

**Table 5 (Continued)**

*The Main Outcomes of the Studies Included in the Systematic Review*

| Study ID | Quality Assessment | SEC  Variable | Measurement  Method | Actual / Perceived | | Anxiety    Inventory Outcomes | | Depression    Inventory Outcomes | | | Note | | |
| --- | --- | --- | --- | --- | --- | --- | --- | --- | --- | --- | --- | --- | --- |
|  |  |  |  |  |  | |  |  | |  | |  | |
| #4 | 8 | Education | Categorized as a) High school or below; b) Some college; c) Bachelors; and d) Graduate degree | Actual | GAD-2 | | Anxiety prevalence was significantly higher among those with lesser education, ranging from 46.7% for those with a high school degree or less to 34.7% for respondents with a graduate degree. | | PHQ-2 | Depression declined with greater education, with prevalence rates dropping from 43% for those with a high school degree or less to 25.2% for respondents with a graduate degree. | | |  |
|  |  |  |  |  |  | |  | |  |  | | |  |
| #243 | 8 | COVID-19 related economic stress | Categorized as a) No impact; b) Low impact; and (c) High impact | Perceived | GAD-7 | | This variable was used in T2 only | | PHQ-9 | This variable was used in T2 only | | | Please see Table 4 for T2 |
| #227 | 8 | Financial stress due to COVID-19 | 5-point Likert scale (1 = No financial stress and 5 = Significant financial stress) | Perceived | GAD-7 | | Financial stress due to COVID-19 was associated with higher anxiety symptoms  (β = .122, p < .001) | | PHQ-9 | Financial stress due to COVID-19 was associated with higher depressive symptoms (β = .117, p < .001) | | |  |
|  |  |  |  |  |  | |  | |  |  | | |  |
| #217 | 8 | Income (annual) | Categorized (in KRW) as a) 6–24 million; b) 24–48 million; c) 48–72 million; and d) ≥72 million | Actual | GAD-2 | | Non-significant | | PHQ-9 | Non-significant | | |  |
|  |  |  |  |  |  | |  | |  |  | | |  |
| #214 | 8 | Income loss | US: Yes / No  Israel: 5-point Likert scale (from no income loss to extreme income loss) | Actual | GAD-7 (US)  PROMIS (Israel) | | Income loss was associated with higher anxiety for Israel (β = .08, p < .001). But non-significant for US.. | | PHQ-2 (US)  PROMIS (Israel) | Income loss was associated with higher depression for Israel (β = .08, p < .001). But non-significant for US. | | | Please see Table 4 for T2 |
|  |  |  |  |  |  | |  | |  |  | | |  |
|  |  | Covid-19 Financial worries | Both US and Israel used 5-point Likert scale (from not at all to a great deal) | Perceived |  | | NA | |  | Higher financial worries were associated with higher depression for US (β = .17, p < .001) and for Israel (β = .15, p < .001) | | |  |
|  |  |  |  |  |  | |  | |  |  | | |  |
|  |  | Pre-COVID-19 income | US: Annual income in bracket  Israel: 5-point Likert scale (1 = Considerably below average and 5 = Considerably above average) | Actual (US)  Perceived (Israel) |  | | This variable is not used in T2 | |  | This variable is not used in T2. | | |  |
|  |  |  |  |  |  | |  | |  |  | | |  |
|  |  |  |  |  |  | |  | |  |  | | |  |
|  |  |  |  |  |  | |  | |  |  | | |  |

**Table 5 (Continued)**

*The Main Outcomes of the Studies Included in the Systematic Review*

| Study ID | Quality Assessment | SEC  Variable | Measurement  Method | Actual / Perceived | | Anxiety    Inventory Outcomes | | Depression    Inventory Outcomes | | | Note | | |
| --- | --- | --- | --- | --- | --- | --- | --- | --- | --- | --- | --- | --- | --- |
|  |  |  |  |  |  | |  |  | |  | |  | |
| #211 | 8 | Health-related socioeconomic risks (HRSRs) | Defined by the Centers for Medicare & Medicaid Services Accountable Health Communities (AHC) screening tool (food insecurity, housing stability, utilities difficulties, transportation difficulties, and IPV). | Perceived | GAD-7 | | The odds of anxiety in the early phase of the pandemic was all significantly higher among women with pre-pandemic HRSRs (p < .01). For every additional pre-pandemic HRSR, the odds of anxiety increased by 1.9. | | PHQ-2 | The odds of depression in the early phase of the pandemic was all significantly higher among women with pre-pandemic HRSRs (p < .01). For every additional pre-pandemic HRSR, the odds of early pandemic depression increased by a factor of 1.9. | | |  |
|  |  |  |  |  |  | |  | |  |  | | |  |
| #208 | 8 | Job loss | Yes / No | Actual | GAD-7 | | Non-significant | | PHQ-9 | Non-significant | | |  |
|  |  |  |  |  |  | |  | |  |  | | |  |
|  |  | Income loss | Categorized as: a) Monthly income loss of < 50%; b) Monthly income loss of >=50%; and c) No loss of income | Actual |  | | Participants with a monthly income loss of 50% or more had a significant higher anxiety symptoms (aOR = 1.42, p = .035). | |  | Non-significant | | |  |
|  |  |  |  |  |  | |  | |  |  | | |  |
|  |  | Education | Categorized as a) Illiterate/ primary school/ junior high school; b) Senior high school/diploma/high vocational; and c) Bachelor's degree/ higher education | Actual |  | | Non-significant | |  | Non-significant | | |  |
|  |  |  |  |  |  | |  | |  |  | | |  |
|  |  | Having debt | Yes / No | Actual |  | | Non-significant | |  | Non-significant | | |  |
|  |  |  |  |  |  | |  | |  |  | | |  |
|  |  | Financial problems | Yes / No | Actual |  | | Financial problem was associated with higher anxiety symptoms (aOR = 2.00, p < .001). | |  | Financial problem was associated with higher depressive symptoms (aOR = 1.84, p < .001). | | |  |
|  |  |  |  |  |  | |  | |  |  | | |  |
| #200 | 8 | Education | Categorized as a) Higher secondary; and b) graduate/above | Actual | GAD-7 | | Non-significant | | PHQ-9 | The possibilities of having depression were 2.62 times more with high academic qualification compared to low educational background | | |  |
|  |  |  |  |  |  | |  | |  |  | | |  |
|  |  | Economic status | Categorized as a) Low; b) Medium; and c) High | Perceived |  | | The odds of having anxiety was 0.077 times lower in the low economic background than high economic class, 0.048 times lower in medium economic class than high economic class, | |  | Non-significant | | |  |
|  |  |  |  |  |  | |  | |  |  | | |  |

**Table 5 (Continued)**

*The Main Outcomes of the Studies Included in the Systematic Review*

| Study ID | Quality Assessment | SEC  Variable | Measurement  Method | Actual / Perceived | | Anxiety    Inventory Outcomes | | Depression    Inventory Outcomes | | | Note | | |
| --- | --- | --- | --- | --- | --- | --- | --- | --- | --- | --- | --- | --- | --- |
|  |  |  |  |  |  | |  |  | |  | |  | |
| #195 | 11 | Race | Black versus Non-black | Actual | PHQ-4 | | Black Americans were significantly less likely than non-Black Americans to have probable anxiety (aOR = 0.474; p < .001) | | PHQ-4 | Black Americans were significantly less likely than non-Black Americans to have probable depression (aOR = 0.522; p < .001) | | | Please see Table 4 for T2 |
|  |  |  |  |  |  | |  | |  |  | | |  |
| #192 | 8 | Negative financial change due to the pandemic | Yes / No | Perceived | STAI-6 | | Negative change in financial status were associated with higher odds of reporting anxiety (OR = 1.22, p < .01) | | PHQ-9 | Negative change in financial status were associated with higher odds of reporting depression (OR = 1.30, p < .001) | | |  |
|  |  |  |  |  |  | |  | |  |  | | |  |
| #185 | 8 | Education | Categorized as a) Less than Bachelor; and b) More than Bachelor | Actual | PHQ-4 | | Having bachelor degree and above was associated with lower prevalence rate of anxiety (aPR = 0.86, p = .02) | | PHQ-4 | Having bachelor degree and above was associated with lower prevalence rate of depression (aPR = 0.86, p = .02) | | |  |
|  |  |  |  |  |  | |  | |  |  | | |  |
|  |  | Satisfaction with economical resources | Categorized as a) Not much adequate; and b) Very adequate | Perceived |  | | Very adequate resources was associated with lower prevalence rate of anxiety (aPR = 0.73, p < .001) | |  | Very adequate resources was associated with lower prevalence rate of depression (aPR = 0.73, p < .001) | | |  |
|  |  |  |  |  |  | |  | |  |  | | |  |
| #164 | 8 | Education | Categorised as a) Basic level (primary and secondary); b) Medium level (baccalaureate and technical education); and c) High level (university studies) | Actual | STAI-S | | Non-significant | | NA | NA | | |  |
|  |  |  |  |  |  | |  | |  |  | | |  |
|  |  | Employment status | Categorised as a) Self-employment; b) Employment; c) Unemployment; d) Homemaker; and e) Student | Actual |  | | Non-significant | |  | NA | | |  |
|  |  |  |  |  |  | |  | |  |  | | |  |
|  |  | Job loss | Yes / No | Actual |  | | Non-significant | |  | NA | | |  |
|  |  |  |  |  |  | |  | |  |  | | |  |
|  |  | Reduced working hours | Yes / No | Actual |  | | Reduced working hours (β = 4.07, p = 0.003) associated with greater anxiety | |  | NA | | |  |
|  |  |  |  |  |  | |  | |  |  | | |  |
|  |  | Reduced income | Yes / No | Actual |  | | Reduced income (β = 2.66, p = 0.025) associated with greater anxiety | |  | NA | | |  |
|  |  |  |  |  |  | |  | |  |  | | |  |
|  |  |  |  |  |  | |  | |  |  | | |  |

**Table 5 (Continued)**

*The Main Outcomes of the Studies Included in the Systematic Review*

| Study ID | Quality Assessment | SEC  Variable | | Measurement  Method | Actual / Perceived | | | Anxiety    Inventory Outcomes | | | Depression    Inventory Outcomes | | | Note | | |
| --- | --- | --- | --- | --- | --- | --- | --- | --- | --- | --- | --- | --- | --- | --- | --- | --- |
|  |  |  | |  |  | |  | |  | |  | |  | |  | |
| #156 | 8 | Income (monthly family) | | Categorized as a) <5000 BDT; b) 5000-10000 BDT; and c) >10000 BDT | Actual | | NA | | NA | | | GDS-15 | Participants in the highest income category (>10,000 BDT) had nearly 60% lower odds of depressive symptoms than those in the lowest income bracket (aOR=0.42, p=.004) | | |  |
|  |  |  | |  |  | |  | |  | | |  |  | | |  |
|  |  | Financial dependency on family for living | | Yes / No | Actual | |  | | NA | | |  | Financial dependency, as indicated by dependency on family members (aOR=1.64, p=.011) was associated with higher odds of depressive symptoms | | |  |
|  |  |  | |  |  | |  | |  | | |  |  | | |  |
|  |  | Difficulty in earning during COVID-19 | | Yes / No | Perceived | |  | | NA | | |  | Non-significant | | |  |
|  |  |  | |  |  | |  | |  | | |  |  | | |  |
|  |  | Received financial support during COVID-19 | | Yes / No | Actual | |  | | NA | | |  | Receiving financial support during the pandemic (aOR: 1.46, p=.047) was associated with higher odds of depressive symptoms | | |  |
|  |  |  | |  |  | |  | |  | | |  |  | | |  |
| #152 | 8 | Financial situation | | Categorized as a) Deteriorated;  b) Stable; and c) Improved | Perceived | | HAD Anxiety scale | | Subjects' deterioration in financial situation significantly predicted higher anxiety (p < .001, β = -.10). | | HAD Depression scale | | Subjects' deterioration in financial situation significantly predicted higher depression (p < .001, β = -.07). | | |  |
|  |  |  | |  |  | |  | |  | |  | |  | | |  |
|  |  | Education | | Categorized as a) First year of bachelor’s degree;  b) Second or third year bachelor’s; c) Master’s degree; and d) Post-master | Actual | |  | | Compared with subjects in their first year of bachelor's degree, subjects in their second or third year of bachelor's degree have significantly higher anxiety (p < .05, β = .03). | | |  | Compared with subjects in their first year of bachelor's degree, subjects with master's degree have significantly higher depression (p < .05, β = .04). | | |  |
|  |  |  | |  |  | |  | |  | | |  |  | | |  |
| #150 | 11 | Financial stress | | Financial stress section of UCLA Life Stress Interview | Unsure | | NA | | NA | BDI-II for mothers, CES-D for adolescents | | | This variable is used in T2 only | | | Please see Table 4 for T2 |
|  |  |  |  | | |  |  | |  | | |  |  | | |  |

**Table 5 (Continued)**

*The Main Outcomes of the Studies Included in the Systematic Review*

| Study ID | Quality Assessment | SEC  Variable | Measurement  Method | Actual / Perceived | | Anxiety    Inventory Outcomes | | Depression    Inventory Outcomes | | | Note | | |
| --- | --- | --- | --- | --- | --- | --- | --- | --- | --- | --- | --- | --- | --- |
|  |  |  |  |  |  | |  |  | |  | |  | |
| #144 | 8 | Economic risk | 5-point Likert scale (1=Very low and 5=Very high).to the question: “To what extent do you think you will suffer financially from the Coronavirus crisis?' | Perceived | GAD-7 | | Financial risk was a significant predictor of higher anxiety across all the samples. | | NA | NA | | |  |
|  |  |  |  |  |  | |  | |  |  | | |  |
| #130 | 11 | Pre-crisis socioeconomic status: Relative financial status | Categorized as a) Below average; b) Average; and c) Above average. | Perceived | NA | | NA | | MDI | This variable is used in T2 only | | | Please see Table 4 for T2 |
|  |  |  |  |  |  | |  | |  |  | | |  |
|  |  | Pre-crisis socioeconomic status: Difficulty paying usual bills | Categorized as a) Easy or very easy; b) Fairly easy; and c) Rather difficult or difficult | Perceived |  | | NA | |  | This variable is used in T2 only | | |  |
|  |  |  |  |  |  | |  | |  |  | | |  |
|  |  | Pre-crisis socioeconomic status: Education | Categorized as a) Compulsory schooling (ISCED 2; 9 years); b) Secondary school diploma (ISCED 34; 12-13 years); c) Apprenticeship (ISCED 35; 12-13 years); d) Bachelor's degree (ISCED 6; 15 years); and e) Master's degree (ISCED 7; 17 years). | Actual |  | | NA | |  | This variable is used in T2 only | | |  |
|  |  |  |  |  |  | |  | |  |  | | |  |
|  |  | Changes in work situation due to COVID-19 | Categorized as a) No change; b) Job loss; c) Partial unemployment; and d) Losing money as self-employed. | Actual |  | | NA |  | | This variable is used in T2 only | | |  |
|  |  |  |  |  |  | |  |  | |  | | |  |

**Table 5 (Continued)**

*The Main Outcomes of the Studies Included in the Systematic Review*

| Study ID | Quality Assessment | SEC  Variable | | Measurement  Method | Actual / Perceived | | | Anxiety    Inventory Outcomes | | | Depression    Inventory Outcomes | | | | Note | | |
| --- | --- | --- | --- | --- | --- | --- | --- | --- | --- | --- | --- | --- | --- | --- | --- | --- | --- |
|  |  |  | |  |  | |  | |  | |  | |  | | |  | |
| #101 | 9 | Job loss | | Yes / No | Actual | | GAD-7 | | This variable is used in T2 only | | | PHQ-9 | This variable is used in T2 only | | | | Please see Table 4 for T2 |
|  |  |  | |  |  | |  | |  | | |  |  | | | |  |
|  |  | Income loss | | Yes / No | Actual | |  | | This variable is used in T2 only | | |  | This variable is used in T2 only | | | |  |
|  |  |  | |  |  | |  | |  | | |  |  | | | |  |
|  |  | Worries about losing job / employment | | Yes / No | Perceived | |  | | This variable is used in T2 only | | |  | This variable is used in T2 only | | | |  |
|  |  |  | |  |  | |  | |  | | |  |  | | | |  |
|  |  | Worries about finances | | Yes / No | Perceived | |  | | This variable is used in T2 only | | |  | This variable is used in T2 only | | | |  |
|  |  |  | |  |  | |  | |  | | |  |  | | | |  |
| #97 | 8 | Job position | | Admin / Not admin | Actual | | K6 | | K6 scores were significantly lower for administrative workers | | K6 | | K6 scores were significantly lower for administrative workers | | | |  |
|  |  |  | |  |  | |  | |  | |  | |  | | | |  |
|  |  | Education | | Categorized as a) Junior high / High school; b) Vocational or junior college; and c) University or graduate school | Actual | |  | | Non-significant | | |  | Non-significant | | | |  |
|  |  |  | |  |  | |  | |  | | |  |  | | | |  |
|  |  | Income (Annual in ‘000 JPY) | | Categorized as a) <150; b) 150-199; c) 200-299; d) 300-399; e) 400-499; f) 500-699; g) 700-999; and h) >=1000 | Actual | |  | | K6 scores were significantly higher for higher income |  | | | K6 scores were significantly higher for higher income | | | |  |
|  |  |  | |  |  | |  | |  |  | | |  | | | |  |
| #93 | 7 | Education | Categorized as a) No qualification; b) Primary school; c) Vocational training and equivalent; d) Secondary school; and e) University | | Actual | | HADS-14 | | Lower education were independently associated with higher anxiety symptoms | | | HADS-14 | | Higher education were independently associated with lower depressive symptom | | |  |
|  |  |  |  | |  | |  | |  | | |  | |  | | |  |
|  |  | Employment status | Categorized as a) Unemployed/Retired; b) Students; and c) Employed | | Actual | |  | | Non-significant | | |  | Non-significant | | | |  |
|  |  |  |  | |  | |  | |  | | |  |  | | | |  |
|  |  | Income | Categorized as a) <=USD105 ; b) USD105-210; c) USD210-315; d) USD315-420; and e) > USD420 | | | Actual |  | | Non-significant | | |  | Non-significant | | | |  |
|  |  |  |  | | |  |  | |  | | |  |  | | | |  |

**Table 5 (Continued)**

*The Main Outcomes of the Studies Included in the Systematic Review*

| Study ID | Quality Assessment | SEC  Variable | Measurement  Method | Actual / Perceived | | Anxiety    Inventory Outcomes | | Depression    Inventory Outcomes | | | Note | | |
| --- | --- | --- | --- | --- | --- | --- | --- | --- | --- | --- | --- | --- | --- |
|  |  |  |  |  |  | |  |  | |  | |  | |
| #90 | 8 | Economic status | Categorized as a) Higher than average; b) Average; and c) Lower than average | Perceived | DASS-21 | | Participants’ anxiety level was positively related to economic status (B = 0.057, p < 0.001) – lower the SES, higher the anxiety | | DASS-21 | Participants’ depression level was positively related to economic status (B = 0.068, p < 0.001) lower the SES, higher the depression | | |  |
|  |  |  |  |  |  | |  | |  |  | | |  |
| #89 | 11 | Income (annual pre-Covid) | Categorized as a) <10k;b) 10-20k; c) 20-30k; d) 30-40k; e) 40-50k; f) 50-60k; g) 60-70k; h) 70-80k; i) 80-90k; j) 90-100k; k) 100-150k; and l) >150k | Actual | SAI | | Subjects with lower income were significantly related to having higher anxiety (β = -.32, p < 0.001). | | SDS | Subjects with lower income were significantly related to higher depression (β = -.37, p< 0.001). | | | Please see Table 4 for T2 |
|  |  |  |  |  |  | |  | |  |  | | |  |
| #80 | 7 | Job loss | Categorized as a) Yes; b) No, but reduced hours; c) No, but expected; and d) No - in response to the question: "Have you lost your job due to the COVID-19 pandemic?" | Actual | PHQ-4 | | Non-significant | | PHQ-4 | Non-significant | | |  |
|  |  |  |  |  |  | |  | |  |  | | |  |
|  |  | Financial stress | Categorized as a) No stress; b) A little; c) Moderate; d) Considerable; and e) Extreme - in response to the question: "To what extent has the COVID-19 pandemic put financial stress on you?" | Perceived |  | | Non-significant | |  | Non-significant | | |  |
|  |  |  |  |  |  | |  | |  |  | | |  |
|  |  | Education | Unclear | Actual |  | | Non-significant |  | | Non-significant | | |  |
|  |  |  |  |  |  | |  |  | |  | | |  |

**Table 5 (Continued)**

*The Main Outcomes of the Studies Included in the Systematic Review*

| Study ID | Quality Assessment | SEC  Variable | | Measurement  Method | Actual / Perceived | | Anxiety    Inventory Outcomes | | | Depression    Inventory Outcomes | | | | Note | | |
| --- | --- | --- | --- | --- | --- | --- | --- | --- | --- | --- | --- | --- | --- | --- | --- | --- |
|  |  |  | |  |  |  | |  | |  | |  | | |  | |
| #78 | 7 | Education | | Highest years of education in the household | Actual | SC-90-R | | This variable is used in T2 only | | | SC-90-R | This variable is used in T2 only | | | | Please see Table 4 for T2 |
|  |  |  | |  |  |  | |  | | |  |  | | | |  |
|  |  | Income (per capita) | | Monthly household income per capita (2017 USD) | Actual |  | | This variable is used in T2 only | | |  | This variable is used in T2 only | | | |  |
|  |  |  | |  |  |  | |  | | |  |  | | | |  |
|  |  | Asset index | | Unclear | Unclear |  | | This variable is used in T2 only | | |  | This variable is used in T2 only | | | |  |
|  |  |  | |  |  |  | |  | | |  |  | | | |  |
|  |  | Beneficiary of conditional cash transfer | | Unclear | Unclear |  | | This variable is used in T2 only | | |  | This variable is used in T2 only | | | |  |
|  |  |  | |  |  |  | |  | | |  |  | | | |  |
|  |  | Job or Income loss | | Unclear | Unclear |  | | This variable is used in T2 only | |  | | This variable is used in T2 only | | | |  |
|  |  |  | |  |  |  | |  | |  | |  | | | |  |
| #76 | 8 | Education | | Categorized as a) Mandatory school; b) High school; c) Vocational; d) Short high; e) Medium high; f) Long Higher; and g) Not reported | Actual | GAD-7 | | Non-significant | | NA | | NA | | | |  |
|  |  |  | |  |  |  | |  | | |  |  | | | |  |
|  |  | Employment status | | Categorized as a) Student; b) Working; c) Long-term sick leave; d) Unemployed; e) Wage-subsized employment; f) Disability pension; g) Age retirement; h) Outside work market; and i) Not reported | Actual |  | | ORs were significantly increased only among patients on disability pension (OR = 2.44) and age retirement (OR = 1.72) | | |  | NA | | | |  |
|  |  |  | |  |  |  | |  |  | | |  | | | |  |
| #57 | 7 | Place of residence | | Categorized as a) Village; b) Town; c) City; and d) Agglomeration | Actual | GAD-7 | | Non-significant | PHQ-9 | | | The only significant difference was observed between towns and villages (d = 0.14), with a higher average for town | | | |  |
|  |  |  |  | |  |  | |  | | |  | |  | | |  |
|  |  | Education | Categorized as a) Bachelor; b) Master; c) Postgraduate; and d) Doctoral - recoded as Bachelor = 0 and Master of higher = 1 | | Actual |  | | Non-significant | | |  | | Depression was significantly lower among participants with master’s degrees and higher among participants with bachelor’s degrees | | |  |
|  |  |  |  | |  |  | |  | | |  |  | | | |  |

**Table 5 (Continued)**

*The Main Outcomes of the Studies Included in the Systematic Review*

| Study ID | Quality Assessment | SEC  Variable | | Measurement  Method | Actual / Perceived | | Anxiety    Inventory Outcomes | | | | | Depression    Inventory Outcomes | | | | Note | | |
| --- | --- | --- | --- | --- | --- | --- | --- | --- | --- | --- | --- | --- | --- | --- | --- | --- | --- | --- |
|  |  |  | |  |  |  | | | |  | |  | |  | | |  | |
| #47 | 8 | Education | | Categorized as a) University; and b) Postgraduate degree | Actual | DASS-21 | | | | Non-significant | | | DASS-21 | Non-significant | | | |  |
|  |  |  | |  |  |  | | | |  | | |  |  | | | |  |
|  |  | Financial concern | | 11-point Likert scale (0 = The worst financial state and 10 = The best financial state) | Perceived |  | | | | Non-significant | |  | | Non-significant | | | |  |
|  |  |  | |  |  |  | | | |  | |  | |  | | | |  |
|  |  | Financial state | | 11-point Likert scale (0 = The worst financial state and 10 = The best financial state) | Perceived |  | | | | Non-significant | |  | | Non-significant | | | |  |
|  |  |  | |  |  |  | | | |  | |  | |  | | | |  |
| #44 | 8 | Financial stability | | Financially stable is categorized as “comfortable”; financially unstable is categorized as “cannot make ends meet” or “have just enough to get along” | Perceived | GAD-7, SHAI | | | | Non-significant | | PHQ-9 | | Non-significant | | | |  |
|  |  |  | |  |  |  | | | |  | | |  |  | | | |  |
| #35 | 8 | Education | | Categorized as a) Secondary school and lower; b) High school; and c) University and higher" | Actual | GAI | | | Compared to secondary school and below education, an education level of university or higher significantly predicted lower anxiety (p < .001, OR = 0.238). | | | | NA | NA | | | |  |
|  |  |  | |  |  |  | | |  | |  | | |  | | | |  |
|  |  | Employment status | | Categorized as a) Working; and  b) Not working | Actual |  | | | | Non-significant |  | | | NA | | | |  |
|  |  |  |  | |  |  | | | |  | | |  | |  | | |  |
|  |  | Financial loss | Yes / No | | Actual |  | | Compared to subjects with no financial loss, subjects with financial loss significantly predicted higher anxiety (p < .05, OR = 2.310). | | | | |  | | NA | | |  |
|  |  |  |  | |  |  | |  | | | | |  |  | | | |  |
|  |  | Financial support | Yes / No | | Actual |  | | | | Non-significant | | |  | NA | | | |  |
|  |  |  |  | |  |  | | | |  | | |  |  | | | |  |

**Table 5 (Continued)**

*The Main Outcomes of the Studies Included in the Systematic Review*

| Study ID | Quality Assessment | SEC  Variable | Measurement  Method | Actual / Perceived | | Anxiety    Inventory Outcomes | | Depression    Inventory Outcomes | | | Note | | |
| --- | --- | --- | --- | --- | --- | --- | --- | --- | --- | --- | --- | --- | --- |
|  |  |  |  |  |  | |  |  | |  | |  | |
| #26 | 8 | COVID-19 financial hardship | Binary response (Yes/No) to the question: “Have you lost your job or seen a reduction in your income as a result of the coronavirus?” | Actual | NA | | NA | | PHQ-9 | There was a statistically significant effect of COVID-19 job loss or reduction in income on levels of depression symptoms after controlling for covariates, F (1, 4754) =187.37, p <.01, d =0.25 (indicating a small effect). | | |  |
|  |  |  |  |  |  | |  | |  |  | | |  |
| #24 | 9 | Education | Categorized as a) No formal education; b) Fundamental education; c) Secondary education; d) University degree; and e) Master or above | Actual | GAD-7 | | Secondary education was significantly associated with lower baseline anxiety  (β = -1.20, p < .05). University degree of master and above was significantly associated with lower baseline anxiety  (β =-1.26, p <.05). Fundamental education and university degree did not significantly predict baseline anxiety. | | CES-D | There were significant associations between fundamental education and lower baseline depression (β=-3.60, p <.05), secondary education and lower baseline depression (β=-3.75, p <.01), university degree and lower baseline depression (β=-3.11, p <.01), and university degree of master and above and lower baseline depression (β=-2.72, p <.05). | | | Please see Table 4 for T2 |
|  |  | Employment status | Categorized as a) Full-time employed; b) In retirement or early retirement; c) In vocational training/retraining/education; d) Looking after home or family; e) Other status; f) Parental leave; g) Part-time employed; h) Permanently sick or disabled; i) Self-employed or working for own family business; and j) Unemployed | Actual |  | | In retirement or early retirement was significantly associated with lower baseline anxiety (β=-1.10, p <.01). Unemployed was significantly associated with higher baseline anxiety (β=1.77, p <.01). In vocational training/retraining/education, looking after home or family, other status, parental leave, part-time employed, permanently sick or disabled, and self-employed or working for own family business did not significantly predict baseline anxiety. | |  | In retirement or early retirement was significantly associated with lower baseline depression (β-1.87, p <.05). Permanently sick or disabled was significantly associated with higher baseline depression (β=5.20, p <.05). In vocational training /retraining / education, looking after home or family, other status, parental leave, part-time employed, self-employed or working for own family business, and unemployed did not significantly predict baseline depression. | | |  |
|  |  |  |  |  |  | |  | |  |  | | |  |
|  |  | Income | Categorized as a) 0-25000 Euros; b) 25000-75000; c) 75000-150000; d) >150000 Euros; and e) No answer | Actual |  | | Non-significant | |  | Non-significant | | |  |
|  |  |  |  |  |  | |  | |  |  | | |  |

**Table 5 (Continued)**

*The Main Outcomes of the Studies Included in the Systematic Review*

| Study ID | Quality Assessment | SEC  Variable | Measurement  Method | Actual / Perceived | | Anxiety    Inventory Outcomes | | Depression    Inventory Outcomes | | | Note | | |
| --- | --- | --- | --- | --- | --- | --- | --- | --- | --- | --- | --- | --- | --- |
|  |  |  |  |  |  | |  |  | |  | |  | |
| #20 | 8 | SES | Categorized as a) Lower SES (<15000 BDT); b) Middle SES (15 000-30000 BDT); and c) Upper SES (>30000 BDT) | Actual | GAD-7 | | Lower SES (β =.10, p <.001) and middle SES (β =.07, p =.012) were associated with higher anxiety | | PHQ-9 | Lower SES (β =.06, p =.021) and middle SES (β =.06, p =.043) were associated with higher depression | | |  |
|  |  |  |  |  |  | |  | |  |  | | |  |
|  |  | Place of residence | Categorized as a) Rural; b) Suburban; and c) Urban | Actual |  | | Non-significant | |  | Non-significant | | |  |
|  |  |  |  |  |  | |  | |  |  | | |  |
| #5 | 8 | Education | Categorized as a) High school/GED or lower; b) Some college; and c) Bachelor or higher" | Actual | GAD-2 | | This variable is used in T2 only | | PHQ-2 | This variable is used in T2 only | | | Please see Table 4 for T2 |
|  |  |  |  |  |  | |  | |  |  | | |  |
|  |  | Income (household) | Categorized as a) Less than $49,000;  b) $50,000-$99,000;  c) $100,000-$149,000;  d) $150,000 or higher; and  e) Unknown" | Actual |  | | This variable is used in T2 only | |  | This variable is used in T2 only | | |  |
|  |  |  |  |  |  | |  | |  |  | | |  |
| #437 | 8 | Education | Categorized as a) high school graduate/GED; b) Some college; c) College graduate or more | Actual | GAD-7 | | Non-significant | | PHQ-9 | Non-significant | | |  |
|  |  | Household income | Categorized as a) $0-$19,999; b) $20,000-$44,999; c) $45,000-$74,999; d) >$75,000 | Actual |  | | Non-significant | |  | Non-significant | | |  |
|  |  |  |  |  |  | |  | |  |  | | |  |
|  |  | Household savings | Categorized as a) < $5,000; b) >$5,000 | Actual |  | | Having less than $5,000 in household savings increased the odds of anxiety symptoms (OR = 1.30, p = <.05). | |  | Non-significant | | |  |
|  |  |  |  |  |  | |  | |  |  | | |  |
|  |  |  |  |  |  | |  | |  |  | | |  |
|  |  |  |  |  |  | |  | |  |  | | |  |

**Table 5 (Continued)**

*The Main Outcomes of the Studies Included in the Systematic Review*

| Study ID | Quality Assessment | SEC  Variable | Measurement  Method | Actual / Perceived | | Anxiety    Inventory Outcomes | | Depression    Inventory Outcomes | | Note | |
| --- | --- | --- | --- | --- | --- | --- | --- | --- | --- | --- | --- |
|  |  |  |  |  |  | |  |  |  | |  |
| #436 | 7 | Financial insecurity | 4-point Likert scale that assess the extent to which a strain on financial resources had been a concern in the past week | Perceived | NA | | NA | CESD-10 | Financial insecurity predicted higher depressive symptoms (B = .22, p < .01). | |  |
|  |  |  |  |  |  | |  |  |  | |  |
|  |  | Resource scarcity | Binary response (Yes/No) to the question: “I was concerned about getting basic necessities such as food and supplies.” | Perceived |  | | NA |  | Resource scarcity predicted higher depressive symptoms (B = .39, p < .05). | |  |
|  |  |  |  |  |  | |  |  |  | |  |
|  |  | Household income | 5-point Likert scale (1 = less than $50,000 and 5 = $200,000 or more) | Actual |  | | NA |  | Lower household income predicted higher depressive symptoms (B = -.13, p < .05). | |  |
|  |  |  |  |  |  | |  |  |  | |  |
| #425 | 8 | Financial concern | Categorized as a) Not stressful at all; b) Somewhat stressful; c) Stressful; and d) Very stressful | Perceived | GAD-7 | | Higher financial concerns were associated with higher anxiety (OR = 1.90, p < .001). | NA | NA | |  |
|  |  |  |  |  |  | |  |  |  | |  |
| #418 | 8 | Occupation | Categorized as a) White-collar; and b) Blue-collar | Actual | DASS Anxiety | | Non-significant | DASS Depression | Non-significant | |  |
|  |  |  |  |  |  | |  |  |  | |  |
|  |  | Education | Categorized as a) Elementary school; b) High school; c) Bachelor; and d) University/Master/PhD | Actual |  | | Non-significant |  | Non-significant | |  |
|  |  |  |  |  |  | |  |  |  | |  |
|  |  | SES | Categorized as a) Below average; b) Average; and c) Above average | Perceived |  | | Above average SES was associated with lower anxiety (OR = 0.58, p < .05). |  | Average SES (OR = 0.55, p < .01) and above average SES (OR = 0.45, p < .01) were associated with lower depression. | |  |

**Table 5 (Continued)**

*The Main Outcomes of the Studies Included in the Systematic Review*

| Study ID | Quality Assessment | SEC  Variable | Measurement  Method | Actual / Perceived | | Anxiety    Inventory Outcomes | | Depression    Inventory Outcomes | | Note | |
| --- | --- | --- | --- | --- | --- | --- | --- | --- | --- | --- | --- |
|  |  |  |  |  |  | |  |  |  | |  |
| #417 | 8 | Education | Categorized as a) Primary school or below; b) Middle or high school; and c) College or above | Actual | NA | | NA | CESD-10 | Compared to an education level of primary school or below, middle or high school (β = -0.16, p < .01) and college or above (β = -0.16, p < .01) predicted lower depression. | |  |
|  |  |  |  |  |  | | NA |  |  | |  |
|  |  | Occupation | Categorized as a) Unemployed; b) Pensioner or retired; and c) Part-time or full-time employment | Actual |  | |  |  | Non-significant | |  |
|  |  |  |  |  |  | |  |  |  | |  |
|  |  | Household income | Categorized as a) Below average; b) Average; and c) Above average | Perceived |  | | NA |  | Compared to below average household income, average (β = -0.08, p < .05) and above average (β = -0.10, p < .01) household income predicted lower depression. | |  |
|  |  |  |  |  |  | |  |  |  | |  |
| #390 | 8 | Employment status | Categorized as a) Regular employees; b) Non-regular employees; c) Self-employed and others; and d) Not working | Actual | K6 | | Non-significant | NA | NA | |  |
|  |  |  |  |  |  | |  |  |  | |  |
|  |  | Education | Categorized as a) University or graduate school; b) Junior college; and c) High school or junior high school | Actual |  | | Compared to an education level of university or graduate school, education level of high school or junior high school was associated with higher odds of anxiety (aOR = 1.21, p < .05). |  | NA | |  |
|  |  |  |  |  |  | |  |  |  | |  |
|  |  | Annual household income | Categorized as a) <2mil yen; b) 2mil to <6mil yen; and c) 6mil yen or more | Actual |  | | Compared to an annual household income of 6mil yen or more, annual household incomes of less than 2mil yen (aOR = 1.67, p < .05) and 2mil to less than 6mil yen (aOR = 1.25, p < .05) were associated with higher odds of anxiety. |  | NA | |  |
|  |  |  |  |  |  | |  |  |  | |  |
|  |  | Financial concern | 5-point Likert scale (1 = Extremely worried and 5 = Not worried) to the question: “Impact on financial conditions such as income.” | Perceived |  | | Non-significant |  | NA | |  |
|  |  |  |  |  |  | |  |  |  | |  |

**Table 5 (Continued)**

*The Main Outcomes of the Studies Included in the Systematic Review*

| Study ID | Quality Assessment | SEC  Variable | Measurement  Method | Actual / Perceived | | Anxiety    Inventory Outcomes | | Depression    Inventory Outcomes | | Note | |
| --- | --- | --- | --- | --- | --- | --- | --- | --- | --- | --- | --- |
|  |  |  |  |  |  | |  |  |  | |  |
| #389 | 8 | Job loss | Binary response (Yes/No) to the question: “Have you lost your job during lockdown?” | Actual | HADS-A | | Job loss negatively predicted anxiety (β = -0.21, p < .01). | HADS-D | Job loss positively predicted depression (β = 0.23, p < .01). | |  |
|  |  |  |  |  |  | |  |  |  | |  |
|  |  | Employment status | Binary response (Yes/No) to the question: “Have you worked during lockdown?” | Actual |  | | Working status positively predicted anxiety (β = 0.18, p < .01). |  | Non-significant | |  |
|  |  |  |  |  |  | |  |  |  | |  |
| #380 | 11 | Employment status | Categorized as a) Working in Wave 1; b) Paid leave in Wave 1; c) Furloughed in Wave 1; d) Not employed in Wave 1; e) Paid leave in Wave 2; f) Furloughed in Wave 2; and g) Not employed in Wave 2 | Actual | NA | | NA | PHQ-2, CESD-10 | Being employed during Wave 1 was associated with decreased depression (OR = -0.20, p < .05). | | Please see Table 4 for T2 |
|  |  |  |  |  |  | |  |  |  | |  |
|  |  | Tertiary Education | Yes / No | Actual |  | | NA |  | Non-significant | |  |
|  |  |  |  |  |  | |  |  |  | |  |
| #376 | 7 | Education | Categorized as a) Primary/Secondary/High school diploma; and b) Bachelor/Master/PhD | Actual | STAI | | Non-significant | BDI-II | Lower education level was associated with higher depression in students (B = 0.12, p < .05) and workers (B = 012, p < .05). | |  |
|  |  |  |  |  |  | |  |  |  | |  |
| #372 | 11 | Education | Years of education | Actual | PROMIS Anxiety | | This variable is used in T2 only | PHQ-8 | This variable is used in T2 only | | Please see Table 4 for T2 |
|  |  |  |  |  |  | |  |  |  | |  |
|  |  | Employment status | Categorized as a) Part-time; and b) Full-time | Actual |  | | This variable is used in T2 only |  | This variable is used in T2 only | |  |
|  |  |  |  |  |  | |  |  |  | |  |
|  |  | Financial well-being | Measured using the CFPB Financial Well-Being Scale | Perceived |  | | This variable is used in T2 only |  | This variable is used in T2 only | |  |
|  |  |  |  |  |  | |  |  |  | |  |

**Table 5 (Continued)**

*The Main Outcomes of the Studies Included in the Systematic Review*

| Study ID | Quality Assessment | SEC  Variable | Measurement  Method | Actual / Perceived | | Anxiety    Inventory Outcomes | | Depression    Inventory Outcomes | | Note | |
| --- | --- | --- | --- | --- | --- | --- | --- | --- | --- | --- | --- |
|  |  |  |  |  |  | |  |  |  | |  |
| #370 | 8 | Monthly household income | Categorized as a) <RM4,850 (B40); b) RM4,850-RM10,599 (M40); and c) RM10,600 and above (T20) | Actual | DASS Anxiety | | Compared to T20 people, B40 people have higher risk for anxiety (aOR = 1.03, p < .001), while M40 people have lower risk for anxiety (aOR = 0.92, p < .001). | DASS Depression | Compared to T20 people, B40 people (aOR = 0.90, p < .001) and M40 people (aOR = 0.81, p < .001) have lower risk for depression. | |  |
|  |  |  |  |  |  | |  |  |  | |  |
|  |  | Education | Categorized as a) Secondary and below; and b) Tertiary | Actual |  | | Compared to secondary and below education level, people with tertiary education have higher risk for anxiety (aOR = 1.80, p < .05). |  | Compared to secondary and below education level, people with tertiary education have higher risk for depression (aOR = 1.82, p < .05). | |  |
|  |  |  |  |  |  | |  |  |  | |  |
|  |  | Financial status | Categorized as a) Poor; b) Medium; and c) Good | Perceived |  | | Compared to good financial status, poor financial status (aOR = 1.28, p < .001) and medium financial status (aOR = 1.27, p < .001) have higher risk for anxiety. |  | Compared to good financial status, poor financial status (aOR = 2.63, p < .001) and medium financial status (aOR = 1.36, p < .001) have higher risk for depression. | |  |
|  |  |  |  |  |  | |  |  |  | |  |
| #360 | 8 | Education | Categorized as a) Junior college; b) Undergraduate; and c) Postgraduate | Actual | GAD-7 | | Compared to junior college students, undergraduates (OR = 2.12, p < .001) and postgraduates (OR = 2.64, p < .001) have higher risk for anxiety. | PHQ-9 | Compared to junior college students, undergraduates (OR = 1.61, p < .01) and postgraduates (OR = 1.77, p < .01) have higher risk for depression. | |  |
|  |  |  |  |  |  | |  |  |  | |  |
| #358 | 8 | Economic pressure | Categorized as a) None or little; b) Moderate; and c) Severe | Perceived | DASS Anxiety | | Compared to students with none or little economic pressure, students with moderate (B = 0.18, p < .05) and severe (B = 0.38, p < .05) economic pressure have higher risk for anxiety. | DASS Depression | Compared to students with none or little economic pressure, students with moderate (B = 0.15, p < .05) and severe (B = 0.32, p < .05) economic pressure have higher risk for depression. | |  |
|  |  |  |  |  |  | |  |  |  | |  |
| #355 | 8 | SES | Categorized as a) Low; b) Low-middle; c) Middle; d) High-middle; and e) High | Actual | NA | | NA | 6-item SES scale | Non-significant | |  |
|  |  |  |  |  |  | |  |  |  | |  |

**Table 5 (Continued)**

*The Main Outcomes of the Studies Included in the Systematic Review*

| Study ID | Quality Assessment | SEC  Variable | Measurement  Method | Actual / Perceived | | Anxiety    Inventory Outcomes | | Depression    Inventory Outcomes | | Note | |
| --- | --- | --- | --- | --- | --- | --- | --- | --- | --- | --- | --- |
|  |  |  |  |  |  | |  |  |  | |  |
| #354 | 11 | Economic adversity | Categorized as a) Faced with new health expenses; b) Experienced adversity but did not reduce food consumption; and c) Reduced food consumption as response to adversity | Actual | GAD-7 | | This variable is used in T2 only | PHQ-8 | This variable is used in T2 only | | Please see Table 4 for T2 |
|  |  |  |  |  |  | |  |  |  | |  |
|  |  | Employment status | Categorized as a) Did not work before pandemic but working now; b) Worked before pandemic and still working; and c) Worked before pandemic but no longer working | Actual |  | | This variable is used in T2 only |  | This variable is used in T2 only | |  |
|  |  |  |  |  |  | |  |  |  | |  |
| #347 | 8 | Education | Categorized as a) 8 years to A-levels; b) Professional college or bachelor; and c) Master or higher | Actual | NA | | NA | SCL-27 | Non-significant | |  |
|  |  |  |  |  |  | |  |  |  | |  |
|  |  | Job impact | Categorized as a) Home office; b) Reduction of hours; c) Unpaid leave; d) Overtime; e) Lost job; and f) No change | Actual |  | | NA |  | Non-significant | |  |
|  |  |  |  |  |  | |  |  |  | |  |
|  |  | Financial impact | Categorized as a) No impact; b) Slight; c) Moderate; d) Big; and e) Extreme | Perceived |  | | NA |  | Non-significant | |  |
|  |  |  |  |  |  | |  |  |  | |  |
| #340 | 7 | Food insecurity | Measured using the USDA Food Security Survey Module | Actual | NA | | NA | PHQ-4 | High level of food insecurity predicted depression (aPR = 1.4, p < .05). | |  |
|  |  |  |  |  |  | |  |  |  | |  |
|  |  | Housing insecurity | Categorized as a) Very worried; b) Somewhat worried; and c) Not worried at all | Perceived |  | | NA |  | People who are very worried about losing their house predicted depression (aPR = 1.3, p < .05). | |  |
|  |  |  |  |  |  | |  |  |  | |  |
|  |  |  |  |  |  | |  |  |  | |  |

**Table 5 (Continued)**

*The Main Outcomes of the Studies Included in the Systematic Review*

| Study ID | Quality Assessment | SEC  Variable | Measurement  Method | Actual / Perceived | | Anxiety    Inventory Outcomes | | Depression    Inventory Outcomes | | Note | |
| --- | --- | --- | --- | --- | --- | --- | --- | --- | --- | --- | --- |
|  |  |  |  |  |  | |  |  |  | |  |
| #336 | 8 | Education | Categorized as a) Less than university degree; and b) University degree | Actual | NA | | NA | CESD | Non-significant | |  |
|  |  | Income | Categorized as a) Less than $40,000; b) $40,000-$79,999; c) $80,000-$119,999; and d) $120,000 or more | Actual |  | | NA |  | Non-significant | |  |
|  |  |  |  |  |  | |  |  |  | |  |
|  |  | Employment Status | Categorized as a) Working from home; b) Laid off; and c) No change | Actual |  | | NA |  | Non-significant | |  |
|  |  |  |  |  |  | |  |  |  | |  |
|  |  | Financial concern | Categorized as a) Not at all to not very worried; b) Somewhat worried; and c) Very worried | Perceived |  | | NA |  | Compared to people who are not at all to not very financially worried, people who are somewhat worried (aOR = 3.14, p < .001) and very worried (aOR = 8.00, p < .001) have higher risk for depression. | |  |
|  |  |  |  |  |  | |  |  |  | |  |
| #334 | 11 | Annual household income | Categorized as a) < $79,999; and b) $80,000 or more | Actual | STAI-SF | | This variable is used in T2 only | CESD-10 | This variable is used in T2 only | | Please see Table 4 for T2 |
|  |  |  |  |  |  | |  |  |  | |  |
|  |  | COVID-19 impact on income or employment | Yes / No | Perceived |  | | This variable is used in T2 only |  | This variable is used in T2 only | |  |
|  |  |  |  |  |  | |  |  |  | |  |
| #323 | 8 | Education | Categorized as a) Completed high school; and b) Did not complete high school | Actual | DASS Anxiety | | People who did not complete high school had higher anxiety (b = 0.85, p < .001). | DASS Depression | People who did not complete high school had higher depression (b = 0.63, p < .01). | |  |
|  |  |  |  |  |  | |  |  |  | |  |
| #318 | 7 | Financial concern | 5-point Likert scale (1=Strongly disagree and 5=Strongly agree) to the question: “I am worried about my financial situation due to the COVID-19 crisis.” | Perceived | NA | | NA | EPDS | Women who were concerned about their financial situation have higher risk for depression (aOR = 2.23, p < .001). | |  |
|  |  |  |  |  |  | |  |  |  | |  |

**Table 5 (Continued)**

*The Main Outcomes of the Studies Included in the Systematic Review*

| Study ID | Quality Assessment | SEC  Variable | Measurement  Method | Actual / Perceived | | Anxiety    Inventory Outcomes | | Depression    Inventory Outcomes | | Note | |
| --- | --- | --- | --- | --- | --- | --- | --- | --- | --- | --- | --- |
|  |  |  |  |  |  | |  |  |  | |  |
| #313 | 8 | Employment status | Categorized as a) Unemployed; and b) Employed | Actual | BSI-18 | | Non-significant | BSI-18 | Non-significant | |  |
|  |  |  |  |  |  | |  |  |  | |  |
|  |  | Financial concern | Categorized as a) Financial; b) Family; c) Peer group; d) Religion; e) Health; f) Academic; and g) Leisure time to the question: “Has COVID-19 stressed you in any of the following areas of life?” | Perceived |  | | Non-significant |  | Non-significant | |  |
|  |  |  |  |  |  | |  |  |  | |  |
| #286 | 8 | Education | Categorized as a) Higher education; and b) Primary or secondary education | Actual | HADS-A | | Non-significant | HADS-D | Non-significant | |  |
|  |  |  |  |  |  | |  |  |  | |  |
|  |  | Household income decline | Yes / No | Actual |  | | Household income decline have higher risk for anxiety (OR = 1.49, p < .001). |  | Household income decline have higher risk for depression (OR = 1.63, p < .001). | |  |
|  |  |  |  |  |  | |  |  |  | |  |
| #252 | 8 | Anticipated income loss | Binary response (Yes=1% or more / No=0%) to the question: “How much are you expecting your income to reduce because of COVID-19?” | Perceived | GAD-2 | | Non-significant | PHQ-2 | Non-significant | |  |
|  |  |  |  |  |  | |  |  |  | |  |
|  |  | Anticipated job loss | Binary response (Yes / No) to the question: “Do you expect to lose your job or be unemployed because of COVID-19?” | Perceived |  | | Non-significant |  | Non-significant | |  |
|  |  |  |  |  |  | |  |  |  | |  |
|  |  | Anticipated health insurance loss | Binary response (Yes / No) to the question: “Do you expect to lose your health coverage because of COVID-19?” | Perceived |  | | Non-significant |  | Non-significant | |  |
|  |  |  |  |  |  | |  |  |  | |  |
|  |  | Cut meals | Binary response (Yes / No) to the question: “Since COVID-19 crisis began, have you had to cut the size of your meals or skip meals because there was not enough money for food? | Actual |  | | Non-significant |  | Non-significant | |  |

**Table 5 (Continued)**

*The Main Outcomes of the Studies Included in the Systematic Review*

| Study ID | Quality Assessment | SEC  Variable | Measurement  Method | Actual / Perceived | | Anxiety    Inventory Outcomes | | Depression    Inventory Outcomes | | Note | |
| --- | --- | --- | --- | --- | --- | --- | --- | --- | --- | --- | --- |
|  |  |  |  |  |  | |  |  |  | |  |
| #247 | 8 | Education | Categorized as a) Primary; b) Secondary; and c) Tertiary | Actual | EQ-5D-5L | | Non-significant | EQ-5D-5L | Non-significant | |  |
|  |  |  |  |  |  | |  |  |  | |  |
| #246 | 8 | Economic status | Categorized as a) Poor; b) Moderate; and c) Good | Perceived | DASS Anxiety | | People with poor economic status have higher risk for anxiety (OR = 0.16, p < .05). | DASS Depression | People with poor economic status have higher risk for depression (OR = 2.25, p < .05). | |  |
|  |  |  |  |  |  | |  |  |  | |  |
|  |  | Job loss | Categorized as a) Did not lose job / not working; and b) Lost job during pandemic | Actual |  | | People who experienced job loss have higher risk for anxiety (OR = 3.40, p < .01). |  | People who experienced job loss have higher risk for depression (OR = 5.91, p < .001). | |  |
|  |  |  |  |  |  | |  |  |  | |  |
| #579 | 8 | Education  Household income  Household saving | Categorized as a) Less than high school graduate, b) High  school graduate or general education diploma equivalent, c) Some college, and d) College graduate or  higher  Categorized as a) $0 to $19 999,  b) $20 000 to $44 999, c) $45 000 to $74 999, and d) $75 000 or more.  Categorized as a) ≤ 4999 or b) ≥5000 | Actual  Actual  Actual | NA | | NA  NA  NA | PHQ-9 | Non-significant  Household income under $19999 was associated with greater depression (OR = 2.37, p = .007)  Household savings at $4999 or under was associated with greater depression (OR = 1.52, p = .04) | |  |
| #572 | 8 | Being out of money | 1-item question to assess the probability of running out of money: "The coronavirus may cause economic challenges for some people regardless of whether they are actually infected. What is the percent chance you will run  out of money because of the coronavirus in the next three months?" | Perceived | NA | | NA | PHQ-4 | High expected probability of running out of money is positively associated with the PHQ-4 mental health score. | |  |

**Table 5 (Continued)**

*The Main Outcomes of the Studies Included in the Systematic Review*

| Study ID | Quality Assessment | SEC  Variable | Measurement  Method | Actual / Perceived | | Anxiety    Inventory Outcomes | | Depression    Inventory Outcomes | | Note | |
| --- | --- | --- | --- | --- | --- | --- | --- | --- | --- | --- | --- |
|  |  |  |  |  |  | |  |  |  | |  |
| #556 | 8 | Education  Employment  Condition  COVID-related economic problem | Categorized as a) Primary; b) Secondary; and c) Higher education  Categorized as a) no change, b) worsened, and c) improved  3-point Likert scale as a) Disagree, b) Neither agree nor disagree, and c) Agree | Actual  Perceived  Perceived | GAD-7 | | Non-significant  There was an increase in anxiety levels among women when employment conditions worsened during lockdown (aOR= 1.42, p <.001).  In women, perceiving COVID-19 as a problem for their economy was associated with higher levels of anxiety (aOR= 1.37, p=.001 | PHQ-9 | Men with higher education were associated with higher depression (aOR= 1.92, p = .024)  In men, worsening working conditions were associated with higher levels of depression (aOR= 1.57, p <.001).  In women, perceiving COVID-19 as a problem for their economy was associated with higher levels of depression (aOR= 1.51, p <.001). | |  |
| #554 | 8 | Education  Employment status  Monthly household income | Categorized as a) Primary or below; b) Secondary; and c) Tertiary or above  Categorized as a) Employed; b) Dependent; and c) Unemployed  Categorized as a) $19,999 or below; b) $20,000-$39,999; c) $40,000-$59,999; d) $60,000-79,999; and e) $80,000 or above | Actual  Actual  Actual | NA | | NA  NA  NA | PHQ-9 | Having primary or below (aOR=1.56, p=<.05) and secondary (aOR=1.28, p=<.05) education levels was associated with higher depressive symptoms  Being unemployed was associated with higher depressive symptoms (aOR=1.80, p=<.01)  Having $19,999 or below (aOR=1.52, p=<.05), $20,000-$39,999 (aOR=1.64, p=<.01), and $40,000-$59,999 (aOR=1.66, p=<.01) was associated with higher depressive symptoms | |  |
| #553 | 8 | SES  Residence | Merging respondents' education, occupation, and family income into a composite scale of SES through a factor analysis  Categorized as a) Rural; and b) Urban | Actual  Actual | BSI-18 | | Non-significant  Non-significant | BSI-18 | Low SES was significantly associated with higher depression (B = -0.30, p = .002)  Non-significant | |  |

**Table 5 (Continued)**

*The Main Outcomes of the Studies Included in the Systematic Review*

| Study ID | Quality Assessment | SEC  Variable | Measurement  Method | Actual / Perceived | | Anxiety    Inventory Outcomes | | Depression    Inventory Outcomes | | Note | |
| --- | --- | --- | --- | --- | --- | --- | --- | --- | --- | --- | --- |
|  |  |  |  |  |  | |  |  |  | |  |
| #546 | 8 | Education  Income  Household finance  Employment status | Categorized as a) With college; and b) Without college  Categorized as a) <4 million yen; b) ≥4 and <8 million yen; and c) 8 million yen or higher  Categorized as a) Unchanged or better off; and b) Worse off  Categorized as a) Permanent Employee; b)  Part-time, temporary worker; c) Self-employed; d) Unemployed, laid off, or on leave; and e) Not in the labor force | Actual  Actual  Actual  Actual | GAD-7 | | Non-significant  Non-significant  Having a worse household financial situation compared to the previous year was associated with higher odds of anxiety symptoms  Being unemployed, laid off, or on leave was associated with higher odds of anxiety symptoms. But being a part-time or temporary worker with anxiety was of borderline statistical significance only | PHQ-9 | Non-significant  Non-significant  Having a worse household financial situation compared to the previous year was associated with higher odds of depressive symptoms  Being unemployed, laid off, part-time or temporary workers, or on leave was associated with higher odds of depressive symptoms | |  |
|  |  |  |  |  |  | |  |  |  | |  |
| #539 | 8 | Job situation  Future economic consequences | Categorised as a) Working; b) Working from home; c) Lockdown; d) Temporarily laid off; e) Laid off; f) Not working; g) Retired; and h) Other  Categorised as a) Not at all; b) Somewhat; c) Considerably; and d) A lot | Actual  Perceived | NA | | NA  NA | PHQ-9 | Working from home (OR = 4.40, p = .030), being temporarily laid off (OR = 5.21, p = .017), being permanently laid off (OR = 6.08, p = .034) and not working (OR = 10.16, p = .001) were all associated with increased depression.  Those who worried a lot about the future impact of COVID on household economy showed more depression (OR = 2.94, p = .029) | |  |
|  |  |  |  |  |  | |  |  |  | |  |

**Table 5 (Continued)**

*The Main Outcomes of the Studies Included in the Systematic Review*

| Study ID | Quality Assessment | SEC  Variable | Measurement  Method | Actual / Perceived | | Anxiety    Inventory Outcomes | | Depression    Inventory Outcomes | | Note | |
| --- | --- | --- | --- | --- | --- | --- | --- | --- | --- | --- | --- |
|  |  |  |  |  |  | |  |  |  | |  |
| #532 | 8 | Household food security status  Employment status  Financial impact | Six-item of HFSSM  A binary response (Yes/No) to assess whether the respondent was employed in the week prior to the interview  Categorized as a) Major; b) Moderate; c) Minor; d) None; and e) Uncertain ("too soon to tell") to assess participants' ability to meet financial obligations or essential needs (e.g., mortgage payments, utilities, groceries) in the previous week | Perceived  Actual  Perceived | GAD-7 | | Severely insecure was associated with higher odds of moderate or severe anxiety symptoms (aOR = 7.57, p < .001); Moderately insecure was associated with higher odds of moderate or severe anxiety (aOR = 2.79, p < .001); but marginally insecure was non-significant.  Non-significant  Major impact was associated with higher odds of moderate or severe anxiety symptoms (aOR = 3.82, p < .001); moderate impact was associated with higher odds of moderate or severe anxiety symptoms (aOR = 2.56, p < .001); uncertain group was associated with higher odds of moderate or severe anxiety symptoms (aOR = 2.12, p < .001); but minor impact was non-significant. | NA | NA  NA  NA | |  |
|  |  |  |  |  |  | |  |  |  | |  |
| #529 | 8 | SES | Based on the location of the hospital they visited | Perceived | NA | | NA | EPDS | No difference in depressive symptoms was observed between the two SES groups prior to social restrictions. In addition, lower SES reported a statistically significant decrease in depression symptoms while high SES showed no changes during restriction. | |  |
|  |  |  |  |  |  | |  |  |  | |  |

**Table 5 (Continued)**

*The Main Outcomes of the Studies Included in the Systematic Review*

| Study ID | Quality Assessment | SEC  Variable | Measurement  Method | Actual / Perceived | | Anxiety    Inventory Outcomes | | Depression    Inventory Outcomes | | Note | |
| --- | --- | --- | --- | --- | --- | --- | --- | --- | --- | --- | --- |
|  |  |  |  |  |  | |  |  |  | |  |
| #505 | 8 | Annual average household income | Categorized as a) <50,000; b) 50,000-120,000; and c) >120,000 | Actual | STAI | | Lower income was a significant predictor of high STAI-T: Being in the income group of 50,000-120,000 significantly predicted the score of STAI-T (OR=1.42, p<0.01). Being in the income group of below 50,000 significantly predicted the score of STAI-T (OR=1.70, p<0.001). Although they were not significant predictor for STAI-S. | NA | NA | |  |
| #498 | 8 | Education  Employment status  Financial stability | Categorized as a) Up to high school; b) Bachelor's degree; and c) Master's degree and above  Categorized as a) Employed; b) Unemployed; and c) student  1-item binary variable (Yes/No) to assess whether the respondent was financially stable | Actual  Actual  Perceived | GAD-7 | | Non-significant  Non-significant  Financial instability predicted higher psychological distress (OR=2.05, p =<.001) | PHQ-9 | Non-significant  Non-significant  Financial instability predicted higher psychological distress (OR=2.05, p =<.001) | |  |
| #492 | 6 | Education  Economic status  Occupation | Categorized as a) School; b) College; c) University; and d) Professional  Categorized as a) Poor; b) Lower middle; c) Upper middle; and d) Upper  Categorized as a) Student; b) Housewife/husband; c) Retired; d) Unemployed; e) Self-employed; f) Salaried; g) Professional; h) Health care professional; and i) Business/employer | Actual  Perceived  Actual | GAD-7 | | University education was associated with higher anxiety (p < .001)  Non-significant  Being employed was associated with lower anxiety (p < .001) | PHQ-9 | University education was associated with higher depression (p < .001)  Non-significant  Employed was associated with lower depression (p < .001) | |  |
|  |  |  |  |  |  | |  |  |  | |  |

**Table 5 (Continued)**

*The Main Outcomes of the Studies Included in the Systematic Review*

| Study ID | Quality Assessment | SEC  Variable | Measurement  Method | Actual / Perceived | | Anxiety    Inventory Outcomes | | Depression    Inventory Outcomes | | Note | |
| --- | --- | --- | --- | --- | --- | --- | --- | --- | --- | --- | --- |
|  |  |  |  |  |  | |  |  |  | |  |
| #491 | 8 | Healthcare access  Ability to manage rent or housing payments  Ability to manage income  Ability to manage utility bills  Ability to manage food/ food insecurity | Categorized as a) Yes; b) No; and c) Not sure to the question: “If you think you had coronavirus, do you think you would have access to the appropriate healthcare resources for treatment?”  A binary response (yes/no) to the question of “Has your ability to manage the following become more difficult as a result of the pandemic?”  A binary response (yes/no) to the question “Has your ability to manage the following become more difficult as a result of the pandemic?”  A binary response (yes/no) to the question “Has your ability to manage the following become more difficult as a result of the pandemic?”  A binary response (yes/no) to the question “Has your ability to manage the following become more difficult as a result of the pandemic?” | Perceived  Perceived  Perceived  Perceived  Perceived | K10+ /  GAD-2 | | Non-significant  Non-significant  Non-significant  Non-significant  Food insecurity significantly predicted higher distress (p < .05, β = 8.71). | NA | NA  NA  NA  NA  NA | |  |

**Table 5 (Continued)**

*The Main Outcomes of the Studies Included in the Systematic Review*

| Study ID | Quality Assessment | SEC  Variable | Measurement  Method | Actual / Perceived | | Anxiety    Inventory Outcomes | | Depression    Inventory Outcomes | | Note | |
| --- | --- | --- | --- | --- | --- | --- | --- | --- | --- | --- | --- |
|  |  |  |  |  |  | |  |  |  | |  |
| #478 | 7 | Economic status | Categorized as a) High/very high; and b) Low/middle | Perceived | NA | | NA | BDI | Non-significant | |  |
| #460 | 8 | Occupation | Categorized as a) White collar; b) Labourer; c) Unemployed | Actual | GAD-7 | | Non-significant | PHQ-9 | Non-significant | |  |
| #459 | 7 | Education  Changes in income during Covid  Economic status | Categorized as a) Diploma and below, b) Undergraduate, c) Postgraduate  Categorized as a) Without job, b) Decreased, c) Without change, d) Increased  Unclear | Actual  Actual  Perceived | DASS-21 | | Anxiety score was significantly higher among less-educated participant (B =. -.13)  Anxiety score was significantly higher among those with lower economic status (B=. -.090)  Perceived economic status had a negative and significant association anxiety (B=-.090) | DASS-21 | Non-significant  People who lost some or all of their income during the COVID-19 outbreak experienced a higher level of depression (p < 0.001)  Perceived economic status had a negative and significant association depression (B= -.093) | |  |
| #451 | 6 | Education  Financial resources | Categorized as a) High school diploma; b) Bachelor's or associate degree; and c) Postgraduate degree to assess highest level of education  Categorized as a) Enough; b) More than enough; and c) Not enough to assess financial resources | Actual  Perceived | Four-item of short-form version 1.0 of PROMIS | | Non-significant  Participants from not enough category showed significant higher anxiety score (B = 5.64) compared to more than enough category; but participants from enough category did not differ significantly compared to more than enough | Four-item of short-form version 1.0 of PROMIS | Non-significant  Participants from not enough category showed significant higher depression score (B = 7.74) compared to more than enough category; Participants from enough category also showed significantly higher depression score (B=2.47) compared to more than enough category | |  |

**Table 5 (Continued)**

*The Main Outcomes of the Studies Included in the Systematic Review*

| Study ID | Quality Assessment | SEC  Variable | Measurement  Method | Actual / Perceived | | Anxiety    Inventory Outcomes | | Depression    Inventory Outcomes | | Note | |
| --- | --- | --- | --- | --- | --- | --- | --- | --- | --- | --- | --- |
|  |  |  |  |  |  | |  |  |  | |  |
| #446 | 8 | Education  Personal income  Family income  Householder  Unemployment experience | Categorized as a) Jr high school; b) High school; c) Associate degree or Diploma; d) Bachelor; and e) Master or Doctorate  Categorized as a) Low; b) Middle; and c) High  Categorized as a) Low; b) Middle; and c) High  Yes / No  Unclear | Actual  Perceived  Perceived  Actual  Actual | NA | | NA  NA  NA  NA  NA | CES-D | This variable is used in T2 only  This variable is used in T2 only  This variable is used in T2 only  This variable is used in T2 only  This variable is used in T2 only | | Please see Table 4 for T2 |
| #445 | 8 | Education  Family income | Years of education  Categorized as a) Low; b) Medium; and c) High | Actual  Perceived | GAD-7 | | Non-significant  Non-significant | PHQ-9 | Non-significant  Lower family income was associated with higher depression | |  |
| #443 | 8 | Education  Occupation  Average monthly household income (MYR) | Categorized as a) Secondary school; and b) Tertiary  Categorized as a) Professional and managerial; b) General worker; c) Student; and d) Housewife / Retiree / Unemployed / Others  Categorized as a)< 2000; b) 2001 - 4000; c) 4001- 8000; and d) > 8000 | Actual  Actual  Actual | STAI-6 | | Non-significant  Non-significant  Non-significant | NA | NA  NA  NA | |  |
|  |  |  |  |  |  | |  |  |  | |  |

**Table 5 (Continued)**

*The Main Outcomes of the Studies Included in the Systematic Review*

| Study ID | Quality Assessment | SEC  Variable | Measurement  Method | Actual / Perceived | | Anxiety    Inventory Outcomes | | Depression    Inventory Outcomes | | Note | |
| --- | --- | --- | --- | --- | --- | --- | --- | --- | --- | --- | --- |
|  |  |  |  |  |  | |  |  |  | |  |
| #442 | 8 | Education  Household income  Employment Change | Categorized as a) High school and below; b) Above high school and below bachelor; and c) Bachelor and above  Categorized as a) Less than $24,999;  b) $25,000 to $49,999;  c) $50,000 to $74,999;  d) $75,000 to $99,999; and  e) $100,000 or more  Categorized as a) Work without change; b) No job; c) Lost job or it was closed temporarily; d) Work home before pandemic; and e) Work from home or be paid for time off | Actual  Actual  Actual | GAD-7 | | Non-significant  Non-significant  Non-significant | PHQ2, PHQ-8 | Non-significant  People with higher household income have lower risk for depression (OR = 0.87, p < .01).  Non-significant | |  |
| #440 | 8 | Education  Employment Status | Categorized as a) Degree holder; b) High school; and c) Secondary or below  Categorized as a) Unemployed; b) Housewife; c) Student; d) Employed; and e) Retired | Actual  Actual | DASS-21 | | Higher education was associated with higher anxiety  Employed were associated with lower anxiety | DASS-21 | Higher education was associated with higher depression  Employed were associated with lower depression | |  |
| #439 | 7 | Education  Household income (annual)  Financial difficulties | Unclear  Unclear  6-item inventory which includes question such as "I lost income due to Covid-19" | Actual  Actual  Perceived | CWS | | Non-significant  Non-significant  More financial difficulties were associated with higher anxiety (Beta = .255, p < .01) | CESD-10 | Higher education was associated with lower depression (Beta = -.261, p < .01)  Non-significant  Non-significant | |  |

**Table 5 (Continued)**

*The Main Outcomes of the Studies Included in the Systematic Review*

| Study ID | Quality Assessment | SEC  Variable | Measurement  Method | Actual / Perceived | | Anxiety    Inventory Outcomes | | Depression    Inventory Outcomes | | Note | |
| --- | --- | --- | --- | --- | --- | --- | --- | --- | --- | --- | --- |
|  |  |  |  |  |  | |  |  |  | |  |
| #759  #756 | 8  8 | Income  Occupation  Job loss  Income loss  Education  Financial difficulty | Unclear  Unclear  Unclear  Unclear  Categorised as a) High school graduate or below; and b) Some college or above  5-point Likert scale (1=Not difficult at all and 5=Completely difficult) to the question: “How difficult is it for (you/your family) to meet monthly payments on (your/your family’s) bills?” | Actual  Actual  Actual  Actual  Actual  Perceived | GAD-7  NA | | Family income of less than 5 thousand MN (OR = .23) or between 5001 and 10 thousand MN (OR = .29) were associated with a higher risk of severe anxiety levels.  Being unemployed (OR = 2.62) were related to a higher probability of suffering severe anxiety.  The loss of the participant's job or that of a family member due to the pandemic (OR = .31) was associated with a higher risk for presenting severe anxiety levels.  Non-significant  NA  NA | CES-D20  CIDI-SF | Non-significant  Those who were unemployed (OR = 2.43) were at a higher risk to suffer from severe depressive symptoms.  Those who lost their job or a family member because of the COVID-19 pandemic (OR = .35) were associated with a higher risk to suffer severe depressive symptoms.  Non-significant  Middle-aged adults (Age 46-64 years) who reported higher education levels (some college or above) had a higher risk of having depression (depression; OR = 2.98, p = 0.03). Non-significant among Older Adults (Age ≥ 65 years).  Financial difficulty was positively associated with depression among middle-aged adults (aged 46-64 years; OR = 1.78, p < 0.001). Non-significant among older adults (aged ≥ 65 years). | |  |

**Table 5 (Continued)**

*The Main Outcomes of the Studies Included in the Systematic Review*

| Study ID | Quality Assessment | SEC  Variable | Measurement  Method | Actual / Perceived | | Anxiety    Inventory Outcomes | | Depression    Inventory Outcomes | | Note | |
| --- | --- | --- | --- | --- | --- | --- | --- | --- | --- | --- | --- |
|  |  |  |  |  |  | |  |  |  | |  |
| #744  #738  #723 | 8  8  8 | Education  Technical title  City level  Financial problems  Property damage (CNY)  Education  Employment status  Average household income | Categorized as a) Secondary school or below; b) College; c) Undergraduate; and d) Post-graduate or higher  Categorized as a) None; b) Junior; c) Intermediate; and d) Senior  Categorized as a) First-tier; b) Second-tier; and c) Third-tier  EHQ  Categorized as a) 0; b) < 5000; c) 5000-9999; d) 10000-29999; and e) >30000 to the question: "How much money have you lost during the COVID-19 outbreak until now?"  Categorized as a) Junior middle school and below; b) Senior middle school; and c) University and above  Categorized as a) Student; b) Working; and c) Not working  Categorized as a) <1500; b) 1500-2999; c) 3000-5999; d) 6000-8999; and e) >=9000 | Actual  Actual  Actual  Perceived  Actual  Actual  Actual  Actual | GAD-7  NA  SAS | | Non-significant  Non-significant  There was significantly higher anxiety between third-tier and first-tier cities. Non-significant between second-tier and first-tier cities.  NA  For affected group, higher losses were associated with higher anxiety (B = 1.035, p = .003). Similarly for unaffected group as well (B = 0.634, p = .001).  Non-significant  Non-significant  For affected group, lower household income was associated with higher anxiety (anxiety; B = -.0975, p = .028). Non-significant for unaffected group. | PHQ-9  PHQ-9  SDS | Non-significant  Non-significant  There was significantly higher depression between third-tier and first-tier cities. Non-significant between second-tier and first-tier cities.  Reporting financial problems increased the risk of having a clinically important worsening of depressive symptoms (aOR = 2.41, p = .015)  For affected group, higher losses were associated with higher depression (depression; B = 1.374, p = .002). Non-significant for unaffected group.  For affected group, higher education was associated with lower depression (B = -1.546, p = .015). Non-significant for unaffected group.  Non-significant  Non-significant | |  |

**Table 5 (Continued)**

*The Main Outcomes of the Studies Included in the Systematic Review*

| Study ID | Quality Assessment | SEC  Variable | Measurement  Method | Actual / Perceived | | Anxiety    Inventory Outcomes | | Depression    Inventory Outcomes | | Note | |
| --- | --- | --- | --- | --- | --- | --- | --- | --- | --- | --- | --- |
|  |  |  |  |  |  | |  |  |  | |  |
| #702  #693 | 8  8 | Employment status  Education  Family income  Employment status  Education | Categorized as a) Employed; b) Retired; and c) Unemployed  Categorized as a) Primary school and below; b) Junior middle school; c) Senior middle school; and d) University and above  Categorized as a) Low; b) Lower; c) Middle; d) Higher; and e) High  Yes / No  Categorized as a) Bachelor; b) Master; and c) PhD | Actual  Actual  Perceived  Actual  Actual | EQ-5D  GAD-7 | | Non-significant  Non-significant  Lower family income predicts lower anxiety (B = 0.231, p = .008).  Unemployment significantly predicted higher anxiety (B = -1.15, p < .001).  Having a bachelor education level significantly predicted higher anxiety (B = 1.61, p < .01). | EQ-5D  PHQ-9 | Non-significant  Non-significant  Lower family income predicts lower depression (B = 0.231, p = .008).  Unemployment significantly predicted higher depression (B = -2.44, p < .001).  Having a bachelor education level significantly predicted higher depression (B = 3.02, p < .001). | |  |

**Table 5 (Continued)**

*The Main Outcomes of the Studies Included in the Systematic Review*

| Study ID | Quality Assessment | SEC  Variable | Measurement  Method | Actual / Perceived | | Anxiety    Inventory Outcomes | | Depression    Inventory Outcomes | | Note | |
| --- | --- | --- | --- | --- | --- | --- | --- | --- | --- | --- | --- |
|  |  |  |  |  |  | |  |  |  | |  |
| #691 | 8 | Education  Employment status  Occupation  Income  Worry about unemployment | Categorized as a) High/vocational school or below; b) Three-year college degree; c) Bachelor’s degree; and d) Postgraduate degree  Categorized as a) Worked at home; b) Worked at office; and c) Worked alternately at home or office  Categorized as a) Ordinary staff; b) Junior manager; c) Middle manager; and d) Senior manager  Categorized as a) Decreased a lot; b) Decreased; c) No change; d) Increased; and e) Increased a lot  Categorized as a) Not at all; b) A little bit; c) Moderate; and d) Very much | Actual  Actual  Actual  Perceived  Perceived | GAD-7 | | Subjects with a high education level have higher risk for anxiety (anxiety; OR = 1.57, p < .05).  Non-significant  Non-significant  Non-significant  Subjects who worry about unemployment have higher risk for anxiety (OR = 2.12, p < .001). | CES-D | Non-significant  Non-significant  Non-significant  Non-significant  Subjects who worry about unemployment have higher risk for depression (OR = 1.93, p < .001) | |  |
| #687  #685 | 8  8 | Economic Loss  Job insecurity | Binary response (Yes/No)  5-point Likert scale (1=very secure  and 5=not at all secure) to the question: “How secure do you feel about your job or career prospects in your current workplace due to the COVID-19 outbreak?” | Perceived  Perceived | HAM-A  NA | | Those who have experienced economic losses showed higher anxiety (anxiety; F(1, 146) = 6.3, p = 0.01)  NA | BDI  SMDA | Those who have experienced economic losses showed higher depression (depression; F(1, 146) = 4.2, p = 0.04).  The perceived job insecurity was positively associated with depressive symptom (depression; B = 0.58, p < .001). | |  |
|  |  |  |  |  |  | |  |  |  | |  |

**Table 5 (Continued)**

*The Main Outcomes of the Studies Included in the Systematic Review*

| Study ID | Quality Assessment | SEC  Variable | Measurement  Method | Actual / Perceived | | Anxiety    Inventory Outcomes | | Depression    Inventory Outcomes | | Note | |
| --- | --- | --- | --- | --- | --- | --- | --- | --- | --- | --- | --- |
|  |  |  |  |  |  | |  |  |  | |  |
| #684  #677 | 8  7 | Education  Worry about finances  Education  Income  Worry about own economy | Categorized as a) Less than high school; b) High school only; c) Trade certificate or diploma; and d) Bachelor's degree or higher  Categorized as a) Not at all; b) A little; c) Moderately; d) Very; and e) Extremely  Categorized as a) Presecondary; b) Secondary; c) Postsecondary; and d) Graduate studies  Categorized as a) Lower middle-income; b) Middle-income; c) Upper middle-income; and d) High-income  5-point Likert scale (1=Not at all worried and 5=Extremely worried) to 1-item variable assessing whether the respondent was worried about their own financial circumstances due to COVID-19 | Actual  Perceived  Actual  Unclear  Perceived | DASS-21  GAD-7 | | Having trade certificate or diploma (β = -0.05, p = .03) and Bachelor's degree or higher (β = -0.11, p = .00) predicted lower anxiety.  Worry about finances predicted higher anxiety (β = .07, p = .00)  Non-significant  Non-significant  Worry about own economy was associated with higher anxiety (β = .165, p = < .001). | DASS-21  PHQ-9 | Having trade certificate or diploma (β = -0.04, p = .08) and Bachelor's degree or higher (β = -0.07, p = .00) predicted lower depression.  Worry about finances predicted higher depression (β = .09, p = .00)  Non-significant  Non-significant  Worry about own economy was associated with higher depression (β = .163, p = < .001). | |  |
|  |  |  |  |  |  | |  |  |  | |  |
| #675  #660 | 7  8 | Whether students are offering private tuition services to earn extra income  Education  Stress in finance | Binary response (Yes/No)  Categorized as a) Bachelor's; b) Master's; and c) Doctoral degree  Binary response (Yes/No) | Actual  Actual  Perceived | GAD-7  GAD-7 | | Students who provided private tuition showed increased anxiety (B = 0.322, p = .01).  Non-significant  Non-significant | PHQ-9  PHQ-9 | Students who provided private tuition showed increased depression (B = 0.181, p = .046).  Non-significant  Financial stress was associated with higher depression (OR = 1.86, p = .013). | |  |
|  |  |  |  |  |  | |  |  |  | |  |
|  |  |  |  |  |  | |  |  |  | |  |

**Table 5 (Continued)**

*The Main Outcomes of the Studies Included in the Systematic Review*

| Study ID | Quality Assessment | SEC  Variable | Measurement  Method | Actual / Perceived | | Anxiety    Inventory Outcomes | | Depression    Inventory Outcomes | | Note | |
| --- | --- | --- | --- | --- | --- | --- | --- | --- | --- | --- | --- |
|  |  |  |  |  |  | |  |  |  | |  |
| #656  #641 | 8  7 | Education  Employment status  Size of residences  COVID-19 economic worries  Education  Worried about loss of income due to workplace closure  Worried about loss of part of income due to reduced working hours  Worried about getting food and other essentials  Worried about getting access to routine health care or getting medications | Categorized as a) Elementary; and b) Other than elementary  Categorized as a) Employed; and b) Not Employed  Categorized as a) 0 - 4999; b) 5000 - 19999; c) 20000 - 99999; and d) 100000 and more  Categorized as a) Definitely yes; b) Rather yes; c) Rather no; d) Definitely no; and e) Not applicable  Categorized as a) Below high school or secondary school; b) High school or secondary school; c) Some college; d) Bachelor degree; and e) Post-graduate degree  Binary response (Yes/No)  Binary response (Yes/No)  Binary response (Yes/No)  Binary response (Yes/No) | Actual  Actual  Actual  Perceived  Actual  Perceived  Perceived  Perceived  Perceived | M.I.N.I  HADS | | Non-significant  Unemployed was associated with greater risk of anxiety (aOR = 2.44, p < .001).  Non-significant  Higher COVID-19 economic worries was associated with higher risk of anxiety (aOR = 1.43, p < .001).  Non-significant  Subjects that worry about loss of income due to workplace closure have increased risk for anxiety (p < .001, OR = 3.24).  Subjects that worry about loss of part of income due to reduced working hours have increased risk for anxiety (p < .001, OR = 3.09).  Non-significant  Non-significant | M.I.N.I  HADS | Non-significant  Unemployed was associated with greater risk of depression (aOR = 2.17, p < .001).  Non-significant  Higher COVID-19 economic worries was associated with higher risk of depression (aOR = 1.44, p < .001).  Non-significant  Subjects that worry about loss of income due to workplace closure have increased risk for depression (p < .001, OR = 2.41).  Subjects that worry about loss of part of income due to reduced working hours have increased risk for depression (p < .001, OR = 2.47).  Non-significant  Non-significant | |  |

**Table 5 (Continued)**

*The Main Outcomes of the Studies Included in the Systematic Review*

| Study ID | Quality Assessment | SEC  Variable | Measurement  Method | Actual / Perceived | | Anxiety    Inventory Outcomes | | Depression    Inventory Outcomes | | Note | |
| --- | --- | --- | --- | --- | --- | --- | --- | --- | --- | --- | --- |
|  |  |  |  |  |  | |  |  |  | |  |
| #637  #636  #630 | 8  7  8 | Income  SEP Index  Income loss  Job loss  Money situation  Food security | Categorized as a) <500; b) 501-1000; c) 1001-1500; d) 1501-2000; and e) >2000  Computed using 5 indicators of SEP: a) Household income; b) Employment status; c) Education; d) Household tenure; e) Household overcrowding  Binary response (Yes/No) to the question: "Have you lost income?"  Binary response (Yes/No)  Categorized as a) Comfortable with extra; b) Enough, but no extra; c) Have to cut back; and d) Cannot make ends meet  Categorized as a) Never true; b) Sometimes true; and c) Often true to the question: "Ran out didn't have money to get more foods" | Actual  Actual  Perceived  Actual  Perceived  Perceived | BAI  NA  GAD-2 | | Higher income was associated with lower anxiety scores (β = -.12, p < .01)  NA  Non-significant  Loss of employment was positively associated with higher anxiety symptoms (B = 0.346, p < .001).  Non-significant  Non-significant | NA  PHQ-9  PHQ-2 | NA  Low-SEP was positively associated with moderate (OR = 1.97; p < .001) and severe (OR = 5.22; p < .001) depressive symptoms.  Non-significant  Loss of employment was positively associated with higher depressive symptoms (B = 0.456, p < .001).  Non-significant  Non-significant | |  |
|  |  |  |  |  |  | |  |  |  | |  |

**Table 5 (Continued)**

*The Main Outcomes of the Studies Included in the Systematic Review*

| Study ID | Quality Assessment | SEC  Variable | Measurement  Method | Actual / Perceived | | Anxiety    Inventory Outcomes | | Depression    Inventory Outcomes | | Note | |
| --- | --- | --- | --- | --- | --- | --- | --- | --- | --- | --- | --- |
|  |  |  |  |  |  | |  |  |  | |  |
| #629  #622 | 8  8 | Financial loss  Income loss | Binary response (Yes/No)  Categorized as a) Complete loss; b) Reduced; and c) Unaffected | Actual  Perceived | GAD-7  GAD-2 | | Financial loss was significantly associated with higher risk of anxiety across all countries (p < .001).  Participants with complete loss of income were significantly higher in anxiety aOR = 1.73, p = .006) compared to unaffected. No significant difference was observed for those with reduced loss of income. | PHQ-9  PHQ-2 | Financial loss was significantly associated with higher risk of depression across all countries (p < .001).  Participants with complete loss of income (aOR = 2.37, p < .001) and reduced income (aOR = 1.48, p = .038) were significantly higher in depression compared to unaffected. | |  |
| #616 | 8 | Employment status  Annual income | Binary response (Yes/No)  Categorized as a) <£15,000; b) £15,000-<£25,000; c) £25,000-<£40,000; d) £40,000-<£60,000; and e) ≥£60,000 | Actual  Actual | BAI | | Non-significant  Using annual income of <£15,000 as reference group,, people with annual income of £25,000-<£40,000 have higher risk for moderate-to-severe anxiety symptoms (p < .05, OR = .54); people with annual income of £40,000-<£60,000 have higher risk for moderate-to-severe anxiety symptoms (p < .01, OR = .39); people with annual income of ≥£60,000 have higher risk for moderate-to-severe anxiety symptoms (p < .01, OR = .38). | BDI | Compared to unemployed people, employed people have higher risk for moderate-to-severe depressive symptoms (p < .05, OR = .55).  Compared to people with an annual income of <£15,000, people with annual income of £40,000-<£60,000 have higher risk for moderate-to-severe depressive symptoms (p < .05, OR = .51). | |  |

**Table 5 (Continued)**

*The Main Outcomes of the Studies Included in the Systematic Review*

| Study ID | Quality Assessment | SEC  Variable | Measurement  Method | Actual / Perceived | | Anxiety    Inventory Outcomes | | Depression    Inventory Outcomes | | Note | |
| --- | --- | --- | --- | --- | --- | --- | --- | --- | --- | --- | --- |
|  |  |  |  |  |  | |  |  |  | |  |
| #602  #585  #584 | 8  7  8 | Education  Financial uncertainty  Inadequate food supply  Education  Financial problem  Economic hardship | Categorized as a) Studying at university; and b) College  Unclear  Unclear  Categorized as a) Primary school; b) Secondary school; c) University undergraduate degree; and d) University postgraduate degree  Unclear  Binary response (Yes/No) to the question: " Difficulty paying for basic needs, including food, clothing" | Actual  Unclear  Unclear  Actual  Perceived  Perceived | DASS-21  HADS  NA | | Non-significant  Financial uncertainty had significant association with higher scores in DASS anxiety subscale (B = 2.15)  Inadequate food supply had significant relationship with higher scores in DASS anxiety subscale (B = 2.63).  Non-significant  Financial problem was associated with higher anxiety (aOR = 2.270, p = .007).  NA | DASS-21  HADS  CESD-7 | Non-significant  Financial uncertainty had significant association with higher scores in DASS depression subscale (B = 3.37).  Inadequate food supply had significant relationship with higher scores in DASS depression subscale (B = 2.95)  Non-significant  Financial problem was associated with higher depression (aOR = 2.275, p = .006).  Economic hardship significantly predict higher depression (B = 1.04, p < .001). | |  |

**Table 5 (Continued)**

*The Main Outcomes of the Studies Included in the Systematic Review*

| Study ID | Quality Assessment | SEC  Variable | Measurement  Method | Actual / Perceived | | Anxiety    Inventory Outcomes | | Depression    Inventory Outcomes | | Note | |
| --- | --- | --- | --- | --- | --- | --- | --- | --- | --- | --- | --- |
|  |  |  |  |  |  | |  |  |  | |  |
| #580 | 7 | Job insecurity  Financial concern  Income  Education | 5-point Likert-scale (1=Not at all and 5=Very worried) to the question:  “If employed, how worried are you about the effect of coronavirus, COVID-19, on your employment?”  6-point Likert-scale (1=Strongly disagree and 6= Strongly agree) to 4-items inventory, including question such as: “My or my family’s financial situation will get much worse over the next 12 months”  Unclear  Unclear | Perceived  Perceived  Actual  Actual | GAD-7 | | Non-significant  Greater financial concern were associated with greater anxiety symptoms (β = .12, p < .001)  Non-significant  Non-significant | PHQ-8 | Greater job insecurity was associated with greater depressive symptoms (β = .07, p < .05)  Non-significant  Non-significant  Non-significant | |  |
|  |  |  |  |  |  | |  |  |  | |  |
| #1706 | 8 | Employment status | Categorized as a) Student; b) Working; and c) Unemployed | Actual | DASS-21 | | Non-significant | DASS-21 | Non-significant | |  |
|  |  |  |  |  |  | |  |  |  | |  |
|  |  | Socioeconomic concerns | 5-point Likert scale (0=Not at all and 4=Extremely) | Perceived | DASS-21 | | Non-significant | DASS-21 | Non-significant | |  |
|  |  |  |  |  |  | |  |  |  | |  |
| #1695 | 8 | Financial difficulties | Categorized as a) None; b) Few; c) Some; and d) A lot | Perceived | NA | | NA | PHQ-9 | Financial difficulties increase probability of depression (aOR = 2.05, p = <.001) | |  |
|  |  |  |  |  |  | |  |  |  | |  |
| #1473 | 7 | Education | Categorized as a) Technician; b) Bachelor’s; c) Masters; and d) Doctorate | Actual | GAD-7 | | Higher education predicted lower anxiety (β = -.75, p = .04) | K6 | Non-significant | |  |
|  |  |  |  |  |  | |  |  |  | |  |

**Table 5 (Continued)**

*The Main Outcomes of the Studies Included in the Systematic Review*

| Study ID | Quality Assessment | SEC  Variable | Measurement  Method | Actual / Perceived | | Anxiety    Inventory Outcomes | | Depression    Inventory Outcomes | | Note | |
| --- | --- | --- | --- | --- | --- | --- | --- | --- | --- | --- | --- |
|  |  |  |  |  |  | |  |  |  | |  |
| #1454 | 8 | Living location | Categorized as a) Rural; b) Town; and c) City | Actual | GAD-7 | | Non-significant | PHQ-9 | Non-significant | |  |
|  |  |  |  |  |  | |  |  |  | |  |
|  |  | Income | Categorized as a) £0 - £15,490 pa; b) £15,490 - £25,340 pa; c) £25,340 - £38,740 pa; d) £38,740 - £57,930 pa; and e) £57,930+ pa | Actual |  | | Income of £0 – £15,490 pa (OR = 2.44, p = <.001), £15,490 - £25,340 pa (OR = 1.67, p = <.01) and £25,340 - £38,740 (OR = 1.69, p = <.01) predicted higher anxiety |  | Income of £0 – £15,490 pa (OR = 2.44, p = <.001), £15,490 - £25,340 pa (OR = 1.67, p = <.01) and £25,340 - £38,740 (OR = 1.69, p = <.01) predicted higher depression | |  |
|  |  |  |  |  |  | |  |  |  | |  |
|  |  | Income loss | Categorized as a) Not lost; and b) Lost | Actual |  | | Income loss predicted anxiety (OR = 1.25, p = <.001) |  | Income loss predicted depression (OR = 1.25, p = <.001) | |  |
|  |  |  |  |  |  | |  |  |  | |  |
| #1397 | 8 | Education | Categorized as a) Secondary; b) Higher secondary; c) Undergraduate; and d) Graduate and higher | Actual | DASS-21 | | Participants from secondary category have significantly higher anxiety (OR = 11.03, p = <.01) compared to graduate and higher category; but participant from higher secondary and undergraduate categories did not differ significantly from graduate and higher category | DASS-21 | Participants from secondary category have significantly higher depression (OR = 11.15, p = <.001) compared to graduate and higher category; but participant from higher secondary and undergraduate categories did not differ significantly from graduate and higher category | |  |
|  |  |  |  |  |  | |  |  |  | |  |
|  |  | Income (family, monthly) | Categorized as a) ≤ 27,000 BDT; and b) >27,000 BDT | Actual |  | | Participants from ≤ 27,000 BDT category have significantly higher anxiety (OR = 2.56, p <.001) compared to participants from >27,000 BDT category |  | Participants from ≤ 27,000 BDT category have significantly higher depression (OR = 2.62, p <.001) compared to participants from >27,000 BDT category | |  |
|  |  |  |  |  |  | |  |  |  | |  |

**Table 5 (Continued)**

*The Main Outcomes of the Studies Included in the Systematic Review*

| Study ID | Quality Assessment | SEC  Variable | Measurement  Method | Actual / Perceived | | Anxiety    Inventory Outcomes | | Depression    Inventory Outcomes | | Note | |
| --- | --- | --- | --- | --- | --- | --- | --- | --- | --- | --- | --- |
|  |  |  |  |  |  | |  |  |  | |  |
| #1353 | 8 | Education | Categorized as a) Senior high school or below; and b) Above senior high school | Actual | GAD-7 | | Non-significant | PHQ-9 and 15 | Non-significant | |  |
|  |  |  |  |  |  | |  |  |  | |  |
| #1321 | 8 | Education | Categorized as a) Technical secondary school and the following; b) Undergraduates and above; c) Junior colleges; and d) Vocational colleges | Actual | SAS | | Non-significant | SDS | Non-significant | |  |
|  |  |  |  |  |  | |  |  |  | |  |
| #1293 | 7 | Education | Categorized as a) Nursing diploma; and b) Bachelor of nursing | Actual | DASS-21 | | Non-significant | DASS-21 | Non-significant | |  |
|  |  |  |  |  |  | |  |  |  | |  |
|  |  | Employment status | Categorized as a) Temporary; and b) Permanent | Actual |  | | Non-significant |  | Non-significant | |  |
|  |  | Financial difficulties | Binary response (Yes/No) | Actual |  | | Non-significant |  | Non-significant | |  |
|  |  |  |  |  |  | |  |  |  | |  |
|  |  |  |  |  |  | |  |  |  | |  |
| #1239 | 8 | Education | Categorized as a) Lower secondary/ upper secondary; b) College; c) University; and d) Postgraduate | Actual | GAD-7 | | Non-significant | PHQ-9 | Non-significant | |  |
|  |  |  |  |  |  | |  |  |  | |  |
|  |  | Residence area | Categorized as a) Village; b) Town; and c) City | Actual |  | | Non-significant |  | Non-significant | |  |
|  |  |  |  |  |  | |  |  |  | |  |
| #1213 | 8 | Education | Categorized as a) Never attended formal school; b) Primary education; and c) Secondary education | Actual | NA | | NA | PHQ-9 | Having primary education (aOR = .34, p = .05) or secondary education or above (aOR = .23, p = .009) was associated with lower depression | |  |
|  |  |  |  |  |  | |  |  |  | |  |
|  |  | Income (daily) | Categorized as a) ≤150 ETB; and b) >150 ETB | Actual |  | | NA |  | Non-significant | |  |
|  |  |  |  |  |  | |  |  |  | |  |

**Table 5 (Continued)**

*The Main Outcomes of the Studies Included in the Systematic Review*

| Study ID | Quality Assessment | SEC  Variable | Measurement  Method | Actual / Perceived | | Anxiety    Inventory Outcomes | | Depression    Inventory Outcomes | | Note | |
| --- | --- | --- | --- | --- | --- | --- | --- | --- | --- | --- | --- |
|  |  |  |  |  |  | |  |  |  | |  |
| #1205 | 7 | Financial threat | Binary response (Yes/No) to 1-tem variable to assess whether the respondents believed that the COVID-19 outbreak represented a private financial risk | Perceived | DASS-21 | | Non-significant | DASS-21 | Perceived COVID-19 as a personal financial threat was negatively associated with depression (β = -.12, p = -.06) | |  |
|  |  |  |  |  |  | |  |  |  | |  |
| #1189 | 7 | Employment status | Binary response (Yes/No) | Actual | NA | | NA | BDI-II | Patient group: non-working status was a significant predictor of an increase in depression.; Healthy group: non-significant | |  |
|  |  |  |  |  |  | |  |  |  | |  |
|  |  | Finance | Categorized as a) Enough for living; and b) Not enough money for living | Perceived |  | | NA |  | Patient group: not having enough money was a significant predictor of an increase in depression; Healthy group: non-significant | |  |
|  |  |  |  |  |  | |  |  |  | |  |
|  |  | Education | Unclear | Actual |  | | NA |  | Non-significant | |  |
|  |  | Income | Categorized as a) Increase; and b) Decrease | Actual |  | | NA |  | Patient group: non-significant; Healthy group: Decrease in income was associated with greater increase in depression | |  |
|  |  |  |  |  |  | |  |  |  | |  |
| #1166 | 8 | Family financial wellbeing | 4 items from the US Consumer Financial Protection Bureau (CFPB) Financial Well-being Scale – 5-point Likert scale (strongly agree to strongly disagree). | Perceived | Seattle Personality Questionnaire | | Non-significant direct effect of mother’s financial wellbeing and child’s psychological wellbeing (self-esteem, optimism, anxiety) Mother’s financial well-being indirectly predicted child’s psychological well-being (β = 0.35, p < .05) | NA | NA | |  |
|  |  |  |  |  |  | |  |  |  | |  |
| #1074 | 8 | Residence area | Categorized as a) Urban; and b) Rural | Actual | GAD-7 | | The prevalence of self-reported anxiety among respondents living in rural areas was less than that of respondents living in urban areas (PR = .67, p = <.05) | NA | NA | |  |
|  |  |  |  |  |  | |  |  |  | |  |

**Table 5 (Continued)**

*The Main Outcomes of the Studies Included in the Systematic Review*

| Study ID | Quality Assessment | SEC  Variable | Measurement  Method | Actual / Perceived | | Anxiety    Inventory Outcomes | | Depression    Inventory Outcomes | | Note | |
| --- | --- | --- | --- | --- | --- | --- | --- | --- | --- | --- | --- |
|  |  |  |  |  |  | |  |  |  | |  |
| #1065 | 7 | Education | Categorized as a) Primary; b) Secondary; and c) Higher | Actual | ASI-3 | | Non-significant | NA | NA | |  |
|  |  |  |  |  |  | |  |  |  | |  |
|  |  | Income (monthly) | Categorized as a) <3,000 PLN; b) 3,000 – 4,999PLN; and c) ≥ PLN | Actual |  | | Non-significant |  | NA | |  |
|  |  |  |  |  |  | |  |  |  | |  |
| #1059 | 8 | SES | Household economic resources in the last 12 months categorized as a) Absolutely insufficient; b) Scarce; c) Adequate; and d) Excellent | Perceived | GHQ-12 | | Non-significant | GHQ-12 | Non-significant | |  |
|  |  |  |  |  |  | |  |  |  | |  |
|  |  | Employment status | Categorized as a) Employed; and b) Others | Actual |  | | Being employed predicted higher anxiety symptoms (β=1.39, p=.000) |  | Being employed predicted higher depressive symptoms (β=1.39, p=.000) | |  |
|  |  | House size | Categorized as a) Small; b) Medium; and c) Large | Perceived |  | | Non-significant |  | Non-significant | |  |
|  |  |  |  |  |  | |  |  |  | |  |
|  |  | Financial loss | Categorized as a) Not at all; b) To a small extent; c) To a moderate extent; and d) A great extent | Perceived |  | | Non-significant |  | Non-significant | |  |
|  |  |  |  |  |  | |  |  |  | |  |
| #1020 | 7 | Employment status (pre-COVID-19) | Categorized as a) Not employed; b) Full-time on campus; c) Part-time on campus; d) Full-time on campus; and e) Part-time off campus | Actual | GAD-7/DASS-21 | | Non-significant | PHQ-9/DASS-21 | Non-significant | |  |
|  |  |  |  |  |  | |  |  |  | |  |

**Table 5 (Continued)**

*The Main Outcomes of the Studies Included in the Systematic Review*

| Study ID | Quality Assessment | SEC  Variable | Measurement  Method | Actual / Perceived | | Anxiety    Inventory Outcomes | | Depression    Inventory Outcomes | | Note | |
| --- | --- | --- | --- | --- | --- | --- | --- | --- | --- | --- | --- |
|  |  |  |  |  |  | |  |  |  | |  |
| #940 | 8 | Assets | High assets categorized as a) Household income above $45,000; b) Household savings above $5,000; c) Home ownership; d) College educated or more; and d) being married | Actual | NA | | NA | PHQ-9 | Low asset (OR = 3.0) was associated with higher odds of probable depression | |  |
|  |  |  |  |  |  | |  |  |  | |  |
|  |  | COVID-19 financial stressor | High COVID-19 financial stressor exposure defined by experiencing one or more of the following: a) Job loss; b) Family member’s job loss; c) Financial difficulties; and d) Difficulty paying rent | Actual |  | | NA |  | High COVID-19 financial stressor exposure (OR = 2.8) was associated with higher odds of probable depression | |  |
|  |  |  |  |  |  | |  |  |  | |  |
|  |  |  |  |  |  | |  |  |  | |  |
| #831 | 8 | Education | Categorized as a) ≤ high school; and b) college + | Actual | K6 | | Mother’s and expectant mothers with a college degree or higher had lower levels of psychological distress (β = -1.346, p = .014) | K6 | Mother’s and expectant mothers with a college degree or higher had lower levels of psychological distress (β = -1.346, p = .014) | |  |
|  |  |  |  |  |  | |  |  |  | |  |
|  |  | Income (family) | Categorized as a) <$1,333 USD; b) $1,333 – $2, 667 USD; and c) >$2,667 USD | Actual |  | | Mother’s and expectant mothers with monthly family income of $1,333 - $2,667 USD (β = -1.38, p = .01) and >$2,667 USD (β = -1.37, p = .01) had lower levels of psychological distress than their counterparts with <$1,333 USD family income |  | Mother’s and expectant mothers with monthly family income of $1,333 - $2,667 USD (β = -1.38, p = .01) and >$2,667 USD (β = -1.37, p = .01) had lower levels of psychological distress than their counterparts with <$1,333 USD family income | |  |
|  |  |  |  |  |  | |  |  |  | |  |

**Table 5 (Continued)**

*The Main Outcomes of the Studies Included in the Systematic Review*

| Study ID | Quality Assessment | SEC  Variable | Measurement  Method | Actual / Perceived | | Anxiety    Inventory Outcomes | | Depression    Inventory Outcomes | | Note | |
| --- | --- | --- | --- | --- | --- | --- | --- | --- | --- | --- | --- |
|  |  |  |  |  |  | |  |  |  | |  |
| #812 | 8 | Residence area | Categorized as a) Urban; and b) Rural | Actual | NA | | NA | CES-D-SF | Non-significant | |  |
|  |  |  |  |  |  | |  |  |  | |  |
|  |  | Education | Categorized as a) Degree level; b) Higher education; c) Secondary school; and d) Below secondary school | Actual |  | | NA |  | Non-significant | |  |
|  |  |  |  |  |  | |  |  |  | |  |
|  |  | Net financial wealth | Categorized as a) Low; b) Mid; and c) High | Perceived |  | | NA |  | Non-significant | |  |
|  |  |  |  |  |  | |  |  |  | |  |
|  |  | Employment status | Categorized as a) Employed; b) Unemployed; c) Retired; and d) Other | Actual |  | | NA |  | Being on a long-term sick leave was a significant predictor of a change in depression scores (F = 9.31, p = .001) | |  |
|  |  |  |  |  |  | |  |  |  | |  |
| #1415 | 11 | Education | Categorized as a) Less than high school; b) Some high school, no diploma; c) High school graduate or equivalent; d) Some college, no degree; e) Associate degree; f) Bachelor’s degree; g) Master’s degree; h) Professional school degree; and i) Doctorate degree | Actual | NA | | NA | PHQ-8 | Education had a positive total effect on depressive symptoms during COVID-19 (total effect =.040) – Higher education was associated with higher depression scores | | Please Table 4 for T2 |
|  |  |  |  |  |  | |  |  |  | |  |
|  |  | Income (household) | Total combined income of all family members 15 years or older who lived in the household over the past year on a 17-point scale (1=less than $5000 and 17=$200000 or above) | Actual |  | | NA |  | Income had a negative total effect on depressive symptoms (total effect = -.014) – Higher income was associated with lower depression score | |  |
|  |  |  |  |  |  | |  |  |  | |  |
| #1278 | 11 | Financial concerns | 11-point Likert scale (0-10) with higher values intended to reflect higher frequency or intensity to the question: “How concerned are you about your financial situation as a result of the pandemic?” | Perceived | GAD-7 | | Weak correlation of anxiety symptoms with financial concerns | PHQ-9 | Weak correlation of depression symptoms with financial concerns | | Please see Table 4 for T2 |
|  |  |  |  |  |  | |  |  |  | |  |

**Table 5 (Continued)**

*The Main Outcomes of the Studies Included in the Systematic Review*

| Study ID | Quality Assessment | SEC  Variable | Measurement  Method | Actual / Perceived | | Anxiety    Inventory Outcomes | | Depression    Inventory Outcomes | | Note | |
| --- | --- | --- | --- | --- | --- | --- | --- | --- | --- | --- | --- |
|  |  |  |  |  |  | |  |  |  | |  |
| #1140 | 11 | Job loss | Binary response (Yes/No) | Actual | GAD-7 | | This variable is not used in T1 | CESD-SF | This variable is not used in T1 | | Please see Table 4 for T2 |
|  |  |  |  |  |  | |  |  |  | |  |
|  |  | Education loss | Binary response (Yes/No) | Actual |  | | This variable is not used in T1 |  | This variable is not used in T1 | |  |
|  |  |  |  |  |  | |  |  |  | |  |
|  |  | Not employed/ in education (pre-pandemic) | Binary response (Yes/No) | Actual |  | | This variable is not used in T1 |  | This variable is not used in T1 | |  |
|  |  |  |  |  |  | |  |  |  | |  |
|  |  | Low SES family | Binary response (Yes/No)  – defined as ≤1 SD on SES scale aggregating annual gross income, parental education level, and parental occupation prestige from ages 15 -17 years | Actual |  | | This variable is not used in T1 |  | This variable is not used in T1 | |  |
|  |  |  |  |  |  | |  |  |  | |  |
| #933 | 8 | Education | Unclear | Actual | GAD-7 | | This variable is not used in T1 | SMFQ | This variable is not used in T1 | | Please see Table 4 for T2 |
|  |  |  |  |  |  | |  |  |  | |  |
|  |  | Income | Unclear | Actual |  | | This variable is not used in T1 |  | This variable is not used in T1 | |  |
|  |  |  |  |  |  | |  |  |  | |  |
|  |  | Recent financial problems | Unclear | Unclear |  | | This variable is not used in T1 |  | This variable is not used in T1 | |  |
|  |  |  |  |  |  | |  |  |  | |  |
| #883 | 11 | Income loss | Categorized as a) No income loss; b) Greater than 0% but less than 50% monthly income loss; and c) Greater than or equal to 50% monthly income loss | Actual | GAD-7 | | This variable is not used in T1 | PHQ-9 | This variable is not used in T1 | | Please see Table 4 for T2 |
|  |  |  |  |  |  | |  |  |  | |  |
| #2742 | 7 | Family income | Categorized as a) Affected; and b) Not affected | Actual | GAD-7 | | Non-significant | NA | NA | |  |
|  |  |  |  |  |  | |  |  |  | |  |

**Table 5 (Continued)**

*The Main Outcomes of the Studies Included in the Systematic Review*

| Study ID | Quality Assessment | SEC  Variable | Measurement  Method | Actual / Perceived | | Anxiety    Inventory Outcomes | | Depression    Inventory Outcomes | | Note | |
| --- | --- | --- | --- | --- | --- | --- | --- | --- | --- | --- | --- |
|  |  |  |  |  |  | |  |  |  | |  |
| #2685  #2627 | 8  7 | Employment status  Job security  Education  Monthly family income  Employment status | Categorized as a) Employed; b) Voluntarily not working; and c) Involuntarily not working  Binary response (Yes/No) to the question: “Expect family job loss”  Categorized as a) Primary or below; b) secondary or tertiary  Categorized as a) < 20,000; b) 20,000 – 39,999; c) 40,000 – 59,999; d) 60,000 – 79,999; and e) ≥ 80,000  Categorized as a) Employed versus unemployed; b) Retired; c) Housewife; and d) Student | Actual  Perceived  Actual  Actual  Actual | GAD-2  NA | | Higher involuntary employment was at higher risk of anxiety (OR = 1.20, p = .001)  Higher job uncertainty was at lower risk of anxiety (OR = 1.37, p = .001)  NA  NA  NA | PHQ-2  PHQ-9 | Higher involuntary employment was at a significant risk of depression (OR = 1.24, p = .001)  Higher job uncertainty was at a significantly lower risk for depression (OR = 1.24, p = .001)  Higher secondary education level was associated with lower risk of depression (B = 0.35, p < .01)  Higher tertiary education level was associated with lower depression (B = 0.30, p < .001)  NA  NA | |  |

**Table 5 (Continued)**

*The Main Outcomes of the Studies Included in the Systematic Review*

| Study ID | Quality Assessment | SEC  Variable | Measurement  Method | Actual / Perceived | | Anxiety    Inventory Outcomes | | Depression    Inventory Outcomes | | Note | |
| --- | --- | --- | --- | --- | --- | --- | --- | --- | --- | --- | --- |
|  |  |  |  |  |  | |  |  |  | |  |
| #2586  #2583 | 8  8 | Education  Occupation  Monthly family income  Income  Perceived financial stressors  Education | Categorized as a) Up to higher secondary; and b) Honours and above  Categorized as a) Businessman; b) Government or private service holders; c) Others; and d) Health care workers  Categorized as a) < 30,000; b) 30,000-70,000; and c) > 70,000  A checklist with four options a) I am not getting any salary in this lockdown situation; b) I have no source of income currently; c) My income is not enough for my family; and d) I am satisfied with my income  A checklist with six options a) I am getting no salary in this lockdown situation; b) Food supply is not enough for my family; c) Dealing with the financial problem; d) Future financial crisis; e) Price increment in daily necessary commodities; and f) Hamper my children’s study  Categorized as a) Primary or below; b) Junior secondary; c) Senior secondary; d) Matriculation; e) Undergraduate; and f) Postgraduate or above | Actual  Actual  Actual  Actual  Perceived  Actual | GAD-7  GAD-7 | | Having education below honours was associated with increased risk of anxiety (OR = 2.30, p < .01)  Working in business was associated with increased risk of anxiety (aOR = 2.40, p < .01)  Earnings of < 30,000 monthly (anxiety; aOR = 3.12, p < .01) and earnings between 30,000-70,000 were at a significant risk for anxiety (aOR = 1.78, p < .05)  Being the only earning member of the family was at a significant risk for anxiety (aOR = 1.47, p < .05)  Non-significant  Non-significant | PHQ-9  PHQ-9 | Having education below honours was associated with increased risk of depression (aOR = 1.95, p < .01)  Working in business was associated with increased risk of depression (aOR = 2.25, p < .05)  Earnings of < 30,000 monthly (depression; aOR = 2.86, p < .01) and earnings between 30,000-70,000 were at a significant risk for depression (aOR = 2.04, p < .01)  Non-significant  Non-significant  Higher level of education was associated with higher risk of depression (OR = 1.31, p = .001) | |  |

**Table 5 (Continued)**

*The Main Outcomes of the Studies Included in the Systematic Review*

| Study ID | Quality Assessment | SEC  Variable | Measurement  Method | Actual / Perceived | | Anxiety    Inventory Outcomes | | Depression    Inventory Outcomes | | Note | |
| --- | --- | --- | --- | --- | --- | --- | --- | --- | --- | --- | --- |
|  |  |  |  |  |  | |  |  |  | |  |
| #2578  #2563  #2527  #2523 | 8  7  7  8 | Income  Financial support  Income changes  Income  Residential area  Education  Monthly family income | Losing a source of income because of COVID-19  Receiving financial support from family, friends, partners, an organisation, or someone else  Categorized as a) No change; b) Decreased income; and c) Increased income  Categorized as a) Increased; and b) Decreased  Categorized as a) Urban; b) Rural  Categorized as a) Less than college; and b) College degree or higher  Categorized as a) 0 – 4,999; b) 5,000 – 11,999; and c) ≥ 12,000 | Actual  Actual  Actual  Perceived  Actual  Actual  Actual | GAD-7  NA  HADS  GAD-7 | | Non-significant  Worrying about getting financial support was at a significant risk for anxiety (aOR = 1.50, p < .05)  NA  Non-significant  Non-significant  Non-significant  Lower family income was associated with higher risk of anxiety (aOR = 1.35 – 1.48, p < .01) | EPDS  PHQ-9  HADS  PHQ-9 | Non-significant  Worrying about getting financial support was at a significant risk for depression (aOR = 1.20, p < .05)  Decreased income was associated with higher depressive symptoms (F[2, 24.76] = 317.44, p < .001)  Non-significant  Non-significant  Non-significant  Lower family income was associated with higher risk of depression (aOR = 1.35 – 1.37, p < .05) | |  |

**Table 5 (Continued)**

*The Main Outcomes of the Studies Included in the Systematic Review*

| Study ID | Quality Assessment | SEC  Variable | Measurement  Method | Actual / Perceived | | Anxiety    Inventory Outcomes | | Depression    Inventory Outcomes | | Note | |
| --- | --- | --- | --- | --- | --- | --- | --- | --- | --- | --- | --- |
|  |  |  |  |  |  | |  |  |  | |  |
| #2496  #2391  #2379 | 7  7  8 | Education  Employment status  Income  Largest open-air space at home  Education  Income  Education  Place of residence | Categorized as a) No answer; b) Primary school; c) Middle school; d) High school; e) Bachelor's degree; e) Master's degree; and f) PhD  Categorized as a) Unemployed; b) Student; c) Student worker; and d) Workers  Categorized as a) Low; b) Medium – low; c) Medium; d) Medium – high; and e) High  Categorized as a) No open-air spaces; b) Balconies and terraces; c) Condominium courtyard; and d) Private garden  Categorized as a) Undergraduate or below; and b) Postgraduate or above  Categorized as a) Unchanged; b) Increased; and c) Reduced  Categorized as a) Middle school; b) High school; c) Junior college; and d) Undergraduate and above  Categorized as a) Urban; and b) Rural | Actual  Actual  Perceived  Actual  Actual  Actual  Actual  Actual | DASS-21  GAD-7  GAD-7 | | Non-significant  Non-significant  Higher income was associated with decreased scores on the anxiety subscale (p = .002)  Non-significant  Non-significant  Income loss was associated with higher anxiety risk (OR = 1.61, p = .01)  Individuals with junior college (aOR = 2.73, p = .038) and undergraduate degrees or above (aOR = 2.95, p = .025) were associated with increased risk of anxiety symptoms  Non-significant | DASS-21; PHQ-9  PHQ-9  PHQ-9 | Non-significant  Non-significant  Lower income was associated with increased scores on the PHQ-9 scale (p < .05)  No open-air spaces was associated with increased scores on the DASS-21 Depression Subscale (p < .05)  Non-significant  Non-significant  Individuals with junior college (aOR = 2.77, p = .002) and undergraduate degrees or above (aOR = 2.98, p = .001) were associated with increased risk of depressive symptoms  Non-significant | |  |

**Table 5 (Continued)**

*The Main Outcomes of the Studies Included in the Systematic Review*

| Study ID | Quality Assessment | SEC  Variable | Measurement  Method | Actual / Perceived | | Anxiety    Inventory Outcomes | | Depression    Inventory Outcomes | | Note | |
| --- | --- | --- | --- | --- | --- | --- | --- | --- | --- | --- | --- |
|  |  |  |  |  |  | |  |  |  | |  |
| #2372  #2368  #2358 | 7  8  7 | Education  Income  Impact on livelihood  Education  Education  Employment status  Continuation of work during COVID-19  Change in economic situation | Categorized as a) Junior high school and below; b) High school or technical school; c) Junior college; d) Undergraduate; and e) Postgraduate and above  Categorized as a) Poor; and b) Not poor  Categorized as a) None; b) Some; c) Relatively large; and d) Very large  Categorized as a) Bachelor or below; and b) Master or doctorate  Categorized as a) Less than high school degree; b) High school degree; and c) More than high school degree  Categorized as a) Employed; b) Unemployed  Binary response (Yes/No)  Unclear | Actual  Perceived  Perceived  Actual  Actual  Actual  Actual  Unclear | NA  GAD-7  NA | | NA  NA  NA  Higher education level was associated with lower risk of anxiety symptoms (aOR = 0.19, p = .03)  NA  NA  NA  NA | CES-D  PHQ-9  CES-D | Individuals in high school or technical school were at higher risk for depression (OR = 1.82, p < .01)  Non-significant  Some impact on livelihood (OR = 1.39, p < .01), relatively large impact on livelihood (OR = 2.48, p < .001), and very large impact on livelihood were at higher risk for depression (OR = 2.26, p < .01)  Non-significant  Non-significant  Non-significant  Non-significant  Positive change in economic situation was associated with lower depression (β = -0.11, p < .001) | |  |

**Table 5 (Continued)**

*The Main Outcomes of the Studies Included in the Systematic Review*

| Study ID | Quality Assessment | SEC  Variable | Measurement  Method | Actual / Perceived | | Anxiety    Inventory Outcomes | | Depression    Inventory Outcomes | | Note | |
| --- | --- | --- | --- | --- | --- | --- | --- | --- | --- | --- | --- |
|  |  |  |  |  |  | |  |  |  | |  |
| #2356  #2355 | 8  8 | Place of residence  Education  Monthly family income  Job insecurity  Education | Categorized as a) Urban; and b) Rural  Categorized as a) Primary school or below; b) Middle school; and c) College degree or above  Categorized as a) <$30,000; b) $30,001 - $50,000; c) $50,001 - $80,000; and d) > $80,000  NA  Categorized as a) High school / General educational development or less; b) Trade school or apprenticeship; c) Partial college; d) Associate degree; e) Bachelor or degree; and f) Graduate degree | Actual  Actual  Actual  Perceived  Actual | SAS  BSI | | Individuals who lived in rural areas were associated with higher SAS scores (β = 0.04, p = .04)  Non-significant  Non-significant  Non-significant  Higher levels of education was associated with lower anxiety (B = -0.11, p = .002) | CES-D  BSI | Non-significant  Non-significant  Higher monthly family income was associated with higher depressive symptoms (B = 0.31, p = .04)  Higher job insecurity was associated with higher depressive symptoms (B = 0.03, p = .014)  Non-significant | |  |

**Table 5 (Continued)**

*The Main Outcomes of the Studies Included in the Systematic Review*

| Study ID | Quality Assessment | SEC  Variable | Measurement  Method | Actual / Perceived | | Anxiety    Inventory Outcomes | | Depression    Inventory Outcomes | | Note | |
| --- | --- | --- | --- | --- | --- | --- | --- | --- | --- | --- | --- |
|  |  |  |  |  |  | |  |  |  | |  |
| #2341  #2340  #2339  #2326 | 8  8  8  7 | Education  Stable income  Negative economic consequences  Worry about economy  Place of residence  Education  Monthly income for personal needs | Categorized as a) Primary school; b) High school; c) University 1-3 years; and d) University more than 3 years  Binary response (Yes/No)  6-point Likert scale (1 = Not at all and 6 = I do not know) to the question: “To what degree have your household suffered from negative economic consequences due to the pandemic?”  5-point Likert scale (1 = Not at all and 5 = Very high) to the question: “To what degree has the pandemic situation made you worry about economic consequences for yourself or someone else in your household?”  Categorized as a) Rural municipality; b) Municipality; c) Sub-metropolitan; and d) Metropolitan  Categorized as a) < 12 years; b) 12 - 16 years; and c) > 16 years  Categorized as a) <100€; b) 100 – 200€; c) 200 – 300€; and d) >300€ | Actual  Actual  Perceived  Perceived  Actual  Actual  Actual | GAD-7  GAD-7  SAS  HADS-A | | Non-significant  Not having stable income was associated with higher anxiety (B = -1.28, p = .001)  Non-significant  Higher levels of worry about economy was associated with higher anxiety (B = 0.72, p = .001)  Non-significant  Non-significant  Having monthly income of <100€ (OR = 1.50, p < 0.1) and 100 - 200€ (OR = 1.55, p < 0.1) for personal needs were at higher risk for anxiety | PHQ-9  PHQ-9  SDS  HADS-D | Higher levels of education was associated with lower depression (B = -0.07, p = .01)  Not having stable income was associated with higher depression (B = -1.78, p = .001)  Non-significant  Higher levels of worry about economy was associated with higher depression (B = 0.67, p = .001)  Non-significant  Non-significant  Having monthly income of <100€ (OR = 1.52, p < 0.1) and 200 - 300€ (OR = 1.39, p < 0.1) for personal needs were at higher risk for depression | |  |

**Table 5 (Continued)**

*The Main Outcomes of the Studies Included in the Systematic Review*

| Study ID | Quality Assessment | SEC  Variable | Measurement  Method | Actual / Perceived | | Anxiety    Inventory Outcomes | | Depression    Inventory Outcomes | | Note | |
| --- | --- | --- | --- | --- | --- | --- | --- | --- | --- | --- | --- |
|  |  |  |  |  |  | |  |  |  | |  |
| #2312  #2293  #2123  #2055  #1865 | 8  8  5  7  8 | Work  SES  Employment status  Changes in economic situation  Employment status  Education  Employment status  SES  Food difficulty during COVID  Dependent on family for a living | Binary response (Yes/No)  1-item question to assess whether they or their siblings were on free or reduced lunch. Categorized as a) Yes; b) No; and c) I don’t know  Categorized as a) Unemployed; b) Self-employed; and c) Other  Binary response (Yes/No)  Categorized as a) Both still employed; b) At least one experienced unemployment/furlough; and c) At least one experienced a reduction in their working hours  NA  Binary response (Yes/No)  Unclear  Categorized as: a) No difficulty; b) Faced difficulty  Binary response (Yes/No) | Actual  Actual  Actual  Actual  Actual  Actual  Actual  Perceived  Actual  Actual | GAD-7  GAD-7  NA  GAD-7  NA | | Non-significant  Individuals with lower SES were at higher risk of being more anxious (p = .02)  Non-significant  NA  NA  Non-significant  Non-significant  Non-significant  NA  NA | PHQ-8  PHQ-9  CESD-7  PHQ-9  GDS-15 | Non-significant  Non-significant  Non-significant  Participants who live in poverty and with changes to their economic situation were at higher risk of having more mental distress than the non-poverty participants (p < .001)  Participants who live in poverty and experienced unemployment/furlough/at least one experienced a reduction in their working hours were at higher risk of having more mental distress than the non-poverty participants (p < .001)  Non-significant  Working individuals were at lower risk of depression (B = -0.11, p = .001)  Non-significant  Non-significant  Individuals who were not dependent on family for a living were at significant risk of developing depressive symptoms (aOR = 3.35, p = .001) | |  |

**Table 5 (Continued)**

*The Main Outcomes of the Studies Included in the Systematic Review*

| Study ID | Quality Assessment | SEC  Variable | Measurement  Method | Actual / Perceived | | Anxiety    Inventory Outcomes | | Depression    Inventory Outcomes | | Note | |
| --- | --- | --- | --- | --- | --- | --- | --- | --- | --- | --- | --- |
|  |  |  |  |  |  | |  |  |  | |  |
| #3004 | 8 | SES | Categorized as a) Poor; b) Middle; and c) Good | Perceived | GAD-7 | | Non-significant | NA | NA | |  |
| #3003 | 7 | Financial restrain due to COVID-19 | Categorized as a) High level; b) Low level; and c) No restraint | Perceived | STAI-A | | Higher financial restraint due to COVID-19 increased anxiety (β=.11, p=<.001) | BDI-SF | Higher financial restraint due to COVID-19 increased depression (β=.11, p=<.001) | |  |
| #2999 | 7 | Employment changes due to COVID-19 | Categorized as a) No change in work; b) Working from home, when I was not before; and c) Lost employment. The categories have been defined in relation to the question: “What is the impact of the recent events on your work life?” | Actual | BAI | | Participants who had lost their job reported higher symptoms of anxiety (g=−0.21, p=0.008) | BDI-II (excluding the suicidality question) | Participants who had lost their job reported higher symptoms of depression (g=−0.20, p=0.003) | |  |
| #2992 | 8 | Job loss | Binary response (Yes/No) to the question: “Have you lost your primary job/income due to COVID-19?” | Actual | GAD-7; SRAS; STAI | | Job loss as a direct result of the pandemic showed greater severity of anxiety | BDI-II; PHQ-9 | Job loss as a direct result of the pandemic showed greater severity of depression | |  |
|  |  | Education | Number of years of education | Actual |  | | Non-significant |  | Non-significant | |  |
|  |  | Income | Categorized as a) ≤$10,000; b) $10,001-$25,000; c) $25,001-$50,000; d) $50,001-$75,000; e) $75,001-$100,000; f) $100,001-$150,000; g) $150,001-$200,000; and h) ≥$200,000 | Actual |  | | Non-significant |  | Non-significant | |  |
|  |  | Financial worry | Binary response (Yes/No) to the question: “Have you become worried about your ability to financially support yourself and loved ones?” | Perceived |  | | Higher financial worry was associated with greater anxiety |  | Higher financial worry was associated with greater depression | |  |

**Table 5 (Continued)**

*The Main Outcomes of the Studies Included in the Systematic Review*

| Study ID | Quality Assessment | SEC  Variable | Measurement  Method | Actual / Perceived | | Anxiety    Inventory Outcomes | | Depression    Inventory Outcomes | | Note | |
| --- | --- | --- | --- | --- | --- | --- | --- | --- | --- | --- | --- |
|  |  |  |  |  |  | |  |  |  | |  |
| #2991 | 8 | Education | Categorized as a) Senior middle school or below; b) College or vocational school; and c) Bachelor degree or above | Actual | GAD-2 | | Patients: Compared to having a college or vocational school education level, having a senior middle school or below educational level was significantly associated with higher risk for anxiety (OR=4.897, p=<.05); Compared to having a college or vocational school education level, having a bachelor degree or above educational level was significantly associated with higher risk for anxiety (OR=6.507, p=<.01). But family members: Non-significant | PHQ-2 | Patients: Compared to having a college or vocational school education level, having a senior middle school or below educational level was significantly associated with higher risk for depression (OR=4.105, p=<.05); Compared to having a college or vocational school education level, having a bachelor degree or above educational level was significantly associated with higher risk for depression (OR=4.168, p=<.05). But family members: Non-significant | |  |
|  |  | Employment status | Categorized as a) Student; b) Employed; and c) Unemployed | Actual |  | | Patients: Non-significant  Family members: Non-significant |  | Patients: Non-significant  Family members: Non-significant | |  |
|  |  |  |  |  |  | |  |  |  | |  |
| #2952 | 8 | Education | Categorized as a) Primary school or below; b) Middle school; c) High school; d) Junior college; and e) Bachelor’s degree and above | Actual | PQEEPH | | Non-significant | PQEEPH | Respondents with education of junior college (compared to primary school or below) were less likely to be depressed (OR=.202, p=.035); Non-significant  for other categories. | |  |
|  |  | Place of residence | Categorized as a) Urban; and b) Rural | Actual |  | | Non-significant |  | Non-significant | |  |
|  |  | Household monthly income | Categorized as a) <600 Yuan; b) 600-6,000 Yuan; and c) >6000 Yuan | Actual |  | | Household monthly income of higher than 6,000 Yuan (compared to <600 Yuan) was associated with lower anxiety (OR=.416, p=.031); Not significant for other categories |  | Non-significant | |  |

**Table 5 (Continued)**

*The Main Outcomes of the Studies Included in the Systematic Review*

| Study ID | Quality Assessment | SEC  Variable | Measurement  Method | Actual / Perceived | | Anxiety    Inventory Outcomes | | Depression    Inventory Outcomes | | Note | |
| --- | --- | --- | --- | --- | --- | --- | --- | --- | --- | --- | --- |
|  |  |  |  |  |  | |  |  |  | |  |
| #2936 | 8 | Traumatic economic stress | 4-item subscale of the COVID-19 traumatic stressor scale using a 5-point scale (1=Not at all and 5=Very much) | Perceived | GAD-7 | | Higher economic stress due to COVID-19 predicted higher anxiety (β=.17, p=.008) | PHQ-9 | Non-significant | |  |
|  |  | SES | Categorized as a) Very low; b) Low; c) Middle; and d) High | Perceived |  | | Non-significant |  | Non-significant | |  |
|  |  | Education | Categorized as a) Read/write proficiency; b) Intermediate level; c) College or university; and d) Graduate degree | Actual |  | | Non-significant |  | Non-significant | |  |
|  |  |  |  |  |  | |  |  |  | |  |
| #2920 | 8 | Education | Categorized as a) <Undergraduate/junior college; b) Undergraduate/junior college; and c) ≥Postgraduate | Actual | HADS | | Frontliners: Non-significant  Non-frontliners: Non-significant | HADS | Frontliners: Non-significant  Non-frontliners: Non-significant | |  |
| #2919 | 8 | Food insecurity | 18-item USDA household food insecurity module | Perceived | PHQ-4 | | Increasing levels of food insecurity was associated with higher odds of anxiety (p=<.001) | PHQ-4 | Increasing levels of food insecurity was associated with higher odds of depression (p=<.001) | |  |
| #2912 | 8 | COVID-19 being a personal financial threat | 1-item binary variable (Yes/No) assessing whether the respondents consider COVID-19 a personal financial threat to them or not | Perceived | DASS-21 | | Considering COVID-19 as a personal financial threat was associated with higher anxiety (β=-.28, p=0) | DASS-21 | Considering COVID-19 as a personal financial threat was associated with higher depression (β =-.33, p=0) | |  |
| #2910 | 8 | Current employment and financial impact | Measured on a scale of 0 to 19 | Perceived | NA | | NA | EPDS | Pregnant women had higher depressive symptoms when they experienced more impacts to their current employment and finances (Spearman’s ρ=.18) | |  |
|  |  | Expected employment and financial impact | Measured on a scale of 0 to 22 | Perceived |  | | NA |  | Non-significant | |  |
|  |  |  |  |  |  | |  |  |  | |  |
|  |  |  |  |  |  | |  |  |  | |  |
|  |  |  |  |  |  | |  |  |  | |  |

**Table 5 (Continued)**

*The Main Outcomes of the Studies Included in the Systematic Review*

| Study ID | Quality Assessment | SEC  Variable | Measurement  Method | Actual / Perceived | | Anxiety    Inventory Outcomes | | Depression    Inventory Outcomes | | Note | |
| --- | --- | --- | --- | --- | --- | --- | --- | --- | --- | --- | --- |
|  |  |  |  |  |  | |  |  |  | |  |
| #2890 | 8 | Education | Categorized as a) Below university; b) College; and c) Master’s or doctorate | Actual | SAS | | For males, not significant; For females, having a Master’s or doctorate was associated with higher odds of anxiety (OR=.37, p=<.005) but no difference was observed between college degree and below university | PHQ-9 | Non-significant | |  |
|  |  | Place of residence | Categorized as a) Rural; and b) Urban | Actual |  | | Non-significant |  | Non-significant | |  |
|  |  | Family income (RMB) of <100,000 | 1-item binary variable (Yes/No) assessing whether the respondent’s family income was <100,000 or not | Actual |  | | Income of below RMB 100,000 was associated with higher anxiety for males (OR=1.99, p=<.005) and females (OR=1.78, p=<.005) |  | For females, not significant; For males, income of below RMB 100,000 was associated with higher depression (OR=1.37, p=<.005) | |  |
| #2888 | 8 | Education | Categorized as a) Primary; b) Secondary; and c) Tertiary | Actual | DASS-21 | | Non-significant | DASS-21 | Non-significant | |  |
|  |  | Fear of job loss | Categorized as a) Afraid; and b) Not afraid | Perceived |  | | Compared to subjects who are not afraid of job loss, subjects who are afraid of job loss have significantly higher anxiety |  | Compared to subjects who are not afraid of job loss, subjects who are afraid of job loss have significantly higher depression | |  |
|  |  | Income | Categorized as a) Less than before; and b) Same or more than before | Actual |  | | Non-significant |  | Non-significant | |  |
| #2881 | 8 | SES | SES index was composed from the average score of three questions assessing level of education, subjective perception of SES, and financial resources for the next 3 months. | Hybrid | GAD-7 | | SES predicted higher anxiety (β=-.138, p=<.01) | PHQ-9 | SES predicted higher depression (β=-.100, p=<.01) | |  |
| #2879 | 7 | Financial hardship | Categorized as a) Not at all; b) A little; c) Somewhat; and d) Very difficult | Perceived | GAD-2 | | Experiencing financial hardship was associated with higher anxiety | PHQ-2 | Experiencing financial hardship was associated with higher depression | |  |

**Table 5 (Continued)**

*The Main Outcomes of the Studies Included in the Systematic Review*

| Study ID | Quality Assessment | SEC  Variable | Measurement  Method | Actual / Perceived | | Anxiety    Inventory Outcomes | | Depression    Inventory Outcomes | | Note | |
| --- | --- | --- | --- | --- | --- | --- | --- | --- | --- | --- | --- |
|  |  |  |  |  |  | |  |  |  | |  |
| #2874 | 8 | Annual household income | Categorized as a) >200 thousand Yuan; b) 30-100 thousand Yuan; and c) 100-200 thousand Yuan | Actual | NA | | NA | PHQ-9 | Non-significant | | Please see Table 6 for T2 |
|  |  | Education | Categorized as a) Bachelor degree or below level; and b) Master degree or higher level | Actual |  | | NA | PHQ-9 | Non-significant | |  |
| #2868 | 7 | Education | Categorized as a) Primary; b) Secondary; and c) Tertiary | Actual | PHQ-4 | | Non-significant | PHQ-4 | Non-significant | |  |
|  |  | Change in income since the outbreak | Categorized as a) No change; b) Small reduction; c) Reduction by half; d) Large reduction; and e) Unemployed | Actual |  | | Small reduction (OR=3.55, p=<.001), reduction by half (OR=4.52, p=<.001), large reduction (OR=4.43, p=<.001), and unemployed (OR=5.61, p=<.001) were all associated with higher anxiety symptoms |  | Small reduction (OR=1.87, p=<.05), reduction by half (OR=2.68, p=<.001), large reduction (OR=2.32, p=<.01), and unemployed (OR=3.53, p=<.001) were all associated with higher depressive symptoms | |  |
| #2852 | 8 | COVID-19 related concerns regarding financial security | 4-point Likert scale to the question: “Are you concerned about potential financial losses due to the coronavirus crisis?” | Perceived | GAD-7 | | Non-significant | WHO-5 | Non-significant | |  |
|  |  | Education | Whether the respondent’s educational level is higher or lower than the enrolment standard | Actual |  | | Non-significant |  | Non-significant | |  |
|  |  |  |  |  |  | |  |  |  | |  |
|  |  |  |  |  |  | |  |  |  | |  |
|  |  |  |  |  |  | |  |  |  | |  |

**Table 5 (Continued)**

*The Main Outcomes of the Studies Included in the Systematic Review*

| Study ID | Quality Assessment | SEC  Variable | Measurement  Method | Actual / Perceived | | Anxiety    Inventory Outcomes | | Depression    Inventory Outcomes | | Note | |
| --- | --- | --- | --- | --- | --- | --- | --- | --- | --- | --- | --- |
|  |  |  |  |  |  | |  |  |  | |  |
| #2846 | 8 | Food insecurity | Household Food Security Screener Short Form | Perceived | PHQ-4 | | Food insecurity is associated with higher prevalence of anxiety | PHQ-4 | Food insecurity is associated with higher prevalence of depression | |  |
|  |  | Education | Categorized as a) High school or less; b) Associate’s degree; c) Some college; d) Bachelor’s degree; and e) Graduate degree | Actual |  | | Non-significant |  | Non-significant | |  |
|  |  | Employment status | Categorized as a) Working full time; b) Working part time/hours reduced; c) Unemployed/seeking work/furloughed; and d) Out of labor force | Actual |  | | Non-significant |  | Non-significant | |  |
|  |  | Job loss | Categorized as a) Yes; b) No; and c) Refused | Actual |  | | Job loss was associated with higher odds of anxiety (aOR=1.41, p=<.001) |  | Job loss was associated with higher odds of depression (aOR=1.34, p=<.001) | |  |
|  |  | Income | Categorized as a) <$20,000; b) $20,000-39,999; c) $40,000-69,999; d) $70,000-99,999; e) $100,000-149,999; f) $150,000 or more; and g) Refused | Actual |  | | Non-significant |  | Non-significant | |  |
| #2822 | 8 | SES | Categorized as a) Lower than average; b) Average; c) Higher than average | Perceived | HADS | | Higher SES was significantly  associated with lower odds of anxiety (OR=0.26, p=.009) | HADS | Higher SES was significantly  associated with lower odds of depression (OR=-0.30, p=.002) | |  |
|  |  | Occupation | Categorized as a) Healthcare; b) Businessman; c) Engineer; d) Teacher; e) Housewife; f) Student; and g) Others | Actual |  | | Non-significant |  | Non-significant | |  |
| #2819 | 8 | Employment status | Categorized as a) Job loss; b) Furloughed; c) Previously unemployed; d) Working (travelling); and e) Work from home | Actual | DASS-21 | | The final model for anxiety and employment status was statistically significant [F(24,689)=16.98, p=<.001] | DASS-21 | The final model for depression and employment status was statistically significant [F(24,690)=25.76, p=<.001] | |  |
|  |  |  |  |  |  | |  |  |  | |  |

**Table 5 (Continued)**

*The Main Outcomes of the Studies Included in the Systematic Review*

| Study ID | Quality Assessment | SEC  Variable | Measurement  Method | Actual / Perceived | | Anxiety    Inventory Outcomes | | Depression    Inventory Outcomes | | Note | |
| --- | --- | --- | --- | --- | --- | --- | --- | --- | --- | --- | --- |
|  |  |  |  |  |  | |  |  |  | |  |
| #2815 | 8 | Job insecurity | Job Insecurity Scale | Perceived | GAD-7 | | There is a positive and highly significant impact of job security on anxiety (β=.213, p=.001) | CESD-20 | There is a positive and highly significant impact of job security on depression (β=.293, p=<.001) | |  |
| #2811 | 8 | Family’s financial impact due to COVID-19 | 1-item binary variable (Yes/No) assessing whether the respondent’s family’s financial situation was impacted by COVID-19 or not | Perceived | BASC-3 | | Non-significant | BASC-3 | Non-significant | |  |
| #2809 | 7 | Parents’ education | Categorized as a) Degree; and b) No degree | Actual | SCARED | | Non-significant | CES-DC | Non-significant | |  |
|  |  | Parents’ employment status | Unclear | Actual |  | | Non-significant |  | Children and adolescents whose parents suffered job loss were more likely to present clinical depression scores (p=<.005) | |  |
| #2773 | 11 | Education | Binary variable (Yes/No) indicating whether or not at least some secondary education was received | Actual | NA | | NA | PHQ-8 | This variable is not used in T1 | | Please see Table 4 for T2 |
|  |  | Employment status | Binary indicator (Yes/No) of whether the respondent was engaged in activity resulting in monetary, food or other forms of compensation | Actual |  | | NA |  | This variable is not used in T1 | |  |
|  |  | Monthly income | Binary variable (Yes/No) indicating whether or not at least 100,000 Ush is earned per month | Actual |  | | NA |  | This variable is not used in T1 | |  |
|  |  | Food security | 5 items adapted from the Food Insecurity Experience Scale | Perceived |  | | NA |  | This variable is not used in T1 | |  |
| #2765 | 8 | Education | Categorized as a) Under diploma; b) Diploma; and c) Academic | Actual | GAD-7 | | Non-significant | PHQ-9 | Non-significant | |  |

**Table 5 (Continued)**

*The Main Outcomes of the Studies Included in the Systematic Review*

| Study ID | Quality Assessment | SEC  Variable | Measurement  Method | Actual / Perceived | | Anxiety    Inventory Outcomes | | Depression    Inventory Outcomes | | Note | |  |  |  |
| --- | --- | --- | --- | --- | --- | --- | --- | --- | --- | --- | --- | --- | --- | --- |
|  |  |  |  |  |  | |  |  |  | |  |  |  |  |
| #7242 | 7 | Neighbourhood deprivation | Area deprivation index (ADI), categorized as a) lowest; b) second; c) third; and d) highest | Actual | NA | | NA | K6 | Non-significant | |  |  |  |  |
| #7206 | 8 | Housing | The housing-condition dimension consists of three items: the number and size of rooms, living conditions (i.e., heating, sanitation, and lighting facilities), and the exterior of the property and surrounding landscape. | Actual | Generalised Anxiety Self-Rating Scale | | Better housing associated with decreased anxiety (B = -0.152, p <.000) | NA | NA | |  |  |  |  |
|  |  |  |  |  |  | |  |  |  | |  |  |  |  |
|  |  |  |  |  |  | |  |  |  | |  |  |  |  |
|  |  | Income | Categorised as a) < ¥3750; b) ¥3750 – 10000; c) ¥10000-30000; and d) > ¥30000 | Actual |  | | Higher income associated with decreased anxiety (B = -0.108, p< .05) |  | NA | |  |  |  |  |
|  |  | Education | Categorised as a) Primary Education; b) Secondary Education; and c) Higher Education | Actual |  | | Having higher education associated with decreased anxiety (B = -0.296, p < .000) |  | NA | |  |  | NA | Convenience |
|  |  |  |  |  |  | |  |  |  | |  |  |  |  |
| #6641 | 8 | Education | Categorized as a) None; b) Secondary / vocational and below; c) Polytechnic / university; and d) Postgraduate | Actual | NA | | NA | GHQ-12 | Non-significant | |  |  |  |  |
|  |  | Employment Status | Categorized as a) Work at the office; b) Work from home c) Stopped work but to resume after lockdown; d) No work before and during the lockdown; and e) Lost work due to the lockdown | Actual |  | | NA |  | Non-significant | |  |  |  |  |
|  |  | Financial stress | A binary response (Yes/No) to the question: "Since the lockdown started, have you experienced increased financial stress?" | Perceived |  | | NA |  | Non-significant | |  |  | NA | Convenience |

**Table 5 (Continued)**

*The Main Outcomes of the Studies Included in the Systematic Review*

| Study ID | Quality Assessment | SEC  Variable | Measurement  Method | Actual / Perceived | | Anxiety    Inventory Outcomes | | Depression    Inventory Outcomes | | Note | |  |  |  |
| --- | --- | --- | --- | --- | --- | --- | --- | --- | --- | --- | --- | --- | --- | --- |
|  |  |  |  |  |  | |  |  |  | |  |  |  |  |
| #5771 | 8 | Availability of economic income | Yes / No | Actual | STAI | | Non-significant | BDI-11 | Having an income was associated with lower depression (β = -1.61, p = .04) | |  |  | NA | Convenience |
| #5662 | 4 | Education | Categorised as a) Primary education; b) Secondary education; c) Higher education; and d) Jobless | Actual | DASS Anxiety | | Non-significant | DASS Depression | Non-significant | |  |  |  |  |
|  |  | Occupation | Categorised as a) Entrepreneur; and b) Employee | Actual |  | | Non-significant |  | Non-significant | |  |  |  |  |
| #5344 | 8 | Occupation | Categorised as a) Entrepreneur; and b) Employee | Actual | GAD-7 | | Non-significant | PHQ-8 | Non-significant | |  |  |  |  |
|  |  | COVID-19 economic threat | 1-item variable asking the respondent to indicate, from 1 to 10, their level of concern about finances due to the COVID-19 pandemic | Perceived |  | | COVID-19 economic threat was associated with higher anxiety symptoms (aOR=1.585, p=<.01) |  | COVID-19 economic threat was associated with higher depressive symptoms (aOR=1.445, p=<.05) | |  |  |  |  |
| #5307 | 7 | Education | Categorized as a) College; b) Bachelor; c) Master; and d) Others | Actual | GAD-7 | | Teachers in the "others" education category (i.e., not college, Bachelor or Master) experienced higher incidence of anxiety (OR=1.17, p=<0.05) | NA | NA | |  |  |  |  |
|  |  | Occupation | Categorised as a) Primary school teacher; b) Junior school teacher; c) High school teacher; and d) University teacher | Actual |  | | High school teachers were less likely to experience anxiety compared to primary school teachers (OR=0.89, p=<0.05) |  | NA | |  |  |  |  |

**Table 5 (Continued)**

*The Main Outcomes of the Studies Included in the Systematic Review*

| Study ID | Quality Assessment | SEC  Variable | Measurement  Method | Actual / Perceived | | Anxiety    Inventory Outcomes | | Depression    Inventory Outcomes | | Note | |  |  |  |
| --- | --- | --- | --- | --- | --- | --- | --- | --- | --- | --- | --- | --- | --- | --- |
|  |  |  |  |  |  | |  |  |  | |  |  |  |  |
| #5260 | 8 | SES before Covid-19 | Categorised as a) No answer; b) Rich; c) Middle class; d) Middle to low; and e) below poverty line | Perceived | LAS-10 | | Subjects' perceived socioeconomic status before COVID was significantly associated with anxiety (p < .001, eta squared = .056); compared to subjects below poverty line, subjects in other socioeconomic classes have significantly lower anxiety (p < .05). | NA | NA | |  |  |  |  |
|  |  |  |  |  |  | |  |  |  | |  |  |  |  |
|  |  |  |  |  |  | |  |  |  | |  |  |  |  |
|  |  | SES after Covid-19 | Categorised as a) No answer; b) Rich; c) Middle class; d) Middle to low; and e) below poverty line | Perceived |  | | Subjects' perceived socioeconomic status after COVID was significantly associated with anxiety (p < .05, eta squared = .024). |  | NA | |  |  |  |  |
|  |  | Health coverage | Categorised as a) No health coverage; b) Private insurance; c) Social security; and d) Other public coverage | Actual |  | | Non-significant |  | NA | |  |  |  |  |
|  |  | Household income | Categorised as a) <675k LP; b) 675k-1500k LP; c) 1500k-3000k LP; and d) >3000k LP | Actual |  | | Non-significant |  | NA | |  |  |  |  |
|  |  | Socioeconomic quartile | Categorized as a) Quartile 1; b) Quartile 2; c) Quartile 3; and d) Quartile 4 (highest SEC) | Actual |  | | Non-significant |  | NA | |  |  |  |  |

**Table 5 (Continued)**

*The Main Outcomes of the Studies Included in the Systematic Review*

| Study ID | Quality Assessment | SEC  Variable | Measurement  Method | Actual / Perceived | | Anxiety    Inventory Outcomes | | Depression    Inventory Outcomes | | Note | |  |  |  |
| --- | --- | --- | --- | --- | --- | --- | --- | --- | --- | --- | --- | --- | --- | --- |
|  |  |  |  |  |  | |  |  |  | |  |  |  |  |
| #5235 | 6 | Food insecurity | Categorised as a) Food insecure; and b) Food secure | Perceived | GAD-7 | | People who are food insecure have higher risk for anxiety (OR = 2.93, p < .001). | NA | NA | |  |  |  |  |
|  |  | Education | Categorised as a) Less than high school; b) High school diploma; c) Trades certificate; d) College diploma; e) University diploma or certificate below the bachelor's level; and f) Bachelor's degree; and g) an advanced degree | Actual |  | | Non-significant |  |  | |  |  |  |  |
|  |  | Employment security | Categorised as a) Does not expect to lose job; b) Might lose job; c) Not employed | Perceived |  | | Compared to people who do not expect to lose their job, people who expect that they might lose their job have higher risk for anxiety (OR = 1.97, p < .01). |  |  | |  |  |  |  |
|  |  | Financial impact of pandemic | Categorised as a) No impact; b) Impacted; and c) Too soon to tell | Perceived |  | | Using people who feel no financial impact as the reference group, people who feel financial impact have higher risk for anxiety (OR = 1.91, p < .05); people who stated that it is too soon to tell have higher risk for anxiety (OR = 1.96, p < .01). |  |  | |  |  |  |  |
| #5204 | 8 | Deterioration of household economy | A 7-point Likert Scale (1 = Not at all and 7 = Extremely) to the question: "The family budget has tightened" | Perceived | K6 | | Higher deterioration of household economy was associated with higher mild-to-moderate and serious distress (β = .04, p = .018; β = .13, p < .001) | PHQ-9 | Non-significant | |  |  |  |  |
|  |  |  |  |  |  | |  |  |  | |  |  |  |  |
|  |  |  |  |  |  | |  |  |  | |  |  |  |  |

**Table 5 (Continued)**

*The Main Outcomes of the Studies Included in the Systematic Review*

| Study ID | Quality Assessment | SEC  Variable | Measurement  Method | Actual / Perceived | | Anxiety    Inventory Outcomes | | Depression    Inventory Outcomes | | Note | |  |  |  |
| --- | --- | --- | --- | --- | --- | --- | --- | --- | --- | --- | --- | --- | --- | --- |
|  |  |  |  |  |  | |  |  |  | |  |  |  |  |
| #5080 | 8 | Change in employment | Categorised as: a) No change in employment; b) Contact with work colleagues greatly reduced; c) Working reduced hours; d) Unemployed prior to the pandemic; e) Lost employment; and f) Others (increased in working hours) | Actual | NA | | NA | PHQ-9 | Non-significant | |  |  |  |  |
|  |  | Covid-19 related financial strains | Categorised as: a) Rent/mortgage; b) Utilities (water, gas, etc.); c) Food/groceries, d) Financially supporting others, and e) Others (medication, health care etc.) | Actual |  | | NA |  | Financial strain posed a higher risk for both depression (OR= 1.85) and having thoughts that they would be better off dead or of hurting themselves (OR= 1.80) | |  |  |  |  |
| #4946 | 8 | Education | Categorised as a) Illiterate; b) Elementary school; c) High school; and d) Higher education | Actual | BAI | | Non-significant | BDI-II | Non-significant | |  |  |  |  |
|  |  | Family income | Categorised as a) < 1 wage; b) ≥ 1 wage < 3 minimum wages; and c) ≥ 3 minimum wages | Actual |  | | Non-significant |  | Non-significant | |  |  |  |  |
|  |  | Financial aid from government | Yes / No | Actual |  | | Those who received financial aid from the government showed reduced anxiety (β =-10.01, p = .042). |  | Non-significant | |  |  |  |  |

**Table 5 (Continued)**

*The Main Outcomes of the Studies Included in the Systematic Review*

| Study ID | Quality Assessment | SEC  Variable | Measurement  Method | Actual / Perceived | | Anxiety    Inventory Outcomes | | Depression    Inventory Outcomes | | Note | |  |  |  |
| --- | --- | --- | --- | --- | --- | --- | --- | --- | --- | --- | --- | --- | --- | --- |
|  |  |  |  |  |  | |  |  |  | |  |  |  |  |
| #4852 | 8 | Parents’ education | Categorised as a) High; b) Middle; c) Low; and d) do not know | Actual | CES-D | | Non-significant | NA | NA | |  |  |  |  |
|  |  | Education | Categorised as a) Bachelor; b) Master; and c) PhD | Actual |  | | Non-significant |  |  | |  |  |  |  |
| #4774 | 8 | Food security | USDA Household Food Security Survey: a Six-Item Short Form | Actual | GHQ-12 | | Food insecurity (aOR=17.06, p=<.001) was found to be a significant factor associated with psychological distress | GHQ-12 | Food insecurity (aOR=17.06, p=<.001) was found to be a significant factor associated with psychological distress | |  |  |  |  |
| #4762 | 8 | Financial aid | A binary response (Yes / No) to whether they would request financial aid if a stay-home order recurred due to a Covid-19 resurgence | Perceived | GAD-7 | | Non-significant | PHQ-9 | Non-significant | |  |  |  |  |
|  |  | Food aid | A binary response (Yes / No) to whether they would request food aid if a stay-home order recurred due to a Covid-19 resurgence | Perceived |  | | Anxiety was higher in participants who said they would request food aid (OR = 2.50, p = .01) |  | Depression was higher in participants who said they would request food aid (OR = 1.99, p = .01) | |  |  |  |  |

**Table 5 (Continued)**

*The Main Outcomes of the Studies Included in the Systematic Review*

| Study ID | Quality Assessment | SEC  Variable | Measurement  Method | Actual / Perceived | | Anxiety    Inventory Outcomes | | Depression    Inventory Outcomes | | Note | |  |  |  |
| --- | --- | --- | --- | --- | --- | --- | --- | --- | --- | --- | --- | --- | --- | --- |
|  |  |  |  |  |  | |  |  |  | |  |  |  |  |
| #4670 | 8 | Occupation | Categorised as: a) Business; b) Govt. services; c) Private MNCs; d) Student; and e) Unemployed | Actual | NA | | NA | DASS-21 | Non-significant | |  |  |  |  |
|  |  | Education | Categorised as: a) Primary; b) Secondary; c) Graduate; and d) post-graduate | Actual |  | | NA |  | Non-significant | |  |  |  |  |
| #4447 | 8 | Education | Categorised as a) High school diploma or less; b) Graduate diploma; c) Bachelor; and d) Masters/Doctorate | Actual | NA | | NA | CES-D | Having high school diploma or less was associated with higher odds of depression (OR=1.58, p=<.001) | |  |  |  |  |
|  |  | Income | Categorised as a) Poor; b) Moderate; and c) Good | Perceived |  | | NA |  | Having poor (OR=3.17, p=<.001) and moderate (OR=1.46, p=<.001) perceived income were associated with higher odds of depression | |  |  |  |  |
| #7203 | 11 | Income | Categorized as (in £) a) < 15000; b) 15000 to < 30000; c) 30000 to 45000; and d) ≥45000 | Actual | K6 | | This variable is used in T2 only | K6 | This variable is used in T2 only | | Please see Table 4 for T2 |  |  |  |
|  |  |  |  |  |  | |  |  |  | |  |  |  |  |
| #6670 | 11 | Perceived financial risk due to Covid-19 | A single-item that asked participants to report the percent chance they will run out of money because of the coronavirus in the next three months. | Perceived | PHQ-4 | | This variable is used in T2 only | PHQ-4 | This variable is used in T2 only | | Please see Table 4 for T2 |  |  |  |

**Table 5 (Continued)**

*The Main Outcomes of the Studies Included in the Systematic Review*

| Study ID | Quality Assessment | SEC  Variable | Measurement  Method | Actual / Perceived | | Anxiety    Inventory Outcomes | | | Depression    Inventory Outcomes | | Note | |  |  |  |
| --- | --- | --- | --- | --- | --- | --- | --- | --- | --- | --- | --- | --- | --- | --- | --- |
|  |  |  |  |  |  | |  | |  |  | |  |  |  |  |
| #3705 | 8 | Annual family income | Categorised as a) 35,000 USD or less; and b) More than 35,000 USD | Actual | NA | | NA | | CES-D | Higher level of annual family income (p<0.001) was significantly correlated with lower depressive symptoms scores. | |  |  |  |  |
|  |  | Food security | 6-item short form in the U.S.A. Household FoodSecurity Survey Module | Actual |  | | NA | |  | Food security (t=-2.28, p=0.03) was significantly correlated with lower depressive symptoms scores. | |  |  |  |  |
|  |  | Negative economic change | Categorised as a) Loss of employment; b) Working less hours; and c) Inability to find a job | Actual |  | | NA | |  | Non-significant | |  |  |  |  |
|  |  | Receipt of financial benefits | Categorised as a) Government-issued relief checks; b) Unemployment benefits; c) Small business support; and d) Student loans relief | Actual |  | | NA | |  | Receipt of any financial benefits (p=0.003) was significantly correlated with lower depressive symptoms scores. | |  |  |  |  |
| #3685 | 8 | Education | Completed / Did not complete high school | Actual | DASS-21 and Children's anxiety scale | | Parents who did not complete high school reported somewhat higher levels of anxiety. But has no significant effect on child anxiety | DASS-21 and Short Mood and Feelings Questionnaire | | Non-significant for both parent and child's depression | |  |  |  |  |
|  |  | Annual household income  Financial deprivation | Categorized as "Low income" if <= AUD 52000  Summed seven item on money shortages in the year prior to the pandemic (e.g., unable to pay bills / mortgage / rent on time) | Actual  Actual |  | | Non-significant  Financial deprivation showed small associations with higher parent anxiety and very small associations with child anxiety |  | | Non-significant  Financial deprivation showed small associations with higher parent depression but has no significant effect on child's depression | |  |  |  |  |

**Table 5 (Continued)**

*The Main Outcomes of the Studies Included in the Systematic Review*

| Study ID | Quality Assessment | SEC  Variable | Measurement  Method | Actual / Perceived | | Anxiety    Inventory Outcomes | | Depression    Inventory Outcomes | | Note | |  |  |  |
| --- | --- | --- | --- | --- | --- | --- | --- | --- | --- | --- | --- | --- | --- | --- |
|  |  |  |  |  |  | |  |  |  | |  |  |  |  |
| #3620 | 8 | COVID-19 stressors - financial uncertainty | 12-items 4-point Likert scale (0 = not at all to 3 = sometimes) | Perceived | DASS-21 | | Higher financial uncertainty was associated with higher anxiety (B = 0.21, p < .05) | DASS-21 | Higher financial uncertainty was associated with higher depression (B = 0.38, p < .01) | |  |  |  |  |
|  |  |  |  |  |  | |  |  |  | |  |  |  |  |
|  |  | COVID-19 stressors - inadequate food supply | 12-items 4-point Likert scale (0 = not at all to 3 = sometimes) | Perceived |  | | Non-significant |  | Non-significant | |  |  |  |  |
|  |  | Education | Categorized by the current degree: Ph.D. Scholars, MPhil, Masters, and Graduates. | Actual |  | | Higher educational degree was associated with higher anxiety (B = 0.21, p < .05) |  | Higher educational degree was associated with higher anxiety (B = 0.28, p < .01) | |  |  |  |  |
| #3419 | 8 | Education  Employment status  Perceived food insecurity  Healthcare insurance | Categorized as a) No school or incomplete primary education;  b) Primary or high school education; and c) College or higher education  Categorized as a) Unemployed;  b) Employed before lockdown; and  c) Employed before and during lockdown  Categorized as a) Food insecure; and  b) Not food insecure  Categorized as a) Medically insured; and b) Uninsured | Actual  Actual  Perceived  Actual | NA | | NA  NA  NA  NA | PHQ-9 | Non-significant  Non-significant  People who were food insecure have higher risk of depression (p < .05, aOR = 2.12)  Non-significant | |  |  |  |  |
| #3344 | 8 | Adult income loss | Categorized as a) None lost; b) Partial; and c) Full | Perceived | NA | | NA | PHQ-2 | Non-significant | |  |  |  |  |
| #3282 | 11 | Economic impact payments | Yes / No | Actual | GAD-2 | | This variable is used in T2 only | PHQ-2 | This variable is used in T2 only | | Please see Table 4 for T2 |  |  |  |

**Table 5 (Continued)**

*The Main Outcomes of the Studies Included in the Systematic Review*

| Study ID | Quality Assessment | SEC  Variable | Measurement  Method | Actual / Perceived | | Anxiety    Inventory Outcomes | | Depression    Inventory Outcomes | | Note | |
| --- | --- | --- | --- | --- | --- | --- | --- | --- | --- | --- | --- |
|  |  |  |  |  |  | |  |  |  | |  |
| #3267 | 7 | Education | Categorized as: a) Elementary, b) Preparatory, c) Secondary, d) University/higher education, e) Postgraduate studies, and f) Others | Actual | GAD-7 | | Non-significant | NA | NA | |  |
|  |  |  |  |  |  | |  |  |  | |  |
|  |  | Employment status | Categorized as: a) Full-time employee; b) Freelancer; c) Student; d) Retired; e) Unemployed; and f) Others | Actual |  | | Non-significant |  | NA | |  |
|  |  |  |  |  |  | |  |  |  | |  |
|  |  | Work status during Covid | Categorized as: a) Still working, b) Tele-working, c) Suspended from work | Actual |  | | Being suspended from work was associated with an increase in the likelihood of anxiety symptoms (OR = 1.57, p < .001) |  | NA | |  |
|  |  |  |  |  |  | |  |  |  | |  |
|  |  |  |  |  |  | |  |  |  | |  |
| #3242 | 8 | Socioeconomic position (SEP) | Categorized into a) Low; b) Medium; and c) High based on a number of measures (e.g., parents' education, number of books at home, number of cars etc.) | Perceived | NA | | NA | HSCL  (6-items) | Low family SEP is associated with higher odds of high depressive symptoms in boys (OR = 2.33, p < .001) and girls (OR = 1.66, p < .01) compared with high SEP | | Please see Table 4 for T2 |
|  |  |  |  |  |  | |  |  |  | |  |
| #3212 | 8 | Income (prior Covid-19) | Categorized into quintiles (1 = Poor and 5 = Rich) | Perceived | GAD | | Quintile 5 (richest) has significantly lower anxiety (OR = 0.832, p < .05) compared to Quintile 1 (poorest). But this was not observed in the other quintiles. | PHQ | Quintile 5 (richest) has significantly lower depression (OR = 0.837, p < .05) compared to Quintile 1 (poorest). But this was not observed in the other quintiles. | |  |
|  |  |  |  |  |  | |  |  |  | |  |
|  |  | Education | Categorized as a) 0 years; b) 1-7 years; c) 7-10 years; d) 10-13 years; and e) 13+ years | Actual |  | | Those with 10-13 years education have significantly lower anxiety (OR = 0.884, p < .05) compared to 0 years. But this was not observed in other years of education. |  | Those with 10-13 years education (OR = 0.881, p < .05) and those with 13+ years (OR = 0,810, p < .05) have significantly lower depression compared to 0 years. But this was not observed in other years of education. | |  |
|  |  |  |  |  |  | |  |  |  | |  |

**Table 5 (Continued)**

*The Main Outcomes of the Studies Included in the Systematic Review*

| Study ID | Quality Assessment | SEC  Variable | Measurement  Method | Actual / Perceived | | Anxiety    Inventory Outcomes | | Depression    Inventory Outcomes | | | Note | | |
| --- | --- | --- | --- | --- | --- | --- | --- | --- | --- | --- | --- | --- | --- |
|  |  |  |  |  |  | |  |  |  | | |  | |
| #3198 | 8 | Occupation | Categorized as a) Nursing; b) Medical; c) Allied health; d) Administrative staff; and e) other roles. | Actual | GAD-7 | | Independent workplace predictors for worse anxiety outcomes  included having a nursing, allied health or other non-medical role | PHQ-9 | Independent workplace predictors for worse depression outcomes  included having a nursing, allied health or other non-medical role | | |  | |
|  |  |  |  |  |  | |  |  |  | | |  | |
|  |  | Concerns or worries about household income | Yes / No | Perceived |  | | Concerns about household income was associated with higher anxiety (OR = 1.96, p = .001) |  | Concerns about household income was associated with higher depression (OR = 1.29, p = .001) | | |  | |
|  |  |  |  |  |  | |  |  |  | | |  | |
| #3188 | 8 | Neighbourhood deprivation | Area Deprivation Index (ADI) | Actual | PROMIS-29 | | There were main effects of neighbourhood deprivation level for anxiety (p = .048, n2 = .063). Pairwise comparisons found those residing in neighbourhoods with the highest deprivation endorsed worse symptoms relative to those residing in neighbourhoods with the lowest ADI. | PROMIS-29 | | There were main effects of neighbourhood deprivation level for depression (p = .019, n2 = .081). Pairwise comparisons found those residing in neighbourhoods with the highest deprivation endorsed worse symptoms relative to those residing in neighbourhoods with the lowest ADI. | | |  |
|  |  |  |  |  |  | |  |  | |  | | |  |
| #3187 | 8 | Housing | House or apartment | Actual | HAD-14 | | Non-significant | HAD-14 | Non-significant | | |  | |
|  |  |  |  |  |  | |  |  |  | | |  | |
|  |  | Presence of garden | Yes / No | Actual |  | | Non-significant |  | Non-significant | | |  | |
|  |  |  |  |  |  | |  |  |  | | |  | |
|  |  | Whole area of the house | Measured in m2 | Actual |  | | Non-significant |  | Non-significant | | |  | |
|  |  |  |  |  |  | |  |  |  | | |  | |
|  |  | Living space per person | Measured in m2 | Actual |  | | Non-significant |  | Non-significant | | |  | |
|  |  |  |  |  |  | |  |  |  | | |  | |
| #3180 | 8 | Perceived economic status | 6-point Likert scale (1 = Very bad and 6 = Very good) to the question: “In your circumstances, do you consider your household’s economic status to be good or bad?” | Perceived | NA | | NA | CESD-SF | The direct effect of perceived economic status on depression was non-significant.. | | |  | |
|  |  |  |  |  |  | |  |  |  | | |  | |

**Table 5 (Continued)**

*The Main Outcomes of the Studies Included in the Systematic Review*

| Study ID | Quality Assessment | SEC  Variable | Measurement  Method | Actual / Perceived | | Anxiety    Inventory Outcomes | | Depression    Inventory Outcomes | | Note | |
| --- | --- | --- | --- | --- | --- | --- | --- | --- | --- | --- | --- |
|  |  |  |  |  |  | |  |  |  | |  |
| #3178 | 8 | Educational | Categorized as a) Junior college; b) Undergraduate, and c) Master and above | Actual | NA | | NA | SDS | Both undergraduate level (β = -.630, p < .001) and master & above level (β = -.455. p = .005) were associated with lower depression. | |  |
|  |  |  |  |  |  | |  |  |  | |  |
|  |  | Residence | Categorized as a) City; b) Town, and c) Village | Actual |  | | NA |  | Non-significant | |  |
|  |  |  |  |  |  | |  |  |  | |  |
| #3142 | 8 | Education | Number of years the respondents had received education | Actual | GAD-7 | | Non-significant | NA | NA | |  |
|  |  |  |  |  |  | |  |  |  | |  |
|  |  | Family annual income | Categorized as a) Less than 80 000 Yuan; and b) More than 80 000 Yuan | Actual |  | | Non-significant |  | NA | |  |
|  |  |  |  |  |  | |  |  |  | |  |
| #3105 | 8 | Economic stress | 4-point Likert scale (1 = Not at all and 4 = Very much) on 3-items (i.e., economic loss, livelihood destruction, and lack of basic necessities). The overall score was summed by three items from 3 to 12. | Perceived | NA | | NA | CES-D | Economic stress strengthened the effect of family care needs on depressive symptoms for sandwich-generation caregivers who provide care to both the elderly and children (B = 0.605, p < .05). While in rural areas, the moderation effects of economic stress were only found for elderly caregivers (B=1.106, p < .05). | |  |
|  |  |  |  |  |  | |  |  |  | |  |
|  |  | Education | Categorized as a) Junior high school and below; b) High school/technical school; c) Junior college; d) Undergraduate and e) Postgraduate and above" | Actual |  | | NA |  | Non-significant | |  |
|  |  |  |  |  |  | |  |  | Non-significant | |  |
|  |  | Income | Categorized as a) Low; b) Middle; and c) High | Perceived |  | | NA |  |  | |  |
|  |  |  |  |  |  | |  |  |  | |  |

**Table 5 (Continued)**

*The Main Outcomes of the Studies Included in the Systematic Review*

| Study ID | Quality Assessment | SEC  Variable | Measurement  Method | Actual / Perceived | | Anxiety    Inventory Outcomes | | Depression    Inventory Outcomes | | Note | |
| --- | --- | --- | --- | --- | --- | --- | --- | --- | --- | --- | --- |
|  |  |  |  |  |  | |  |  |  | |  |
| #3104 | 8 | Highest parent education | Categorized as a) Less than secondary; b) Secondary; and c) Higher education | Actual | NA | | NA | CESD-8 | Non-significant | |  |
|  |  |  |  |  |  | |  |  |  | |  |
|  |  | Subjective financial status (before Covid-19) | Students who (strongly) disagreed to the statement - "I had sufficient financial resources to cover my monthly costs", were group together (score 1) | Perceived |  | | NA |  | Those who struggled with current resources were associated with greater depression | |  |
|  |  |  |  |  |  | |  |  |  | |  |
|  |  |  |  |  |  | |  |  |  | |  |
|  |  | Changes in financial situation | Categorized as a) Similar; b) Worse during Covid; and c) Better during Covid | Perceived |  | | NA |  | Those who experienced adverse change were associated with greater depression, compared to no change. But better financial change did not significantly differ from no change. | |  |
|  |  |  |  |  |  | |  |  |  | |  |
| #3097 | 8 | Household income | Categorized as : 1) Less than 200 JD; 2) 200-400 JD; 3) 400-600 JD; 4) 600-800 JD; 5) 800-1000 JD; 6) 1000-1200 JD; 6) 1200-1500 JD; and 7) more than 1500 JD | Actual | NA | | NA | CES-D | Non-significant | |  |
|  |  |  |  |  |  | |  |  |  | |  |
|  |  | Father's education | Categorized as: a) Post-graduate; b) Bachelor; c) Diploma; d) High school; and e) others | Actual |  | | NA |  | Non-significant | |  |
|  |  |  |  |  |  | |  |  |  | |  |
|  |  | Mother's education | Categorized as: a) Post-graduate; b) Bachelor; c) Diploma; d) High school; and e) others | Actual |  | | NA |  | Non-significant | |  |

**Table 5 (Continued)**

*The Main Outcomes of the Studies Included in the Systematic Review*

| Study ID | Quality Assessment | SEC  Variable | Measurement  Method | Actual / Perceived | | Anxiety    Inventory Outcomes | | Depression    Inventory Outcomes | | Note | |
| --- | --- | --- | --- | --- | --- | --- | --- | --- | --- | --- | --- |
|  |  |  |  |  |  | |  |  |  | |  |
| #3081 | 5 | Parental education | Categorized as a) Low; b) Medium; and c) High. | Perceived | German version of SCARED | | Parental education has a low negative correlation with generalized anxiety (B = -.00, aR-squared = .04). | German version of CES-DC | Non-significant | |  |
|  |  |  |  |  |  | |  |  |  | |  |
| #3080 | 8 | Concern about personal finance | Two questions about financial concerns: a) “Right now, how concerned are you about personal finances due to the COVID-19 pandemic?” (0 = Not concerned at all and 100 = Very concerned); and b) “How has the COVID-19 pandemic impacted you financially?” (0 = No impact and 4 = Major impact). | Perceived | GAD-7 | | Concern about finances predicted higher anxiety symptoms (B = 0.05, p < .001). | PHQ-8 | Concern about finances predicted higher depression symptoms (B = 0.03, p = .018). | |  |
|  |  |  |  |  |  | |  |  |  | |  |
| #3070 | 7 | Perceived economic status | Categorized as a) Low; and b) Medium-high | Perceived | NA | | NA | QIDS-SR16 | Low perceived economic status significantly predicted higher depressive symptoms (aOR=2.32, p = .001) | |  |
|  |  |  |  |  |  | |  |  |  | |  |
| #3061 | 7 | Financial burden | Measured on a 5-point Likert-scale (0-4) | Perceived | GAD-2 | | Higher financial burden was associated with higher probable case of anxiety (p < .001) | PHQ-2 | Higher financial burden was associated with higher probable case of depression (p < .001) | |  |
|  |  |  |  |  |  | |  |  |  | |  |
| #3053 | 8 | Financial hardship | Coded as "1" (for responses: "Yes, a little" and "Yes, a lot") and "0" ("No") to the question: "Do you consider yourself to be in financial hardship as a result of the current public health situation?" | Perceived | NA | | NA | CESD-8 | Perceived financial hardship was associated with higher depression ( B = 0.18, p = .030) | |  |
|  |  |  |  |  |  | |  |  |  | |  |
|  |  | Household income | Includes all earnings including for example, from pensions | Actual |  | | NA |  | Non-significant | |  |
|  |  |  |  |  |  | |  |  |  | |  |
|  |  |  |  |  |  | |  |  |  | |  |
|  |  |  |  |  |  | |  |  |  | |  |
|  |  |  |  |  |  | |  |  |  | |  |

**Table 5 (Continued)**

*The Main Outcomes of the Studies Included in the Systematic Review*

| Study ID | Quality Assessment | SEC  Variable | Measurement  Method | Actual / Perceived | | Anxiety    Inventory Outcomes | | Depression    Inventory Outcomes | | Note | |
| --- | --- | --- | --- | --- | --- | --- | --- | --- | --- | --- | --- |
|  |  |  |  |  |  | |  |  |  | |  |
| #3046 | 6 | Education | Categorized as a) Reading and writing level; b) Middle to high school level; c) College level; and d) Graduate studies | Actual | GAD-7 | | Non-significant | PHQ-9 | Non-significant | |  |
|  |  |  |  |  |  | |  |  |  | |  |
|  |  | SES (income) | Categorized as a) Very low; b) Low; c) In the middle; d) High; and e) Very high | Perceived |  | | Higher income was a significant predictor of lower anxiety (β = -.039, p<.001); |  | Higher income was a significant predictor of lower depression (β = -.056, p= .024); | |  |
|  |  | Employment | Categorized as a) Work with government; b) Students; c) Private business; d) Retired; and e) Unemployed | Actual |  | | Non-significant |  | Non-significant | |  |
|  |  |  |  |  |  | |  |  |  | |  |
| #3023 | 8 | Employment stressor | Categorized as a) Worked fewer hours/shifts; b) Salary/benefits were cut/decreased; c) Temporarily furloughed; and d) Laid off/lost a job permanently) | Actual | PHQ-4 | | Non-significant | PHQ-4 | Non-significant | |  |
|  |  |  |  |  |  | |  |  |  | |  |
|  |  | Financial stressor | Categorized as worried about not being able to pay a) Medical costs of a serious illness or accident; b) Medical costs for normal or routine health care; and c) Not being able to afford basic household expenses such as rent/mortgage or utilities. | Perceived |  | | For both men and women the odds of anxiety were greater for those who reported financial stressors (OR=2.64 for women, OR=2.94 for men) |  | For both men and women the odds of depression were greater for those who reported financial stressors (OR=2.50 for women, OR=2.92 for men) | |  |
|  |  |  |  |  |  | |  |  |  | |  |
|  |  | Food insecurity | Categorized as a) “I worried whether my food would run out before I got money to buy more”; and b) “The food that I bought just didn’t last, and I didn’t have money to get more” | Perceived |  | | Non-significant |  | Non-significant | |  |
|  |  |  |  |  |  | |  |  |  | |  |

**Table 5 (Continued)**

*The Main Outcomes of the Studies Included in the Systematic Review*

| Study ID | Quality Assessment | SEC  Variable | Measurement  Method | Actual / Perceived | | Anxiety    Inventory Outcomes | | Depression    Inventory Outcomes | | Note | |
| --- | --- | --- | --- | --- | --- | --- | --- | --- | --- | --- | --- |
|  |  |  |  |  |  | |  |  |  | |  |
| #3009 | 6 | Working status (currently) | Categorized as a) I did not work; b) I worked mentally; c) I worked physically; and d) I ran my own business. | Actual | DASS-21 | | Non-significant  . | DASS-21 | Participants not working were associated with higher depression. | |  |
|  |  |  |  |  |  | |  |  |  | |  |
|  |  | Economic situation | Categorized as a) I have a stable family income, nothing has changed; b) I have a stable family income, but the situation is worse than before; c) I have to start using savings; d) I have to borrow money from my family/friends during the outbreak of the pandemic because I do not have enough money to support myself; and f) I barely have enough money for living. | Actual |  | | Participants in the “Stable family income, nothing has changed” had the lowest result in anxiety score. |  | Participants in the “Stable family income, nothing has changed” had the lowest result in depression score. | |  |
| #4928 | 8 | Education | Categorised as a) Lower than senior high school; b) Senior high school; c) Bachelor; d) Graduate degree | Actual | DASS-21 | | Non-significant | DASS-21 | Non-significant | |  |
|  |  |  |  |  |  | |  |  |  | |  |
|  |  | Family income | Categorised in USD as a) < 69, b) 69â€“345, c) 346 - 690, d) > 690 | Actual |  | | Non-significant |  | Non-significant | |  |
|  |  |  |  |  |  | |  |  |  | |  |
|  |  | Family expenses | Categorised as a) More than income; b) Similar with income; c) Less than income | Perceived |  | | Housewives and working mothers whose family spend more than the family income report more anxiety (housewives: p = .023, working mothers: p = .017). |  | Working mothers whose family spend more than the family income report more depression (p = .001). Housewives non-significant. | |  |
|  |  |  |  |  |  | |  |  |  | |  |
|  |  | Working status | Housewife or working mother | Actual |  | | Housewives report more anxiety than working mothers (p < .001) |  | Housewives report more depression than working mothers (p < .001) | |  |
|  |  |  |  |  |  | |  |  |  | |  |

**Table 5 (Continued)**

*The Main Outcomes of the Studies Included in the Systematic Review*

| Study ID | Quality Assessment | SEC  Variable | Measurement  Method | Actual / Perceived | | Anxiety    Inventory Outcomes | | Depression    Inventory Outcomes | | | Note | | |
| --- | --- | --- | --- | --- | --- | --- | --- | --- | --- | --- | --- | --- | --- |
|  |  |  |  |  |  | |  |  | |  | |  | |
| #56 | 11 | Employment Status | Categorized as a) Employed; b) Out of work; c) Homemaker; d) Student; and e) Retired | Actual | GAD-7 | | Non-significant | | NA | NA | | | Please see Table 4 for T2 |
|  |  |  |  |  |  | | Non-significant | |  | NA | | |  |
|  |  | Income | Categorized as a) < $50,000; b) $50,000 to $ 90,000; and c) >=$100,000 | Actual |  | |  | |  |  | | |  |
|  |  |  |  |  |  | |  | |  |  | | |  |
|  |  | Income loss | Unclear | Unclear |  | | Having lost income due to COVID-19 was positively associated with moderate or severe anxiety symptoms (aPR = 1.27). | |  | NA | | |  |
| #6487 | 6 | Income | Categorised as a) <500 JOD; b) 500-1000 JOD; and c) >1000 JOD | Actual | K10 | | Monthly income (<500 JOD) was associated with higher odds of severe mental disorder (OR = 3.6, p = 0.01) | | K10 | Monthly income (<500 JOD) was associated with higher odds of severe mental disorder (OR = 3.6, p = 0.01) | | |  |
|  |  | Employment status | Categorised as a) Unemployed; b) Students; c) Self-employed; and d) Retired | Actual |  | | Employment status (unemployed) was associated with higher odds of severe mental disorder (OR = 2.4, p = 0.001) | |  | Employment status (unemployed) was associated with higher odds of severe mental disorder (OR = 2.4, p = 0.001) | | |  |
| #6296 | 8 | Income loss | A binary response (Yes/No) to whether their income had been adversely affected due to the COVID-19 pandemic | Perceived | GAD-7 | | Income loss (p=.01) was found to be a predictor of anxiety | | NA | NA | | |  |
|  |  |  |  |  |  | |  | |  |  | | |  |
